# Oak genome reveals facets of long lifespan

**DOI:** 10.1038/s41477-018-0172-3

**Published:** 2018-06-18

**Authors:** Christophe Plomion, Jean-Marc Aury, Joëlle Amselem, Thibault Leroy, Florent Murat, Sébastien Duplessis, Sébastien Faye, Nicolas Francillonne, Karine Labadie, Grégoire Le Provost, Isabelle Lesur, Jérôme Bartholomé, Patricia Faivre-Rampant, Annegret Kohler, Jean-Charles Leplé, Nathalie Chantret, Jun Chen, Anne Diévart, Tina Alaeitabar, Valérie Barbe, Caroline Belser, Hélène Bergès, Catherine Bodénès, Marie-Béatrice Bogeat-Triboulot, Marie-Lara Bouffaud, Benjamin Brachi, Emilie Chancerel, David Cohen, Arnaud Couloux, Corinne Da Silva, Carole Dossat, François Ehrenmann, Christine Gaspin, Jacqueline Grima-Pettenati, Erwan Guichoux, Arnaud Hecker, Sylvie Herrmann, Philippe Hugueney, Irène Hummel, Christophe Klopp, Céline Lalanne, Martin Lascoux, Eric Lasserre, Arnaud Lemainque, Marie-Laure Desprez-Loustau, Isabelle Luyten, Mohammed-Amin Madoui, Sophie Mangenot, Clémence Marchal, Florian Maumus, Jonathan Mercier, Célia Michotey, Olivier Panaud, Nathalie Picault, Nicolas Rouhier, Olivier Rué, Camille Rustenholz, Franck Salin, Marçal Soler, Mika Tarkka, Amandine Velt, Amy E. Zanne, Francis Martin, Patrick Wincker, Hadi Quesneville, Antoine Kremer, Jérôme Salse

**Affiliations:** 1 BIOGECO, INRA, Université de Bordeaux, Cestas, France; 2 Commissariat à l’Energie Atomique (CEA), Genoscope, Institut de Biologie François-Jacob, Evry, France; 3 URGI, INRA, Université Paris-Saclay, Versailles, France; 4 GDEC, INRA-UCA, Clermont-Ferrand, France; 5 IAM, INRA, Université de Lorraine, Champenoux, France; 6 HelixVenture, Mérignac, France; 7 INRA, US 1279 EPGV, Université Paris-Saclay, Evry, France; 8 BIOFORA, INRA, Orléans, France; 9 AGAP, Université de Montpellier, CIRAD, INRA, Montpellier SupAgro, Montpellier, France; 10 Department of Ecology and Genetics, Evolutionary Biology Centre, Science for Life Laboratory, Uppsala University, Uppsala, Sweden; 11 CIRAD, UMR AGAP, Montpellier, France; 12 Université de Montpellier, CIRAD, INRA, Montpellier SupAgro, Montpellier, France; 13 CNRGV, INRA, Castanet, France; 14 UMR Silva, INRA, Université de Lorraine, AgroPariTech, Nancy, France; 15 Department of Soil Ecology, UFZ–Helmholtz Centre for Environmental Research, Halle/Saale, Germany; 16 Plateforme bioinformatique Toulouse Midi-Pyrénées, INRA, Auzeville Castanet-Tolosan, France; 17 Université de Toulouse, CNRS, UMR 5546, LRSV, Castanet-Tolosan, France; 18 German Centre for Integrative Research (iDiv), Halle–Jena–Leipzig, Leipzig, Germany; 19 SVQV, Université de Strasbourg, INRA, Colmar, France; 20 Université de Perpignan, UMR 5096, Perpignan, France; 21 Laboratori del Suro, University of Girona, Girona, Spain; 22 Department of Biological Sciences, George Washington University, Washington, DC USA; 23 Génomique Métabolique, Genoscope, Institut de Biologie François-Jacob, Commissariat à l’Energie Atomique (CEA), CNRS, Université d’Evry, Université Paris-Saclay, Evry, France

**Keywords:** Plant evolution, Plant immunity, Genomics, Sequencing, Forestry

## Abstract

Oaks are an important part of our natural and cultural heritage. Not only are they ubiquitous in our most common landscapes^1^ but they have also supplied human societies with invaluable services, including food and shelter, since prehistoric times^2^. With 450 species spread throughout Asia, Europe and America^3^, oaks constitute a critical global renewable resource. The longevity of oaks (several hundred years) probably underlies their emblematic cultural and historical importance. Such long-lived sessile organisms must persist in the face of a wide range of abiotic and biotic threats over their lifespans. We investigated the genomic features associated with such a long lifespan by sequencing, assembling and annotating the oak genome. We then used the growing number of whole-genome sequences for plants (including tree and herbaceous species) to investigate the parallel evolution of genomic characteristics potentially underpinning tree longevity. A further consequence of the long lifespan of trees is their accumulation of somatic mutations during mitotic divisions of stem cells present in the shoot apical meristems. Empirical^4^ and modelling^5^ approaches have shown that intra-organismal genetic heterogeneity can be selected for^6^ and provides direct fitness benefits in the arms race with short-lived pests and pathogens through a patchwork of intra-organismal phenotypes^7^. However, there is no clear proof that large-statured trees consist of a genetic mosaic of clonally distinct cell lineages within and between branches. Through this case study of oak, we demonstrate the accumulation and transmission of somatic mutations and the expansion of disease-resistance gene families in trees.

## Abstract (web summary)

Oaks can live hundreds of years. Comparative genomics using a high-quality genome sequence provides new insights that may explain tree longevity. Samples from branches and corresponding acorns also help quantify heritable somatic mutations.

## Main

**Fig. 1:**
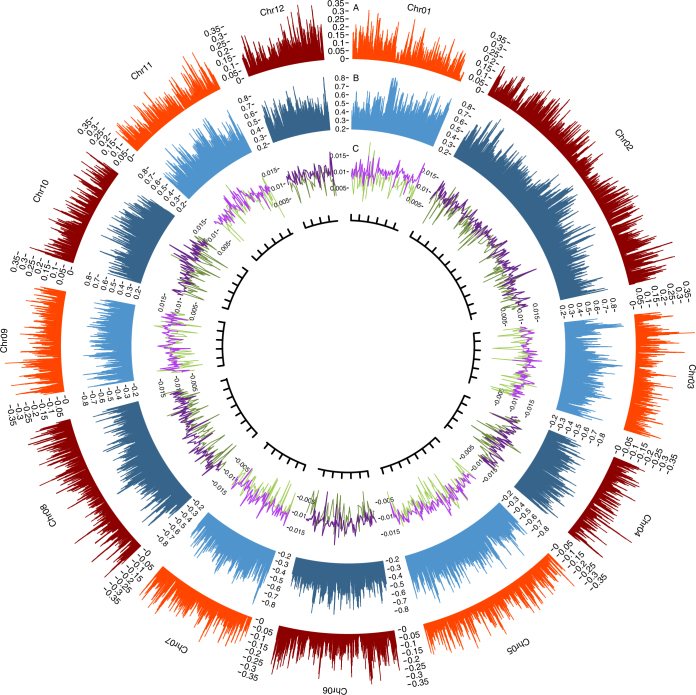
Genomic landscape of the 12 assembled oak chromosomes. Gene (A) and TE (B) density, percentage heterozygosity (purple in C) and genetic diversity (green in C). These four metrics are calculated in 1-Mb sliding windows, moved in 250-kb steps. A ruler is drawn on each chromosome, with tick marks every 10 Mb.

**Fig. 2:**
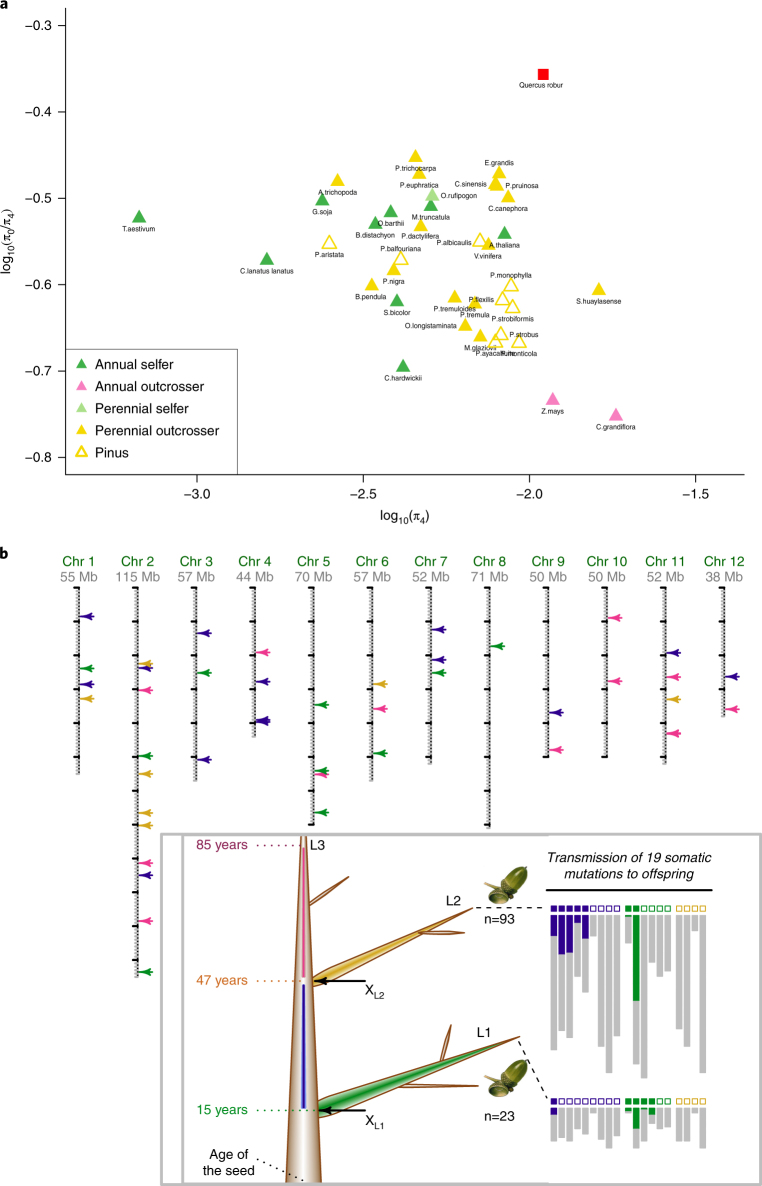
Genetic diversity and somatic mutations. **a**, Distribution of π_0_/π_4_ plotted against π_4_ among plants (modified from a previous publication^9^), including oak (red square). Species names are according to^9^. **b**, Genomic location of somatic mutations along the 12 chromosomes of a 100-year-old oak tree. Mutations are represented as coloured arrows according to where they took place during tree growth (see inset). Location and age (left of the trunk) of the three levels (L1, L2 and L3) sampled for somatic mutation detection in the reference pedunculate oak genotype 3P. L1, L2 and L3 represent the end of selected branches; X_L1_ and X_L2_ represent L1-branch and L2-branch initiation sites, respectively. For each branch, the recovery or non-recovery of mutations in acorns is indicated by filled and open squares, respectively. The numbers of copies of the alternative (coloured) and reference (grey) alleles are shown below each square.

We sequenced the highly heterozygous genome of pedunculate oak (*Quercus robur* L.; Supplementary Notes 1 and 2) using a combination of long and short sequence reads (Supplementary Table 1). We generated a highly contiguous haploid genome sequence of a heterozygous tree comprising 1,409 nuclear scaffolds, with an N50 of 1.35 Mb (Supplementary Note 2.2, Supplementary Table 2, Supplementary Fig. 1). A comparison with existing tree genomes is shown in Supplementary Table 3. In total, 871 scaffolds, covering 96% (716.6 Mb) of the estimated physical size of the oak genome and containing 90% of the 25,808 predicted protein-coding genes (Supplementary Data Set 1, Supplementary Note 3.3), were anchored to the 12 oak chromosomes. To this end, we used the existing high-density oak gene-based linkage map^8^ combined with a synteny-driven approach using *Prunus persica* as a pivotal genome. Non-anchored scaffolds harbouring genes syntenic to peach were placed on the pseudomolecules based on the local microsynteny identified between oak and peach (Fig. 1, Supplementary Note 2.3, Supplementary Fig. 2, Supplementary Data Set 2). Overall, 52% of the genome was found to consist of diverse transposable elements (TEs), which were dominated by class I retrotransposons (70%) (Supplementary Table 4, Supplementary Fig. 3, Supplementary Notes 3.1 and 3.4). Genome-wide genetic diversity, as assessed by an analysis of single-nucleotide polymorphisms (SNPs) at the individual level (heterozygosity rate) and using a population of 20 genotypes (π), amounted to ~1%, with significant variation within and between chromosomes (Fig. 1, Supplementary Fig. 4). Nucleotide diversity in protein-coding genes was 0.011 for fourfold degenerate sites and 0.005 for non-degenerate sites, with a non-synonymous-to-synonymous nucleotide diversity ratio (π_0_/π_4_) of 0.44. A comparison of these values with those obtained in a recent survey of plant and animal species^9^ indicated that oak was remarkable in terms of both its high nucleotide diversity (π_4_) and the high rate at which it accumulates deleterious mutations (Fig. 2a, Supplementary Note 4.1). Indeed, the value for oak shows the largest deviation from the regression line, with the largest residual (0.25) compared with the other 37 plant species (ranging from −0.13 to 0.12).

In addition to the spontaneous meiotic mutations in each generation, long-lived plants are expected to accumulate somatic mutations throughout their lifetime. These mutations occur during the mitotic divisions of stem cells in the shoot apical meristems^4^. In trees, unlike animals, these mutations can be passed from the soma to the reproductive tissue and on to the offspring. Somatic mutations may therefore increase genetic diversity in long-lived trees such as oaks. Oaks have weak apical control (that is, an inability to control the flushing and growth of lateral buds from the previous year^10^), resulting in a multi-stemmed morphology. As such, oaks constitute a particularly appropriate model for studies of the somatic generation of diversity. We sampled buds at the extremities of branches initiated at the ages 15, 47 and 85 years on the reference tree sequenced in this study (Fig. 2b, Supplementary Fig. 5). Using a frequency-dependent method for detecting somatic point mutations in genomic DNA^11^, we identified 46 reliable somatic mutations (Supplementary Note 4.2, Supplementary Table 5) most of which (44) were located on scaffolds anchored to the 12 chromosomes (Fig. 2b). Compared with a recent report that also used the pedunculate oak as a model system^12^, we detected 2.7 times more somatic mutations on a tree that was 3 times younger. This difference is probably due to our superior ability to detect somatic mutations on a higher fraction of the genome (owing to the quality of our genome assembly) and smaller changes in allele frequency by applying a frequency-explicit method. This method was developed for cancer research and, in our case, accounts for the mosaic of mutated and non-mutated stem cells in shoot apical meristems. Given that most somatic mutations have a low allele frequency (1/2*N* stem cells) during growth^13^, most somatic mutations are expected to remain at frequencies too low to be unambiguously detected. Thus, while this work provides clear evidence that somatic mutations exist in trees, it still remains particularly challenging to determine the actual rate of somatic mutations. Consequently, we consider that the number of somatic mutations identified in the studied genotype reported here is only the tip of the iceberg of the total number of somatic mutations. A previous study^12^ formulated an interesting working hypothesis whereby stem cell mutagenesis protects shoot apical meristems against ultraviolet damage. This hypothesis was based on the discrepancy between theoretical expectations and the low number of empirically identified somatic mutations. However, considering the detection bias for low allele frequency variants, the hypothesis remains unsupported even with the best genomic data available to date. We then investigated the transmission of mutations to the offspring by evaluating a subset of 19 somatic mutations (Supplementary Table 6) in 116 acorns collected from the extremities of lateral branches (Fig. 2b). Despite the limited number of seeds collected, we recovered 47% (9/19) of the somatic mutations in the embryonic tissues of the acorns, confirming intergenerational transmission (Fig. 2b). Our work demonstrates that somatic mutations exist in oak and are passed onto the next generation. However, our results do not allow conclusions to be drawn on the contribution of somatic mutations to the high genetic diversity level and large-scale evolution of oaks.

**Fig. 3:**
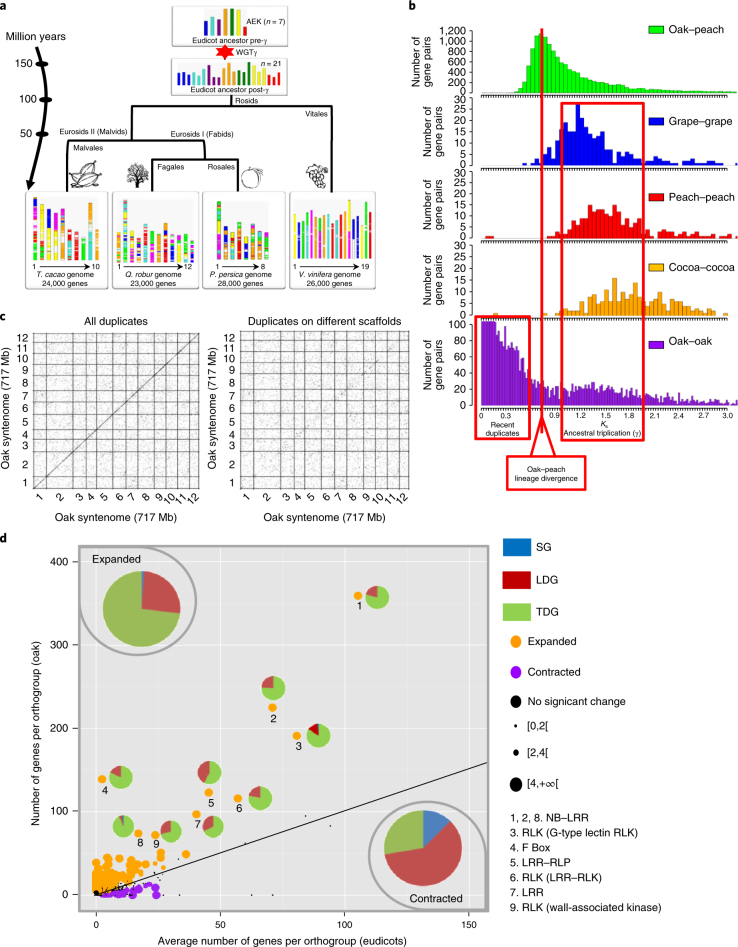
Evolutionary history of the oak genome. **a**, Evolutionary scenario of oak from the AEK of 21 (post-γ) and 7 (pre-γ) protochromosomes reconstructed from a comparison of the Vitales (grape), Rosales (peach) and Malvales (cocoa) major subfamilies. The modern genomes (bottom) are illustrated with different colours reflecting the seven ancestral chromosomes of AEK origin (top). WGT (red star) refers to the whole-genome triplication (γ) shared among the eudicots. **b**, *K*_s_ distribution of gene pairs for oak–peach orthologues as well as the shared γ triplication in grape, peach, cocoa and oak. *K*_s_ distribution of all gene pairs in oak illuminate gene pairs from the γ triplication as well as recent duplicates. **c**, Dot plot representation of the oak genome against itself for the complete set of paralogous pairs (left) and without TDGs (right) representing the disappearance of the diagonal (TDGs) when low *K*_s_ values are removed. **d**, Expansion (524 orthogroups) and contraction (72 orthogroups) in oak relative to 15 other eudicot species. The pie charts reflect the contribution of TDGs, LDGs and singleton genes (SGs) to the significantly expanded and contracted orthogroups and to outstanding outliers (labelled 1–9). Numbers in square brackets associated with circle sizes stand for -log(*P-*value), computed from the oak branch-specific *P*-value provided by CAFE.

We searched for genomic features specific to oak that might contribute to its longevity by first reconstructing its paleohistory within the rosid clade. We compared the ancestral eudicot karyotype (AEK^14^) reconstructed from a comparison of the Vitales (grape^15^), Rosales (peach^16^) and Malvales (cocoa^17^) major subfamilies to reveal that oak experienced 5 fissions and 14 fusions from 21 AEK^18^ chromosomes to reach the modern 12 chromosomes (Fig. 3a). The synonymous substitution rate (*K*_s_) of paralogues (Fig. 3b) indicated that oak did not experience lineage-specific whole-genome duplication in addition to the ancestral triplication shared among the eudicots (γ^19^). We also found that oak experienced a recent burst of local gene duplications (accounting for 35.6% of the oak gene repertoire) after the oak–peach lineage diverged (Fig. 3b). The eucalyptus genome is the only other plant genome shown to date to display such high levels of tandem duplication^20^ (34%), contrasting strongly with the other four genomes investigated (<25% tandem duplicates). We next validated that recent tandemly duplicated genes (TDGs) were true duplicates rather than different alleles or duplication artefacts generated during haplome construction (that is, during the scaffolding or merging steps of our hierarchical assembly pipeline). To this end, we applied two verification procedures based on a comparison of polymorphisms of allelic gene pairs (Supplementary Fig. 22) and a sequence coverage analysis (Supplementary Fig. 23).

A comparison of gene families (36,844 orthogroups, including 435,095 genes from 16 plant species (Supplementary Table 7)) provided further clues to the functional significance of tandem duplications. Of the 524 orthogroups found to have undergone expansion in oak relative to the other 15 species (Supplementary Data Set 3), 73% of the genes of concerned were tandem duplicates (Supplementary Data Set 4). Such a tight relationship between TDGs and lineage-specific selection is not a novel observation^21^, and it seems to be particularly common for disease-resistance (R) genes^22^. However, the higher frequency of such relationships in long-lived plants, such as oak and eucalyptus, suggests that there may be a convergent mechanism in trees towards an expansion of these families of genes in long-lived species.

**Fig. 4:**
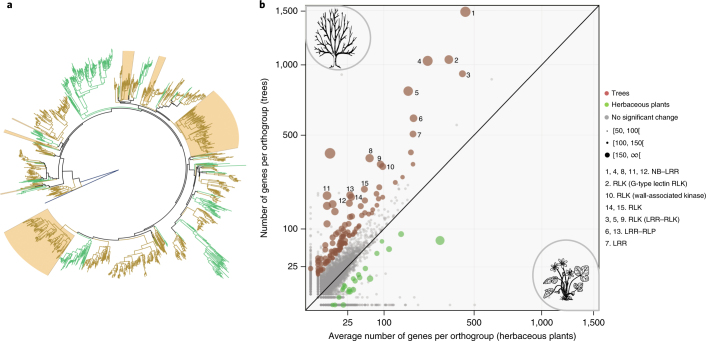
Expanded gene families in trees. **a**, Phylogeny of orthogroup 1 from Figs. 3d and 4b, established from the nucleotide-binding domains of 1,641 NB–LRR genes. Branches for trees and herbaceous species are shown in brown and green, respectively. Branches expanded in oak are shaded. For a higher resolution image see Supplementary Fig. 6. **b**, Scatter plot showing orthogroups expanded in trees and herbaceous plants (images from http://openclipart.org). Numbers in square brackets associated with circle sizes stand for -log(*P*-adjust), where *P*-adjust is the *P*-value of the binomial test adjusted for multiple testing.

The orthogroups expanded in oaks are clearly enriched in Gene Ontology (GO) terms relating to biotic interactions. They included 95% of the 1,091 nucleotide-binding site leucine-rich repeat (NB–LRR)-related protein genes and 55% of the 1,247 receptor-like kinase (RLK)-encoding genes (Supplementary Data Sets 5 and 6, Supplementary Table 8, Supplementary Notes 3.5.6 and 3.5.7). We detected a particularly strong expansion of two major clades of toll interleukin receptor (TIR)–NB–LRRs in orthogroup 1 (shaded areas in Fig. 4a and Supplementary Fig. 6). In addition, three of the nine orthogroups displaying the strongest expansions (Fig. 3d, Supplementary Data Set 3) corresponded to intracellular receptors (NB–LRRs for orthogroups 1, 2 and 8) and four corresponded to cell surface receptors of the innate immune response (RLKs for orthogroups 3, 6 and 9, and LRR–receptor-like protein (RLP) for orthogroup 5). The entire complement of NB–LRR and RLK genes accounted for 9% of all oak genes, a proportion that is approximately twice that reported for other plants^23,24^. Moreover, 75% and 65% of the NB–LRR and RLK expansions, respectively, can be accounted for by tandem duplications. The distribution of the LRR–RLK genes between the established subgroups based on an analysis of 31 angiosperms^25^ also revealed remarkable expansions, with subgroup XIIa (shown as orthogroup 6 in Fig. 3d) and subgroup XIIb harbouring the highest global expansion rates in oak. That is, 102 copies for subgroup XIIa and 50 copies for subgroup XIIb, corresponding to an expansion rate of 11.3-fold and 12.5-fold, respectively. Subgroup XIIa (containing, for example, flagellin-sensitive 2 (FLS2), EF-TU receptor (EFR) and Xa21) and subgroup XIIb (containing Xoo-induced kinase 1 (XIK1), for example) included receptors known to play a role in the response to bacterial infections^26^. The orthogroups expanded in oaks also presented a significantly (*P* < 2 × 10^−16^) higher π_0_/π_4_ ratio than contracted or stable orthogroups (Supplementary Table 9). Moreover, the efficacy of purifying selection was remarkably low for the NB–LRR and RLK gene families, with mean π_0_/π_4_ ratios of 0.68 and 0.58, respectively (Supplementary Note 4.1).

The enrichment of gene families relating to receptor-mediated signalling in oak led us to investigate whether similar enrichment had occurred in other trees. To this end, we compared trees and herbaceous species among the 16 plant genomes investigated. In eudicots, each distinct tree lineage provides an independent evolutionary experiment for investigating the genomic features relating to the tree lifestyle^27^. We found that 126 of the 36,844 orthogroups had undergone tree-specific expansion (Fig. 4b, Supplementary Data Set 7). These orthogroups were enriched in 61 GO terms, largely (63%) related to plant immunity (Supplementary Data Set 8, Supplementary Fig. 7). Ten of the 15 gene families displaying striking expansion in tree genomes (Fig. 4b) corresponded to NB–LRRs (orthogroups 1, 4, 8, 11 and 12), LRR–RLKs (orthogroups 3 (subgroup XIIb), 5 (subgroup XIIa) and 9) or LRR–RLPs (orthogroups 6 and 13). A phylogenetic analysis of the orthogroup most strongly expanded in trees (orthogroup 1 in Figs. 3d and 4b) clearly highlighted the expansion of TIR–NB–LRRs in woody perennials relative to herbaceous species (Fig. 4a, Supplementary Fig. 6). Several TIR–NB–LRR genes from this cluster are involved in the perception of bacterial or oomycete pathogens in *Arabidopsis* (for example, *Rps4* or *Rpp5*^28,29^). We also investigated the adaptive value of R genes within expanded orthogroups, making use of a recent meta-analysis of these membrane-bound receptor genes in 31 angiosperm genomes^25^. We isolated 24 groups of oak lineage-specific expanded LRR–RLK paralogues and explored footprints of positive selection (Supplementary Data Set 9) based on the divergence between paralogous copies. In total, 19 groups (80%) had a significant signature of positive selection, with similar proportions reported for only two other tree species (*Malus*, 73% and *Populus*, 87%). We identified 260 sites subject to positive selection after the manual curation of protein sequence alignments in oak. More than 78% of these sites were located in LRR domains. As reported in a previous study^25^, positive selection mostly targeted four amino acids of the hypervariable region of the characteristic LXXLXLXX β-sheet/β-turn structure of LRRs (Supplementary Fig. 8), which has been implicated in protein–protein interactions^30^. The high proportion of sites under positive selection in this domain therefore confirms the amino acid sequence diversification of these genes through fixation of amino acid changes.

In an opinion article^31^, it was suggested that the following three non-exclusive mechanisms could allow plants “to grow old without antibodies”: numerous and highly diversified defence genes; favoured expansion of R gene families; and accumulation of somatic mutations, which can be transmitted to the next generation. Our study tackles all three genomic features that may contribute to the success of long-lived trees and finds support for all three suggested mechanisms.

In conclusion, we sequenced the oak genome and revealed its considerable genetic diversity, to which heritable somatic mutations may contribute. This work poses new research questions about the contribution of this mutational load in adaptation, in particular with regard to defences against new pests and pathogens. We also showed that the genome of this iconic tree went through a single paleohexaploidization event (γ, shared among the eudicots), followed by a massive burst of recent local gene duplication. These duplications have amplified families of genes involved in defence against pathogens. We observed a parallel expansion of R gene-related gene families across multiple tree species, suggesting that the immune system makes an essential contribution to the survival of long-lived plants over several centuries. The remarkable relaxation of purifying selection observed in oaks may facilitate the evolution of a richer and more diverse set of R genes, thereby conferring an advantage on these trees in their continuous arms race with pathogens^32^. This dynamic is likely to apply particularly to oaks, with their remarkably long lifespan. However, the maintenance of such a diversity of R genes may be costly, and future studies should look at how trees control the expression of these immune receptors, through microRNA control, for example^22^.

## Methods

### Tree material

Pedunculate oak *(Q. robur* L., 2*n* = 2× = 24) is an outcrossing, highly heterozygous diploid species. Flow cytometry analysis has shown that this species has a genome of 740 Mb per C^33^, where the C-value is the amount, in picograms, of DNA contained within a haploid nucleus. The “3P” accession selected for establishment of the reference genome sequence for pedunculate oak is a tree of ~100 years of age located at the INRA Pierroton forestry research station (Aquitaine, France; 44 °44′ N, 00 °46′ W). This tree has already been characterized at the genetic^34,35^ and genomic^36,37^ levels. The tree (used as a female parent) has also been crossed with accession A4 (used as a male parent) to generate a full-sibling progeny for studies of the genetic architecture of quantitative traits^38–47^. A graft copy of 3P was placed in darkness in July 2009, to trigger the release of as much starch as possible from second-flush leaves, in an in-house procedure that has been shown to improve the quality of DNA extraction from oak leaves. We harvested 140 g of etiolated leaves and stored them at −80 °C before DNA extraction.

### DNA sample preparation for reference genome sequencing

An Invisorb Spin Plant Mini Kit (Stratec Molecular) was used to isolate genomic DNA and prepare short-read libraries for the Roche-454 and Illumina sequencing platforms. DNA concentrations were determined using a Quant-iT dsDNA Assay Kit (Life Technologies) and a Qubit Fluorometer (Invitrogen). We checked the integrity of the genomic DNA by agarose gel electrophoresis and pulsed-field gel electrophoresis. Agarose-embedded high-molecular weight (HMW) DNA was prepared as described previously^48^, and modified as described previously^49^, to construct Illumina TruSeq Synthetic Long Read (TSLR) libraries. Agarose gel plugs were washed three times in Tris EDTA buffer and subjected to digestion with 8 U of β-agarase (New England Biolabs) for 12–16 h at 42 °C. HMW DNA was then drop-dialysed for 2.5 h. DNA concentrations were quantified with the Quant-iT dsDNA Assay Kit. DNA quality was then checked using an Argus Qcard Kit (OpGen) and was estimated at 20–100 kb.

### Sequencing

We prepared 454 single-end read libraries according to the standard procedure provided by Roche, with RL adaptors (GS FLX Titanium Rapid Library Preparation Kit; Roche Diagnostic). The libraries were sequenced with titanium chemistry on a 1/2 Pico Titer Plate on a 454 GS FlX instrument (Roche Diagnostic).

Illumina overlapping and tightly sized paired-end libraries were prepared using a semi-automated protocol. Briefly, genomic DNA (250 ng) was sheared using a Covaris E210 instrument (Covaris) to generate fragments of 150–400 bp or 200–800 bp in size for the overlapping and tightly sized libraries, respectively. End repair, A-tailing and ligation with Illumina-compatible adaptors (Bioo Scientific) were performed using a SPRIWorks Library Preparation System and a SPRI-TE instrument (Beckmann Coulter) according to the manufacturer’s protocol. We selected fragments of 200–400 bp or 300–600 bp in size for the overlapping and tightly sized libraries, respectively. DNA fragments were then amplified by 12 cycles of PCR with Pfx Platinum Taq polymerase (ThermoFisher) and Illumina adapter-specific primers. We selected amplified library fragments of ~300 bp in size by electrophoresis in a 3% agarose gel for the overlapping libraries. For tightly sized libraries, fragments of ~600 bp in size were selected by electrophoresis in a 2% agarose gel.

The 3-kb mate–pair library was prepared according to the initial Illumina protocol (Illumina Mate Pair Library Kit), with ~10 µg of genomic DNA subjected to Covaris fragmentation in the first step. The other mate–pair libraries were prepared using a Nextera Mate Pair Sample Preparation Kit (Illumina). Briefly, genomic DNA (4 µg) was simultaneously fragmented by enzymatic treatment and tagged with a biotinylated adaptor. The resulting fragmented and tagged (tagmented) DNA was subjected to size selection (3–5, 5–8 and 8–11 kb) by regular gel electrophoresis, and circularized by overnight incubation with a ligase. Linear, non-circularized fragments were digested and circularized DNA was fragmented to generate fragments of 300–1,000 bp in size with the Covaris E210 system. Biotinylated DNA was immobilized on streptavidin beads, end-repaired, then 3′-adenylated, and Illumina adapters were added. DNA fragments were amplified by PCR with Illumina adapter-specific primers and purified.

All Illumina library traces were evaluated using an Agilent 2100 Bioanalyzer (Agilent Technologies) and quantified by quantitative PCR using a KAPA Library Quantification Kit (KapaBiosystems) on a MxPro instrument (Agilent Technologies). Libraries were then sequenced as described in Supplementary Table 1.

Finally, 39,092 BACs (corresponding to a physical coverage of 3.5×, Supplementary Note 2.1) were end-sequenced with dye terminator chemistry using an ABI 3730 sequencer (Applied Biosystems) as described previously^50^. The sequences can be obtained from GenBank (accession numbers HN154083–HN174138, JS673272–JS676292, JS676293–JS684825 and FO926004–FO981373).

We prepared 14 libraries (Supplementary Table 1) from 5 different extracted HMW DNA samples with TSLR technology (previously known as Moleculo) according to the Illumina protocol. Briefly, genomic DNA (500 ng) was sheared into fragments of approximately 10 kb in size with g-Tube (Covaris). The fragments were subjected to end repair, A-tailing and adaptor ligation, and the ligated products were size-selected by gel electrophoresis to obtained fragments of 8–10 kb in size, which were quantified by qPCR. The long-insert library was then diluted such that each well of a 384-well plate contained 3 fg of the library. The diluted products were subjected to long-range PCR, tagmentation and barcoding with 384 different barcoding PCR primers. The 384 barcoded libraries were pooled, purified and subjected to size selection. Each library was sequenced by 100 or 150 base-length read chemistry instrument (Illumina).

### Sequence processing

Raw Roche/454 reads were used for subsequent analyses without processing. Illumina paired-end and mate–pair reads were cleaned in the following three-step procedure: sequencing adapters and low-quality nucleotides (quality value <20) were removed; sequences between the second unknown nucleotide (N) and the end of the read were removed; and reads shorter than 30 nucleotides after trimming were discarded, together with reads and their mates mapping onto run quality control sequences (PhiX genome). The TSLRs were generated using the BaseSpace workflow. The primary sequencing data were then uploaded without modification to the BaseSpace cloud and processed using the standard Illumina workflow to generate long synthetic reads.

### Genome size estimation by k-mer analysis

Before assembly, we analysed the k-mer distribution of Illumina 100-bp paired-end reads (two lanes representing 95-fold coverage of the haploid genome) to obtain an independent estimate of the haploid size of the oak genome. The 31-mer distribution was generated using Jellyfish^51^ (with the following parameters: -m 31 -s 2048M -C) and was uploaded to the GenomeScope website (http://qb.cshl.edu/genomescope/). We obtained an estimated haploid genome size of 736 Mb (Supplementary Fig. 25), a value close to the 740 Mb estimated by flow cytometry^33^.

### Genome assembly

We first assembled the longest reads together (obtained from 454 and Moleculo libraries) to maximize the separation of the two haplotypes of accession 3P and to overcome the high level of heterozygosity. We used Newbler and Celera^52^ as the overlap-layout-consensus (OLC) assemblers. We used Newbler software (version MapAsmResearch-04/19/2010-patch-08/17/2010) with default parameters, with the addition of the -large and -sio options. As Newbler does not accept reads longer than 2 kb, we split Moleculo reads into overlapping 1,999-bp fragments (with overlaps of 1,499 bp) and retained the origin of each fragment for further analysis (see next section). We obtained an assembly (named A1 in Supplementary Table 10) of 300,113 contigs with an N50 of 9.3 kb and a cumulative size of 1.31 Gb, corresponding roughly to the size of the two haplotypes. We ran Celera with the following parameters: unitigger=bogart; merSize=31; merThreshold=auto*2; ovlMinLen=800; obtErrorRate=0.03; obtErrorLimit=4.5; ovlErrorRate=0.03; utgErrorRate=0.015; utgGraphErrorRate=0.015; utgGraphErrorLimit=0; utgMergeErrorRate=0.03; batThreads=20; utgMergeErrorLimit=0. This process produced an assembly (named C1 in Supplementary Table 11) composed of 29,255 contigs with an N50 of 9.5 kb and a cumulative size of 1.31 Gb. The Celera assembler allows the direct input of raw Moleculo reads and we performed the scaffolding (that is, ordering and orienting of contigs) step directly on the Celera contigs of the C1 assembly.

### Use of long reads to simplify the contig graph

Once the initial Newbler assembly was obtained, we used long-range information from Moleculo reads to simplify the contig graph. The Newbler output file “454ContigGraph.txt” describes the contig graph, in which the nodes are contigs and the edges are links between two contigs spanned by a read. Contigs were generally fragmented due to the presence of repeat or heterozygous regions. We extracted links between the contigs created from different parts of single long reads. Finally, a file containing all the links was generated (in DE format) and used as input for the string graph assembler (SGA) scaffolding module^53^. We obtained an assembly (named A2 in Supplementary Table 10) composed of 198,695 contigs with a N50 of 16.2 kb and a cumulative size of 1.33 Gb.

### Scaffolding step

We used Illumina paired-end and mate–pair libraries to organize contigs and to produce scaffolds. We ran three iterations of the SSPACE scaffolder^54^ with the parameters -k 5 and -a 0.7, using the following libraries, ranked by increasing fragment size: 400-bp paired-end, 3-kb mate–pairs, 5-kb mate–pairs and 8-kb mate–pairs. We then ran SSPACE again, with -k 2 and -a 0.7, using the Sanger BAC-ends and the previously scaffolded assembly. Sanger reads were transformed into Illumina-like reads by selecting the 100-bp window with the highest quality according to Sickle software^55^. We obtained two assemblies (A3 and C2 in Supplementary Tables 10 and 11, respectively). The most contiguous of these assemblies (A3) consisted of 9,025 scaffolds with an N50 of 818 kb and a cumulative size of 1.45 Gb (including 11.19% ambiguous bases).

### Choice of the final assembly

The choice of the final assembly was based on the metrics of the two assemblies obtained with Celera and Newbler (assemblies C2 and A3) and comparisons with high-quality BACs (see Supplementary Note 2.1.3 and examples in Supplementary Fig. 9). We chose the Newbler assembly because it better discriminated between the two haplotypes.

### Gap filling

The scaffold gaps of the A3 assembly were closed with GapCloser software^56^ and Illumina paired-end reads. As input, we used 95× coverage (of the haploid genome) of overlapping paired-end reads and 95× coverage (of the haploid genome) of a standard paired-end library (400–600-bp fragments). We obtained an assembly (named A4 in Supplementary Table 10) consisting of 9,025 scaffolds with an N50 of 821 kb and a cumulative size of 1.46 Gb (including 4.63% ambiguous bases).

### Bacterial decontamination

SNAP gene finder^57^ was applied to the entire assembly for draft gene prediction. We used an optimized calibration of SNAP based on the genewise alignment of *P. persica* coding sequences with the oak genome assembly. Predicted genes were then aligned against the NCBI NR database with BLAST-p. We kept the best match for each predicted protein and used the corresponding taxon. The 198 scaffolds containing >50% bacterial genes for the assigned proteins were considered to be putative contaminants and were removed from the assembly file (assembly A5 in Supplementary Table 10).

### Single-haplotype assembly

We used the Haplomerger v.1 pipeline^58^ to reconstruct allelic relationships in the released polymorphic diploid assembly and to reconstruct a reference haploid assembly. The diploid genome was first soft-masked with the following programs: TRF^59^ to mask tandem repeats; RepeatMasker^60^ to mask simple repeats, low-complexity and Viridiplantae-specific TEs; DUST^61^ to mask low-complexity sequences; and RepeatScout^62^ to mask unknown TEs. We then inferred a scoring matrix specific to the oak genome sequence, using 5% of the diploid assembly. The haploid genome was obtained from the soft-masked assembly and the specific scoring matrix with Haplomerger. We used the “selectLongHaplotype=1” parameter to maximize gene content as recommended in the Haplomerger documentation, as we knew this would generate frequent switches between haplotypes (Supplementary Fig. 11). We also prevented Haplomerger from creating false joins between scaffolds by using external information. We used the genetic linkage map (see Supplementary Note 2.3) and prevented Haplomerger from joining scaffolds from different linkage groups by modifying the “hm.new_scaffolds” file. We obtained an assembly (named H1, Supplementary Table 2) composed of 1,409 scaffolds with an N50 of 1,343 kb and a cumulative size of 814 Mb (including 2.94% ambiguous bases). We halved the size of the assembly, while retaining a completeness of gene content (evaluated with BUSCO^63^, similar to that of the diploid assembly, see Supplementary Table 2). The haploid scaffolds were aligned with BACs for visual inspection to determine the correctness of this final release (Supplementary Figs. 11, 12 and 13). A comparison with an existing heterozygous plant genome shows that our assembly ranks among the best for a series of metrics (number of contigs and scaffolds, scaffold N50 size; Supplementary Table 3). As introduced in Supplementary Note 2.3, a chromosome-scale genome was finally established using a high-density linkage map based on SNP markers^8^. We assessed the linear association between the genetic and physical positions of the SNPs using Spearman rank correlation.

### Detection and annotation of transposable element

The REPET pipeline (http://urgi.versailles.inra.fr/Tools/REPET) was used for the detection, classification (TEdenovo^64,65^) and annotation (TEannot^66^) of TEs. The TEdenovo pipeline detects TE copies, groups them into families and defines the consensus sequence for each family containing at least five copies. The TEannot pipeline then annotates TEs using the library of consensus sequences.

The TEdenovo pipeline was used to search for repeats in contigs longer than 29,034 bp (50% of the genome) from the first diploid version (V1) of the *Q*. *robur* reference genome sequence^50^. The first step used Blaster with the following parameters: [identity >90%, HSP (high-scoring segment pairs) length >100 bp and <20 kb, e-value ≤ 1e-300]. The HSPs detected were clustered using Piler^67^, Grouper^66^ and Recon^68^. Multiple alignments (with MAP^69^) of the 20 longest members of each cluster (*n* clusters) containing at least 5 members were used to derive a consensus. Consensus sequences were then classified on the basis of their structure and similarities relative to Repbase Update (v.17.11)^70^ and PFAM domain library v.26.0^71^, before the removal of redundancy (with Blaster + Matcher as in the TEdenovo pipeline). Consensus sequences with no known structure or similarity were classified as ‘unknown’.

The library of 4,552 classified consensus sequences provided by the TEdenovo pipeline was used to annotate TE copies throughout the genome with the TEannot pipeline. Three methods were used for annotation (Blaster, Censor and RepeatMasker). The resulting HSPs were filtered and combined. Three methods (TRF, Mreps and RepeatMasker) were also used to annotate simple sequence repeats (SSRs). TE annotation covered only by SSRs were then removed. Finally a “long join procedure”^72^ was used to address the problem of nested TEs. This procedure finds and connects fragments of TEs interrupted by other more recently inserted TEs to build a TE copy. The nesting patterns of such insertions must respect the following three constraints: fragments must be collinear (both in the genome and with the same reference TE consensus sequence); of the same age; and separated by a more recent TE insertion. The percentage identity to the reference consensus sequence was used to estimate the age of the copy. Using the results of this first TEannot pipeline, we filtered out 2,047 consensus sequences that did not have a full-length copy in the genome. A copy may be built from one or more fragments joined by the TEannot long join procedure. We then performed manual curation to improve the TE annotation. We removed TE copies with consensus sequences identified as part of the host gene. These consensus sequences were built from a family of repeats containing at least five members and were classified as unknown by the TEdenovo pipeline. They were predicted to be host genes from multigene families. We also filtered out consensus sequences identified as chimeric. We obtained a final library of 1,750 consensus sequences, which together captured 52% of the oak genome, a value in the upper range of the values previously reported for plants.

### Gene prediction and functional annotation of protein-encoding genes

We used EuGene v.4.0^73^ to predict gene structure. EuGene predicts gene models from a combination of several lines of in silico evidence (ab initio and similarity). The EuGene pipeline was trained on a set of 342 genomic and full-coding complementary DNA pairs for which coding sequences were confirmed by protein evidence. One-third of the dataset was used for training the following ab initio gene structure prediction software: Eugene_IMM^74^, which is based on probabilistic models for discriminating between coding and non-coding sequences; SpliceMachine^75^, which was used to predict coding sequence (CDS) start and intron splicing sites; and FGENESH, an ab initio gene finder (http://linux1.softberry.com/berry.phtml), which was used with *Populus trichocarpa* parameters. Another one-third of the dataset was used to optimize the EuGene parameters. The final one-third of the training dataset was used to calculate the accuracy of EuGene predictions. Sensitivity values of 85.8% and 75.2%, and specificity values of 87.7% and 74.6%, for exons and genes, respectively, were estimated.

We refined alignments with nucleotide similarity-based methods (Blat and Sim4) using transcript contigs from *Q. robur* and *Quercus petraea*^76^. We ensured that alignment quality was high by respecting the following criteria: 100% coverage and 98% identity for alignments with contigs shorter than 300 bp; <98% coverage and 98% identity for alignments with contig lengths between 300 and 500 bp; <95% coverage and an identity of 98% for alignments with contigs longer than 500 bp; and <95% identity for all other cases. We also used BLAST-x 2.2.29+ to match protein sequences with sequences in protein databases, such as SwissProt, and databases built for species phylogenetically related to oak, such as *P. persica* v.1.39, *Vitis vinifera* v.1.45, *P. trichocarpa* v.2.10, *Eucalyptus grandis* v.2.01 and *Arabidopsis thaliana* v.1.67. We filtered out predicted genes overlapping TEs identified with the REPET package (see previous section), but retained TEs in introns and untranslated regions. The results of the various analyses were combined in EuGene to predict the final gene models. Predicted genes of <100 nucleotides in length were automatically filtered out by EuGene.

We initially predicted 77,043 protein-coding genes from the diploid version (V2) of the *Q. robur* genome sequence. In total, 2,067 genes from different gene families were manually curated by experts (Supplementary Note 3.5). From the 77,043 predicted genes, 43,240 were entirely recovered in the haplome, including 1,176 of the manually curated genes. Genes were tagged as ‘unreliable’ if their coding sequences were <500-bp long (corresponding to 166 amino acids), transcript coverage was <90% or the genes were not curated manually. Based on these criteria, 13,575 genes were tagged as unreliable, and the remaining genes were tagged as ‘regular’ (28,484 genes) or ‘manual’ (1,176 genes).

We then performed a manual analysis of the 43,240 candidate gene models, guided first by an OrthoMCL run of the 16 genome sequences used in the evolutionary analysis (see the section “Oak karyotype evolution and genome organization”), in which we filtered out genes from OrthoMCL clusters associated with the following criteria: domains identified as plant mobile element domains (PMD domain) or TE domains (for example, transposases or GAG, a structural protein for virus-like particles within which reverse transcription takes place); and similarity to TE proteins, based on BLAST analyses against KEGG library results. We also checked that the OrthoMCL clusters contained >90% *Q. robur* genes (that is, with only a minor contribution from other species) as follows: we filtered out ‘potential pseudogenes’ or small gene fragments predicted in regions of dubious assembly due to a high repeat content (that is, presence of TEs or repeated motifs in genes, such as NBS-LRR); we also filtered out unreliable and regular singletons (single genes not clustered with OrthoMCL) with a CDS <500 bp. Some small genes were classified as regular, as they were sufficiently covered by mRNA contigs, but they could be mapped to multiple sites within the genome and could not therefore be considered specific for the gene tagged.

Automated functional annotation was performed on the 25,808 predicted proteins (listed in Supplementary Data Set 1), using an in-house pipeline (FunAnnotPipe), mostly largely on the InterProScan v.5.13–52.0^77^ webservice for domain and motif searches. This included all the manually curated genes, 78% of the regular set and 17% of the unreliable set. Subcellular targeting signals and transmembrane domains were predicted with SignalP, TargetP and TMHMM^78^ and InterProScan. We also carried out similarity searches with BLAST-x V2.2.29+ against PDB, Swissprot and KEGG^79^, and rpsBLAST (14 June 2009) searches for conserved domains against the CDD database^80^ and KOG^81^. We also used the BLASTKoala webservice (http://www.kegg.jp/blastkoala/, January 2016) to associate KEGG orthology groups, and E2P2 to identify the associated enzyme codes when relevant (https://dpb.carnegiescience.edu/labs/rhee-lab/software, v.3.0).

We assigned ‘definitions’ to the predicted proteins as proposed by Phytozome^82^ and D. M. Goodstein (personal communication). We used the annotation from the most accurate analysis as input: EC number (E2P2), KEGG orthology group (KO; KEGGKOALA), PANTHER (InterProScan), KOG (conserved domain database for eukaryotic organisms) and PFAM (InterProScan). We then calculated the multiplicity (M) of annotations across the entire genome, both as single (for example, KOG0157, PF0064 and PF0005) and same-type compound keys (for example, PF0064//PF0005). Mixed compound keys were not considered (for example, KOG0157//PF0064). Weighting (W) factors were applied to protein definitions to give priority to the most informative annotations as follows: EC = 1, KO = 1.1, PANTHER = 2, KOG = 3, PFAM = 4. The final protein definition corresponds to the least frequent description (minimum M × W value) from this analysis. The key advantage of this approach is that it makes it possible to assign a protein definition without over-representing a single type of annotation found at multiple locations. As a result, a protein definition was assigned to 87% of the predicted oak proteins (Supplementary Data Set 1).

### Estimation of heterozygosity of the reference genotype 3P

All the short Illumina paired-end reads used to produce the 3P oak reference genome were mapped against the haplome assembly with bowtie2^83^, using standard parameters for the “fast end-to-end” mode. Duplicated mapped reads were removed with Picard (http://broadinstitute.github.io/picard/). SAMtools/bcftools^84^ were used to call variants. We then used a combination of custom-made scripts (available at http://www.oakgenome.fr) to calculate coverage and estimated allele frequency from the “DP4” tag of the .vcf file. We discarded all SNPs with a minor allele frequency value <0.25 and all insertions and deletions; the proportion of heterozygous sites on the chromosomes was then calculated with a sliding window approach. For each window, this proportion was weighted by the *N*% and the fraction covered, defined here as the proportion of bases within a window satisfying the same sequence depth criteria as used for SNP calling.

### Pool-seq-based estimator of oak genetic diversity

Branches from 38 pedunculate oak trees were sampled in spring 2011 from oak stands within the maritime pine forest (Supplementary Table 17, Supplementary Fig. 53) of the Landes (Southwest France). Branches were harvested with a telescopic pole pruner and placed in darkness for 3 days to trigger the release of starch from chloroplasts. Etiolated leaves were then harvested and their DNA was extracted using a DNeasy Plant Mini Kit according to the manufacturer’s instructions (Qiagen). The amount of DNA was assessed using a NanoDrop ND-1000 spectrophotometer (NanoDrop Technologies Inc, Rockland, DE, USA) and DNA quality was assessed visually by electrophoresis in a 1.2% agarose gel. The 38 genotypes were genotyped with a 12-plex of expressed sequence tag SSRs and an 8-plex of genomic SSRs^85^. We estimated genetic relatedness between genotypes with COANCESTRY^86^, as described previously^87^, and the degree of introgression of sequences from sessile oak (*Q. petreae*) was assessed using STRUCTURE^88^, as described previously^85^. Following this analysis, we excluded three samples identified as possibly related and eight samples displaying a large degree of introgression from sessile oak. We then randomly selected 20 of the remaining 27 trees (Supplementary Table 18) for whole-genome sequencing by pool-sequencing (pool-seq) techniques^89^.

DNA from these 20 oaks was re-extracted from individual samples using an Invisorb Spin Plant Mini Kit (Stratec Molecular). We visually checked the DNA quality by gel electrophoresis (1.5% agarose) and estimated the concentration and purity using a NanoDrop 1000 spectrophotometer (NanoDrop Technologies). We then pooled DNA from individual samples to obtain an equimolar solution with a final concentration of 570 ng µl^–1^. We used this pool of DNA to prepare a paired-end genomic library with a Paired-End DNA Sample Preparation Kit (Illumina). This library was sequenced on 10 lanes of a HiSeq2000 sequencer (Illumina) (2× 100-bp paired-end reads), generating 1,732,899,595 paired-end reads (331 Gb, that is, ~400× haploid genome coverage).

Raw reads were trimmed to remove low-quality bases, as described in the “Sequence processing” section. All reads were then mapped against the oak haplome assembly with bowtie2^83^, using standard parameters for the “sensitive end-to-end” mode. Potential PCR duplicates were removed using Picard (http://broadinstitute.github.io/picard/). Samtools^84^ and PoPoolation2^90^ were then used to call SNPs with counts of at least 10 for the alternate allele and a depth between 50 and 1,000× at the position concerned. All SNPs with a minor allele frequency value <0.05 were discarded. After subsampling the pileup at all retained positions to a uniform coverage of 30× (“subsample-pileup.pl”, PoPoolation suite^91^), we used the “variance-sliding.pl” script (PoPoolation^91^) to calculate π along chromosomes by a sliding window approach (1-Mb sliding windows, 250-kb steps, Supplementary Figs. 4 and 15).

### Estimate of genetic diversity and π0/π4 ratio

We estimated genetic diversity as pairwise nucleotide diversity (π) at zerofold and fourfold sites for each protein-coding gene, as described previously^9^. We then defined the π_0_/π_4_ ratio as the ratio of mean π_0_ to mean π_4_ over all genes. We also computed these metrics on manually curated genes, which showed that the gene model quality did not compromise our findings. We compared estimates between genes from expanded, contracted and unchanged gene families (orthogroups) in oak. We accounted for the different gene family sizes by randomly sampling 1,000 genes from each of these three categories and repeating the operation 100 times.

### Detection of somatic mutations

Our objective was to show that somatic mutations (in terms of SNPs) exist in a long-lived plant and transmitted to the next generation. Because we did not intend to provide a comprehensive estimate of the number of somatic mutations in the studied 100-year-old tree, it is meaningless to compare our result to an expected number of somatic mutations because of the following unknown factors: the substitution rate per site and per generation; the number and pattern of mitotic divisions from zygote and axillary buds; and cell death and bud abortion rates.

We investigated somatic mutations by resampling the 3P genotype used to sequence and assemble the reference genome, as described below.

Vegetative buds were collected from the extremities of three second-order branches of the 2011 increment in February 2012: two lateral branches (L1 and L2) and the tree apex (L3). We used dendrochronology (tree-ring dating) to date the time of initiation of the L1 and L2 branches (Supplementary Fig. 5). To this end, we collected 5-mm diameter wood cores from the insertion point of the selected branches with an increment borer. We also dated the age of the tree by taking a core just above ground level and counting the number of rings under a microscope. We estimated that the L1 and L2 branches had been initiated 15 and 47 years earlier, respectively, and that the terminal branch was at least 85 years old.

DNA was extracted from three sets of vegetative buds sampled at location L1, L2 and L3 using the Invisorb Spin Plant Mini Kit (Stratec Molecular). For each sample, six independent DNA extractions were carried out on a pool of buds. DNA quality was checked by electrophoresis in a 1.5% agarose gel. DNA concentration and purity were assessed with a NanoDrop 1000 spectrophotometer (NanoDrop Technologies). Individual DNA samples from the same branch were the pooled in an equimolar solution to obtain a final concentration of 769–1,388 ng µl^–1^. We prepared tightly sized paired-end libraries (600 bp in size) as described in the “Sequencing” section and sequenced each of these libraries on one to four lanes of a HiSeq2000 or HiSeq2500 sequencer (Illumina) (Supplementary Table 19, 100-bp or 250-bp paired-end reads). We obtained 284-fold (L1), 250.5-fold (L2) and 264.9-fold (L3) haploid genome coverage for these samples. For each of the three branches (L1, L2 and L3), reads were mapped against the reference genome sequence with BWA-MEM^92^ using the default parameters, except for minimum seed length (*k* = 79). After sorting, PCR duplicates were removed with Picard (http://broadinstitute.github.io/picard/). We searched for somatic mutations using MuTect (a program developed for the detection of somatic point mutations in heterogeneous cancer samples^11^) to compare the three libraries (six pairwise combinations; Supplementary Table 20). This frequency-dependent detection approach was considered to be particularly well suited to identify somatic mutations in plants.

Because considering sequencing error (that is, false positives) is essential for detecting mutations and is vital for drawing valid conclusions, particularly with respect to the detection of somatic mutations within a single individual, we addressed this concern and took all possible actions to minimize it. Thus, the accuracy of somatic point mutations was ensured by considering only those sites with the following characteristics: a minimum depth of 50× in both the reference and potentially mutated libraries; no mutant (that is, alternative) allele in the reference library; and a minimum frequency of 20% for the mutant allele in the potentially mutated library (that is, each somatic mutation was supported by 10 alternative alleles or more). We then filtered out candidate somatic mutations by using a cross-validation procedure. Across all pairwise comparisons, we only kept somatic mutations with a temporal pattern coherent with the chronology of branch development (see Supplementary Table 20 for details). These multiple comparisons made it possible both to validate the detected mutations and to reconstruct their mutational history along the trunk or the two branches. Finally, we discarded 15 additional candidate mutations among the set of 61 reliable somatic mutations. Indeed, for this set of 15 somatic mutations, we recovered the same alternate allele in the pool of 20 pedunculate individuals (see the section “Pool-seq-based estimator of oak genetic diversity”) at a frequency >0.005. Note that f(alt) <0.005 remains a stringent criterion considering Illumina sequencing error calls (0.024). As a consequence, we cannot rule out that some true positives were excluded at this step. However, our objective was to be as conservative as possible in order to study the transmission of these somatic mutations to the next generation (Supplementary Table 5).

We studied the transmission of somatically acquired mutations to the offspring by extracting DNA using a DNAeasy 96-Plant Kit (Qiagen) from 116 acorns sampled from the extremities of the L1 and L2 branches (Fig. 2b). DNA was extracted after the dissection of embryonic tissues (radicle and plumule) from the acorn. We used 15 ng DNA to genotype the offspring using a MassArray iPLEX Assay (Agena Bioscience) according to the manufacturer’s instructions. Primers were designed, and 33 SNPs were multiplexed in the Assay Design Suite (Agena Bioscience). Allele calling was processed in Typer Viewer v.4.0.26.75 (Agena Bioscience). This 39-plex assay contained 12 control SNPs and 21 candidate somatic mutations (Supplementary Table 5). Control SNPs were used to provide an estimate of the selfing rate likely to impair interpretation of the segregation of somatic mutations in the offspring. The control SNPs were loci homozygous in the reference genotype 3P and found at a very low frequency in the pool of 20 pedunculate oaks; that is, with minimum allele frequencies ranging from 0.02 to 0.05. Embryos resulting from the self-pollination of 3P were expected to be homozygous for the reference allele, and most outcrossed embryos were expected to be heterozygous. We observed a mean heterozygosity of 0.54 over the 12 control loci. In the absence of selfing and based on allele frequencies estimated in the pool of 20 individuals, mean heterozygosity would have been close to 0.96, thus suggesting a relatively high rate of selfing (44%). Unamplified loci (2/21 SNPs; Supplementary Table 6) were excluded from the analysis. The overall rate of missing data was high (39% for missing somatic mutations and 54% for control SNPs), so all polar plots from Typer Viewer software of the MassArray iPLEX assay were inspected visually to check that genotyping calls were accurate.

### Oak karyotype evolution and genome organization

We used two previously defined parameters^93^ to increase the stringency and significance of BLAST sequence alignment by either parsing BLAST results and rebuilding HSPs or using pairwise sequence alignments to identify accurate paralogous relationships within oak (25,808 gene models; Supplementary Data Set 1). Orthologous relationships between oak and grape (26,346 genes on 19 chromosomes^15^), peach (28,086 genes on 8 chromosomes^16^) and cocoa (23,529 genes on 10 chromosomes^17^) were also determined. We estimated the sequence divergence of paralogues and orthologues from the *K*_s_ calculated with the PAML 4 package^94^ for oak–peach, grape–grape, peach–peach, cocoa–cocoa and oak–oak gene pairs. Dot plot representations of synteny and paralogy were obtained with the R package ggplot2 (http://ggplot2.org/; Supplementary Fig. 16).

### Gene family expansion and contraction in oak

A classification of groups of orthologous sequences (orthogroups, also referred to here as gene families or clusters) was developed for 16 eudicot plant species: all the predicted oak proteins (corresponding to 25,808 gene models) and the proteins catalogued from 15 other eudicot species (Supplementary Table 21, Supplementary Note 5.1.2). The other eudicot species were *Arabidopsis lyrata, A. thaliana, Citrus clementina, Carica papaya, E. grandis, Fragaria vesca, Glycine max, Malus domestica, P. persica, P. trichocarpa, Ricinus communis, Solanum tuberosum, Theobroma cacao, V. vinifera* (genomes available from https://phytozome.jgi.doe.gov) and *Citrullus lanatus* (genome available from http://www.icugi.org/cgi-bin/ICuGI/index.cgi). These 15 plant genomes were selected on the basis of the following criteria: availability of genome sequences and gene models from public databases; assembly quality (N50 length of assembled fragments) and the number of predicted genes; classification (order, family and genus), the main goal being to cover the entire range of eudicots. The classification was based on a BLAST-p all-against-all comparison of the complete proteomes (E-value <10^−5^) of these species, followed by clustering with OrthoMCL 2.0.9^95^ using default parameters. GO terms for 15 of the plant proteomes were retrieved from Phytozome. For watermelon, the CDS were downloaded from the following website: http://cucumber.genomics.org.cn/page/cucumber/index.jsp. We used Interproscan^96^ to assign GO terms. GO term enrichment analysis was then carried out on the expanded orthogroups in oak (Supplementary Note 5.4).

We then used CAFE v.3.1^97,98^ with phylogenetic tree information (drawn from http://etetoolkit.org/treeview/) derived from previous studies^18^ (Supplementary Fig. 17) to identify the orthogroups displaying expansion and contraction in oak using a *P* value threshold of 0.01.

### Identification and validation of TDGs in oak

Duplicated genes in oak were identified from the *K*_s_ paralogue distribution (see purple *K*_s_ distribution in Supplementary Fig. 20) and are illustrated in the dot blot shown in Supplementary Fig. 21 (see also Supplementary Note 5.2). We extracted duplicated genes from the complete repertoire of paralogues and generated pairwise alignments of protein sequences with BLAST-p and filters based on alignment identity and length (CIP (cumulative identity percentage)/CALP (cumulative alignment length percentage) = 50%/50%). Then we sorted protein sequences by their coordinates on each of the 12 oak chromosomes. We defined TDGs as duplicates separated by up to three genes and LDGs as duplicates separated by more than three genes. The remaining genes were classified as singleton genes.

We checked that these recent TDGs in oak were true duplicates rather than different alleles or duplication artefacts arising during haplome construction (during the scaffolding or merging steps of our hierarchical assembly pipeline) by applying two verification procedures based on sequence variation and sequence coverage. First, we obtained pairwise nucleotide sequence alignments, using MUSCLE with standard parameters^99^, for all 9,189 putative TDGs. For each alignment, summary statistics were calculated with AMAS^100^. We found that 15 gene pairs involved in local duplications presented no gaps or polymorphisms and could be considered to be putative assembly artefacts. This corresponds to only a minor fraction (0.13%) of the 11,695 pairwise alignments. In contrast, we found that 8,115 pairs of TDGs (69.4%) displayed substantial sequence divergence (gap length >10% and a proportion of variable sites >2%), greater than that between pairs of alleles (Supplementary Fig. 22). Indeed, from the 12,603 allelic pairs obtained by comparing the diploid and haploid versions of the oak genome sequence available for this comparison (indicated as 2:1 relationships in the last column of Supplementary Data Set 1), 1,278 (that is, 10.1%) had a gap length >10% and a proportion of variable sites >2%. Second, a per-base coverage analysis based on reads from the genes classified as TDGs, LDGs and singleton genes indicated that TDGs did not represent half the coverage of the other two categories (illustrated for the longest scaffold in Supplementary Fig. 23), ruling out the alternative hypothesis that TDGs are allelic regions or artefactual duplications due to errors in the assembly process.

### Detection of significant expansion and contraction in woody perennials

Particular outcomes of gene family expansion and contraction may be associated with the lifestyle of a tree, but no study of differential gene gains and losses has been performed at the genomic scale in eudicots (Supplementary Note 5.3). We therefore applied an additional criterion when selecting the 15 plant species for comparative genomic analyses; that is, the growth habit (woody perennial versus herbaceous). The genomes of nine woody perennials and seven herbaceous species were available for the investigation of orthogroup expansion in woody species (trees). These two categories were homogeneous in terms of OrthoMCL orthogroups. For a range of variables, including the number of genes per orthogroup (Supplementary Fig. 24), the mean number of genes per orthogroup, the percentage of orthogroups with no genes, and the number of species-specific orthogroups (Supplementary Table 22), no statistical difference was found between the two categories.

We investigated whether a given orthogroup showed significant expansion or contraction in trees by comparing the total number of genes per orthogroup between the two types of growth habit. Given the relatively small number of species per category, we performed a binomial test with a probability of success of *p*(W) = 9/16. From the initial set of 36,844 orthogroups, we retained orthogroups displaying a statistically significant outcome in terms of gene counts (false discovery rate-adjusted *P* value <0.05^101^). The minimal contribution to each category was set to five for trees and four for herbaceous species to minimize bias due to the number of species analysed. We found that 126 orthogroups were expanded (corresponding to 23,321 genes; that is, 155.1 genes per orthogroup on average) and 23 were contracted in woody perennials relative to herbaceous species. Functional identities and orthogroup sizes are presented for all significantly expanded or contracted orthogroups in Supplementary Data Set 7 (sheets 2 and 4). GO term enrichment analysis was carried out on the 126 expanded and 23 contracted orthogroups (see next section). We also identified a set of remarkable orthogroups (outliers in Fig. 3d), differing between trees and herbaceous species and including at least five genes in five different species.

### GO enrichment analysis

All GO term enrichment analyses were performed using R 3.3.1 software^102^ and the topGO 2.22.0 package^103^. The weight01 algorithm^103^ and Fisher’s exact test were used to detect significant enrichment in GO terms in the various test sets. As stated by the authors of topGO, the *P* value of a GO term is conditioned on the neighbouring terms. The tests are therefore not independent, and the multiple testing theory does not directly apply. *P* values should therefore be interpreted as corrected or not affected by multiple testing.

Fold-enrichment was defined as illustrated below:

- At the gene level, if 52/9,189 (that is, 0.56%) of input genes are involved in “chitinase activity” and the background level is 60/25,808 genes (that is, 0.23%) associated with chitinase activity, the fold-enrichment is approximately 0.56%/0.23% = 2.43 for this molecular function.
- At the orthogroup level, if 6/126 (that is, 4.76%) of input orthogroups are involved in “protein serine/threonine kinase activity” and the background level is 50/36,844 orthogroups (that is, 0.136%) associated with protein serine/threonine kinase activity, the fold-enrichment is approximately 4.76%/0.136% = 35 for this molecular function.

The first example corresponds to the fold-enrichment calculations performed for TDGs, LDGs, singleton genes and orthogroups expanded in oak. The second corresponds to the fold-enrichment calculation for orthogroups expanded in woody perennials.

### Web resources

We set up several tools and a browser based on the international open-source project Generic Model Organism Database (http://www.gmod.org) to provide us with access to both structural information and functional annotation (Supplementary Note 6). WebApollo/JBrowse^104^ was set up (https://urgi.versailles.inra.fr/WebApollo_oak_PM1N/jbrowse/) and populated with the oak reference genome sequence (that is, 12 chromosomes comprising 876 scaffolds and 533 unassigned scaffolds) and 34 BAC sequences. Several tracks were superimposed on these sequences, including predicted genes, predicted TEs, predicted non-coding RNAs, proteins from several species, oak unigenes, RNA-sequencing data and quantitative trait loci. The ‘chunk’ track represents virtual contig sequences separated by *N* stretches of no more than 11 consecutive bases. Intermine (v.1.3.9)^105^ was used to gather and make available all the information (structural and functional) produced for each protein-coding gene (https://urgi.versailles.inra.fr/OakMine_PM1N/begin.do). All the details about data sources are available from the application in the datasource panel. For JBrowse, tracks have been generated from the reference genome using data generated for EuGene prediction.

### Reporting summary

Further information on experimental design is available in the Nature Research Reporting Summary linked to this article.

### Code availability

The source code for the prediction of miRNA is available as a workflow at https://forgemia.inra.fr/genotoul-bioinfo/ngspipelines/tree/master/workflows/srnaseq. Custom-made scripts for the estimation of heterozygosity of the reference genotype 3P are available at the oak genome website (http://www.oakgenome.fr/?page_id=587).

### Data availability

The oak haploid genome assembly and corresponding annotation have been deposited in the European Nucleotide Archive under project accession code PRJEB19898. Other sequence release data are indicated in Supplementary Tables 1, 13, 14 and 19, and Supplementary Data Set 10. Data (including intermediate genome assemblies, .vcf files used to detect somatic mutations and estimate heterozygosity) are available at the oak genome website hosted as a permanent resource by INRA (http://www.oakgenome.fr/).

## Supplementary information

Supplementary Information Supplementary Figures 1–60, Supplementary Tables 1–43 and Supplementary References.

Reporting Summary

Supplementary Dataset 1 List of 25,808 oak gene models with their annotations.

Supplementary Dataset 2 Mapping data used to anchor the scaffolds onto the oak genetic linkage map.

Supplementary Dataset 3 List of orthogroups (orthoMCL analysis) and expanded gene families (CAFE analysis) in pedunculate oak.

Supplementary Dataset 4 List of gene categories.

Supplementary Dataset 5 Classification of NB-LRR-related genes.

Supplementary Dataset 6 Classification of RLK-related genes.

Supplementary Dataset 7 Summary of orthogroups expanded in ‘trees’.

Supplementary Dataset 8 Summary of the gene ontology (GO) term analysis showing significantly enrichment in GO terms for molecular functions (MF), biological processes (BP) and cellular components (CC).

Supplementary Dataset 9 Footprint of selection in RLK-related genes.

Supplementary Dataset 10 List of pedunculate oak BAC clones used in this study.
