# Supplementary material for: Oak genome reveals facets of long lifespan
Source: Nat Plants. 2018 Jun 18;4(7):440–52. doi: 10.1038/s41477-018-0172-3 (PMC6086335; doi:10.1038/s41477-018-0172-3)

nature plants

SUPPLEMENTARY INFORMATION
https://doi.org/10.1038/s41477-018-0172-3

In the format provided by the authors and unedited.

Oak genome reveals facets of long lifespan

Christophe Plomion[1,24*], Jean-Marc Aury[2,24], Joëlle Amselem[3,24], Thibault Leroy[1], Florent Murat[4], Sébastien Duplessis[5], Sébastien Faye[2], Nicolas Francillonne[3], Karine Labadie[2], Grégoire Le Provost[1], Isabelle Lesur[1,6], Jérôme Bartholomé[1], Patricia Faivre-Rampant[7], Annegret Kohler[5], Jean-Charles Leplé[8], Nathalie Chantret[9], Jun Chen[10], Anne Diévart[11,12], Tina Alaeitabar[3], Valérie Barbe[2], Caroline Belser[2], Hélène Bergès[13], Catherine Bodénès[1], Marie-Béatrice Bogeat-Triboulot[14], Marie-Lara Bouffaud[15], Benjamin Brachi[1], Emilie Chancerel[1], David Cohen[14], Arnaud Couloux[2], Corinne Da Silva[2], Carole Dossat[2], François Ehrenmann[1], Christine Gaspin[16], Jacqueline Grima-Pettenati[17], Erwan Guichoux[1], Arnaud Hecker[5], Sylvie Herrmann[18], Philippe Hugueney[19], Irène Hummel[14], Christophe Klopp[16], Céline Lalanne[1], Martin Lascoux[10], Eric Lasserre[20], Arnaud Lemainque[2], Marie-Laure Desprez-Loustau[1], Isabelle Luyten[3], Mohammed-Amin Madoui[2], Sophie Mangenot[2], Clémence Marchal[5], Florian Maumus[3], Jonathan Mercier[2], Célia Michotey[3], Olivier Panaud[20], Nathalie Picault[20], Nicolas Rouhier[5], Olivier Rué[16], Camille Rustenholz[19], Franck Salin[1], Marçal Soler[17,21], Mika Tarkka[15], Amandine Velt[19], Amy E. Zanne[22], Francis Martin[5], Patrick Wincker[23], Hadi Quesneville[3], Antoine Kremer[1] and Jérôme Salse[4]

[1]BIOGECO, INRA, Université de Bordeaux, Cestas, France. [2]Commissariat à l'Energie Atomique (CEA), Genoscope, Institut de Biologie François-Jacob, Evry, France. [3]URGI, INRA, Université Paris-Saclay, Versailles, France. [4]GDEC, INRA-UCA, Clermont-Ferrand, France. [5]IAM, INRA, Université de Lorraine, Champenoux, France. [6]HelixVenture, Mérignac, France. [7]INRA, US 1279 EPGV, Université Paris-Saclay, Evry, France. [8]BIOFORA, INRA, Orléans, France. [9]AGAP, Université de Montpellier, CIRAD, INRA, Montpellier SupAgro, Montpellier, France. [10]Department of Ecology and Genetics, Evolutionary Biology Centre, Science for Life Laboratory, Uppsala University, Uppsala, Sweden. [11]CIRAD, UMR AGAP, Montpellier, France. [12]Université de Montpellier, CIRAD, INRA, Montpellier SupAgro, Montpellier, France. [13]CNRGV, INRA, Castanet, France. [14]UMR Silva, INRA, Université de Lorraine, AgroPariTech, Nancy, France. [15]Department of Soil Ecology, UFZ–Helmholtz Centre for Environmental Research, Halle/Saale, Germany. [16]Plateforme bioinformatique Toulouse Midi-Pyrénées, INRA, Auzeville Castanet-Tolosan, France. [17]Université de Toulouse, CNRS, UMR 5546, LRSV, Castanet-Tolosan, France. [18]German Centre for Integrative Research (iDiv), Halle–Jena–Leipzig, Leipzig, Germany. [19]SVQV, Université de Strasbourg, INRA, Colmar, France. [20]Université de Perpignan, UMR 5096, Perpignan, France. [21]Laboratori del Suro, University of Girona, Girona, Spain. [22]Department of Biological Sciences, George Washington University, Washington, DC, USA. [23]Génomique Métabolique, Genoscope, Institut de Biologie François-Jacob, Commissariat à l'Energie Atomique (CEA), CNRS, Université d'Evry, Université Paris-Saclay, Evry, France. [24]These authors contributed equally: Christophe Plomion, Jean-Marc Aury, Joëlle Amselem.
*e-mail: christophe.plomion@inra.fr

© 2018 Macmillan Publishers Limited, part of Springer Nature. All rights reserved.

SUPPLEMENTARY INFORMATION

Oak genome reveals facets of long lifespan

Christophe Plomion[1*]†, Jean-Marc Aury[2]†, Joëlle Amselem[3]†, Thibault Leroy[1]‡, Florent Murat[4]‡, Sébastien Duplessis[5], Sébastien Faye[2], Nicolas Francillonne[3], Karine Labadie[2], Grégoire Le Provost[1], Isabelle Lesur[1,6], Jérôme Bartholomé[1], Patricia Faivre-Rampant[7], Annegret Kohler[5], Jean-Charles Leplé[8], Nathalie Chantret[9], Jun Chen[10], Anne Diévart[11,12], Tina Alaeitabar[3], Valérie Barbe[2], Caroline Belser[2], Hélène Bergès[13], Catherine Bodénès[1], Marie-Béatrice Bogeat-Triboulot[14], Marie-Lara Bouffaud[15], Benjamin Brachi[1], Emilie Chancerel[1], David Cohen[14], Arnaud Couloux[2], Corinne Da Silva[2], Carole Dossat[2], François Ehrenmann[1], Christine Gaspin[16], Jacqueline Grima-Pettenati[17], Erwan Guichoux[1], Arnaud Hecker[5], Sylvie Herrmann[18], Philippe Hugueney[19], Irène Hummel[14], Christophe Klopp[16], Céline Lalanne[1], Martin Lascoux[10], Eric Lasserre[20], Arnaud Lemainque[2], Marie-Laure Desprez-Loustau[1], Isabelle Luyten[3], Mohammed-Amin Madoui[2], Sophie Mangenot[2], Clémence Marchal[5], Florian Maumus[3], Jonathan Mercier[2], Célia Michotey[3], Olivier Panaud[20], Nathalie Picault[20], Nicolas Rouhier[5], Olivier Rué[16], Camille Rustenholz[19], Franck Salin[1], Marçal Soler[17,21], Mika Tarkka[15], Amandine Velt[19], Amy E. Zanne[22], Francis Martin[5], Patrick Wincker[23], Hadi Quesneville[3], Antoine Kremer[1], Jérôme Salse[4]

1. Information about the genus *Quercus* **3**
2. Reference genome sequencing, assembly and anchoring **5**
2.1. BAC sequence analysis 5
2.1.1. Construction and screening of libraries, sequencing and annotation 5
2.1.2. Results of BAC sequence analysis 8
2.1.3. Comparison of genomic structure between haplotypes 10
2.2. Comparison of the V1 and V2 assemblies and assembly validation 11
2.3. Pseudomolecule construction 12
3. Genome annotation **14**
3.1. Detection and annotation of transposable elements 14
3.2. Identification and preliminary characterization of endogenous Caulimoviridae 15
3.3. Gene prediction and functional annotation of protein-encoding genes 16
3.4. TEs and genome dynamics 16
3.4.1. Estimation of the age of TE families from consensus sequences 16
3.4.2. Retrotransposition dynamics 17
3.4.3. Distribution of TEs and genes in the oak genome 18
3.4.4. Role of TEs in gene expansion and tandem duplication 19
3.4.5. Horizontal transfer of TEs 19
3.5. Gene prediction, functional annotation of protein-encoding genes and manual curation 20
3.5.1. Aquaporin 21
3.5.2. MYB 22

3.5.3. SWEET 24
3.5.4. Thioredoxin, glutaredoxin and glutathione transferase 26
3.5.5. MLO 28
3.5.6. NB-LRR 30
3.5.7. RLK 32
3.5.8. Biosynthesis of hydrolysable tannins 35
3.5.9. Laccases 37
3.6. Non-coding RNA prediction and annotation 38
4. Mutational landscape 42
4.1. Estimate of genetic diversity and π0/π4 ratio 42
4.2. Detection of somatic mutations 43
5. Comparative and evolutionary genomics 44
5.1. Macroevolutionary analysis 44
5.1.1. Oak karyotype evolution and genome organization 44
5.1.2. Gene family expansion/contraction in oak 44
5.2. Identification of tandemly duplicated genes in oak 45
5.3. Challenges in the identification of genes related to tree habit 45
5.4. Gene ontology (GO) enrichment analysis 47
5.4.1. GO term enrichment in three categories of genes 47
5.4.2. GO term enrichment in orthogroups expanded in pedunculate oak 50
5.4.3. GO enrichment within the gene families expanded in woody perennial trees relative to herbaceous species 51
6. Web resources 53
7. Data availability 53
8. References 53
9. Supplementary Data Sets 71
10. Supplementary Tables 74
11. Supplementary Figures 139

1. Information about the genus *Quercus*

Oaks are the dominant tree species in many temperate ecosystems and landscapes. Their species diversity and geographic distribution underlie this predominance. There are about 350 to 450 oak species worldwide[1], although species delineation remains a matter of debate due to considerable phenotypic variation within species and frequent hybridization. However, oak species diversity is much greater in North and Central America (about 200 species) than in Asia (about 150 species), and Europe (about 30 species)[2]. On all three continents, a few species have a continent-wide distribution: *Q. petraea* and *Q. robur* in Europe, *Q. macrocarpa* in North America[3], and *Q. acutissima* and *Q. mongolica* in Asia[4]. The IUCN lists 13 oak species as critically endangered and 16 as endangered, mostly due to land use conflicts or overexploitation[5]. About 240 species are maintained in *ex situ* collections, in arboreta and botanical gardens. Unlike other important temperate forest species, such as conifers, oaks are not intensively cultivated in artificial forest plantations. Forest renewal is driven mostly by natural regeneration, and oak plantations often target specialist output markets, for veneer, cork or truffles. In many countries, oaks are also used outside their native distribution range, in urban forestry, for example, in which they are planted in parks and streets. Horticultural cultivars have occasionally been selected for these highly specialized purposes.

Oaks provide major ecosystem services, ranging from the provision of raw construction materials and the regulation of natural resources, to the conservation of biodiversity and the provision of recreational and cultural services[6]. Oaks have been making an invaluable contribution to human society since humans first reached the Northern Hemisphere, when acorns were a regular part of the human diet[7]. Timber can be obtained from most temperate oak species, but oaks have always fulfilled multiple functions in human societies, by providing a combination of habitat, economic and cultural services[8]. With recent increases in public awareness of the environment, forest ecosystem services have been extended to include

the enhancement of carbon sequestration and biomass production for bioenergy purposes. Oaks produce numerous raw materials, including wood, cork, fiber, biomass, and biomolecules; these raw materials are used to produce diverse manufactured products for the construction, food, pharmaceutical, and cosmetics industries[9]. This tremendous utility of oaks is illustrated by the diverse uses of wood, bark (cork), leaves, and even acorns. Oak wood is frequently used for fuel, timber frames, interior paneling, veneers, and barrels for wines and spirits, whereas cork is use to make stoppers for bottles and in coverings for floors, walls and ceilings. Tannins have been extracted from oaks for centuries, for use in the leather industry, and oak secondary metabolites are now used in the cosmetics industry. Finally, there is a renewed interest in the possible use of acorns in the human diet, for both nutritional and ecological reasons, to meet the challenges raised by human population growth in a context of substitution for food products with a high carbon cost[10]. Oaks also generate other important biological products by providing a habitat for associated species. Edible mushrooms, such as boletes and truffles in particular, are the fruiting bodies of mycorrhizal fungi that grow in association with oak roots and are harvested in many countries for their gastronomic and nutritional qualities. Iron gall ink, which was used for centuries for the writing of official documents and parchments, to ensure that they did not fade, is made from iron salts and gallic acid from oak galls. Oaks also provide ecological services as single trees and as forests, by offering shelter to a very large range of fungi, insects, birds, and other wildlife, the list of species benefiting from these services being continually updated and lengthened. In many parts of the world, oak forests have been assigned functions in habitat conservation, contributing to the preservation of natural resources, such as water or soils.

Oaks also occupy a special position in science, for case studies of tree biology and evolution, and as a major research tool in the fields of archeology, history and climatology[11]. Oaks are very long-lived, and oak-ring width series in Central Europe have been reconstructed as far

back as 8,480 BC. These ring series are used as a standard dating tool with a yearly resolution in archeology, and, in some cases, as a tool for dendro-provenancing[12]. This resource is continually updated with data from archeological remains, opening up additional possibilities for applications in climate reconstruction[13]. Oak microfossil remains are frequent and widely distributed, due to the past and present widespread distribution of these trees, and may therefore serve as biological indicators of previous plant distributions. The use of oak-ring series also poses new research questions concerning the stasis or microevolution of oaks across the Holocene and Anthropocene. Climate reconstruction, inferred from ring series, assumes the conservation of a climate-growth relationship, which may be challenged by the plastic or evolutionary response of oaks to environmental changes. These questions have triggered genetic and genomic investigations of the ability of long-lived species to adapt to rapid environmental changes.

2. Reference genome sequencing, assembly and anchoring

2.1. BAC sequence analysis

2.1.1. Construction and screening of libraries, sequencing and annotation

BAC library construction

Two BAC libraries were constructed from high-molecular weight genomic DNA from the *Q. robur* “3P” accession, partially digested with *Eco*RI for one library, and *Hin*dIII for the other. The *Eco*RI library, which was obtained from Clemson University (CUGI), was generated by a standard procedure[14], as previously described[15]. This library comprises 92,160 BAC clones with a mean insert size of 135 kb, corresponding to approximately 12x coverage of the haploid pedunculate oak genome. A second BAC library was constructed, with *Hin*dIII digestion, to increase genome coverage and reduce the bias associated with the uneven

distribution of restriction sites. The *Hin*dIII library was constructed at the French Plant Genomic Resource Center (CNRGV, http://cnrgv.toulouse.inra.fr/). Nuclei were isolated from young leaves[14]. High-molecular weight DNA was partially digested with *Hin*dIII and subjected to size selection and the ligation of appropriately sized fragments into the pIndigoBAC-5 *Hin*dIII-Cloning Ready vector (Epicentre Biotechnologies, Madison, Wisconsin, USA). This library contained 98,304 clones with a mean insert size of 120 kb, providing 14x coverage of the haploid genome. These two BAC libraries are available from the CNRGV (http://cnrgv.toulouse.inra.fr/Library/Oak) under accession codes Qro-B-EnglishOak 3P (*Eco*RI) and Qro-B-3Ph (*Hin*dIII).

Screening of BAC libraries

The two BAC libraries were arranged in plate and row pools for PCR screening, as described by Chalhoub et al.[16]. Three sets of BAC clones were screened (summarized in **Supplementary Data Set 10 sheet #1**): i) allelic BACs, to validate the assembly procedure for the diploid genome sequence, i.e. the presence of distinct scaffolds (haplotypes) corresponding to the allelic BACs, ii) BACs carrying expressional or positional candidate genes coinciding with QTLs for water-use efficiency, bud burst or epinasty, for further studies aiming to characterize QTLs associated with adaptive traits, and iii) BACs selected at random or on the basis of BAC end sequences. The primer pairs for library screening were designed from expressed sequence tags, gene sequences, genetic markers or BAC end sequences. PCR was performed as described by Faivre-Rampant et al.[15]. The success of PCR amplification was checked by subjecting the PCR to electrophoresis in 2% agarose gels. Positive plate pools were used to identify potential clones, which were subsequently validated by a second PCR analysis of individual clones. For the identification of allelic BAC clones, the pools were screened with primer pairs specific to single-copy microsatellite loci shown during genetic mapping experiments to be heterozygous in the reference “3P” genotype[17]. Each allelic BAC

clone was selected by visualizing length polymorphisms between PCR products or by direct sequencing of the products of PCR amplification for biallelic markers.

Sequencing and annotation of BAC inserts

In total, 34 BAC inserts were fully sequenced and annotated. Selected clones were cultured individually on LB medium and DNA was isolated by the standard alkaline method[16]. DNA inserts were sequenced in pools at 40x coverage, with the 454 mate-pair (5 kb) procedure. The sequence reads were assembled with Newbler (version MapAsmResearch-04/19/2010-patch-08/17/2010). Additional sequencing was carried out with the Illumina MiSeq platform (paired-end overlapping reads of 2 × 250 bp) at a coverage depth of 400x, and GapCloser (-V1.12-6) software was used to reduce the gaps between contigs.

Repeated elements were identified and classified in a two-step approach: i) Censor software and Repbase V21.08[18] were initially used (see Plomion et al.[19]), but detection was limited to BAC insert regions displaying identity to sequences in Repbase; ii) searches for the remaining repeated elements were performed with the library of consensus repetitive elements presented in section 3.1. Structural and functional gene annotations were added to the BAC sequence, as described in the approach presented in section 3.3, using: i) Eugene to integrate *ab initio* and similarity-based gene finding programs[20], and ii) FunAnnotPipe, an in-house bioinformatic pipeline based principally on InterproScan[21]. The data were then manually curated with BLAST tools from the NCBI website and NetGen2[22] for the confirmation of exon/intron boundaries. Transcript evidence (ESTs and oak unigenes[23] were used to establish gene model structures. We also used FGENESH[24] and Augustus[25] software to confirm or update Eugene predictions, with *Vitis vinifera* or *Theobroma cacao* as the model. Some short-gene models (encoding < 50 amino acids) were removed. Manually curated genes were then compared with gene models predicted from the genome sequence.

Haplotype diversity analysis

We compared sequences between allelic BACs previously reported by Plomion et al. [19], using Dotter Yass software (http://bioinfo.lifl.fr [26]). Local alignments were generated with NUCmer from the MUMmer package[27] and visualized with the Easyfig pipeline http://easyfig.sourceforge.net[28].

Data availability

BAC sequences were deposited in the European Nucleotide Archive under accession numbers LT99005-LT99038 (see **Supplementary Data Set 10 sheet #1** for the accessions). The 34 BAC sequences, with their annotations, are available from the oak genome browser (https://urgi.versailles.inra.fr/WebApollo_oak_PM1N/PseudoMolecule.html). The track “Gene_BAC_manual” provides manually curated gene models.

2.1.2. Results of BAC sequence analysis

Eight of the 34 sequenced BAC inserts were assembled into continuous sequences without gaps. The others formed a set of oriented contigs separated by stretches of 100 nucleotides. Each BAC corresponded to one or two scaffolds of the diploid version of the oak genome sequence (**Supplementary Data Set 10 sheet #1**). Gaps were flanked by low-complexity repeat sequences. One of the 34 BACs sequenced (#10P13) corresponded to chloroplast DNA, nine corresponded to randomly picked clones from the libraries and 24 corresponded to selected clones identified by PCR screening with single-copy genetic markers or candidate genes. BLAST-n analysis with primer, genetic marker or candidate gene sequences confirmed the presence of these sequences in the targeted BAC. The nuclear BAC assembly covered 4,282,332 bp. The mean G+C content of nuclear BAC sequences was 35.9%, and all BAC sequences had G+C contents close to this mean. A similar G+C content was reported in a previous study for 20,056 BES[16].

The 33 BAC sequences corresponding to nuclear regions were screened for simple sequence repeats (SSRs). In total, 1,342 perfect SSRs with a motif length of two to five nucleotides were detected within the 4,282,332 bp analyzed, corresponding to one SSR every 3,200 bp. A previous study had already shown the density of SSRs within the oak genome to be high. As previously described for BAC end sequences[16], AT/TA dinucleotide motifs were the most abundant.

Repeat masking resulted in the masking of 24.7% of the BAC sequences, 99% of which corresponded to transposable elements (TEs), the other 1% corresponding to other types of sequence repeats. The percentage repeat content varied from 32% (clone #138D21) to 49% (#108022). Retrotransposons were the most abundant repeated elements, with 27% of the Gypsy type and 15% of the Copia type (**Supplementary Table 23**). A *de novo* repeat search detected 38.5% TEs, a value lower than that estimated for the whole genome (52%).

Putative genes were predicted with a combination of Eugene, trained on the oak genome, FGENESH and Augustus (**Supplementary Table 24)**. In total, 322 gene models were predicted. Manual annotation was performed, with BLAST queries against NCBI non-redundant proteins, oak unigenes and oak ESTs available from the NCBI Short Read Archive. Exon and intron structures were manually optimized on the basis of evidence for splice sites. After manual curation, 44 of the 322 predicted genes were found to be located at the end of BAC sequences and were not curated, 28 were deleted (corresponding to gene models encoding < 50 amino acids), and intron/exon structure remained unresolved in 50, which were therefore considered “problematic”. Thus, 200 predicted genes with a resolved intron/exon structure were finally approved. Intron/exon structure was modified for 37 of these 200 genes, merged for 25 genes, and 138 genes (i.e. 69%) had already been accurately predicted by the automatic annotation procedure described in section 3.3. This proportion is close to that of validated CDS from the set of 1,714 manually curated genes (79%, see section 3.5). We thus

found a mean of six genes per 100 kb, corresponding to one one protein-coding gene per 16.7 kb **(Supplementary Table 24**), a value twice the mean gene density across the genome (3.2 genes/100 kb). This bias probably results from the selection of genes for insertion into BACs.

Gene function was assigned on the basis of sequence identity to proteins within the phytozome and NCBI non-redundant protein database and/or the presence of Pfam domains (**Supplementary Data Set 10 sheet #2**).

2.1.3. Comparison of genomic structure between haplotypes

Primer pairs of mapped simple sequence repeat (SSR) markers, PIE033, PIE260, PIE275, PIE257, and ZQR111 and the CL4 candidate gene, were used to screen the two BAC libraries (**Supplementary Data Set 10 sheet #1**) for allelic BAC identification. Allelic BAC clones were identified by sequencing of the PCR products. Six sets of homologous BAC clones (50E24-177A20/38C23, 27L3-48K1/72H20, 5E10-107I07, 64H03-30P1, 4E16/12J1-121F1, and 4N17-11F22) were selected and sequenced. Except for BACs 111F22 and 64H03, an analysis of BAC sequences confirmed the presence of the markers within the BAC clones. The sequences of BACs 4E16 and 12J1 overlapped fully. We therefore removed 4E16 from further analyses. Surprisingly, 4N17 and 111F22 did not overlap, and neither was therefore considered in subsequent analyses. The overlap between the remaining allelic BACs ranged from 22 kb to 84 kb and the mean sequence identity in overlapping regions was 97% (Evalue =0.0) (**Supplementary Table 25** and **Supplementary Table 26**). Pairwise sequence alignment revealed insertions and deletions within the intergenic regions, for all pairs (**Supplementary Fig. 26**). We identified TE insertion/deletion as the main factor accounting for the considerable structural polymorphism observed between allelic BACs. Gene order and structure were nevertheless well conserved.

2.2. Comparison of the V1 and V2 assemblies and assembly validation

We compared the previous release (version 1: V1[19]) of the diploid assembly with the current release obtained by the addition of synthetic long reads generated by highly parallel library preparation and the local assembly of short read data. Standard metrics revealed a huge difference in terms of contiguity (see **Supplementary Table 27**). Indeed, the N90 of our assembly was six times better than that of the previous assembly, and the proportion of ambiguous bases was only 4.6% for our assembly, whereas it was 11.6% for the previous assembly. We used Busco to assess the degree of gene completion for the two assemblies. The V2 release presented a completeness of 90.8% (**Supplementary Table 27**), i.e. greater than the V1 assembly (90.4%). Standard metrics also suggested that haplotypes were better resolved in the V2 release (as indicated by the cumulative sizes of the assemblies). We then validated the better differentiation between the two haplotypes of the V2 assembly, by mapping a dataset of Illumina paired-end reads (2x250 bp) on both assemblies. Collapsing the two haplotypes should increase the observed coverage by a factor of 2, whereas keeping the two haplotypes separate should yield identical observed and expected coverages. As expected, we observed fewer regions with twice the coverage in the V2 release (**Supplementary Fig. 1**). We also aligned the V1 (**Supplementary Fig. 27, Supplementary Fig. 28, Supplementary Fig. 29**) and V2 (**Supplementary Fig. 11, Supplementary Fig. 12, Supplementary Fig. 13**) releases with three pairs of BACs, each pair corresponding to the two alleles of the same genomic region. We first aligned the whole assembly against each BAC with BLAT alignment tool[29] and default parameters, retaining only the scaffold with the highest alignment score. Alignment positions were then extracted from a NUCmer[30] alignment (identity > 90%) between each BAC and the corresponding scaffold. We selected SNPs between allelic BACs, using a sliding window of 100 bp, and we used a seed sequence of 41 bp (20 bp on either side of the SNP) to retrieve the allelic variants of the scaffolds. We

generated graphical representations (see **Supplementary Fig. 11, Supplementary Fig. 12, Supplementary Fig. 13, Supplementary Fig. 27, Supplementary Fig. 28, Supplementary Fig. 29**) highlighting the advantages of long reads for differentiating between haplotypes. The V1 release often merged the two haplotypes into a single scaffold for the three genomic regions, whereas the V2 release contained a pair of scaffolds for each pair of BACs. Furthermore, the V2 scaffolds showed fewer switches between haplotypes than the V1 release (see **Supplementary Fig. 11, Supplementary Fig. 12, Supplementary Fig. 13, Supplementary Fig. 27, Supplementary Fig. 28, Supplementary Fig. 29**).

2.3. Pseudomolecule construction

Scaffolding can extend the contiguity of a genome sequence assembly by orders of magnitude relative to contigs, but the construction of a chromosome-scale genome requires either physical or genetic maps to anchor the scaffolds. We used a genetic map for this purpose. A composite genetic map was first established with LPmerge software[31], bringing together 5,589 already mapped EST-SSR and SNP markers from eight individual linkage maps[17,32] (**Supplementary Data Set 2 sheet #1**), including one map for accession '3P' used to establish the reference genome sequence. Gene model sequences for the 5,589 mapped loci were then aligned with the 1,409 scaffold sequences, using BLAT[29] ($\geq$ 95% identity). In total, 2,615 unique scaffold/marker relationships (**Supplementary Data Set 2 sheet #2**) were identified and classified into four categories (**Supplementary Table 28**). Overall, the scaffold-anchoring strategy (taking into account 2,285 markers from the most reliable assigned scaffolds, i.e. from categories 1, 2 and 3) delivered 612 (43%) anchored scaffolds, covering 624.8 Mb (77%) of the haplome. Additional scaffolds were then anchored onto the 12 oak linkage groups, according to the synteny-driven strategy illustrated in **Supplementary Fig. 2** (see Pont et al.[33] for the details). To this end, the 1,409 scaffold sequences were aligned (BLAST-n, >70% identity) with the eight chromosomal sequences of *Prunus persica* (a

species phylogenetically related to *Q. robur*). This approach yielded a set of 653 scaffolds, including 259 scaffolds anchored by synteny only (i.e. locally ordered according to the gene order in *Prunus persica*), and 394 scaffolds already anchored and ordered with markers (**Supplementary Data Set 2 sheet #3**). These scaffolds highlighted links shared between the peach genome and the oak map and made it possible to intercalate the 259 scaffolds initially anchored on the basis of synteny alone. Using the second set of 394 scaffolds, and comparing gene order between the *Prunus* and *Quercus* genomes, we estimated the accuracy of the syntenomic approach for the correct positioning and orientation of the first set of scaffolds (anchored on the basis of synteny alone) at 86%. The 12 oak pseudomolecules (hereafter referred to as chromosomes and numbered according to the SNP-based linkage map[32]) were then constructed on the basis of 871 (62%) anchored and oriented scaffolds, with the filling in of 100-nucleotide tracts between consecutive scaffolds (**Supplementary Data Set 2 sheet #4**): i) 218 scaffolds anchored and ordered by genetic markers only, ii) 259 scaffolds anchored by synteny only, with local ordering according to gene order in peach, and iii) 394 scaffolds anchored and ordered by both procedures. Overall, the 871 scaffolds cover 716.6 Mb (i.e. 88% of the haplome) and contain 23,220 (90%) genes. The 12 chromosomes and the 538 unanchored scaffolds are available from the oak genome JBrowse (https://urgi.versailles.inra.fr/WebApollo_oak_PM1N/PseudoMolecule.html).

Based on scaffold order and orientation on the 12 chromosomes, the oak genome browser was populated with a “marker” track including an optimized set of markers tolerant of inversions between physical and genetic positions within a maximum window of 5 cM. This track was designed to project the position of any quantitative trait locus (QTL) from the eight individual linkage maps onto the oak genome sequence, to facilitate subsequent biological interpretation of their genetic bases. The track was created according to the procedure described in **Supplementary Fig. 30**. We found that 2,127 of the 2,615 markers (retained for scaffold

anchoring) fitted the criteria presented in **Supplementary Fig. 30** (referred to as set#1 in **Supplementary Data Set 2 sheet #1**), and 1,943 were retained from the other set of 2,974 markers initially excluded from scaffold anchoring (set#2 in **Supplementary Data Set 2 sheet #1**). As a result, the "marker" track included 4,070 markers spanning the 12 linkage groups (red horizontal lines in **Supplementary Fig. 31**). The alignment of each marker set with the 12 chromosomes is shown in **Supplementary Fig. 32**. Overall, the rank correlation between genetic and physical positions ranged from 0.991 to 0.999 (**Supplementary Table 29**).

3. Genome annotation

3.1. Detection and annotation of transposable elements

As in other sequenced plant genomes, the class I retrotransposon fraction predominated (70% of TE sequences), consisting of 53% LTRs (long terminal repeats: 26% Gypsy-like and 21% Copia-like) and 16% non-LTR retrotransposons (mostly LINE). Class II DNA transposons accounted for 15% of TE sequences, and 92% of the transposons in this fraction were TIRs (terminal inverted repeats) (**Supplementary Fig. 3**, **Supplementary Table 4**).

Thirteen of the 1,750 consensus sequences (0.6% of the TE content) were further characterized as Caulimoviridae sequences (see section 3.2). **Supplementary Table 30** shows a comparative analysis of TEs across the 16 species (including oak) used for the comparative genomic analysis in section 4. We found no correlation between TE content and the phylogeny of these species (based on NCBI Taxonomy Browser findings) (**Supplementary Fig. 33**).

3.2. Identification and preliminary characterization of endogenous Caulimoviridae

Plant viruses can have a major impact on the populations and genomes of their hosts. Paleovirology approaches can provide insight into virus-host associations by detecting fragments of viral genomes integrated in host genomes[34]. Caulimoviridae is a major family of plant viruses with deleterious effects on plant populations and crop production[35]. Caulimoviridae do not need to integrate into the host genome during their replication cycle, but such integration occurs randomly and repeatedly, resulting in the presence of significant numbers of Caulimoviridae genome fragments in plant genomes[36,37]. We screened the oak genome for the presence of genomic fragments from endogenous Caulimoviridae. Reverse transcriptase (RT) is the best conserved domain of the Caulimoviridae family, so we began by searching the oak genome for RT domains displaying the highest levels of identity to homologs from known Caulimoviridae genera. Protein clustering (>80% identity) identified eight groups including seven comprising several sequences corresponding to RT sequences from Caulimoviridae. This viral family contains eight genera. Phylogenetic analysis revealed that one of the RT cluster from endogenous oak Caulimoviridae belonged to the genera *Petuvirus*, whereas the other seven belonged to the recently discovered *Florendovirus* genera[37] (**Supplementary Fig. 34**).

We then performed targeted clustering (98% identity and 95% length) on the nucleotide sequences corresponding to putative Caulimoviridae loci in the oak assembly and built consensus sequences based on the multiple sequence alignment (MSA) for each cluster. We then clustered the consensus sequences with the closest evolutionary relationships to Caulimoviridae into seven families, each of which displayed at least 90% local identity. In five families, the longest consensus sequence accounted for a complete, or almost complete Caulimoviridae genome and was, thereafter, considered the representative sequence for each family. Remarkably, we noticed that, while representative consensus sequences were built

from the MSA of only a few highly similar copies, we found cases in which consensus sequences corresponding to truncated variants of the representative Caulimoviridae genomes were generated from the MSA of hundreds of almost identical copies (**Supplementary Fig. 35**). We compared the representative sequences with the library of repetitive elements built by TEdenovo and found that most were well represented in this library (see section 3.1). Collectively, copies of consensus sequences from the TEdenovo library corresponding to fragments of the Caulimoviridae genome accounted for 4.4 Mb of the REPET annotation, 0.6 % of the haplome, and were distributed evenly over the 12 chromosomes (**Supplementary Fig. 36**).

3.3. Gene prediction and functional annotation of protein-encoding genes

We retained a core set of 25,808 high-confidence genes (listed in **Supplementary Data Set 1**). The total gene space was 74 Mb in size, with a density of 0.32 genes/10 kb on average **(Supplementary Table 31**). This density is lower than that reported for other species, such as *A. thaliana* (2.3 genes /10 kb; TAIR 10[38]), *P. persica* (1.22 genes /10 kb[39]), *M. domestica* (0.78 genes /10 kb[40]), but similar to that for species with a similar genome size and TE content, such as *E. grandis* (817 Mb; 50% TE; 0.45 genes /10 kb[41]), *C. papaya* (815 Mb; 52% TE; 0.34 genes/10 kb[42]) and *C. clementina* (816 Mb; 43% TE; 0.3 genes /10 kb; [43]). Overall, 99% of the predicted *Q. robur* genes (i.e. 25,516) were found to encode proteins, with at least domain/motif, localization/targeting signal, or similarity-based evidence (**Supplementary Fig. 37**).

3.4. TEs and genome dynamics

3.4.1. Estimation of the age of TE families from consensus sequences

The consensus sequences used to annotate the TE copies in the oak *g*enome represent common ancestral structural variants (TE families) of TEs transposing in the oak genome in

the past[44]. Indeed, they were constructed from highly repeated genome segments (see section 3.1). We investigated the evolution of TEs in the oak genome, by plotting and comparing the observed divergence (1-identity %) of TE copies from their respective consensus sequences, to estimate their relative age[45]. We performed this analysis separately for different orders of TEs (LTR and Non-LTR retrotransposons, TIRs and Helitron DNA transposons) and superfamilies (LTR Gypsy and Copia superfamiles). Most TE copies (i.e. 62% representing 44% of the TE space) displayed more than 15% divergence from the corresponding consensus sequence (**Supplementary Fig. 38**), whereas only 6.7% of TE copies (17% of the TE space) displayed low levels of divergence (<5%) from their respective consensus. This result suggests that all the TEs present in the oak genome are relatively ancient, contrasting with findings for *A. thaliana*, in which 73% of TE copies (52% of the TE space) display more than 15% divergence and 10.5% of the copies (26% of the TE space) display less than 5% divergence[45]. By contrast, the divergence of the LTR retroelement superfamilies Gypsy and Copia suggests that TE activity continued until fairly recently for the elements of these families.

3.4.2. **Retrotransposition dynamics**

We first refined the annotation of LTR retrotransposons with the dedicated LTR Harvest tool[46], retaining the 5,904 complete elements that displayed more than 90% reciprocal overlap with those from the general annotation of TEs with the REPET pipeline. We then classified these elements into families by sequence clustering of their left LTRs with SiLiX, as previously described[47]. We analyzed retrotranspositional history on a subset of 4,333 elements from families of more than 200 elements. The insertion date of each element was calculated from the sequence similarity between its left and right LTRs, as determined by LTR Harvest, as follows: $\text{date} = ((1 - (\%\ \text{identity}/100)) / 2.6) \times 10^{8}$ [48]. We plotted the data as density histograms representing the distribution of insertion dates within each family, together with a

curve representing local density estimates (**Supplementary Fig. 39)**. We observed a general asynchronism of retrotranspositional history, with families displaying one of three contrasting patterns of activity history: ancient (e.g. fam #6 and #10), constant (e.g. fam #8) or recent (e.g. fam #12, #14 and #29). This result suggests that the complexity of the oak genome developed through repeated bursts of retrotransposition over the last five million years, with no clear increase in such activity in the recent past. These findings differ from those for annual plants of similar genome size, for which genome complexification has occurred more recently, through concomitant bursts of transposition (over the last 1-2 million years[49]).

3.4.3. Distribution of TEs and genes in the oak genome

TEs are often associated with genome rearrangements. They have been found in breakpoint-containing windows in comparisons of *A. thaliana* and *A. lyrata*[38]. In maize and *A. thaliana,* the pericentromeric regions of the chromosomes are highly enriched in LTR retrotransposons of the Gypsy superfamily, and maize also displays an accumulation of TEs from the Copia superfamily in regions of euchromatin[50,51]. We investigated whether TEs, particularly those of the Gypsy and Copia superfamilies, were evenly distributed throughout the oak genome. We calculated the percentage of TEs and annotated genes in sliding windows (300 kb, with a 200 kb overlap). We found that TEs accumulated in gene-poor regions. We also identified a region of chromosome #2 displaying strong TE accumulation, potentially corresponding to the centromeric region (**Supplementary Fig. 40**). The Copia elements tended to accumulate away from the potential centromere, both upstream and downstream. This pattern was particularly marked for chromosome #2 (**Supplementary Fig. 41**). Below, we consider the potential role of TEs located in close proximity to genes.

3.4.4. **Role of TEs in gene expansion and tandem duplication**

We investigated the possible role of TEs in gene duplication and/or gene family expansion, by comparing the genomic environment (in terms of proximity to TEs) of several categories of genes according to their distance to the closest TE. The distance from each gene to the closest TE was calculated with getDistance.py from the S-MART package[52]. Only distances up to 5 kb were considered. We assessed the dependence between the different classes of distances and belonging to an expanded gene family, for oak genes. We found that genes from expanded gene families (see section 4.1.2) were closer to TEs than other genes (Chi-squared p-value $< 2.2e^{-16}$; **Supplementary Fig. 42**). TE-mediated gene family expansion has been described in multiple species[53,54]. We obtained similar results for tests of the dependence of different classes of distances and membership of the TDG (tandem duplicated genes), LDG (long distance-duplicated genes) and SG (singleton genes) classes. TDGs were closer to TEs than SGs or LDGs (Chi-squared p-value $< 2.2e^{-16}$; illustrated for SG in **Supplementary Fig. 43**), but no significant difference was observed for comparisons of LGD and membership of the SG class. This result suggests that TEs may favor tandem duplications leading to gene family expansion.

3.4.5. **Horizontal transfer of TEs**

We studied the horizontal transfer of TEs (HTT), by performing an *in silico* analysis on all plant genomes available from the NCBI and Phytozome databases, focusing on LTR retrotransposons. We chose one element for each family identified in the annotation step. BLAST-n searches were performed to identify high levels of nucleotide sequence identity, with the NCBI nr (http://www.ncbi.nlm.nih.gov/) and Phytozome v9.0 (http://www.phytozome.net/) databases. Candidates for HTT (listed in **Supplementary Table 32**) were detected by applying a 90% identity threshold[47], to ensure that we detected

horizontally, as opposed to vertically inherited TE sequences. Eight horizontal transfers of LTR-retrotransposons were identified. All potential candidates were validated by checking that the LTR retrotransposon sequences were located on large contigs and not on isolated, short sequences in genome assemblies, and that the high degree of sequence identity was limited to the elements themselves and not in their flanking sequences, to eliminate possible contamination during genome assemblies and annotation errors. Moreover, analysis with Dotter software confirmed that all the horizontally transferred elements harbored both the LTR and an internal sequence in the two species involved. We identified six HTT events involving oak and grapevine (*Vitis vinifera*), with sequence identities of 90 to 94%. We found one HTT event involving oak, grapevine and peach (*Prunus persica*). The HTT event between grapevine and peach had already been identified (BO6) in the analysis by El Baidouri et al.[47], and 92% identity was found between the corresponding sequences from the two species. We also identified one HTT event between oak and poplar (*Populus trichocarpa*), with 91% identity. HTTs have been shown to occur frequently between flowering plants[47], but our findings for the oak genome provide the first evidence of multiple HTTs in a single species.

3.5. Gene prediction, functional annotation of protein-encoding genes and manual curation

The total gene space of the 25,808 predicted proteins was 74 Mb in size, with a density of 0.32 genes/10 kb on average **(Supplementary Table 31**). This density is lower than that reported for other species, such as *A. thaliana* (2.3 genes /10 kb; TAIR10 [38]), *P. persica* (1.22 genes /10 kb[39]), *M. domestica* (0.78 genes /10 kb[40]), but similar to that for species with a similar genome size and TE content, such as *E. grandis* (817 Mb; 50% TE; 0.45 genes /10 kb[41]), *C. papaya* (815 Mb; 52% TE; 0.34 genes/10 kb[42]) and *C. clementina* (816 Mb; 43% TE; 0.3 genes /10 kb[43]). Overall, 99% of the predicted *Q. robur* genes (i.e. 25,516) were

found to encode proteins, with at least domain/motif, localization/targeting signal, or similarity-based evidence (**Supplementary Fig. 37**).

Experts manually checked (using WebAppolo) the protein-coding sequence structures of 1,714 mRNAs. They validated 79% of the transcripts without the need for additional modification, whereas the remaining 21% had to be corrected (**Supplementary Table 12**). We then aligned the coding sequences of these 1,714 mRNAs, to validate 2,067 genes of the *Q. robur* genome (diploid V2). Finally, 1,176 of these 2,067 genes were recovered in the *Q. robur* haplome. In the following sections we provide information concerning some of the gene families manually curated.

3.5.1. **Aquaporin**

Forty genes encoding putative aquaporins were identified in the *Q. robur* haplome. Aquaporins are intrinsic channel proteins found in all organisms. Their overall structure is highly conserved, with six transmembrane helices connected by five loops, a tetrad of amino-acids (helix 2, helix 5 and loop E) forming an aromatic/arginine constriction region (Ar/R filter), and two membrane embedded half-helices with an asparagine-proline-alanine signature (NPA motif)[55,56]. Five conserved amino-acid residues discriminated aquaporins from other major intrinsic proteins[57]. One *Q. robur* gene was invalidated due to the absence of a key signature (**Supplementary Table 33**).

Q. robur was found to have aquaporins from the five subfamilies found in higher plants (**Supplementary Fig. 44**), with 14 plasma membrane-intrinsic proteins (PIPs), nine tonoplast-intrinsic proteins (TIPs), eight nodulin-26 intrinsic proteins (NIPs), three small basic intrinsic proteins (SIPs) and five unrecognized X intrinsic proteins (XIPs). Two subclasses of XIPs were identified in *Q. robur*, with a particular mapping pattern for *XIP2*, suggestive of local amplification on Qrob_H2.3_Sc0000154. Except for the XIPs, the composition of the *Q.*

robur aquaporin family was similar to those of *Arabidopsis* and maize[58,59]. However, the full-length *TIP3* gene was missing from the *Q. robur* genome. In several species, TIP3s have been reported to be specific to maturing and dry seeds[60]. Variations at key motifs and in gene structure between the *Q. robur* aquaporin subclasses were consistent with published findings[58,61]. The global rate of tandem duplication in this gene family was 37.5%, which similar to the overall rate for the oak genome (35.6%).

3.5.2. **MYB**

MYB genes are characterized by a highly conserved DNA-binding domain (MYB domain) consisting of up to four imperfect repeats of a sequence of about 52 amino acids in length (R). They constitute one of the largest families of transcription factors in plants, with members regulating many key biological processes, including cell fate, developmental processes, primary and secondary metabolism, and responses to biotic and abiotic stresses[62]. MYB proteins can be classified into several classes on the basis of the number of contiguous repeats of the MYB domain. The most abundant of these classes contains MYB proteins with two repeats of the MYB domain (R2R3-MYBs).

We identified 139 R2R3-MYBs, five 3R-MYBs and one 4R-MYB (**Supplementary Table 34**). This distribution of MYB proteins is similar to that in other species, such as *A. thaliana* (126 R2R3-MYBs, five 3R-MYBs, and one 4R-MYB), *E. grandis* (141 R2R3-MYBs) and *V. vinifera* (123 R2R3-MYBs). We performed a comparative phylogenetic analysis of the R2R3-MYB sequences from *Q. robur*, *P. trichocarpa*, *E. grandis*, *V. vinifera*, *A. thaliana* and *O. sativa* (**Supplementary Fig. 45**). The topology of the phylogenetic tree was similar to that described for *Arabidopsis*[62], with most of the subgroups conserved. However, like other woody perennial plants, oak presented subgroups with more members than in herbaceous annual plants such as *Arabidopsis* or rice (**Supplementary Fig. 45)**. These expanded clusters in woody plants include the so-called “woody preferential subgroups”, which are completly

absent from the basal lineages of bryophytes and lycophytes and from the more recent Brassicaceae and Monocot lineages[63]. We investigated the possible role of the MYB gene family in tree habit specialization by classifying R2R3-MYB genes according to their duplication and expansion profiles in woody perennials. The global rate of tandem duplication in the R2R3-MYB family (32.4%) was slightly lower than the overall rate for the oak genome (35.6%). However, the tandemly duplicated MYBs were remarkably enriched within the woody-expanded subgroups (**Supplementary Fig. 46**, Fisher's exact test p-value < 0.0001). A substantial enrichment of tandemly duplicated genes belonging to woody expanded subgroups has been also observed in other woody plants, such as eucalyptus, poplar and grapevine[64].

The few genes from subgroups expanded in woody perennials that have been characterized seem to regulate phenylpropanoid metabolism, mostly controlling flavonoid biosynthesis, although, in some cases, they also directly or indirectly alter the content of lignin and other soluble compounds, such as oligolignols or salicinoid phenolic glucosides[64–69]. During evolution, tandemly duplicated genes have a greater likelihood of being retained if they are involved in responses to environmental factors[70]. Unlike herbaceous annuals, which die after reproduction, perennial plants, such as trees and shrubs, must survive many periods of challenging stressful environmental conditions over their long lifespans. Woody perennial plants may, therefore, contain more elaborate stress resistance mechanisms. The large number of tandemly duplicated genes regulating the biosynthesis of flavonoids and other phenylpropanoid-derived compounds, mostly known to be protective, may enable oak trees to develop complex protective mechanisms and to adapt woody growth to environmental conditions. It is also possible that the production of the some of the many phenolic compounds accumulating in oak heartwood, such as ellagitannins, or the gallotannins found in oak galls, are controlled by these genes.

3.5.3. **SWEET**

The host plant supplies the mycorrhizal fungi with hexoses, which support the production of the external fungal mycelium, a prerequisite for effective nutrient acquisition by hyphal networks. Plant sugar transporters of the SWEET superfamily deliver sugars to microbes[71], and the microbe-specific modulation of *SWEET* gene expression may alter sugar efflux at the site of colonization[72]. We therefore analyzed the phylogeny of the pedunculate oak SWEET superfamily, and performed RNAseq analyses to determine whether the abundances of *Q. robur SWEET* transcripts were altered by inoculation of the oak clone DF159 with the ectomycorrhizal fungus (EMF) *Piloderma croceum*[73], the mycorrhizal helper bacterium (MHB) *Streptomyces* sp. AcH 505, and the causal agent of oak powdery mildew, *Erysiphe alphitoides*[74]. Oak clone DF159 was micropropagated and rooted for gene expression analysis as described by Herrmann et al.[75], and cultivated in gamma-sterilized soil-based microcosms, as described by Herrmann et al.[74]. Culture and inoculation conditions, RNA extraction, sequencing and data processing for fungi and bacteria were as described by Tarkka et al.[73], Kurth et al.[76] and Herrmann et al.[74]. Sequence data were deposited in the NCBI Short Read Archive (accessions for *P. croceum*: SRX383906, SRX383899, SRX383898, SRX798260, SRX798261, SRX798262; for AcH 505: SRX976815, SRX976817, SRX976819, SRX976827, SRX976829, SRX976831; for *E. alphitoides*: SRX2398909, SRX2398916, SRX2398917, SRX2398913). *T. magnatum* ectomycorrhizae were sampled on 6-month-old inoculated *Q. robur* plantlets produced by Robin nurseries (St Laurent du Cros, France), following their standard protocols. Total RNA was extracted from ectomycorrhizal root tips of *T. magnatum/Q.robur* using the RNeasy Plant Mini Kit of Qiagen with DNase step and addition of 20 mg/ml polyethylene glycol to the RLC extraction buffer. Three replicates were used for RNA-seq. Preparation of libraries from total RNA and 2 x 100bp Illumina HiSeq sequencing (RNA-Seq) was performed at the GET platform (Génopole Toulouse Midi-

Pyrénées, Auzeville, France) following their standard protocol. Quality filtered reads were aligned to the *Q.robur* haplome reference transcripts using CLC Genomics Workbench 9 (Qiagen). To identify transcripts differentially regulated in ectomycorrhizae compared to control roots (greenhouse grown non-mycorrhizal roots; ERX1916509-11) the test from Baggerly et al. implemented in CLC Genomic Workbench and *p*-values from the differential expression tests were adjusted for false discovery rate (Benjamini & Hochberg). The *T. magnatum* RNA-Seq data are available at NCBI/GEO as Series GSE97122.

We identified 14 *SWEET* genes in the oak genome (**Supplementary Fig. 47**), belonging to the four clades identified in *A. thaliana*[71]. Clade IV seems to have been expanded, with six members in oak, *versus* only one in *Malus domestica*, two in *Arabidopsis thaliana* (SWEET16 and SWEET17), two in *Eucalyptus grandis* and three in *Solanum tuberosum*. Biochemical characterization of the SWEETs of *Arabidopsis thaliana* showed that the members of clade I and II preferentially encoded monosaccharide transporters, whereas the members of clade III encoded disaccharide transporters, mostly for sucrose[71,77].

In total, five *SWEET* genes from clades I, III and IV were differentially expressed in oak with the EMF *Piloderma croceum* and *Tuber magnatum,* the MHB *Streptomyces* sp. AcH 505, or *Erysiphe alphitoides*. Oak clade I transcript *Qrob_P322480.2,* homologous to *SWEET1,* was upregulated by the EMF *P. croceum* and *T. magnatum*. Consistent with these findings, arbuscular mycorrhiza (AM) formation also leads to *SWEET1* induction in potato and in *Medicago truncatula*[78]. By contrast, the abundance of the oak *SWEET1* transcript was decreased by *Erysiphe alphitoides* infection or inoculation with the MHB *Streptomyces* sp. AcH 505. The *SWEET1* gene therefore displayed differential regulation as a function of the biotic interaction.

The oak clade I *SWEET3* homolog *Qrob_P321700.2* was induced by *P. croceum* and *Streptomyces*, and a related gene was among those upregulated in the potato AM symbiosis[79].

All the clade III SWEETs have sucrose transporter activity, and the oak clade III transcript *Qrob_P657550.2,* homologous to *SWEET12,* was upregulated upon interaction with *P. croceum* and *Streptomyces*. Interestingly, a related gene is upregulated in the protocorms of the orchid *Serapias vomeracea* during interaction with an orchid mycorrhizal fungus[80]. By contrast, most of the transcripts repressed in the arbuscular mycorrhizal symbiosis of potato corresponded to clade III SWEETs[79], suggesting that clade III SWEETs are differentially regulated in different types of mycorrhizal interactions.

Clade IV SWEETs are vacuolar glucose, fructose and sucrose carriers in *A. thaliana*[81]. The oak clade IV *SWEET17* homolog *Qrob_P216890.2* was upregulated upon interaction with *P. croceum* and *Streptomyces*, as also reported for the closely related potato *StSWEET17a* and *StSWEET17b* in AM symbiosis[79]. By contrast, *Streptomyces* treatment led to the downregulation of *Qrob_P546940.2* in leaves, and the expression of a related gene, *StSWEET17c,* was suppressed in potato AM symbiosis[79]. Different expression patterns were observed for clade IV genes during mycorrhizal interactions with oak, suggesting that *SWEET17* genes are regulated in a complex manner in beneficial symbioses. Thus, the predicted expansion of clade IV *SWEET* sugar efflux carrier genes in the oak genome and the differential abundances of oak *SWEET* transcripts, may reflect the adaptation of oak to a remarkably rich spectrum of biotic interactions.

3.5.4. **Thioredoxin, glutaredoxin and glutathione transferase**

Redox changes are major cellular disturbances that affect a range of processes throughout the organism's lifetime, through their involvement in various stages of development and in stress responses, in particular. Post-translational redox modifications of proteins are increasingly being recognized as a rapid, targeted mechanism for initiating cellular responses in a very short timeframe[82]. For example, light is known to control carbon metabolism enzymes through a cascade of electron exchange reactions, including dithiol-disulfide exchanges.

Within cells, these reactions are controlled by thioredoxins (TRX) and glutaredoxins (GRX), encoded by two multigenic families containing 20 to 40 genes and constituting the reducing systems[83,84]. Different isoforms are often present in different subcellular compartments, probably because they catalyze different reactions or have different protein partners. In addition to these regulatory functions, TRX and GRX are also required for the regeneration of some detoxification enzymes, particularly those requiring a reactive catalytic cysteine residue. This residue oxidized upon reaction with the substrate, as in peroxiredoxins and methionine sulfoxide reductases, must be recycled for the next turnover. We have also investigated the glutathione transferase (GST) family, the members of which have certain structural and biochemical features in common with GRXs. An analysis of the possible expansion of this gene family was also particularly enlightening, because its members are involved in secondary metabolism and in xenobiotic detoxification, and display transcriptional regulation in very diverse stress conditions. The TRX, GRX and GST gene content of *Q. robur* was thus analyzed at the level of defined subclasses, with comparisons with other photosynthetic organisms, including multiple tree species (*Populus trichocarpa*, *Prunus persica*, *Citrus clementina* and *Eucalyptus grandis*) (**Supplementary Table 35**). In most pairwise comparisons, the number of genes remained remarkably constant, indicating that these systems are essential for plants.

The genes of the GRX family displayed the greatest variation in number in plant-specific class III, with 9 to 24 genes in angiosperms, the oak genome having an average number of these genes (N=14), 50% of them being tandem-duplicated. Hence, variations in this class can be accounted for mostly by species-specific duplications. Similarly, the oak genome has a number of genes from the TRX/TRX reductase family similar to that in other organisms, and none of the subclasses are missing. The most striking characteristic is the presence of six genes for NADPH-TRX reductase a/b type (NTRa/b), rather than the one or two in other

species, with four of the genes in oak identified as tandem duplicate genes. In line with this observed expansion, the associated orthogroup (#2778) was found to be enriched in GO term (GO:0004791) analysis.

Fourteen classes of GST genes were identified in the last phylogenetic analysis performed with photosynthetic organisms[85]. Only 11 of these classes are present in angiosperms, the other three classes being found in *Physcomitrella patens*. The total number of genes (88) in oak is in the upper part of the range, as are those for *P. trichocarpa*, *E. grandis* and *S. bicolor*. A detailed subclass analysis revealed that the difference between oak and other organisms resulted principally from the presence of a larger number of GST Tau (GSTU) family members. This finding was confirmed by the orthoMCL analysis (see section 4.1.2), with an expansion observed for four clusters, including one with 19 GSTU genes (red branches in **Supplementary Fig. 48**). From this very variable gene content (there is no GSTU in *P. patens,* but 21 to 62 GSTU genes in the analyzed angiosperms) and the presented phylogenetic tree (many sequences cluster by species), it seems clear that GSTU genes evolved relatively recently and in a species-specific manner in plants. This situation differs from that for most GRX and TRX classes, for which photosynthetic organisms usually have the same number of each isoform (**Supplementary Table 35**)[83,84]. This difference is also highlighted by the 76.1% rate of tandem duplication for the GST family (mostly due to the largest classes, GSTF and GSTU), much higher than the 22.0% and 28.0% reported for the TRX/TRX reductase and GRX families, respectively, and the value obtained for the oak genome (35.6%).

3.5.5. **MLO**

Studies of the MLO (mildew locus O) family of disease resistance genes are particularly relevant in *Quercus,* the plant genus infected by the largest number of powdery mildew

species (16 from six different genera[86]). This large group of obligate plant pathogenic fungi infects almost 10,000 species of angiosperms[87]. The first MLO gene was isolated from barley[88,89]. It was found to act as a susceptibility gene, with recessive loss-of-function alleles (mlo) associated with broad-spectrum resistance (i.e. to all genotypes/races) to the fungal pathogen *Erysiphe graminis f. sp. hordei*, one of the causal agents of powdery mildew[90]. Unlike many of the resistance genes used in crop plants, which have been rapidly overcome by virulent races of pathogens after deployment, *mlo* resistance has remained durable in the field for decades, despite its widespread use. Mildew-resistant *mlo* mutants have also been described in *Arabidopsis thaliana*, tomato and pea[91]. The *Mlo* gene encodes a protein of unknown biochemical activity, with seven transmembrane domains, located at the plasma membrane. *Mlo* genes have been found into small families (often about 15 genes) in the genomes of many higher plant species, including *Prunus persica*[92], *Vitis vinifera*, *Cucumis sativus*, and others[93]. Functional studies in *Arabidopsis* have shown that MLO function is not restricted to plant–powdery mildew interactions. Instead, these proteins are also involved in pollen perception[94] and root thigmotropism[91].

We found 19 MLO genes in the haplome of *Q. robur* (**Supplementary Table 36, Supplementary Table 37, Supplementary Fig. 49**), including seven belonging to clade V. This clade contains the genes associated with powdery mildew susceptibility/resistance in *Arabidopsis thaliana*[95] and some other species. The large number of MLO genes in oak, particularly in clade V, is only surpassed by soybean, cotton and apple, all of which have undergone recent whole-genome duplication events. Most of the MLO genes are located on chromosomes #8 (5 genes, 4 of which belong to clade V), #10 (4 and 1) and #1 (3 and 2). We also found seven incomplete genes with strong homology to MLO. As MLO genes are susceptibility genes, these incomplete genes may confer resistance[90]. There are three incomplete genes on chromosome #10, at the 5' and 3' ends of a complete clade V *mlo* gene.

If we consider all complete and partial genes, the overall rate of tandem duplication of MLO genes is 46.2%, slightly higher than the overall rate for the oak genome (35.6%).

3.5.6. **NB-LRR**

NLR-parser ([96], https://github.com/steuernb/NLR-Parser) was used to identify disease resistance genes encoding nucleotide-binding leucine-rich repeat proteins (NB-LRRs or NLRs) and related proteins from the oak genome (haplome). We identified an initial set of 1,431 genes. Based on the orthoMCL analysis, 81 proteins from NB-LRR-related classes (i.e. orthogroup #1000, 1004, 1015, 1031, 1084, 1140, 1187, 1269, 1540, 1697, 2368, 2399, 4549, 5011, 7397, 14497 and 15991, see **Supplementary Data Set 3 sheet #1**) were added to the set of putative disease resistance genes. The accuracy of domain prediction was checked with the NCBI Conserved Domains CD-search website ([97], Batch CD-search version). Each protein was manually inspected and attributed to a given category based on the presence of the following canonical domains: Toll-interleukin receptor-like (TIR), NB and LRR. The non-TIR domains found in oak putative NLRs consisted of coiled-coil (CC) and resistance to powdery mildew protein (RPW8) domains, referred to as CNL and RNL, respectively. Finally, we also recorded any other domains (X) potentially representing integrated domains[98,99]. After curation and removal of mispredictions, we recovered a total of 1,091 putative NB-LRR-related protein-encoding genes (**Supplementary Data Set 5**), 834 of which had a putative complete or partial NB domain and 54 showed a non-canonical and putative integrated domain. Many of these integrated domains, possibly acting as decoys for pathogen effectors[100,101], are DNA-interacting domains such as zinc-finger or Myb/SANT family domains. Other notable integrated domains had signaling functions (e.g. WD40) or were previously reported in secreted proteins from animal parasites and pathogens, i.e. the Rhomboid protease family (Pfam PF1694).

Beyond the large number of single domains retrieved (i.e. 14 CC, 151 TIR, 1 RPW8, 61 NB and 85 LRR, **Supplementary Table 8**), the total complement of NB-LRR genes in the oak genome is remarkable by comparison to those of other species. The list LRR genes is probably incomplete, as this category is inherently very difficult to characterize due to the highly variable number of LRRs and the abundance of other LRR-related proteins (e.g. LRR-RLK or LRR-RLP), a group that is also expanded in the oak genome. If we exclude single domains, then, for the 1,091 genes, TIR-related NB-LRR proteins account for 43% of the remaining disease resistance genes (335 of 779 genes). This ratio of TIR- to non-TIR- NB-LRRs close to 1 indicates that the disease resistance gene content of the oak genome is more balanced than reported for other eudicots[102–105]. One group of non-TIR NB-LRRs, the expanded set of RNLs (orthogroup #1140) may also reflect the evolutionary history of pedunculate oak with the fungus *Erysiphe alphitoides,* responsible for oak powdery mildew. No disease resistance gene for this disease has been identified and cloned or described in oak trees, but this gene complement suggests considerable potential for resistance to these pathogens and represents a valuable source of genetic information.

We investigated the expansions detected in the oak genome by the orthoMCL/CAFE analysis in more detail, by retrieving protein sequences with an NB domain from classes that displaying marked expansion relative to other plant species. We focused in particular on orthogroup #1000 (labeled as #1 in **Fig. 3d and 4b**). Multiple alignments were constructed for selected proteins, with the hmmalign program, from the HMMER 3.0 package[106], and the Pfam NB-ARC domain (PF00931) seed alignment converted into a hidden Markov model profile by hmmbuild. Collected NB domains were manually inspected and truncated domains and obvious outliers were discarded. Orthogroup #1000 genes encoding TNL-related proteins accounted for 1,927 sequences from the 16 plant genomes used in the orthoMCL analysis, only 1,641 of which had an NB domain suitable for alignment. There were 308 oak genes in

the final set: 174 TNLs, 115 NLs, 16 TNs and 3 Ns. The *Homo sapiens* apoptotic protease-activating factor-1 (APAF-1) sequence, a commonly used outgroup for NB phylogenetic analysis, was added to orthogroup #1000 for tree rooting. A global alignment was obtained with Clustal-Omega in Seaview and conserved sites were selected manually with G-block implemented in Seaview[107]. The maximum likelihood tree was estimated in RAxML 7.7.2, with the standard algorithm, the PROTGAMMAIWAG model of sequence evolution and 1,000 bootstrap replicates[108]. The phylogenetic tree was designed with FigTree v1.4.3 (http://tree.bio.ed.ac.uk). The TNL-containing orthogroup #1000 (**Supplementary Fig. 6**) displayed two major specific expansions in oak that were well supported by bootstrap values. Within these two clades, several small physical clusters containing more than three contiguous genes were identified. These physical clusters were well supported by bootstrap values and consisted of numerous tandem duplicates (see **Supplementary Data Set 5** for details). With 75 genes in total, chromosome 9 was found to have the largest number of TNL clusters distributed along its length. Although based only on 85% of the genes of orthogroup #1000 showing a correct NB domain for alignment, the phylogenetic analysis highlights the obvious expansion of TNLs and related resistance proteins in woody species, shown in brown, relative to other selected plants, shown in green. Other notable large expanded clades corresponded to *E. grandis* and *M. domestica* (**Supplementary Fig. 6**).

3.5.7. **RLK**

Receptor-like kinases (RLKs) constitute one of the largest gene families in plants. The functions of most RLKs are unknown, but the functions described for members of this family include innate immunity, pathogen response, abiotic stress, development, and, in some cases, multiple functions. RLKs usually consist of three domains: an N-terminal extracellular domain, a transmembrane domain, and a C-terminal kinase domain (KD). Leucine-rich

repeat-receptor-like kinases (LRR-RLKs), which contain up to 30 leucine-rich repeat (LRRs) in their extracellular domain, constitute the largest RLK family.

We identified a genome-wide repertoire of oak RLKs containing a KD (PF00069.16), with the hmmsearch program[109]. The KDs of oak RLKs were then aligned with those of RLKs from *Arabidopsis thaliana* (623) and *Oryza sativa* (1,147), using MAFFT[110]. Alignments were cleaned with trimAl (gt 0.2,[111]) and used to build an approximate maximum-likelihood phylogenetic tree (Fastree 2.1.8,[112]). The quality of the alignments was systematically manually checked around all sites on which a positive selection footprint was detected. If the alignment was dubious (less than 4 sequences, presence of numerous gaps, or too divergent sequences), the site was not considered.

With *Arabidopsis* and rice genes as references, this tree was used to classify the oak RLKs into subfamilies, and into 20 subgroups (SG) for LRR-RLKs[113]. We identified 1,247 RLK genes, corresponding to 4.83% of the gene repertoire, *versus* only 2.28% in Arabidopsis and 2.06% in rice (**Supplementary Data Set 6**). Two RLK subfamilies are clearly overrepresented in oak: SD1 (0.88% of the oak gene repertoire, *versus* 0.11% in Arabidopsis and 0.04% in rice) and LRR-RLK (1.69% of the oak gene repertoire, *versus* 0.83% in *Arabidopsis* and 0.67% in rice). A comparison with the LRR-RLK repertoire of 31 other angiosperm species[113] showed that two subgroups, SG-XIIa and SG-XIIb, displayed the highest overall expansion rates relative to the estimated number of genes in the angiosperm last common ancestor (102 copies in oak, expansion rate of 6.8 for SG-XIIa, and 50 copies in oak, expansion rate of 10 for SG-XIIb). As for NBS-LRR genes, a large proportion of LRR-RLK expansions were caused by tandem duplications: 72% and 79% for SG-XIIa and SG-XIIb, respectively. In addition, LRR-RLKs from SG-XIIa, most of which belonged to orthogroup #1006, displayed significant expansion in oak (labeled as #6 in **Fig. 3d**) and in trees more generally (labeled as #5 in **Fig. 4b**), whereas LRR-RLKs from SG-XIIb, mostly

from orthogroup #1003, displayed significant expansion only in trees (labeled as #3 **Fig 4b**). The few known genes in SG-XIIa include *FLS2* (FLAGELLIN-SENSITIVE 2) and *EFR* (EF-TU RECEPTOR) in Arabidopsis, and *Xa21* in rice. The SG-XIIb subgroup includes *XIK1* (Xoo-induced kinase 1). All these receptors are involved in the response to bacterial aggression.

The detection of a positive selection signature provides direct objective evidence of the adaptive role of lineage-specific duplications. We therefore investigated whether, and to what extent, the lineage-specific expanded LRR-RLKs and LRR-RLPs in oak harbored positive selection signatures in oak, as they do in other species[113]. Indeed, the detection of positive signature of selection is a direct and objective evidence of the adaptive role of lineage specific duplications. We used the orthoMCL families built from the same set of 16 species (15 species plus oak). We realigned the proteins from three significantly expanded families of LRR-RLKs (orthoMCL orthogroups #1003, #1006 and #1016 in **Supplementary Data Set 3**), and two of LRR-RLPs (#1009 and #1049 in **Supplementary Data Set 3**) using MAFFT[110] and trimAl (gt 0.2)[111]. Phylogenetic trees were built for each family (PhyML 3.0[114] and groups of oak ultraparalogs (*i.e.* sequences only related by duplication) were identified using a tree reconciliation approach (between the gene trees and species tree), as described by Fischer et al.[113,115]. For each group of ultraparalogs, sequences were aligned to preserve the coding phase (using Prank with the 'codon' option[116] and Guidance for cleaning[117]. We used the EggLib package[118] to infer the maximum likelihood phylogeny at the nucleotide level for every alignment, with PhyML 3.0[114], under the GTR substitution model. We ran the codeml site model implemented in PAML 4 software[119] to infer positive selection on codons under several substitution models (for more details about the models used, see Fisher et al.[113]). The significance of positive selection was assessed in likelihood ratio tests (LRT). The sites at which positive selection was detected were checked manually and we identified the domain to

which they belong, including the specific residue of the LRR when required. In the five gene families identified as displaying significant expansion in oak, 24 groups of oak ultraparalog genes containing up to 28 sequences were identified. Nineteen of these groups had a significant strong signature of positive selection (11 corresponding to LRR-RLKs and 8 to LRR-RLPs, **Supplementary Data Set 9**). The 11 LRR-RLK groups of ultraparalogs belonged to four previously defined subgroups: SG-VIII-2 (1 group), SG-XI (1 group), SG-XIIa (5 groups) and SG-XIIb (4 groups). The two SG-XII subgroups were shown to have undergone species-specific expansion events in a study of 31 angiosperm genomes[113]. Most of the SG-XIIa genes described to date are involved in responses to biotic stresses. After manual curation, 260 sites were confirmed to be targets of positive selection (175 in LRR-RLK, and 85 in LRR-RLP genes). We found that 78% (205) of the 260 sites were located in the LRR domain (150 in LRRs of LRR-RLK genes and 55 in LRR-RLP genes). An investigation of the precise location of the 150 sites within the LRR of LRR-RLK genes revealed that four amino acids in particular (6, 8, 10 and 11), were more frequently targeted by positive selection (121 of the 150 sites, i.e. more than 80%, **Supplementary Fig. 8**). These variable amino acids lie in the unconserved part of the LXXLXLXX β-sheet/β-turn structure typical of LRRs that is involved in protein-protein interactions[120,121]. The residues targeted by positive selection were solvent-exposed[122,123].

3.5.8. Biosynthesis of hydrolysable tannins

Oak tissues have a very high hydrolyzable tannin (HTs) or gallotannin content, and have been one of the chief sources of HTs for leather tanning and dye manufacture for centuries. We studied the oak genome, to find potential clues to the ability of oak to synthesize HTs, which are esters of gallic acid with a polyol (typically β-D-glucose). Gallic acid is a derivative of the shikimate pathway generated by the dehydrogenation of a 5-dehydroshikimate intermediate[124]. The first committed step in HT biosynthesis is the formation of β-glucogallin

(1-O-galloyl-β-D-glucose), which is generated by the esterification of gallic acid and glucose followed by transesterification to generate di-, tri-, tetra-, and pentagalloylglucose **Supplementary Fig. 50**). Ellagitannins and gallotannins are derived from pentagalloylglucose by the addition of further galloyl residues or oxidation[125]. The UDP-glucose:gallic acid glucosyltransferase UGT84A13 has recently been identified as a candidate enzyme in the biosynthesis of β-glucogallin in *Q. robur*[126]. However, the genes and enzymes involved in further esterification steps to generate di-, tri-, tetra-, and pentagalloylglucose remain unknown.

A first set of genes potentially involved in the biosynthesis of HTs was annotated on the basis of sequence similarities to genes involved in the chorismate pathway in *Arabidopsis thaliana*[127]. Uridine diphosphate (UDP) glycosyltransferases (UGTs) mediate the transfer of glycosyl residues from activated nucleotide sugars to acceptor molecules, and a superfamily of over 100 genes encoding UGTs has been identified in *A. thaliana*[128]. Based on the recent characterization of UGT84A13 in *Q. robur*[126], we focused on the members of the neighboring UGT 74, 75, 83 and 84 families[129] (http://www.p450.kvl.dk/At_ugts/family.shtml). We identified 91 genes potentially associated with HT biosynthesis in the oak genome and we performed phylogenetic analyses of the relationships between these genes and their *Arabidopsis* orthologs from the chorismate pathway (**Supplementary Fig. 51a**) and from the UGT 74, 75, 83 and 84 families (**Supplementary Fig. 51b**). We detected significant expension of the UGT 74, 75 and 83 families in the oak genome, and most of the duplications appeared to be in tandem arrays. Thus, tandem duplications seem to have driven the expansion of these UGT families. Conversely, neither the genes of the UGT84 family nor those involved in the chorismate pathway were expanded in the oak genome.

3.5.9. **Laccases**

The so-called "laccases" (EC 1.10.3.2) are a particularly disparate group of multicopper oxidases (MCOs) in plants, also known as laccase-like multicopper oxidases or simply laccase-like proteins[130]. Laccases can oxidize multiple substrates, whereas other enzymes from the MCO family, such as ascorbate oxidases (EC 1.10.3.3), oxidize only specific substrates. Ascorbate oxidases and laccases are structurally related but different MCOs[131]**,** and ascorbate oxidases are often used as an outgroup in phylogenetic analyses of plant laccases.

Little is known about the functions of plant laccases. They can polymerize various phenolic compounds to form insoluble polymers with possible roles in wound healing, plant defense, lignification and the oxidation of seed coat tannins[132]. Three *Arabidopsis* laccases (AtLAC4, 11 and 17) were recently shown to play a role in lignin polymerization, and one (AtLAC15) was implicated in the polymerization of flavonoids in the *Arabidopsis* seed coat[133,134] . These results suggest, at least for lignin polymerization, that laccases are functionally redundant, with multiple mutations required to have a significant effect. In *Arabidopsis* and poplar, the laccases involved in lignification are targets of miR397a[132]**.** This micro-RNA downregulates laccases and transgenic poplars displaying miR397 overexpression have been produced. These trees displayed low levels of expression for 17 laccases and decrease of up to 40% in the laccase activity of the stem xylem[135].

We found 27 laccase genes in the haplome of *Q. robur* and performed a comparative phylogenetic analysis of the laccase protein sequences from *Q. robur, P. trichocarpa*, *E. grandis*, *V. vinifera*, *A. thaliana* and *O. sativa* (**Supplementary Fig. 52**). Sequences were retrieved from Phytozome (https://phytozome.jgi.doe.gov) and the Rice Genome Annotation Project (http://rice.plantbiology.msu.edu/cgi-bin/putative_function_search.pl) by BLAST-p searches. Protein name aliases were used in place of gene model names for *A. thaliana*[133] and *P. trichocarpa* (**Supplementary Table 38**). On the phylogenetic tree, *Q. robur* sequences

were distributed across the seven phylogenetic groups already described in *Arabidopsis*. **Supplementary Table 39** shows the number of laccases within each phylogenetic group for *Arabidopsis*, poplar and pedunculate oak. Pedunculate oak was found to have about twice as many laccase genes as *Arabidopsis*, and about half as many as poplar. Our results therefore suggest that the laccase gene family has undergone expansion in oak, but to a lesser extent than in poplar that however shows a recent whole-genome duplication. Groups #2 and #6 displayed a clear expansion of the laccase gene family in tree species relative to *Arabidopsis*, with group #6 displaying stronger expansion in oak. Group #2 corresponds to laccase homologs of ATLAC4, 11 and 17, essential for lignification in *Arabidopsis*. This biological function is of primary importance for wood cell lignification in trees, so the patterns of duplication and functionalization may differ between trees and herbaceous plants. Group #6 contains seven laccases, including AtLAC14.

3.6. Non-coding RNA prediction and annotation

The prediction of long non-coding RNAs was based on 13 RNAseq libraries (listed in **Supplementary Table 13**). Paired fastq files for the different libraries were aligned with the reference genome fasta file with STAR (version STAR_2.4.0i)[136]. The 13 libraries included 29 million to 72 million sequence pairs and originated from different tissues and conditions: six from buds, four from roots, one from xylem, one from leaf and one from callus tissues. PCR duplicates were pruned from alignment files (SAMtools rmdup, Version: 1.1)[137], which were then merged (SAMtools merge, Version: 1.1) before new transcript and gene calling (Stringtie v1.0.1)[138]. The unique alignment rate for read-pairs exceeded 82 %. The Stringtie model included 158,714 genes and 215,270 transcripts. The resulting GTF file was processed with FEELnc (https://github.com/tderrien/FEELnc, Version 26/05/2015) to remove known genes and transcripts and to calculate the coding potential of the remaining sequences. The predicted lncRNAs were classified with FEELnc_classifier.pl. FEELnc predicted 16,017

genes and 27,147 transcripts not overlapping with the existing haplome gene model and with more than one exon. Using the FEELnc coding potential program, we identified 12,327 long non-coding RNA genes (corresponding to 19,712 transcripts) and 4,312 new protein-coding genes (corresponding to 7,299 transcripts). FEELnc classified one third of the long non-coding RNA candidates as sense and two thirds as antisense, one third as genic and two thirds as intergenic. A track called 'lncRNA' was added to the genome browser with the FEELnc candidate_feelnc_lncRNA.gtf.lncRNA.gtf file.

Other non-coding RNA genes were predicted and annotated with 12 paired RNAseq datasets (listed in **Supplementary Table 14**). In total, 28,001 loci corresponding to ncRNAs were predicted and annotated with tRNAscan-SE[139], RNAmmer[140], cmsearch[141] with RFAM covariance models[142] and sRNA-PlAn (**Supplementary Table 15**). Transfer RNA (tRNA) and ribosomal RNA (rRNA) genes were predicted with tRNAscan-SE and RNAmmer, respectively. The tRNAscan-SE software predicted 827 tRNAs, including 757 tRNAs decoding standard amino acids, 57 pseudogenes, 12 tRNAs of unknown isotypes and one possible suppressor tRNA. RNAmmer software found 82 rRNA loci, including 13 large subunit (LSU) rRNA genes, 20 small subunit (SSU) rRNA genes and 49 rRNA 5S genes. We used the cmsearch program from the Infernal suite with a selection of covariance models relating to families found in eukaryotic genomes, including tRNAs, rRNAs, small nucleolar RNAs (snoRNAs), small nuclear RNAs (snRNAs), miRNAs, SRP RNAs, RnaseMRP RNAs, telomerase RNAs and Vault RNAs. The Infernal cmsearch program found no LSU, but predicted 14 rRNA 5.8S loci, 52 SSU rRNA loci, and 65 rRNA 5S loci. Thus, considering all rRNA predictions, 136 loci in total were predicted and annotated as ribosomal RNA, including 70 rRNA 5S and 61 LSU and/or SSU rRNAs. In total, 44 predicted rRNA 5 S genes were common to the cmsearch and RNAmmer analyses. Seven of the 13 LSU loci predicted by RNAmmer overlapped the 5.8S rRNA predictions calculated by cmsearch. The Infernal

cmsearch program predicted 815 tRNAs. In total, 852 tRNAs were predicted, 790 of which were detected by both tRNAscan-SE and Infernal cmsearch; 25 were specific to Infernal cmsearch and 37 were specific to tRNAscan-SE. Among the other ncRNA genes, a total of 412 C/D box and 74 H/ACA box snoRNA genes were predicted, corresponding to 73 and 17 different families of snoRNA, respectively. With 146 predicted candidates, the C/D box snoRNA71 family was the most heavily represented. An analysis of snoRNA gene organization showed that 190 of these genes were organized into 59 clusters containing two to 11 snoRNA genes. Other snoRNA genes were also identified by eye in the clusters (**Supplementary Fig. 14**, sequence of a snoRNA H/ACA gene conserved in *A. thaliana*). The cmsearch program also predicted 263 pre-miRNA loci, two RNase MRP RNA genes, 31 SRP RNA genes, 225 spliceosomal snRNA genes including 34 U1 snRNA genes, one U11 snRNA gene, 55 U2 snRNA genes, one U12 snRNA gene, 33 U4 snRNA genes, 24 U5 snRNA genes, 64 U6 snRNA genes and 13 U6atac snRNA genes. We retained only one of the pre-miRNA predictions made on both strands at same positions, resulting in the consideration of 204 pre-miRNA loci in the set of pre-miRNA gene predictions. We also predicted miRNA genes with sRNA-PlAn (source code available as a workflow at https://forgemia.inra.fr/genotoul-bioinfo/ngspipelines/tree/master/workflows/srnaseq) on the 12 paired paired small RNAseq datasets. sRNA-PlAn implements a model of miRNA biogenesis. Loci are built by considering the regions of the genome to which reads produced by sRNA-seq experiments map. Candidate loci are subjected to the miRNA prediction procedure, which considers the expected pre-miRNA stem-loop structure, the size of the pre-miRNA sequence, the size of pre-miRNA loops (bulges, internal loops, stem loop), the size of the most represented sequence (20-24 nt), the alignment of this most represented sequence with the stem of the pre-miRNA and the expected expression profile of the pre-miRNA. A score is assigned to each predicted pre-miRNA locus, taking into account the characteristics described above. Each

predicted pre-miRNA locus is then subjected to an annotation procedure in which it is aligned with miRBase[143] and RFAM[142] sequences with BLAST+[144], to differentiate between known ncRNA families and new candidate miRNA families. In total, 26,109 miRNA loci were predicted by sRNA-PlAn, from which 1,508 mature miRNA loci predicted by sRNA-PlAn with a high score were annotated as miRNAs on the basis of strong similarities (one error allowed) to sequences in the miRBase or RFAM databases (fasta files). We found that 145 of the related pre-miRNAs were specific to the RFAM cmsearch program and that 64 of these pre-miRNAs encoded members of the mir-69 gene family. Interestingly, 59 of the pre-miRNA loci predicted by cmsearch contained one or both of the mature miRNAs predicted and annotated with sRNA-PlAn. Different tracks relating to ncRNA predictions/annotations were added to the genome browser, according to the software used.

Finally, the 12 paired small RNAseq datasets as well as ncRNA predictions, lncRNA genes and TEs were used to assign expression evidence to the whole set of predicted noncoding regions. Reads obtained from small RNAseq datasets were mapped onto the genome with Bowtie2[145], using default parameters and retaining only one alignment. Transcription evidence and count data were obtained with Featurecounts for each predicted non-coding RNA locus[146]. Predicted lncRNAs were used to confirm expression at predicted non-coding loci and to identify clusters of shorter ncRNA genes. SAMtools[137] and BEDtools[147] functions were used to manipulate alignments and to identify regions of overlap between the predicted lncRNA and ncRNA genes. We found that 212 of the predicted lncRNA genes overlapped annotated ncRNA loci (strand not considered). Fourteen overlapped 14 rRNA predictions, three overlapped six SRP RNA predictions, with two lncRNAs containing two and three SRP RNA predictions, respectively; 114 overlapped 124 mature miRNA or pre-miRNA predictions, with six lncRNAs overlapping two or more pre-miRNA predictions; 34 overlapped 82 snoRNA, with 19 of lncRNAs overlapping two to 11 snoRNA predictions; 22

overlapped 22 tRNA loci; seven overlapped 10 U6 snRNA predictions, with one lncRNA overlapping four U6 snRNA predictions; one overlapped one U6atac snRNA prediction; three overlapped three U1 snRNA predictions; six overlapped 13 U2 snRNA predictions, with five lncRNAs overlapping two to five U2 snRNA predictions; five overlapped six U4 snRNA predictions, with one lncRNA overlapping two U4 snRNA predictions. The content of small RNAseq datasets was analyzed for non-coding elements, such as predicted lncRNAs, other predicted/annotated ncRNAs and predicted TEs (**Supplementary Table 16**). Bowtie2 aligned 83.34% of the 383,274,162 reads on scaffolds. Using Featurecounts with non-coding elements, such as TEs, lncRNA and ncRNA annotations and predictions, we were able to assign a total of 231,211,802 reads, corresponding to 72.4 % of the mapped reads, to annotated non-coding elements.

4. Mutational landscape

4.1. Estimate of genetic diversity and π0/π4 ratio

The genetic diversity of oak (π) was 0.011 at synonymous sites (π_4), and 0.005 at non-synonymous sites (π_0), with a mean π_0/π_4 ratio of 0.44 (**Supplementary Table 9**). For 1,176 manually curated genes, we recovered the π_0/π4 ratio equals to 0.43 (π_0 = 0.00429 and π_4 = 0.00990). Oak has a higher genetic diversity and π_0/π_4 ratio (**Fig. 2a**) than the other woody perennial species studied by Chen et al.[148]. Further comparisons between "Expanded", "Contracted", and "Unchanged" gene families showed that π_0 estimates were significantly higher for expanded gene families in oak (0.007, p-value<2×10^{-16}) whereas π_4 values were similar for all types of gene families (0.012, **Supplementary Fig. 54**), resulting in a higher π_0/π_4 ratio (0.56). Contracted family genes had a significantly lower π_0/π_4 ratio (0.30) than unchanged families (0.32, p-value=5.2×10^{-3}). TDGs also had a higher π_0/π_4 ratio (0.53), and

an even higher π_0/π_4 was found in the families expanded in oak (0.62). Similar estimates were obtained from the analysis of the pool-seq dataset, i.e. average $\pi_0/\pi 4$ of 0.50 (π_0 = 0.00538 and π_4 = 0.0108), increasing to 0.59 for TDG, and 0.60 for expanded, 0.30 for contracted and 0.32 for unchanged genes. Higher π_0/π_4 values suggest a potential accumulation of deleterious mutations in expanded gene families relative to contracted or unchanged families. We calculated the frequency of mutations (as unnormalized pairwise differences) likely to cause protein malfunction (e.g. premature stop codons, start/stop codon changes). Genes from expanded families displayed significantly more potentially deleterious mutations (mean =0.23, p-value<2×10^{-16}) than those from contracted (0.09) or unchanged families (0.06).

4.2. Detection of somatic mutations

We compared the three libraries L1, L2 and L3 (i.e. 6 pairwise combinations, **Supplementary Table 20**) and detected 61 reliable somatic mutations. A total of 46 somatic mutations were completely absent from the poolseq dataset (40 SNPs), or and had MAFs below 0.5% (6 SNPs), *i.e.* below the minimum threshold used to exclude sequencing errors in the poolseq dataset). Considering our high sequencing depth and the number of individual pooled (20 genotypes), each allele is expected to be near 2.5%. As a consequence, low allele frequency variants are expected to be related to sequencing errors (estimated at 2.4%, see **Supplementary Figure 25**). Thus, to be conservative, we filtered out all candidate somatic mutations with an allele frequency above 0.005. As a result, 75% of the somatic mutations (46/61) could be considered to be detected exclusively in the "3P" accession (**Supplementary Table 5**).

Noteworthy, one of the 40 somatic mutations detected exclusively in "3P" was found in a gene coding sequence: Sc0000066_1207928 in Qrob_T0204900.2 corresponded to a member of the large cytochrome P450 superfamily encoding a protein of the CYP4/CYP19/CYP26

subfamilies with annotations relating to secondary metabolite biosynthesis, transport and catabolism, lipid transport and metabolism. This mutation was synonymous (ACC->ACT, corresponding to a threonine residue in the protein).

5. Comparative and evolutionary genomics

5.1. Macroevolutionary analysis

5.1.1. Oak karyotype evolution and genome organization

Considering grape to be the closest modern representative of the n=21 rosid ancestor (derived from a post-γ ancestor with 7 protochromosomes (shown in color on the *y*-axis of the dotplots of **Supplementary Fig. 16**) the comparisons between grape-eucalyptus and grape-watermelon shows a clear 1:2 relationships, while that between grape-coco, grape-peach and grape-oak genomes shows a clear a 1:1 relationships see dotplot diagonals in each chart, shown with green circles in **Supplementary Fig. 16**.

5.1.2. Gene family expansion/contraction in oak

For the total of 541,339 gene models across the 15 species (**Supplementary Table 21**, **Supplementary Fig. 17**) plus *Q. robur*, 435,095 were classified into 36,844 orthogroups (gene families) (**Supplementary Data Set 3 sheet #1**), with 106,444 genes remaining singletons after clustering (**Supplementary Table 7**). In total, 4860 orthogroups were common to all species. For the 25,808 oak proteins, 22,498 clustered into 11,813 orthogroups, 479 of which were oak-specific and contained 1,737 oak proteins. There were also 3,310 singleton proteins for oak. From the 36,844 orthogroups 524 and 72 were found to be expanded and contracted in oak, respectively (**Supplementary Data Set 3 sheet #2 and #4**). A total of 154 orthogroups were specific to oak (**Supplementary Fig. 18**), whereas 65 were common to all species (**Supplementary Fig. 18**). We found that 73% of the genes within

expanded orthogroups were tandemly duplicated genes (TDGs), this percentage increasing to 99% if we also included long distance-duplicated genes (LDGs), whereas the 72 contracted orthogroups contained only 47% TDGs *(***Supplementary Fig. 19**).

5.2. Identification of tandemly duplicated genes in oak

Speciation and duplication events in the pedunculate oak genome were identified using the K_s distribution of orthologous gene pairs between oak and peach (green bars in **Supplementary Fig. 20**) and paralogs in oak (purple bars in **Supplementary Fig. 20**), respectively. The oak/peach ortholog K_s distribution defines the position of the speciation event between these two species, with a single ancestral triplication event (γ) common to grape, peach, cocoa and oak and predating the speciation event. The burst of tandem duplicates highlighted by the purple K_s peak occurred after oak/peach speciation and appears to be an oak-specific event. The dot plot representation of tandemly duplicated genes (TDGs) in oak is depicted in **Supplementary Fig. 21**. We identified 9,189 TDG (**Supplementary Data Set 4**) using the threshold and methodology presented in the method section. They were validated based on (i) the comparison with polymorphism of allelic gene pairs (**Supplementary Fig. 22**) and (ii) sequence coverage analysis (**Supplementary Fig. 23**). Besides, we identified 8,797 genes as long distance duplicated genes (LDGs) and 7,822 genes as single genes (SGs) (**Supplementary Data Set 4**).

5.3. Challenges in the identification of genes related to tree habit

The increasingly rapid rate at which full genome sequences are being published opens up exciting possibilities, but the 16 species for which suitable genome sequences were available for this study represents only a small proportion of the worldwide diversity of plants (there are currently ~350,000 accepted angiosperm species (http://www.theplantlist.org/). It was recently estimated that almost 50% of vascular plants, most of which are angiosperms, are

woody[149]. There are probably, therefore, many genomic changes associated with shifts in growth form not captured in our analyses. Given the modest number of genomes included in this study, it remains unclear whether the patterns highlighted here can be generalized to larger numbers of species and clades. It is also unclear whether the same sets of expanding and contracting gene families would be identified in all evolutionary transitions from herbaceous to woody forms. Fortunately, even with the limited number of genomes available, it is possible to identify the branch points within the phylogeny at which additional targeted sequencing would help to provide an answer to this question. The most dynamic aspects of growth form shifts within the angiosperm phylogeny have occurred within the eudicots, a group consisting largely of rosids and asterids[149–152]. The sequencing of genomes for additional tree species in this part of the phylogeny would be particularly informative. Fitzjohn et al.[149] used the distribution of growth forms from Zanne et al.[152] to estimate the proportion of woody taxa across vascular plants at the genus, family and order levels. The clades highlighted by Fitzjohn et al.[149] as both variable in growth form (defined here as clades with 30-70% of species considered to be woody according to the strong prior) and diverse (defined here as containing ≥10 species), comprise 470 genera, 41 families and 12 orders. Paired comparisons of close relatives in these clades, ideally genera with both woody and herbaceous members, would make it possible to determine whether gene expansions in R-gene families are correlated with evolutionary shifts in growth form. It is clear that certain clades are extraordinarily variable in terms of growth habit, but it seems unlikely that growth habit *per se* drives expansions and contractions in R-gene families. Instead, with their longer lifespans, woody species probably accumulate a greater pathogen load than herbaceous taxa. It would therefore appear reasonable to consider longevity as a driver of these functional gene shifts, and growth habit as a correlate of such differences in life history.

We examined the full genome sequences currently or soon to be available for eudicots (as reported in (https://genomevolution.org/wiki/index.php/Sequenced_plant_genomes) as of 18 December 2016), to identify future targets building on existing genomic resources. We had access to genome sequences for 46 herbaceous and 27 woody species from 18 orders (**Supplementary Table 40 and Supplementary Fig. 55**), 14 of which display growth habit variation (defined here as clades with 30-70% of woody species according to the strong prior) and sufficient diversity (defined here as ≥10 species), accordin to Fitzjohn et al.[149]. The sequencing of herbaceous species from four variable genera (*Nicotiana, Linum, Genlisea, Solanum*) is underway, but full genome sequences for both a woody and a herbaceous species from the same genus have yet to be published. In Fabaceae, complete genome sequences have been released for herbaceous species from eight genera and for one woody species from the genus *Cajanus*. Rosaceae also includes four woody and one herbaceous species (*Fragaria*) for which complete genome sequences have been released. Three variable orders have fully sequenced herbaceous species (Ranunculales, Caryophyllales, Solanales). In addition, Lamiales has three herbaceous and one woody (*Fraxinus*) species for which full genome sequences have been obtained, and Fabales has eight herbaceous and one woody (*Cajanus*) species with full genome sequences. Additional genome sequences for species from any of these clades (ideally within genera), considered together with the oak genome sequence, would improve our understanding of the evolution of genomic features favoring a long lifespan and woodiness in plants.

5.4. Gene ontology (GO) enrichment analysis

5.4.1. GO term enrichment in three categories of genes

We assigned a total of 3,433 GO terms (**Supplementary Table 41**): 1,179 for molecular function (MF), 1,867 for biological process (BP) and 387 for cellular component (CC). At

least one GO term was assigned to 16,820 of the 25,808 oak gene models (65.2%). The mean number of GO terms per gene was 3.73 (ranging from 1 to 18) (**Supplementary Fig. 56**) and gene counts per GO term are provided in **Supplementary Fig. 57**. We found that 332 GO terms were associated with only one gene.

GO term enrichment was compared between three categories of genes: (i) TDGs (i.e. genes located in close proximity, see section 4.2 for the method), (ii) LDGs and (iii) SGs. The number of significant GO terms (*p*-value < 0.05) was 97 for TDGs, 144 for LDGs and 240 for SGs (**Supplementary Table 41**).

For TDGs (**Supplementary Data Set 8 sheet #1**), the best supported GO terms (in terms of *p*-value and fold-enrichment) highlighted gene products involved in ‘protein phosphorylation’ (GO:0006468, $P<1\times\times10^{-30}$), ‘signal transduction’ (GO:0007165, $P<1\times10^{-30}$), ‘recognition of pollen’ (GO:0048544, $P<1\times10^{-30}$), ‘oxidation-reduction process’ (GO:0055114, $P=7.2\times10^{-29}$), ‘metabolic process’ (GO:0008152, $P=2.2\times10^{-17}$), ‘chitin catabolic process’ (GO:0006032, $P=1.9\times10^{-13}$), ‘response to biotic stimulus’ (GO:0009607, $P=4\times10^{-12}$), ‘cell wall macromolecule catabolic process’ (GO:0016998, $P=2.3\times10^{-10}$), ‘response to oxidative stress’ (GO:0006979, 10^{-8}), ‘drug transmembrane transport’ (GO:0006855, $P=1.9\times10^{-7}$) and ‘defense response’ (GO:0006952, $P=9\times10^{-7}$). Thus, gene products executing activities related to ‘ADP binding’ (GO:0043531, $P<1\times10^{-30}$), ‘transferase activity’ (GO:0016758, $P=3.8\times10^{-28}$), ‘heme binding’ (GO:0020037, $P=1.3\times10^{-27}$), ‘protein kinase activity’ (GO:0004672, $P=1.2\times10^{-26}$), ‘oxidoreductase activity’ (GO:0016705, $P=5.8\times10^{-23}$), iron ion binding (‘GO:0005506’, $P=1.1\times10^{-17}$), ‘chitinase activity’ (GO:0004568, $P=2.1\times10^{-13}$), ‘protein serine/threonine kinase activity’ (GO:0004674, $P=2.4\times10^{-13}$), and ‘nutrient reservoir activity’ (GO:0045735, $P=1.3\times10^{-10}$) were the most frequently detected. Membrane-bound (LRR-RLKs and LRR-RLPs) and cytosolic (NB LRR) receptors, together with UDP-glycosyltransferase,

cytochrome P450, chitinase, peroxidase, and psathogenesis-related protein were among the most frequently detected proteins corresponding to the BP and MF ontologies.

By contrast, a very different molecular signature was obtained for LDGs (**Supplementary Data Set 8 sheet #2**). The best supported GO terms included ‘regulation of transcription, DNA-templated’ (GO:0006355, P=1.20×10^{-10}), ‘protein dephosphorylation’ (GO:0006470, P=3.50×10^{-9}), ‘small GTPase-mediated signal transduction’ (GO:0007264, P=1.30×10^{-8}), ‘microtubule-based process’ (GO:0007017, P=2.60×10^{-8}), ‘translation’ (GO:0006412, P=1.30×10^{-7}), ‘response to heat’ (GO:0009408, P=3.50×10^{-7}), ‘protein folding’ (GO:0006457, P=1.70×10^{-6}), ‘fatty acid biosynthetic process’ (GO:0006633, P=2.20×10^{-6} ‘biosynthetic process’ (GO:0009058, P=3.60×10^{-5} and ‘protein polymerization’ (GO:0051258, P=6.90×10^{-5}). Thus, gene products with activities relating to ‘protein serine/threonine phosphatase activity’ (GO:0004722, P=3.70×10^{-13}), ‘DNA binding’ (GO:0003677, P=1.70×10^{-12} ‘sequence-specific DNA binding’ GO:0043565, P=1.90×10^{-11}), ‘GTP binding’ (GO:0005525, P=9.30×10^{-11}), ‘structural constituent of ribosome’ (GO:0003735, P=2.60×10^{-9}), ‘transcription factor activity, sequence-specific DNA binding’ (GO:0003700, P=5.90×10^{-9}), ‘NADH dehydrogenase (ubiquinone) activity’ (GO:0008137, P=1.30×10^{-8}), ‘GTPase activity’ (GO:0003924, P=2.40×10^{-8}), ‘structural constituent of cytoskeleton’ (GO:0005200, P=8.40×10^{-7}), and ‘microtubule binding’ (GO:0008017, P=2.80×10^{-6}) were the most frequently detected. Protein phosphatases, proteins with DNA-binding and homeobox domains, transcription factors, elongation factors, ribosomal proteins, microtubule-associated proteins, and DNA gyrases were among the most widespread proteins corresponding to the BP and MF ontologies.

For SGs (**Supplementary Data Set 8 sheet #3**), the best supported GO terms concerned ‘DNA replication’ (GO:0006260, P=5×10^{-13}), ‘transcription, DNA-templated’ (GO:0006351, P=8.6×10^{-13}), 'DNA repair’ (GO:0006281, P=1.1×10^{-11}), 'RNA processing’ (GO:0006396,

$P=1.2\times10^{-9}$), 'photosynthesis’ (GO:0015979, $P=1.2\times10^{-9}$), ‘pseudouridine synthesis’ (GO:0001522, $P=1.5\times10^{-9}$), ‘DNA recombination’ (GO:0006310, $P=3.9\times10^{-9}$), 'protein ubiquitination’ (GO:0016567, $P=9.8\times10^{-7}$), ‘glycerol ether metabolic process’ (GO:0006662, $P=1.3\times10^{-6}$) and 'translation’ (GO:0006412, $P=3.4\times10^{-6}$). Thus, gene products with activities relating to ‘binding’ (GO:0005488, $P=4.7\times10^{-24}$), ‘RNA binding’ (GO:0003723, $P=6.2\times10^{-20}$), ‘nucleic acid binding’ (GO:0003676, $P=2.4\times10^{-19}$), ‘zinc ion binding’ (GO:0008270, $P=6.9\times10^{-16}$), ‘metal ion binding’ (GO:0046872, $P=4.8\times10^{-10}$), ‘pseudouridine synthase activity’ (GO:0009982, $P=1.5\times10^{-8}$), 'DNA-directed RNA polymerase activity’ (GO:0003899, $P=1.9\times10^{-8}$), ‘nucleotide binding’ (GO:0000166, $P=4.1\times10^{-8}$), ‘ubiquitin-protein transferase activity’ (GO:0004842, $P=8\times10^{-8}$), ‘threonine-type endopeptidase activity’ (GO:0004298, $P=1.1\times10^{-5}$), 'DNA helicase activity’ (GO:0003678, $P=4.5\times10^{-5}$) and ‘DNA binding’ (GO:0003677, $P=9.5\times10^{-5}$) were the most frequently detected. Zinc finger and DNA repair proteins, as well as DEAD/DEAH box helicase, RNA pseudouridylate synthase and RNA polymerase were among the most frequently detected proteins corresponding to the BP and MF ontologies.

5.4.2. GO term enrichment in orthogroups expanded in pedunculate oak

The 524 orthogroups expanded in oak comprise 5,910 genes (3 to 359 genes per orthogroup, with a mean of 11.3 genes per orthogroup, **Supplementary Fig. 58**). In total, 366 orthogroups were annotated with at least one GO term. The number of GO terms per gene family ranged from 1 to 17 (mean value, 2.89). We found that 4,217 of the 5,910 genes (71.4%) were annotated with at least one GO term (**Supplementary Table 42**). The annotation used 3,433 unique GO terms, including 1,722 singletons (GO terms used only once) (**Supplementary Fig. 59**). We identified 58 significantly enriched GO terms (33 MF, 17 BP and 8 CC) (**Supplementary Data Set 8 sheet #4**) in orthogroups displaying expansion in oaks. We compared sample counts (numbers of genes annotated with particular GO terms among the

genes belonging to the orthogroups expanded in oak) with genome counts (number of genes annotated with particular GO terms among the 25,808 oak gene models; **Supplementary Fig. 60**). The enriched term with the best statistical support was ‘protein kinase activity’ (GO:0004672, $P<10^{-30}$), which was attributed to 726 genes (in the 524 expanded orthogroups) of the 1,556 genes found in the 25,808 oak gene models, corresponding to two-fold enrichment. These 726 genes belonged to 31 orthogroups containing genes encoding both cytosolic (NB-LRRs) and membrane (LRR-RLKs, LRR-RLPs) receptors of the innate immune system (i.e. R-genes). This overrepresentation of R-genes was also supported by the enrichment of the orthogroups in the following annotations: ‘protein serine/threonine kinase activity’ (GO:0004674), ‘protein binding’ (GO:0005515), ‘polysaccharide binding’ (GO:0030247), ‘ADP binding’ (GO:0043531), ‘protein phosphorylation’ (GO:0006468), ‘signal transduction’ (GO:0007165), ‘recognition of pollen’ (GO:0048544), all with $P<10^{-30}$ and fold-enrichments of 1.7 to 3.9 (for ‘ADP binding’). The highest fold-enrichment (about 4.4) was observed for the MF ‘thioredoxin-disulfide reductase activity’ (GO:0004791, $P=8.4\times10^{-5}$) and the BP ‘removal of superoxide radicals’ (GO:0019430, $P=2.9\times10^{-5}$), with seven genes annotated as pyridine nucleotide-disulfide oxidoreductases.

5.4.3. GO enrichment within the gene families expanded in woody perennial trees relative to herbaceous species

Overall, 18,855 of the 36,844 othoMCL orthogroups (**Supplementary Data Set 3 sheet #1**) (51.2%) were annotated with at least one GO term, with 16,703 orthogroups annotated for molecular function (MF), 11,495 for biological process (BP) and 5,073 for cellular component (CC). In total, 3,936 unique GO terms were used in the annotation. Of the 126 orthogroups expanded in “trees” (**Supplementary Data Set 7 sheet #2**), 108 were annotated with GO terms used in the GO term enrichment analysis. We detected significant enrichment for 61

GO terms (38 MFs, 19 BPs and 4 CCs, **Supplementary Table 43 and Supplementary Data Set 8 sheet #5**).

The functions of the set of gene families expanded in woody species were identified against the background of all orthogroups. The degree of orthogroup size expansion for statistically significant GO terms, represented by fold-enrichment in woody perennials is depicted in **Supplementary Fig. 7**. The term with most statistical support was ‘apoptotic process’ (GO:0006915, $P=7.4\times10^{-14}$). It was found in 10 of the 126 expanded orthogroups, but only 37 of the total number of 36,844 orthogroups, giving a fold-enrichment of 79 (**Supplementary Fig. 7**). These 10 clusters included R-genes with a characteristic NB-ARC domain. ‘ATP binding’ (GO:0005524, $P=2.8\times10^{-10}$), ‘ADP binding’ (GO:0043531, $P=10^{-9}$), ‘protein serine/threonine kinase activity’ (GO:0004674, $P=6.4\times10^{-7}$), ‘protein tyrosine kinase activity’ (GO:0004713, $P=1.4\times10^{-6}$), ‘protein phosphorylation’ (GO:0006468, $P=1.8\times10^{-6}$) ‘DNA integration’ (GO:0015074, $P=3.2\times10^{-6}$), ‘polysaccharide binding’ (GO:0030247, $P=1.7\times10^{-5}$), transmembrane signaling receptor activity’ (GO:0004888, $P=9.6\times10^{-5}$), ‘innate immune response’ (GO:0045087, $P=1.4\times10^{-4}$) and ‘recognition of pollen’ (GO:0048544, $P=2\times10^{-4}$) ranked among the next most significant GO terms, with fold-enrichments ranging from 7 up to 83.5 for ‘protein serine/threonine kinase activity’. The orthogroups concerned included almost exclusively cytosolic and membrane receptors of the innate immune system (**Supplementary Data Set 8 sheet #5**). For instance, the 10 most frequent orthogroups (orthogroups #1000, 1004, 1021, 1084, 1006, 1010, 1016, 1017, 1037, 1003) cited 115 times in a total of 367 occurrences, i.e. over 30%, corresponded to the two major types of plant receptors: leucine-rich repeat-receptor-like kinase/receptor-like proteins (LRR-RLKs, LRR-RLPs) and nucleotide-binding leucine-rich repeat proteins (NB-LRRs).

6. Web resources

Genome data	Web access
Oak genome assembly PM1N (haploid version: 12 pseudomolecules + 538 unassigned scaffolds)	Download: https://urgi.versailles.inra.fr/download/oak/Qrob_PM1N.fa.gz<br>Blast: https://urgi.versailles.inra.fr/blast<br>Pseudomolecule: https://urgi.versailles.inra.fr/WebApollo_oak_PM1N/PseudoMolecule.html<br>JBrowse: https://urgi.versailles.inra.fr/WebApollo_oak_PM1N/jbrowse<br>Intermine: https://urgi.versailles.inra.fr/OakMine_PM1N/begin.do
Oak genome assembly V2_2N (diploid version 2)	Download: https://urgi.versailles.inra.fr/download/oak/Qrob_V2_2N.fa.gz<br>Blast: https://urgi.versailles.inra.fr/blast<br>JBrowse: https://urgi.versailles.inra.fr/WebApollo_oak_V2/jbrowse/
Oak genome assembly V1_2N (diploid version 1, [19])	Download: https://urgi.versailles.inra.fr/download/oak/Qrob_V1_2N.fa.gz<br>Blast: https://urgi.versailles.inra.fr/blast
Oak transcriptome (*de novo* assembly, [23])	Download: https://urgi.versailles.inra.fr/download/oak/OCV4_assembly_final.fsa.gz<br>Blast: https://urgi.versailles.inra.fr/blast
Oak protein-coding sequences predicted on PM1N (haploid version)	Download CDS (aa) https://urgi.versailles.inra.fr/download/oak/Qrob_PM1N_CDS_aa_20161004.fa.gz<br>Download CDS (nt) https://urgi.versailles.inra.fr/download/oak/Qrob_PM1N_CDS_nt_20161004.fa.gz<br>Blast: https://urgi.versailles.inra.fr/blast

7. Data availability

The oak haploid genome assembly and corresponding annotation have been deposited in the European Nucleotide Archive under project accession code PRJEB19898. Other sequence release data are indicated in **Supplementary tables 1, 13, 14 and 19 and Supplementary Data Set 10.** We also invite readers to download data stored at the URLs indicated in section 6 (Web resources) as well as in the oakgenome web site: http://www.oakgenome.fr.

8. References

1. Manos, P. S. & Stanford, A. M. The historical biogeography of Fagaceae: tracking the tertiary history of temperate and subtropical forests of the Northern Hemisphere. *Int. J. Plant Sci.* **162,** S77--S93 (2001).
2. Hubert, F. et al. Multiple nuclear genes stabilize the phylogenetic backbone of the

genus Quercus. *Syst. Biodivers.* **12,** 405–423 (2014).

3. Johnson, P. S. in *Silvics of North America 2, Hardwoods* (ed. Burns, R. M., Honkala, B. H.) 686–692 (U.S. Department of Agriculture, For. Serv., 1990).
4. Menitskii, I. L. & Fedorov, A. A. *Oaks of Asia*. (Science Publishers, 2005).
5. Oldfield, S. & Eastwood, A. The red list of oaks. (2007).
6. Cavender-Bares, J. Diversity, distribution, and ecosystem services of the North American oaks. *Int. Oaks* **27,** 37–48 (2016).
7. Antolin, F. & Jacomet, S. Wild fruit use among early farmers in the Neolithic (5400-2300 cal bc) in the north-east of the Iberian Peninsula: an intensive practice? *Veg. Hist. Archaeobot.* **24,** 19–33 (2015).
8. Logan, W. B. *Oak: the frame of civilization*. (WW Norton & Company, 2005).
9. Eaton, E., Caudullo, G., Oliveira, S. & de Rigo, D. *Quercus robur* and *Quercus petraea* in Europe: distribution, habitat, usage and threats. *Quercus robur Quercus petraea Eur. Distrib. habitat, usage Threat.* 160–163 (2016).
10. Vinha, A. F., Barreira, J. C. M., Costa, A. S. G. & Oliveira, M. B. P. P. A new age for *Quercus* spp. fruits: review on nutritional and phytochemical composition and related biological activities of acorns. *Compr. Rev. Food Sci. Food Saf.* **15,** 947–981 (2016).
11. Büntgen, U. et al. 2500 years of European climate variability and human susceptibility. *Science (80-.).* **331,** 578–582 (2011).
12. Haneca, K., Čufar, K. & Beeckman, H. Oaks, tree-rings and wooden cultural heritage: a review of the main characteristics and applications of oak dendrochronology in Europe. *J. Archaeol. Sci.* **36,** 1–11 (2009).
13. Cufar, K. et al. Common climatic signals affecting oak tree-ring growth in SE Central Europe. *Trees* **28,** 1267–1277 (2014).
14. Rani, J., Chauhan, P. & Tripathi, R. Li-Fi (Light Fidelity)-the future technology in

wireless communication. *Int. J. Appl. Eng. Res.* **7,** 1517–1520 (2012).

15. Faivre Rampant, P. et al. Analysis of BAC end sequences in oak, a keystone forest tree species, providing insight into the composition of its genome. *BMC Genomics* **12,** 292 (2011).
16. Chalhoub, B., Belcram, H. & Caboche, M. Efficient cloning of plant genomes into bacterial artificial chromosome (BAC) libraries with larger and more uniform insert size. *Plant Biotechnol. J.* **2,** 181–188 (2004).
17. Bodénès, C. et al. Comparative mapping in the Fagaceae and beyond with EST-SSRs. *BMC Plant Biol.* **12,** (2012).
18. Bao, W., Kojima, K. K. & Kohany, O. Repbase Update, a database of repetitive elements in eukaryotic genomes. *Mob. DNA* **6,** 11 (2015).
19. Plomion, C. et al. Decoding the oak genome: public release of sequence data, assembly, annotation and publication strategies. *Mol. Ecol. Resour.* **16,** 254–265 (2016).
20. Sallet, E., Gouzy, J. & Schiex, T. EuGene-PP: a next-generation automated annotation pipeline for prokaryotic genomes. *Bioinformatics* **30,** 2659–2661 (2014).
21. Quevillon, E. et al. InterProScan: protein domains identifier. *Nucleic Acids Res.* **33,** W116-20 (2005).
22. Hebsgaard, S. M. et al. Splice site prediction in *Arabidopsis thaliana* pre-mRNA by combining local and global sequence information. *Nucleic Acids Res.* **24,** 3439–52 (1996).
23. Lesur, I. et al. The oak gene expression atlas: insights into Fagaceae genome evolution and the discovery of genes regulated during bud dormancy release. *BMC Genomics* **16,** 112 (2015).
24. Salamov, A. A. & Solovyev, V. V. Ab initio gene finding in *Drosophila* genomic

DNA. *Genome Res.* **10,** 516–522 (2000).

25. Stanke, M. & Morgenstern, B. AUGUSTUS: a web server for gene prediction in eukaryotes that allows user-defined constraints. *Nucleic Acids Res.* **33,** 465–467 (2005).
26. Noé, L. & Kucherov, G. YASS: enhancing the sensitivity of DNA similarity search. *Nucleic Acids Res.* **33,** 540–543 (2005).
27. Delcher, A. L., Phillippy, A., Carlton, J. & Salzberg, S. L. Fast algorithms for large-scale genome alignment and comparison. *Nucleic Acids Res.* **30,** 2478–2483 (2002).
28. Sullivan, M. J., Petty, N. K. & Beatson, S. A. Easyfig: a genome comparison visualizer. *Bioinformatics* **27,** 1009–1010 (2011).
29. Kent, W. J. BLAT — The BLAST -Like Alignment Tool. *Genome Res.* **12,** 656–664 (2002).
30. Kurtz, S. et al. Versatile and open software for comparing large genomes. *Genome Biol.* **5,** R12 (2004).
31. Endelman, J. B. & Plomion, C. LPmerge: an R package for merging genetic maps by linear programming. *Bioinformatics* **30,** 1623–1624 (2014).
32. Bodénès, C., Chancerel, E., Ehrenmann, F., Kremer, A. & Plomion, C. High-density linkage mapping and distribution of segregation distortion regions in the oak genome. *DNA Res.* **23,** 115–124 (2016).
33. Pont, C. et al. Wheat syntenome unveils new evidences of contrasted evolutionary plasticity between paleo- and neoduplicated subgenomes. *Plant J.* **76,** 1030–1044 (2013).
34. Katzourakis, A. Paleovirology: inferring viral evolution from host genome sequence data. *Philos. Trans. R. Soc. Lond. B. Biol. Sci.* **368,** 20120493 (2013).
35. Geering, A. D. W. Caulimoviridae (Plant Pararetroviruses). *eLS* (2007).

36. Jakowitsch, J., Mette, M. F., van Der Winden, J., Matzke, M. a & Matzke, a J. Integrated pararetroviral sequences define a unique class of dispersed repetitive DNA in plants. *Proc. Natl. Acad. Sci. U. S. A.* **96,** 13241–13246 (1999).

37. Geering, A. D. W. et al. Endogenous florendoviruses are major components of plant genomes and hallmarks of virus evolution. *Nat. Commun.* **5,** 5269 (2014).

38. Hu, T. T. et al. The *Arabidopsis lyrata* genome sequence and the basis of rapid genome size change. *Nat. Genet.* **43,** 476–81 (2011).

39. Verde, I. et al. The high-quality draft genome of peach (*Prunus persica*) identifies unique patterns of genetic diversity, domestication and genome evolution. *Nat. Genet.* **45,** 487–94 (2013).

40. Velasco, R. et al. The genome of the domesticated apple (*Malus* × *domestica* Borkh.). *Nat. Genet.* **42,** 833–839 (2010).

41. Myburg, A. A. et al. The genome of *Eucalyptus grandis*. *Nature* **510,** 356–+ (2014).

42. Ming, R. et al. The draft genome of the transgenic tropical fruit tree papaya (*Carica papaya* Linnaeus). *Nature* **452,** 991–996 (2008).

43. Wu, G. A. et al. Sequencing of diverse mandarin, pummelo and orange genomes reveals complex history of admixture during citrus domestication. *Nat. Biotechnol.* **32,** 656–662 (2014).

44. Flutre, T., Duprat, E., Feuillet, C. & Quesneville, H. Considering transposable element diversification in de novo annotation approaches. *PLoS One* **6,** (2011).

45. Maumus, F. & Quesneville, H. Ancestral repeats have shaped epigenome and genome composition for millions of years in *Arabidopsis thaliana. Nat Commun* **5,** 4104 (2014).

46. Ellinghaus, D., Kurtz, S. & Willhoeft, U. LTRharvest, an efficient and flexible software for de novo detection of LTR retrotransposons. *BMC Bioinformatics* **9,** 18

(2008).

47. Baidouri, M. El et al. Widespread and frequent horizontal transfers of transposable elements in plants. *Genome Res.* **24,** 831–838 (2014).

48. Ma, J. & Bennetzen, J. L. Rapid recent growth and divergence of rice nuclear genomes. *Proc. Natl. Acad. Sci. U. S. A.* **101,** 12404–10 (2004).

49. Baidouri, M. El & Panaud, O. Comparative genomic paleontology across plant kingdom reveals the dynamics of TE-driven genome evolution. *Genome Biol. Evol.* **5,** 954–965 (2013).

50. Baucom, R. S. et al. Exceptional diversity, non-random distribution, and rapid evolution of retroelements in the B73 maize genome. *PLoS Genet.* **5,** e1000732 (2009).

51. Peterson-Burch, B. D., Nettleton, D. & Voytas, D. F. Genomic neighborhoods for Arabidopsis retrotransposons: a role for targeted integration in the distribution of the Metaviridae. *Genome Biol.* **5,** R78 (2004).

52. Zytnicki, M. & Quesneville, H. S-MART, a software toolbox to aid RNA-Seq data analysis. *PLoS One* **6,** e25988 (2011).

53. Jiang, S.-Y. et al. Sucrose metabolism gene families and their biological functions. *Sci. Rep.* **5,** 17583 (2015).

54. Panchy, N., Lehti-Shiu, M. D. & Shiu, S.-H. Evolution of gene duplication in plants. *Plant Physiol.* **171,** 2294–2316 (2016).

55. Jung, J. S., Prestont, G. M., Smith, B. L., Guggino, W. B. & Agre, P. Molecular structure of the water channel through aquaporin CHIP: the hourglass model. *J. Biol. Chem.* **269,** 14648–14654 (1994).

56. Murata, K. et al. Structural determinants of water permeation through aquaporin-1. *Nature* **407,** 599–605 (2000).

57. Froger, A., Tallur, B., Thomas, D. & Delamarche, C. Prediction of functional residues

in water channels and related proteins. *Protein Sci.* **7,** 1458–1468 (1998).

58. Johanson, U. et al. The complete set of genes encoding major intrinsic proteins in Arabidopsis provides a framework for a new nomenclature for major intrinsic proteins in plants. **126,** 1358–1369 (2016).

59. Chaumont, F., Barrieu, F., Wojcik, E., Chrispeels, M. J. & Jung, R. Aquaporins constitute a large and highly divergent protein family in maize. *Plant Physiol.* **125,** 1206–1215 (2001).

60. Cohen, D. et al. Developmental and environmental regulation of aquaporin gene expression across populus species: divergence or redundancy? *PLoS One* **8,** (2013).

61. Gupta, A. & Sankararamakrishnan, R. Genome-wide analysis of major intrinsic proteins in the tree plant *Populus trichocarpa*: characterization of XIP subfamily of aquaporins from evolutionary perspective. *BMC Plant Biol.* **9,** 134 (2009).

62. Dubos, C. et al. MYB transcription factors in Arabidopsis. *Trends Plant Sci.* **15,** 573–581 (2010).

63. Soler, M. et al. The Eucalyptus grandis R2R3-MYB transcription factor family: evidence for woody growth-related evolution and function. *New Phytol.* **206,** 1364–1377 (2015).

66. Gonzalez, A., Mendenhall, J., Huo, Y. & Lloyd, A. TTG1 complex MYBs, MYB5 and TT2, control outer seed coat differentiation. *Dev. Biol.* **325,** 412–421 (2009).

67. Cavallini, E. et al. The phenylpropanoid pathway is controlled at different branches by a set of R2R3-MYB C2 repressors in grapevine. *Plant Physiol.* **167,** 1448–70 (2015).

68. Yoshida, K., Ma, D. & Constabel, C. P. The MYB182 protein down-regulates proanthocyanidin and anthocyanin biosynthesis in poplar by repressing both structural and regulatory flavonoid genes. *Plant Physiol.* **167,** 693–710 (2015).

69. Soler, M. et al. The woody-preferential gene EgMYB88 regulates the biosynthesis of

phenylpropanoid-derived compounds in wood. *Front. Plant Sci.* **7,** 1422 (2016).

70. Hanada, K., Zou, C., Lehti-Shiu, M. D., Shinozaki, K. & Shiu, S.-H. Importance of lineage-specific expansion of plant tandem duplicates in the adaptive response to environmental stimuli. *Plant Physiol.* **148,** 993–1003 (2008).

71. Chen, L.-Q. et al. Sugar transporters for intercellular exchange and nutrition of pathogens. *Nature* **468,** 527–32 (2010).

72. Chen, L.-Q. et al. A cascade of sequentially expressed sucrose transporters in the seed coat and endosperm provides nutrition for the Arabidopsis embryo. *Plant Cell* **27,** 607–19 (2015).

73. Tarkka, M. T. et al. OakContigDF159.1, a reference library for studying differential gene expression in *Quercus robur* during controlled biotic interactions: use for quantitative transcriptomic profiling of oak roots in ectomycorrhizal symbiosis. *New Phytol.* **199,** 529–540 (2013).

74. Herrmann, S. et al. Endogenous rhythmic growth, a trait suitable for the study of interplays between multitrophic interactions and tree development. *Perspect. Plant Ecol. Evol. Syst.* **19,** 40–48 (2016).

75. Herrmann, S., Munch, J. C. & Buscot, F. A gnotobiotic culture system with oak microcuttings to study specific effects of mycobionts on plant morphology before, and in the early phase of, ectomycorrhiza formation *Paxillus involutus* and *Piloderma croceum*. *New Phytol.* **138,** 203–212 (1998).

76. Kurth, F. et al. Large scale transcriptome analysis reveals interplay between development of forest trees and a beneficial mycorrhiza helper bacterium. *BMC Genomics* **16,** 658 (2015).

77. Chen, L.-Q. et al. Sucrose efflux mediated by SWEET proteins as a key step for phloem transport. *Science (80-.).* **335,** 207–211 (2012).

78. Manck-Götzenberger, J. & Requena, N. Arbuscular mycorrhiza symbiosis induces a major transcriptional reprogramming of the potato SWEET sugar transporter family. *Front. Plant Sci.* **7,** 1–14 (2016).

79. Manck-Götzenberger, J. & Requena, N. Arbuscular mycorrhiza symbiosis induces a major transcriptional reprogramming of the potato SWEET sugar transporter family. *Front. Plant Sci.* **7,** 1–14 (2016).

80. Perotto, S. et al. Gene expression in mycorrhizal orchid protocorms suggests a friendly plant-fungus relationship. *Planta* **239,** 1337–1349 (2014).

81. Guo, W.-J. et al. SWEET17, a facilitative transporter, mediates fructose transport across the tonoplast of Arabidopsis roots and leaves. *Plant Physiol.* **164,** 777–89 (2014).

82. Couturier, J., Chibani, K., Jacquot, J.-P. & Rouhier, N. Cysteine-based redox regulation and signaling in plants. *Front. Plant Sci.* **4,** 105 (2013).

83. Couturier, J., Jacquot, J. P. & Rouhier, N. Evolution and diversity of glutaredoxins in photosynthetic organisms. *Cell. Mol. Life Sci.* **66,** 2539–2557 (2009).

84. Chibani, K., Wingsle, G., Jacquot, J. P., Gelhaye, E. & Rouhier, N. Comparative fenomic study of the thioredoxin family in photosynthetic organisms with emphasis on *populus trichocarpa*. *Mol. Plant* **2,** 308–322 (2009).

85. Lallement, P. A., Brouwer, B., Keech, O., Hecker, A. & Rouhier, N. The still mysterious roles of cysteine-containing glutathione transferases in plants. *Front. Pharmacol.* **5,** 1–22 (2014).

86. Limkaisang, S. et al. Molecular phylogenetic analyses reveal a close relationship between powdery mildew fungi on some tropical trees and *Erysiphe alphitoides*, an oak powdery mildew. *Mycoscience* **47,** 327–335 (2006).

87. Glawe, D. A. The powdery mildews: a review of the world's most familiar (yet poorly

known) plant pathogens. *Annu. Rev. Phytopathol.* **46,** 27–51 (2008).

88. Jørgensen, J. H. Discovery, characterization and exploitation of Mlo powdery mildew resistance in barley. *Euphytica* **63,** 141–152 (1992).

89. Büschges, R. et al. The barley Mlo gene: a novel control element of plant pathogen resistance. *Cell* **88,** 695–705 (1997).

90. Piffanelli, P. et al. A barley cultivation-associated polymorphism conveys resistance to powdery mildew. *Nature* **430,** 887–891 (2004).

91. Acevedo-Garcia, J., Kusch, S. & Panstruga, R. *Magical mystery tour*: MLO proteins in plant immunity and beyond. *J. Physiol.* **204,** 273–281 (2014).

92. Pessina, S. et al. Characterization of the MLO gene family in Rosaceae and gene expression analysis in *Malus domestica*. *BMC Genomics* **15,** 618 (2014).

93. Kusch, S., Pesch, L. & Panstruga, R. Comprehensive phylogenetic analysis sheds light on the diversity and origin of the MLO family of integral membrane proteins. *Genome Biol. Evol.* **8,** 878–895 (2016).

94. Kessler, S. A. et al. Conserved molecular components for pollen tube reception and fungal invasion. *Science (80-.).* **330,** 968–971 (2010).

95. Consonni, C. et al. Conserved requirement for a plant host cell protein in powdery mildew pathogenesis. *Nat. Genet.* **38,** 716–720 (2006).

96. Steuernagel, B., Jupe, F., Witek, K., Jones, J. D. G. & Wulff, B. B. H. NLR-parser: rapid annotation of plant NLR complements. *Bioinformatics* **31,** 1665–1667 (2015).

97. Marchler-Bauer, A. et al. CDD: NCBI's conserved domain database. *Nucleic Acids Res.* **43,** D222–D226 (2015).

98. Sarris, P. F., Cevik, V., Dagdas, G., Jones, J. D. G. & Krasileva, K. V. Comparative analysis of plant immune receptor architectures uncovers host proteins likely targeted by pathogens. *BMC Biol.* **14,** 8 (2016).

99. Kroj, T., Chanclud, E., Michel-Romiti, C., Grand, X. & Morel, J. B. Integration of decoy domains derived from protein targets of pathogen effectors into plant immune receptors is widespread. *New Phytol.* **210,** 618–626 (2016).

100. Le Roux, C. et al. A receptor pair with an integrated decoy converts pathogen disabling of transcription factors to immunity. *Cell* **161,** 1074–1088 (2015).

101. Sarris, P. F. et al. A plant immune receptor detects pathogen effectors that target WRKY transcription factors. *Cell* **161,** 1089–1100 (2015).

102. Meyers, B. C., Kozik, A., Griego, A., Kuang, H. & Michelmore, R. W. Genome-wide analysis of NBS-LRR – encoding genes in Arabidopsis. *Plant Cell* **15,** 809–834 (2003).

103. Mun, J. H., Yu, H. J., Park, S. & Park, B. S. Genome-wide identification of NBS-encoding resistance genes in *Brassica rapa*. *Mol. Genet. Genomics* **282,** 617–631 (2009).

104. Kohler, A. *et al.* Genome-wide identification of NBS resistance genes in *Populus trichocarpa*. *Plant Mol. Biol.* **66,** 619–636 (2008).

105. Jupe, F. et al. Identification and localisation of the NB-LRR gene family within the potato genome. *BMC Genomics* **13,** 75 (2012).

106. Eddy, S. R. A probabilistic model of local sequence alignment that simplifies statistical significance estimation. *PLoS Comput. Biol.* **4,** e1000069 (2008).

107. Gouy, M., Guindon, S. & Gascuel, O. SeaView version 4: a multiplatform graphical user interface for sequence alignment and phylogenetic tree building. *Mol. Biol. Evol.* **27,** 221–224 (2010).

108. Stamatakis, A. RAxML-VI-HPC: maximum likelihood-based phylogenetic analyses with thousands of taxa and mixed models. *Bioinformatics* **22,** 2688–2690 (2006).

109. Eddy, S. R. a New Generation of Homology Search Tools Based on Probabilistic Inference. *Genome Informatics* **23,** 205–211 (2009).

110. Katoh, K., Kuma, K. I., Toh, H. & Miyata, T. MAFFT version 5: improvement in accuracy of multiple sequence alignment. *Nucleic Acids Res.* **33,** 511–518 (2005).

111. Capella-Gutiérrez, S., Silla-Martínez, J. M. & Gabaldón, T. trimAl: a tool for automated alignment trimming in large-scale phylogenetic analyses. *Bioinformatics* **25,** 1972–1973 (2009).

112. Price, M. N., Dehal, P. S. & Arkin, A. P. FastTree 2--approximately maximum-likelihood trees for large alignments. *PLoS One* **5,** e9490 (2010).

113. Fischer, I., Diévart, A., Droc, G., Dufayard, J.-F. & Chantret, N. Evolutionary dynamics of the leucine-rich repeat receptor-like kinase (LRR-RLK) subfamily in angiosperms. *Plant Physiol.* **170,** 1595–1610 (2016).

114. Guindon, S. et al. New algorithms and methods to estimate maximum-likelihood phylogenies: Assessing the performance of PhyML 3.0. *Syst. Biol.* **59,** 307–321 (2010).

115. Fischer, I. et al. Impact of recurrent gene duplication on adaptation of plant genomes. *BMC Plant Biol.* **14,** 151 (2014).

116. Löytynoja, A. & Goldman, N. An algorithm for progressive multiple alignment of sequences with insertions. *Proc. Natl. Acad. Sci. U. S. A.* **102,** 10557–62 (2005).

117. Penn, O., Privman, E., Landan, G., Graur, D. & Pupko, T. An alignment confidence score capturing robustness to guide tree uncertainty. *Mol. Biol. Evol.* **27,** 1759–1767 (2010).

118. De Mita, S. & Siol, M. EggLib: processing, analysis and simulation tools for population genetics and genomics. *BMC Genet.* **13,** 27 (2012).

119. Yang, Z. PAML 4: phylogenetic analysis by maximum likelihood. *Mol. Biol. Evol.* **24,** 1586–1591 (2007).

120. Enkhbayar, P., Kamiya, M., Osaki, M., Matsumoto, T. & Matsushima, N. Structural principles of leucine-rich repeat (LRR) proteins. *Proteins Struct. Funct. Genet.* **54,**

394–403 (2004).

121. Jones, D. A. & Jones, J. D. G. The role of leucine-rich repeat proteins in plant defences. *Adv. Bot. Res.* **24,** 89–167 (1997).

122. Parniske, M. et al. Novel disease resistance specificities result from sequence exchange between tandemly repeated genes at the Cf-4/9 locus of tomato. *Cell* **91,** 821–832 (1997).

123. Wang, G.-L. Xa21D encodes a receptor-like molecule with a leucine-rich repeat domain that determines race-specific recognition and is subject to adaptive evolution. *Plant Cell Online* **10,** 765–780 (1998).

124. Werner, R. A. et al. Biosynthesis of gallic acid in *Rhus typhina*: discrimination between alternative pathways from natural oxygen isotope abundance. *Phytochemistry* **65,** 2809–2813 (2004).

125. Niemetz, R. & Gross, G. G. Enzymology of gallotannin and ellagitannin biosynthesis. *Phytochemistry* **66,** 2001–2011 (2005).

126. Mittasch, J., Böttcher, C., Frolova, N., Bönn, M. & Milkowski, C. Identification of UGT84A13 as a candidate enzyme for the first committed step of gallotannin biosynthesis in pedunculate oak (*Quercus robur*). *Phytochemistry* **99,** 44–51 (2014).

127. Tzin, V. & Galili, G. The biosynthetic pathways for shikimate and aromatic amino acids in *Arabidopsis thaliana*. *Arab. B.* **8,** e0132 (2010).

128. Ross, J., Li, Y., Lim, E. & Bowles, D. J. Higher plant glycosyltransferases. *Genome Biol.* **2,** 3004 (2001).

129. Yonekura-Sakakibara, K. & Hanada, K. An evolutionary view of functional diversity in family 1 glycosyltransferases. *Plant J.* **66,** 182–193 (2011).

130. McCaig, B. C., Meagher, R. B. & Dean, J. F. D. Gene structure and molecular analysis of the laccase-like multicopper oxidase (LMCO) gene family in *Arabidopsis thaliana*.

Planta **221,** 619–636 (2005).

131. Messerschmidt, A. & Huber, R. The blue oxidases, ascorbate oxidase, laccase and ceruloplasmin modelling and structural relationships. *Eur. J. Biochem.* **187,** 341–352 (1990).

132. Berthet, S. et al. Role of plant laccases in lignin polymerization. *Adv. Bot. Res.* **61,** 145–172 (2012).

133 Pourcel, L. et al. Transparent Testa10 encodes a laccase-like enzyme involved in oxidative polymerization of flavonoids in Arabidopsis seed coat. *Plant Cell* **17,** 2966–2980 (2005).

134. Zhao, Q. et al. LACCASE is necessary and nonredundant with PEROXIDASE for lignin polymerization during vascular development in Arabidopsis. *Plant Cell* **25,** 3976–3987 (2013).

135. Lu, S. et al. Ptr-miR397a is a negative regulator of laccase genes affecting lignin content in *Populus trichocarpa*. *Proc. Natl. Acad. Sci. U. S. A.* **110,** 10848–53 (2013).

136. Dobin, A. et al. STAR: ultrafast universal RNA-seq aligner. *Bioinformatics* **29,** 15–21 (2013).

137. Li, H. et al. The Sequence Alignment/Map format and SAMtools. *Bioinformatics* **25,** 2078–2079 (2009).

138. Pertea, M. et al. StringTie enables improved reconstruction of a transcriptome from RNA-seq reads. *Nat. Biotechnol.* **33,** 290–5 (2015).

139. Lowe, T. M. & Eddy, S. R. tRNAscan-SE: a program for inproved detection of transfer RNA genes in genomic sequence. *Nucleic Acids Res.* **25,** 955–964 (1997).

140. Lagesen, K. et al. RNAmmer: consistent and rapid annotation of ribosomal RNA genes. *Nucleic Acids Res.* **35,** 3100–3108 (2007).

141. Nawrocki, E. P. & Eddy, S. R. Infernal 1.1: 100-fold faster RNA homology searches. *Bioinformatics* **29,** 2933–2935 (2013).

142. Nawrocki, E. P. et al. Rfam 12.0: updates to the RNA families database. *Nucleic Acids Res.* **43,** D130–D137 (2015).

143. Kozomara, A. & Griffiths-Jones, S. MiRBase: annotating high confidence microRNAs using deep sequencing data. *Nucleic Acids Res.* **42,** D68-73 (2014).

144. Camacho, C. et al. BLAST+: architecture and applications. *BMC Bioinformatics* **10,** 421 (2009).

145. Langmead, B. & Salzberg, S. L. Fast gapped-read alignment with Bowtie 2. *Nat Methods* **9,** 357–359 (2012).

146. Liao, Y., Smyth, G. K. & Shi, W. FeatureCounts: an efficient general purpose program for assigning sequence reads to genomic features. *Bioinformatics* **30,** 923–930 (2014).

147. Quinlan, A. R. & Hall, I. M. BEDTools: a flexible suite of utilities for comparing genomic features. *Bioinformatics* **26,** 841–842 (2010).

148. Chen, J., Gl, S. & Lascoux, M. Genetic diversity and the efficacy of purifying selection across plant and animal species. *Mol. Biol. Evol.* **34**, 1417–1428 (2017).

149. Fitzjohn, R. G. et al. How much of the world is woody? *J. Ecol.* **102,** 1266–1272 (2014).

150. Beaulieu, J. M., O'Meara, B. C. & Donoghue, M. J. Identifying hidden rate changes in the evolution of a binary morphological character: the evolution of plant habit in campanulid angiosperms. *Syst. Biol.* **62,** 725–737 (2013).

151. Lens, F. et al. Embolism resistance as a key mechanism to understand adaptive plant strategies. *Current Opinion in Plant Biology* **16,** 287–292 (2013).

152. Zanne, A. E. et al. Three keys to the radiation of angiosperms into freezing environments. *Nature* **506,** 89–92 (2014).

153. Guo, S. et al. The draft genome of watermelon (*Citrullus lanatus*) and resequencing of 20 diverse accessions. *Nat. Genet.* **45,** 51–8 (2013).

154. Shulaev, V. et al. The genome of woodland strawberry (*Fragaria vesca*). *Nat. Genet.* **43,** 109–116 (2011).

155. Schmutz, J. et al. Genome sequence of the palaeopolyploid soybean. *Nature* **463,** 178–83 (2010).

156. Chan, A. P. et al. Draft genome sequence of the oilseed species *Ricinus communis*. *Nat. Biotechnol.* **28,** 951–6 (2010).

157. Sanderkar, M. & Nielsen, K. L. Genome sequence and analysis of the tuber crop potato : the potato genome sequencingconsortium. *Nature* **476,** 189–195 (2011).

158. Tuskan, G. A. et al. The genome of black cottonwood, *Populus trichocarpa* (Torr. & Gray). *Science* **313,** 1596–604 (2006).

159. Motamayor, J. C. et al. The genome sequence of the most widely cultivated cacao type and its use to identify candidate genes regulating pod color. *Genome Biol.* **14,** r53 (2013).

160. Jaillon, O. et al. The grapevine genome sequence suggests ancestral hexaploidization in major angiosperm phyla. *Nature* **449,** 463–7 (2007).

161. Chen, Z. et al. Two seven-transmembrane domain MILDEW RESISTANCE LOCUS O proteins cofunction in Arabidopsis root thigmomorphogenesis. *Plant Cell* **21,** 1972–1991 (2009).

162. Iovieno, P. et al. Structure , evolution and functional inference on the Mildew Locus O (MLO) gene family in three cultivated. *BMC Genomics* 1–13 (2015).

163. Zhou, S. J., Jing, Z. & Shi, J. L. Genome-wide identification, characterization, and

expression analysis of the MLO gene family in *Cucumis sativus*. *Genet. Mol. Res.* **12,** 6565–78 (2013).

164. Deshmukh, R. & Singh, V. K. S. B. D. Comparative phylogenetic analysis of genome ‑ wide Mlo gene family members from *Glycine max* and *Arabidopsis thaliana*. 345–359 (2014).

165. Wang, X. et al. Genome-wide characterization and comparative analysis of the MLO gene family in cotton. *Plant Physiol. Biochem.* **103,** 106–119 (2016).

166. Liu, Q. & Zhu, H. Molecular evolution of the MLO gene family in *Oryza sativa* and their functional divergence. *Gene* **409,** 1–10 (2008).

167. Jiwan, D., Roalson, E. H., Main, D. & Dhingra, A. Antisense expression of peach mildew resistance locus O (PpMlo1) gene confers cross-species resistance to powdery mildew in *Fragaria x ananassa*. *Transgenic Res.* **22,** 1119–1131 (2013).

168. Chen, Y., Wang, Y., Zhang, H. & others. Genome-wide analysis of the mildew resistance locus o ('MLO') gene family in tomato (*Solanum lycopersicum* L.). *Plant Omics* **7,** 87 (2014).

169. Appiano, M. et al. Identification of candidate MLO powdery mildew susceptibility genes in cultivated Solanaceae and functional characterization of tobacco NtMLO1. *Transgenic Res.* **24,** 847–858 (2015).

170. Konishi, S., Sasakuma, T. & Sasanuma, T. Identification of novel Mlo family members in wheat and their genetic characterization. *Genes Genet. Syst.* **85,** 167–175 (2010).

171. Feechan, A., Jermakow, A. M., Torregrosa, L., Panstruga, R. & Dry, I. B. Identification of grapevine MLO gene candidates involved in susceptibility to powdery mildew. *Funct. Plant Biol.* **35,** 1255–1266 (2008).

172. Chai, G. et al. R2R3-MYB gene pairs in Populus: Evolution and contribution to secondary wall formation and flowering time. *J. Exp. Bot.* **65,** 4255–4269 (2014).

173. Katoh, K., Misawa, K., Kuma, K. & Miyata, T. MAFFT: a novel method for rapid multiple sequence alignment based on fast Fourier transform. *Nucleic Acids Res.* **30,** 3059–3066 (2002).

174. Tamura, K. et al. MEGA5: Molecular evolutionary genetics analysis using maximum likelihood, evolutionary distance, and maximum parsimony methods. *Mol. Biol. Evol.* **28,** 2731–2739 (2011).

175. Sievers, F. et al. Fast, scalable generation of high-quality protein multiple sequence alignments using Clustal-Omega. *Mol. Syst. Biol.* **7,** 539 (2011).

176. Castresana, J. Selection of conserved blocks from multiple alignments for their use in phylogenetic analysis. *Mol. Biol. Evol.* **17,** 540–552 (2000).

177. Gascuel, O. BioNJ: an improved version of the NJ algorithm based on a simple model of sequence data. *Mol. Biol. Evol.* **14,** 685–95 (1997).

178. Dereeper, A. et al. Phylogeny.fr: robust phylogenetic analysis for the non-specialist. *Nucleic Acids Res* **36,** W465--9 (2008).

9. Supplementary Data Sets

Supplementary Data Set 1 List of 25,808 oak gene models with their annotations.

Supplementary Data Set 2 Mapping data used to anchor the scaffolds onto the oak genetic linkage map. Sheet #1: List of 5,589 mapped markers. Sheet #2: Subset of 2,615 markers matching sequence scaffolds, classified by categories according to **Supplementary Table 27**. Sheet #3: syntenic relationships between oak markers and peach gene models. Sheet #4 ordered scaffolds along the 12 chromosomes.

Supplementary Data Set 3 List of orthogroups (orthoMCL analysis) and expanded gene families (CAFE analysis) in pedunculate oak. Sheet #1 List of clusters obtained with orthoMCL. The family *P*-value is provided by CAFE and corresponds to the probability of observing the data (orthogroup size distribution between taxa). Orthogroups with larger size variance are expected to have lower *P*-values. The Qr_*P*-value is the oak branch-specific *P*-value. It corresponds to the probability of transitions between the parent and child family sizes for the oak branch. A low *P*-value indicates a rapidly evolving orthogroup. These data were provided by CAFE. Sheet #2 List of clusters expanded in oak. Sheet#3 list of outstanding outlier clusters expanded in oak. Sheet#4 list of clusters contracted in oak.

Supplementary Data Set 4 List of gene categories. Sheet #1: tandemly duplicated genes (TDG). Sheet #2: list of TDG relationships. Sheet #3: long distance-duplicated genes (LDG). Sheet #4: singleton genes (SG).

Supplementary Data Set 5 Classification of NB-LRR-related genes. List of oak NB-LRR-related genes. For each gene, the gene model ID and the proteinID are provided. NB-LRR genes were classified into categories according to their canonical domains, i.e. CC, coiled-coil; LRR, leucine-rich repeat; NB, nucleotide-binding; RPW8, resistance to powdery mildew

protein; TIR, Toll interleukin receptor-like; X, putative integrated decoy. The position of the genes (gene start and gene end) on pseudomolecules or unassigned scaffolds is indicated. The orthogroup ID is also provided for the orthoMCL analysis, together with the family expansion/contraction status (oak vs. other species, 1% threshold), and tandem duplication status.

Supplementary Data Set 6 Classification of RLK-related genes. Sheet #1: list of RLK-related genes from oak, Arabidopsis and rice - phylogeny and assignment. Sheet #2: subgroups of RLK-related genes and subgroups within LRR-RLK from sheet #1. Sheet #3 data from Fischer et al.[113] for comparison with oak. Sheet #4: list of oak genes from sheet #1, with their classification into orthoMCL orthogroups and their status (in a tandem array or not). Sheet #5: results from sheet #4.

Supplementary Data Set 7 Summary of orthogroups expanded in 'trees'. Sheet #1: *P*-value and FDR for all orthoMCL orthogroups. Sheet #2: orthogroups expanded (FDR<0.05) in 'trees'. Shee #3: list of outstanding 'tree' orthogroups and their functional annotations. Sheet #4: orthogroups expanded [contracted] in herbaceous species [trees].

Supplementary Data Set 8 Summary of the Gene Ontology (GO) term analysis showing significantly enrichment in GO terms for molecular functions (MF), biological processes (BP), and cellular components (CC). Sheet #1: tandem duplicated genes (TDGs). Sheet #2: long distance-duplicated genes (LDGs). Sheet #3: singletons (SGs). Sheet #4: orthogroups expanded in oak. Sheet #5: orthogroups expanded in woody perennials. Sheet #6: expanded orthogroups in herbaceous species. *P*-values are from Fisher's exact tests.

Supplementary Data Set 9 Footprint of selection in RLK-related genes. Sheet #1: ID of ultraparalogous genes with their association group, annotation, orthoMCL orthogroup and

label from **Fig. 3d and 4b**. Sheet #2: codeml results for 24 groups of ultraparalogs. Sheet #3: results of all the manually validated sites (domain plus position in the LRR motif).

Supplementary Data Set 10 List of pedunculate oak BAC clones used in this study. Sheet #1: list of sequenced BAC clones and matching scaffolds on the diploid version of the oak genome sequence. Sheet #2: gene annotation on the sequenced BACs.

10. Supplementary Tables

Supplementary Table 1 List of genomic and cDNA libraries used to sequence and annotate the pedunculate oak genome accession "3P". DNAseq: genomic libraries used to sequence the oak genome. RNAseq: cDNA libraries used to annotate the oak genome.

Project	Material	Filename	# reads	# bases	Library	Technology	Accession ID	Diploid genome (1.5G/2C) coverage
**DNAseq**								
Qr	A	Oak_7_1_sequence.fastq	62418280	4681371000	Paired-end	Illumina (GAIIx)	SRX739064	3x
Qr	B	Oak_5Kb_MatePair_6_1_sequence.fastq	56012498	2800624900	Mate-pairs 5 Kb	Illumina (GAIIx)	SRX739054	2x
AWU	A	AWU_AOSC_3_D11KBACXX.IND3	183557983	35871118970	Mate-pairs 3Kb	Illumina	ERX546778	24x
AWU	A	AWU_AOSF_1_C0BULACXX.IND1	33955645	6731457878	overlapping PE	Illumina	ERX546816	86x
AWU	A	AWU_AOSF_1_D0J4FACXX.IND1	184348978	36406701485	overlapping PE	Illumina	ERX546795	
AWU	A	AWU_AOSF_2_D0J4FACXX.IND1	173912862	34132669204	overlapping PE	Illumina	ERX546803	
AWU	A	AWU_AOSF_4_D0J4KACXX.IND1	118761014	23565095820	overlapping PE	Illumina	ERX546766	
AWU	A	AWU_AOSF_6_C0D1LACXX.IND1	142592731	28210290830	overlapping PE	Illumina	ERX546794	
AWU	A	AWU_AOSN_2_C2MP1ACXX.IND18	112059267	18207771474	Mate-pairs Nextera 3 Kb	Illumina	ERX546793	26x
AWU	A	AWU_AOSN_4_D2BM7ACXX.IND18	130196132	21273821373	Mate-pairs Nextera 3 Kb	Illumina	ERX546821	
AWU	A	AWU_AOSN_1_C2MP1ACXX.IND2	128515390	20882553184	Mate-pairs Nextera 5 Kb	Illumina	ERX546851	35x
AWU	A	AWU_AOSN_4_D2C4BACXX.IND2	128931290	21392347587	Mate-pairs Nextera 5 Kb	Illumina	ERX546756	
AWU	A	AWU_AOSN_7_D25ULACXX.IND2	63924125	10508889688	Mate-pairs Nextera 5 Kb	Illumina	ERX546847	
AWU	A	AWU_AOSN_4_D2C5KACXX.IND4	132901113	21958813992	Mate-pairs Nextera 8 Kb	Illumina	ERX546787	22x
AWU	A	AWU_AOSN_7_D25ULACXX.IND4	65407657	10724020167	Mate-pairs Nextera 8 Kb	Illumina	ERX546842	
AWU	A	AWU_AORS_HLG9GIU01	376951	139474495	Single Reads	454	ERX546760	15x
AWU	A	AWU_AORS_HO6MKXJ01	615920	275537385	Single Reads	454	ERX546817	
AWU	A	AWU_AORS_HO6MKXJ02	600921	244313411	Single Reads	454	ERX546828	

AWU	A	AWU_AORS_HO8LWZP01	566389	250562733	Single Reads	454	ERX546783
AWU	A	AWU_AORS_HO8LWZP02	576975	250783193	Single Reads	454	ERX546855
AWU	A	AWU_AORS_HOGWG9K01	471512	216412101	Single Reads	454	ERX546798
AWU	A	AWU_AORS_HOGWG9K02	623520	290152549	Single Reads	454	ERX546765
AWU	A	AWU_AORS_HOKMQ7201	415422	194219290	Single Reads	454	ERX546804
AWU	A	AWU_AORS_HOKMQ7202	415767	194112470	Single Reads	454	ERX546810
AWU	A	AWU_AORS_HOTXFWN01	573904	249396146	Single Reads	454	ERX546789
AWU	A	AWU_AORS_HOTXFWN02	616636	251440354	Single Reads	454	ERX546797
AWU	A	AWU_AORS_HOXJLF101	619309	271457362	Single Reads	454	ERX546826
AWU	A	AWU_AORS_HOXJLF102	589373	262356681	Single Reads	454	ERX546833
AWU	A	AWU_AORS_HP9GW5D01	616582	329032439	Single Reads	454	ERX546796
AWU	A	AWU_AORS_HPAKMIN01	604431	247521317	Single Reads	454	ERX546808
AWU	A	AWU_AORS_HPAKMIN02	599433	252223557	Single Reads	454	ERX546799
AWU	A	AWU_AORS_HPDXEDZ01	576140	267133896	Single Reads	454	ERX546781
AWU	A	AWU_AORS_HPDXEDZ02	604043	268649166	Single Reads	454	ERX546786
AWU	A	AWU_AORS_HPJN7LD01	627802	303967961	Single Reads	454	ERX546856
AWU	A	AWU_AORS_HPJN7LD02	632803	301178645	Single Reads	454	ERX546780
AWU	A	AWU_AORS_HPLH4SP01	574419	279919660	Single Reads	454	ERX546839
AWU	A	AWU_AORS_HPLH4SP02	541488	269140415	Single Reads	454	ERX546763
AWU	A	AWU_AORS_HPNH9JK01	566545	277699403	Single Reads	454	ERX546825
AWU	A	AWU_AORS_HPNH9JK02	542232	250634628	Single Reads	454	ERX546835
AWU	A	AWU_AORS_HPPEQTK01	509099	253840880	Single Reads	454	ERX546755
AWU	A	AWU_AORS_HPPEQTK02	397398	183638911	Single Reads	454	ERX546776
AWU	A	AWU_AORS_HPQ6PEM01	324214	140937382	Single Reads	454	ERX546759

AWU	A	AWU_AORS_HPQ6PEM02	604340	258006924	Single Reads	454	ERX546792
AWU	A	AWU_AORS_HPWTB8401	632065	303346757	Single Reads	454	ERX546782
AWU	A	AWU_AORS_HPWTB8402	628856	283953031	Single Reads	454	ERX546850
AWU	A	AWU_AORS_HPYQNEV01	541025	254463651	Single Reads	454	ERX546785
AWU	A	AWU_AORS_HPYQNEV02	595941	281903473	Single Reads	454	ERX546775
AWU	A	AWU_AORS_HQ3AIIJ01	645852	273877444	Single Reads	454	ERX546812
AWU	A	AWU_AORS_HQ3AIIJ02	635565	261155309	Single Reads	454	ERX546853
AWU	A	AWU_AORS_HQ6XQKF01	602974	279814892	Single Reads	454	ERX546854
AWU	A	AWU_AORS_HQ6XQKF02	450682	192755614	Single Reads	454	ERX546857
AWU	A	AWU_AORS_HQBAEKW02	638393	315269575	Single Reads	454	ERX546779
AWU	A	AWU_AORS_HQC2HZF03	233986	110390642	Single Reads	454	ERX546820
AWU	A	AWU_AORS_HQC2HZF04	252397	132477279	Single Reads	454	ERX546757
AWU	A	AWU_AORS_HQC7JZG01	601648	292351551	Single Reads	454	ERX546829
AWU	A	AWU_AORS_HQC7JZG02	598632	280339696	Single Reads	454	ERX546824
AWU	A	AWU_AORS_HQE6HNV01	575033	294218540	Single Reads	454	ERX546837
AWU	A	AWU_AORS_HQE6HNV02	562072	276129568	Single Reads	454	ERX546771
AWU	A	AWU_AORS_HQG0JXZ01	605366	302641389	Single Reads	454	ERX546843
AWU	A	AWU_AORS_HQG0JXZ02	608598	289064135	Single Reads	454	ERX546774
AWU	A	AWU_AORS_HQR99NJ02	267774	134696910	Single Reads	454	ERX546806
AWU	A	AWU_AORS_HQR99NJ03	268450	136910337	Single Reads	454	ERX546840
AWU	A	AWU_AORS_HQR99NJ04	259009	128977810	Single Reads	454	ERX546813
AWU	A	AWU_AORS_HQVT54K01	670862	357817336	Single Reads	454	ERX546767
AWU	A	AWU_AORS_HQVT54K02	629420	327604262	Single Reads	454	ERX546852
AWU	A	AWU_AORS_HQXPWBU01	575053	311123919	Single Reads	454	ERX546834

AWU	A	AWU_AORS_HQXPWBU02	592747	314220456	Single Reads	454	ERX546814
AWU	A	AWU_AORS_HR0B5WL01	476167	129297327	Single Reads	454	ERX546762
AWU	A	AWU_AORS_HR0B5WL02	522655	143434538	Single Reads	454	ERX546832
AWU	A	AWU_AORS_HR7Y0Z201	494273	151280248	Single Reads	454	ERX546827
AWU	A	AWU_AORS_HR7Y0Z202	451613	137776321	Single Reads	454	ERX546773
AWU	A	AWU_AORS_HRJY4EM01	583444	236195624	Single Reads	454	ERX546772
AWU	A	AWU_AORS_HRJY4EM02	507965	197165750	Single Reads	454	ERX546805
AWU	A	AWU_AORS_HRLT5NJ01	439153	146160122	Single Reads	454	ERX546777
AWU	A	AWU_AORS_HRLT5NJ02	400105	146048898	Single Reads	454	ERX546822
AWU	A	AWU_AORS_HRS9HCN01	472104	238860086	Single Reads	454	ERX546802
AWU	A	AWU_AORS_HRS9HCN02	540831	273328131	Single Reads	454	ERX546800
AWU	A	AWU_AORS_HRWNJ0F01	332429	137572971	Single Reads	454	ERX546819
AWU	A	AWU_AORS_HRWNJ0F02	483017	197840503	Single Reads	454	ERX546836
AWU	A	AWU_AORS_HRYGP8A01	593882	234911883	Single Reads	454	ERX546764
AWU	A	AWU_AORS_HRYGP8A02	568850	225739689	Single Reads	454	ERX546809
AWU	A	AWU_AORS_HS3NGS202	593496	271995164	Single Reads	454	ERX546848
AWU	A	AWU_AORS_HSBPWMC07	91079	36958456	Single Reads	454	ERX546791
AWU	A	AWU_AORS_HSBPWMC08	85328	34801846	Single Reads	454	ERX546801
AWU	A	AWU_AORS_HSDIBFO01	581079	282921477	Single Reads	454	ERX546838
AWU	A	AWU_AORS_HSDIBFO02	592838	266142240	Single Reads	454	ERX546784
AWU	A	AWU_AORS_HSFJ8MK01	607026	254366558	Single Reads	454	ERR588819
AWU	A	AWU_AORS_HSFJ8MK02	598573	217174740	Single Reads	454	ERX546849
AWU	A	AWU_AORS_HT2R6K001	616436	354909746	Single Reads	454	ERX546818
AWU	A	AWU_AORS_HTAXONP01	492955	227121584	Single Reads	454	ERX546770

AWU	A	AWU_AORS_HTAXONP02	605770	266418303	Single Reads	454	ERX546844	
AWU	A	AWU_AORS_HTCPZFN01	586104	289733463	Single Reads	454	ERX546788	
AWU	A	AWU_AORS_HTCPZFN02	620227	293411238	Single Reads	454	ERX546807	
AWU	A	AWU_AORS_HTEFU8101	598533	239492636	Single Reads	454	ERX546769	
AWU	A	AWU_AORS_HTEFU8102	622271	230573314	Single Reads	454	ERX546841	
AWU	A	AWU_AORS_HTNM2OH02	602554	281689812	Single Reads	454	ERX546830	
AWU	A	AWU_AORS_HTRLXNS01	618531	306184837	Single Reads	454	ERX546811	
AWU	A	AWU_AORS_HTRLXNS02	614926	297895268	Single Reads	454	ERX546754	
AWU	A	AWU_AORS_HTRQELM01	582923	257934536	Single Reads	454	ERX546846	
AWU	A	AWU_AORS_HTRQELM02	619410	261141889	Single Reads	454	ERX546768	
AWU	A	AWU_AORS_HTTFXII01	657236	291923817	Single Reads	454	ERX546845	
AWU	A	AWU_AORS_HTTFXII02	642957	282417197	Single Reads	454	ERX546761	
AWU	A	AWU_AORS_HTTJ7TZ01	592087	288519958	Single Reads	454	ERX546831	
AWU	A	AWU_AORS_HTTJ7TZ02	594370	280348006	Single Reads	454	ERX546758	
AWU	A	AWU_AORS_HTVBL6N01	660475	383854941	Single Reads	454	ERX546823	
AWU	A	AWU_AORS_HTVBL6N02	634782	363284614	Single Reads	454	ERX546815	
AWU	A	AWU_AORS_HTVFTYI02	664033	336384976	Single Reads	454	ERX546790	
BBX	A	BBX_AOSW_1_D1D53ACXX.IND5	178225679	34704998557	Paired-end	Illumina	ERX697294	471x
BBX	A	BBX_AOSW_1_H32GMBCXX.IND5	129340482	61406442546	Paired-end	Illumina	ERX1886616	
BBX	A	BBX_AOSW_1_H57N7BCXX.IND5	114968046	54158595214	Paired-end	Illumina	ERX1886621	
BBX	A	BBX_AOSW_2_H32GMBCXX.IND5	132162999	63021298371	Paired-end	Illumina	ERX1886622	
BBX	B	BBX_BOSW_2_C1CRDACXX.IND6	185226443	36388653397	Paired-end	Illumina	ERX697299	
BBX	C	BBX_COSW_1_H072TAMXX.IND7	88896954	42521091728	Paired-end	Illumina	ERX697298	
BBX	C	BBX_COSW_2_D1D53ACXX.IND7	187527862	36107158449	Paired-end	Illumina	ERX697297	

BBX	C	BBX_COSW_2_H072TAMXX.IND7	89768432	42909747910	Paired-end	Illumina	ERX697296	
BBX	C	BBX_COSW_2_H57N7BCXX.IND7	141600651	66340720909	Paired-end	Illumina	ERX1886620	
BBX	D	BBX_DOSW_3_C1CRDACXX.IND8	195620139	38283308256	Paired-end	Illumina	ERX697295	
BBX	E	BBX_EOSW_1_H55MLBCXX.IND9	127908274	59324914635	Paired-end	Illumina	ERX1886617	
BBX	E	BBX_EOSW_2_H32GLBCXX.IND9	134000800	55186245541	Paired-end	Illumina	ERX1886619	
BBX	E	BBX_EOSW_2_H55MLBCXX.IND9	104846648	48165716278	Paired-end	Illumina	ERX1886618	
BBX	E	BBX_EOSW_3_D1D53ACXX.IND9	185076013	35974428528	Paired-end	Illumina	ERX697292	
BBX	F	BBX_FOSW_4_D1D53ACXX.IND10	173673937	33077225178	Paired-end	Illumina	ERX697293	
AWU	A2	LR6000024-DNA_B02-LRAAD-01	96268	477206869	TruSeq Synthetic Reads	Illumina	ERX1936767	6x
AWU	A2	LR6000024-DNA_B02-LRAAD-02	97511	485762221	TruSeq Synthetic Reads	Illumina	ERX1936768	
AWU	A2	LR6000024-DNA_B02-LRAAD-03	99930	497943141	TruSeq Synthetic Reads	Illumina	ERX1936769	
AWU	A2	LR6000024-DNA_B02-LRAAD-04	96777	481729766	TruSeq Synthetic Reads	Illumina	ERX1936770	
AWU	A2	LR6000024-DNA_B02-LRAAD-05	99331	488240514	TruSeq Synthetic Reads	Illumina	ERX1936771	
AWU	A2	LR6000024-DNA_B02-LRAAD-06	133440	621932686	TruSeq Synthetic Reads	Illumina	ERX1936772	
AWU	A2	LR6000024-DNA_B02-LRAAD-07	212972	890601310	TruSeq Synthetic Reads	Illumina	ERX1936773	
AWU	G1	AWU_msDDZ	166474	741066645	TruSeq Synthetic Reads	Illumina	ERX1936761	
AWU	G1	AWU_msDEA	171897	734825556	TruSeq Synthetic Reads	Illumina	ERX1936762	
AWU	G1	AWU_msDED	121611	589161920	TruSeq Synthetic Reads	Illumina	ERX1936765	
AWU	G1	AWU_msDEF	124691	592617439	TruSeq Synthetic Reads	Illumina	ERX1936766	
AWU	G1	AWU_msDEB	175574	750471312	TruSeq Synthetic Reads	Illumina	ERX1936763	
AWU	G1	AWU_msDEC	167087	731676445	TruSeq Synthetic Reads	Illumina	ERX1936764	
AWU	G1	AWU-msDBX	110436	530240247	TruSeq Synthetic Reads	Illumina	ERX1936760	

**RNAseq**							
AXF	AA	AXF_AAOSW_8_C0D1LACXX.IND2	59050722	11928245844	Paired-end # RNA	Illumina	ERX332625
AXF	BA	AXF_BAOSW_8_C0D1LACXX.IND4	63191029	12764587858	Paired-end # RNA	Illumina	ERX332624
AXF	CA	AXF_CAOSW_8_C0D1LACXX.IND5	68158203	13767957006	Paired-end # RNA	Illumina	ERX332621
AXF	DA	AXF_DAOSW_7_C0D1LACXX.IND6	72263408	14597208416	Paired-end # RNA	Illumina	ERX332626
AXF	EA	AXF_EAOSW_7_C0D1LACXX.IND7	57005112	11515032624	Paired-end # RNA	Illumina	ERX332623
AXF	FA	AXF_FAOSW_7_C0D1LACXX.IND12	65878896	13307536992	Paired-end # RNA	Illumina	ERX332622
BHC	AF	BHC_AFOSW_8_C4VAEACXX.IND12	29858750	5931520880	Paired-end # RNA	Illumina	ERX1916513
BHC	AG	BHC_AGOSW_7_C4VBLACXX.IND13	27938013	5587303314	Paired-end # RNA	Illumina	ERX1916512
BHC	BA	BHC_BAOSW_3_C4VR1ACXX.IND15	32668746	6486518369	Paired-end # RNA	Illumina	ERX1796981
BHC	BB	BHC_BBOSW_3_C4VR1ACXX.IND16	29920489	5929334824	Paired-end # RNA	Illumina	ERX1796984
BHC	BC	BHC_BCOSW_3_C4VR1ACXX.IND18	33368451	6625154612	Paired-end # RNA	Illumina	ERX1796982
BHC	BD	BHC_BDOSW_3_C4VR1ACXX.IND19	33982054	6753975751	Paired-end # RNA	Illumina	ERX1796983
BHD	AA	BHD_AAOSW_1_C3YEPACXX.IND1	29134976	5808377002	Paired-end # RNA	Illumina	ERX1796974
BHD	AB	BHD_ABOSW_1_C3YEPACXX.IND3	32355510	6448224478	Paired-end # RNA	Illumina	ERX1796976
BHD	AC	BHD_ACOSW_1_C3YEPACXX.IND8	43958162	8765695282	Paired-end # RNA	Illumina	ERX1796975
BHD	AK	BHD_AKOSW_2_C3YEPACXX.IND23	31082716	6204885589	Paired-end # RNA	Illumina	ERX1916511
BHD	AL	BHD_ALOSW_2_C3YEPACXX.IND25	27615039	5520361779	Paired-end # RNA	Illumina	ERX1916509
BHD	AM	BHD_AMOSW_2_C3YEPACXX.IND27	30157050	6022460185	Paired-end # RNA	Illumina	ERX1916510
BIG	G	BIG_GOSW_5_C49VTACXX.IND7	27305012	5442125902	Paired-end # RNA	Illumina	ERX1916514

Supplementary Table 2 Metrics of final haploid (haploid V2) and diploid (diploid V2) versions of the pedunculate oak genome sequence assembly.

	**Diploid V2 (Assembly A5[a])**	**Haploid V2 (Assembly H1[b])**
**Assembly**	Diploid	Haploid
**No. of sequences**	8,827	1,409
**Cumulative size**	1,455,104,916	814,282,569
**N50**	821,707	1,342,530
**N90**	198,501	333,129
**L50**	537	192
**L90**	1,880	649
**% of N's**	4.6	2.94
**Completeness using BUSCO**	210 (90.4%)	202 (90.8%)

[a] from **Supplementary Table 10**

[b] from **Supplementary Table 11**

Supplementary Table 3 Comparison of genome assemblies from available heterozygous trees. Best (green) and worst (red) assembly metrics, excluding *Poplulus trichocarpa*.

Species	Assembly availability	# contigs	Cumulative size of contigs (Mb)	Contigs N50 size	# scaffolds	Cumulative size of scaffolds (Mb)	% of N	Scaffold N50 size	Busco %C	Busco %D
Olea europaea	http://denovo.cnag.cat/genomes/olive/download/Oe6/Oe6.scaffolds.fa.gz	38,053	1,265	87,946	11,038	1,319	4.09	443,100	277 (91.4%)	126 (41.6%)
Quercus robur	This study	22,615	790	69,349	1,409	814	2.94	1,342,530	269 (88.8%)	49 (16.2%)
Betula pendula	https://genomevolution.org/coge/api/v1/genomes/35079/sequence	27,580	425	49,342	5,642	435	2.34	239,520	261 (86.1%)	38 (12.5%)
Fraxinus excelsior	http://www.ashgenome.org/assemblies	119,515	718	24,932	89,514	867	17.19	103,995	272 (89.8%)	97 (32.0%)
Castanea mollissima	https://hardwoodgenomics.org/chinese-chestnut-genome#genomedownloads	70,867	710	22,063	41,260	724	1.86	39,561	264 (87.1%)	50 (16.5%)
Quercus lobata	https://valleyoak.ucla.edu/genomicresources/	255,152	1,069	17,576	94,394	1,183	9.64	161,656	271 (89.4%)	98 (32.3%)
Populus trichocarpa	https://genomevolution.org/coge/api/v1/genomes/25127/sequence	8,313	423	552,806	1,446	434	2.57	19,465,461	279 (92.0%)	108 (35.6%)

Supplementary Table 4 Annotation of transposable elements.

		# TE consensus	# genome copies	Genome coverage (kb)	Genome coverage %	TE content coverage %
**Class I Retro-elements LTR**	**Copia**	211	89,447	87,215	11.04	20.71
	**Gypsy**	276	91,652	107,561	13.61	25.54
	**LARD/TRIM/Other**	80	38,726	29,058	3.68	6.90
**Class I Retro-elements non-LTR**	**LINE**	408	157,114	66,135	8.37	15.70
	**SINE**	30	4,571	1,216	0.15	0.29
**Class I**	**Other**	16	20,970	4,224	0.53	1.00
**Class II DNA transposons**	**TIR**	313	141,489	52,207	6.61	12,.39
	**MITE**	67	28,124	7,760	0.98	1.84
	**Helitron**	8	3,642	2,006	0.25	0.48
	**Other**	11	2,987	2,012	0.25	0.48
**Unknown**		317	134,652	54,428	7.27	13.63
**Endovirus**		13	2,818	4,385	0.55	1.04
**Total**		1,750	716,192	420,651	53.30[a]	100

[a] The total 53.3% of genome coverage reported here corresponds to the cumulative sum of coverage for the different orders/familes. Total TE genome coverage is 52%, without redundancy between copies.

Supplementary Table 5 List of control SNPs (C) and somatic mutations (SMs) detected in the "3P" pedunculate oak accession. C: control SNP, SM: somatic mutation, Mutation: reference /alternative (alt) allele, f(alt)pool: frequency of the alternative allele in the pool-seq data set.

Mutation category	Locus ID	Chromosomal location	Mutation	Origin of the mutation	f(alt)pool	f(alt)pool >0.5%
C	Sc0000093_652917	Chr1-41939193	T/A	within species	0.9697	
C	Sc0000158_1024005	Chr2-27130279	T/A	within species	0.9637	
C	Sc0000067_389965	Chr3-27491298	A/T	within species	0.9534	
C	Sc0000033_2516576	Chr4-10285224	A/C	within species	0.9585	
C	Sc0000505_233875	Chr5-39212547	T/G	within species	0.9507	
C	Sc0000170_1375115	Chr6-36333647	C/A	within species	0.9542	
C	Sc0000268_125122	Chr7-23637407	T/C	within species	0.9605	
C	Sc0000187_1162488	Chr8-53746285	T/C	within species	0.9700	
C	Sc0000168_672869	Chr9-31527061	C/T	within species	0.9541	
C	Sc0000447_97317	Chr10-22125827	A/G	within species	0.9526	
C	Sc0000099_1051673	Chr11-7530106	A/G	within species	0.9679	
C	Sc0000425_378736	Chr12-27206974	G/A	within species	0.9691	
SM	Sc0000080_1329750	Chr8-58757192	G/A	3P – between XL1 and XL2	0.0330	y
SM	Sc0000573_185294	Chr7-21324198	A/T	3P – between XL1 and XL2	0.0000	
SM	Sc0000010_1057132	Chr3-13766752	G/A	3P – between XL1 and XL2	0.1154	y
SM	Sc0000003_4011526	Chr2-84974261	A/T	3P – between XL1 and XL2	0.0000	
SM	Sc0000010_758473	Chr3-13468093	G/A	3P – between XL1 and XL2	0.0000	
SM	Sc0000015_2644541	Chr3-50836723	G/T	3P – between XL1 and XL2	0.0000	
SM	Sc0000057_1996281	Chr11-19272464	C/T	3P – between XL1 and XL2	0.0000	
SM	Sc0000235_409999	Chr12-5868120	C/T	3P – between XL1 and XL2	0.1384	y
SM	Sc0000122_532208	Chr1-8511503	C/T	3P – between XL1 and XL2	0.0000	
SM	Sc0000139_351870	Chr1-28576871	T/C	3P – between XL1 and XL2	0.0000	
SM	Sc0000233_840676	Chr7-12397418	G/A	3P – between XL1 and XL2	0.0000	
SM	Sc0000588_301268	Chr4-27781193	G/A	3P – between XL1 and XL2	0.0000	
SM	Sc0000065_545730	Chr5-66436424	T/C	3P – L1 branch	0.0000	
SM	Sc0000181_1118667	Chr2-50905345	C/T	3P – L1 branch	0.0782	y
SM	Sc0000200_640712	Chr5-54088853	G/A	3P – L1 branch	0.0000	
SM	Sc0000667_35498	Chr1-10565982	G/A	3P – L1 branch	0.0133	y
SM	Sc0000444_256472	Chr3-25184616	C/T	3P – L1 branch	0.0049	

SM	Sc0000277_447345	Chr2-113550344	T/C	3P – L1 branch	0.0000	
SM	Sc0000135_631742	Chr7-25169846	T/C	3P – L1 branch	0.0000	
SM	Sc0000001_4448299	Chr6-48948241	T/G	3P – L1 branch	0.0000	
SM	Sc0000219_286289	Chr2-49724208	A/T	3P – L1 branch	0.0000	
SM	Sc0000395_657452	Chr8-17311448	G/T	3P – L1 branch	0.0000	
SM	Sc0000099_1809337	Chr11-6772442	C/T	3P – L2 branch	0.0078	y
SM	Sc0000035_1061781	Chr1-32821443	C/T	3P – L2 branch	0.0043	
SM	Sc0000066_1207928	Chr2-22466474	G/A	3P – L2 branch	0.0000	
SM	Sc0000103_228814	unanchored scaffold	C/T	3P – L2 branch	0.0000	
SM	Sc0000578_47594	Chr2-70260112	C/T	3P – L2 branch	0.0000	
SM	Sc0000031_1042378	Chr9_36922447	C/T	3P – between XL1 and XL2	0.0035	
SM	Sc0000114_1570819	Chr4_39071460	A/G	3P – between XL1 and XL2	0.0000	
SM	Sc0000114_960640	Chr4_39681639	G/A	3P – between XL1 and XL2	0.0030	
SM	Sc0000146_1249018	Chr12_26296592	C/T	3P – between XL1 and XL2	0.0000	
SM	Sc0000975_67191	Chr2_23741692	T/C	3P – between XL1 and XL2	0.0000	
SM	Sc0000000_3201322	Chr1_23876533	G/A	3P – L1 branch	0.0000	
SM	Sc0000041_558993	Chr1_2895035	G/A	3P – L1 branch	0.0935	y
SM	Sc0000228_252981	Chr5_34619071	C/T	3P – L1 branch	0.0000	
SM	Sc0000277_395519	Chr2_113498518	G/A	3P – L1 branch	0.0608	y
SM	Sc0000570_213658	Chr8_40730574	C/T	3P – L1 branch	0.0244	y
SM	Sc0001123_18450	unanchored scaffold	G/A	3P – L1 branch	0.0270	y
SM	Sc0000002_4278465	Chr2_66564257	T/A	3P – L2 branch	0.0000	
SM	Sc0000005_165035	Chr11_33010956	T/G	3P – L2 branch	0.0000	
SM	Sc0000242_170918	Chr2_55024334	C/T	3P – L2 branch	0.0000	
SM	Sc0000312_31989	Chr6_28492838	A/T	3P – L2 branch	0.0000	
SM	Sc0000584_280071	unanchored scaffold	A/T	3P – L2 branch	0.3152	y
SM	Sc0000026_779464	unanchored scaffold	G/A	3P – between XL2 and L3	0.0359	y
SM	Sc0000027_691249	Chr2_98491575	A/G	3P – between XL2 and L3	0.0000	
SM	Sc0000042_876919	Chr4_19144163	T/A	3P – between XL2 and L3	0.0000	
SM	Sc0000051_786541	Chr2_30320278	C/T	3P – between XL2 and L3	0.0000	
SM	Sc0000056_626880	Chr5_18059797	C/T	3P – between XL2 and L3	0.0690	y
SM	Sc0000085_693443	Chr10_14911573	C/T	3P – between XL2 and L3	0.0140	y
SM	Sc0000097_1855202	Chr9_47939472	C/T	3P – between XL2 and L3	0.0000	
SM	Sc0000108_1664655	Chr10_27649124	C/T	3P – between XL2 and L3	0.0000	

SM	Sc0000132_1066473	unanchored scaffold	T/A	3P – between XL2 and L3	0.0000	
SM	Sc0000167_777615	Chr2_81379058	T/C	3P – between XL2 and L3	0.0029	
SM	Sc0000170_850309	Chr6_35808841	G/T	3P – between XL2 and L3	0.0000	
SM	Sc0000210_857733	Chr12_35930326	C/T	3P – between XL2 and L3	0.0000	
SM	Sc0000227_150153	Chr10_8928796	G/A	3P – between XL2 and L3	0.0000	
SM	Sc0000266_244617	Chr11_43001996	G/A	3P – between XL2 and L3	0.0000	
SM	Sc0000266_72243	Chr11_43174370	A/C	3P – between XL2 and L3	0.0000	
SM	Sc0000274_597070	Chr2_35105614	G/A	3P – between XL2 and L3	0.0305	y
SM	Sc0000300_540958	Chr5_55161003	C/T	3P – between XL2 and L3	0.0000	
SM	Sc0000620_260076	Chr11_26408896	C/T	3P – between XL2 and L3	0.0028	

Supplementary Table 6 List of control SNPs (C) and somatic mutations (SMs) in the offspring of accession "3P". Acorns of the reference genotype "3P" were collected from the L1 and L2 branches indicated in **Fig. 2b**. C: control SNP, SM somatic mutation, N: sample size with accurate genotypic information, H0: observed heterozygosity. f(pool): frequency of the alternative allele. A value of 0 in this last column indicates a mutation detected only in the reference "3P" genotype and transmitted to its offspring.

SNP Category	Locus ID	Origin of the mutation	Success of the assay	% missing data	N	H0	f(pool)
C	Chr1-41939193	within species	Y	0.440	65	0.400	0.9697
C	Chr2-27130279	within species	Y	0.853	17	0.588	0.9637
C	Chr3-27491298	within species	Y	0.276	84	0.595	0.9534
C	Chr4-10285224	within species	Y	0.819	21	0.619	0.9545
C	Chr5-39212547	within species	Y	0.466	62	0.306	0.9507
C	Chr6-36333647	within species	Y	0.905	11	0.455	0.9542
C	Chr7-23637407	within species	Y	0.138	100	0.820	0.9605
C	Chr8-53746285	within species	Y	0.259	86	0.814	0.9700
C	Chr9-31527061	within species	Y	0.198	93	0.215	0.9541
C	Chr10-22125827	within species	Y	0.888	13	0.231	0.9526
C	Chr11-7530106	within species	Y	0.914	10	0.600	0.9679
C	Chr12-27206974	within species	Y	0.172	96	0.875	0.9691
SM	Sc0000573_185294	3P – between XL1 and XL2	Y	0.172	96	0.000	0.000
SM	Sc0000003_4011526	3P – between XL1 and XL2	Y	0.284	83	0.084	0.000
SM	Sc0000010_758473	3P – between XL1 and XL2	Y	0.233	89	0.124	0.000
SM	Sc0000015_2644541	3P – between XL1 and XL2	Y	0.595	47	0.191	0.000
SM	Sc0000057_1996281	3P – between XL1 and XL2	Y	0.491	59	0.288	0.000
SM	Sc0000122_532208	3P – between XL1 and XL2	Y	0.871	15	0.000	0.000
SM	Sc0000139_351870	3P – between XL1 and XL2	Y	0.198	93	0.000	0.000
SM	Sc0000233_840676	3P – between XL1 and XL2	Y	0.026	113	0.000	0.000
SM	Sc0000588_301268	3P – between XL1 and XL2	Y	0.267	85	0.000	0.000
SM	Sc0000065_545730	3P – L1 branch	Y	0.836	19	0.000	0.000

SM	Sc0000200_640712	3P – L1 branch	Y	0.069	108	0.111	0.000
SM	Sc0000444_256472	3P – L1 branch	Y	0.034	112	0.018	0.005
SM	Sc0000277_447345	3P – L1 branch	Y	0.690	36	0.111	0.000
SM	Sc0000135_631742	3P – L1 branch	Y	0.595	47	0.000	0.000
SM	Sc0000001_4448299	3P – L1 branch	Y	0.629	43	0.000	0.000
SM	Sc0000219_286289	3P – L1 branch	N	NA	0	NA	0.000
SM	Sc0000395_657452	3P – L1 branch	N	NA	0	NA	0.000
SM	Sc0000035_1061781	3P – L2 branch	Y	0.276	84	0.000	0.004
SM	Sc0000066_1207928	3P – L2 branch	Y	0.190	94	0.000	0.000
SM	Sc0000103_228814	3P – L2 branch	Y	0.897	12	0.000	0.000
SM	Sc0000578_47594	3P – L2 branch	Y	0.026	113	0.000	0.000

Supplementary Table 7 Result of the OrthoMCL analysis and comparison between the 16 eudicot species used in this study.

Species acronym*	#genes	#orthogroups	#genes in orthogroups	#singletons	% Genes in orthogroups	#genes shared with at least one other species	%genes shared with at least one other species	#species specific orthogroups	#genes in species specific orthogroups
Al	32,657	17,186	27,260	5,397	0.83	24,449	0.75	813	2,811
At	27,416	16,716	24,733	2,683	0.90	24,334	0.89	141	399
Wa	23,440	12,775	20,192	3,248	0.86	17,402	0.74	364	2,790
Fv	32,831	14,304	25,093	7,738	0.76	19,953	0.61	1,275	5,140
Gm	56,044	15,235	46,400	9,644	0.83	41,978	0.75	1,552	4,422
Rc	31,220	14,658	21,088	10,132	0.68	18,913	0.61	754	2,175
St	35,119	13,041	28,897	6,222	0.82	21,825	0.62	1,060	7,072
Cp	27,584	13,483	20,285	7,299	0.74	18,334	0.66	520	1,951
Cc	24,533	13,916	21,425	3,108	0.87	20,497	0.84	316	928
Eg	36,376	13,615	29,063	7,313	0.80	26,402	0.73	722	2,661
Md	63,514	17,217	46,524	16,990	0.73	37,225	0.59	3,324	9,299
Pt	41,335	14,921	33,604	7,731	0.81	31,412	0.76	728	2,192
Pp	27,864	14,545	24,651	3,213	0.88	23,230	0.83	311	1,421
Qr	**25,808**	**11,813**	**22,498**	**3,310**	**0.87**	**20,761**	**0.80**	**479**	**1,737**
Tc	29,452	14,591	23,608	5,844	0.8	21,722	0.74	465	1,886
Vv	26,346	12,951	19,774	6,572	0.75	18,135	0.69	589	1,639
**Total #genes**	**541,539**		**435,095**	**106,444**		**386,572**			**48,523**

** Al Arabidopsis lyrata, At Arabidopsis thaliana, Wa Citrullus lanatus, Fv Fragaria vesca, Gm Glycine max, Rc Ricinus communis, St Solanum tuberosum, Cp Carica papaya, Cc Citrus climentina, Eg Eucalyptus grandis, Md Malus domestica, Pt Populus trichocarpa, Pp Prunus persica, Qr Quercus robur, Tc Theobroma cacao, Vv Vitis vinifera.*

Supplementary Table 8 Repertoire of NB-LRR-related disease resistance genes in oak. Genes are classified in different categories according to the presence of the canonical NB-ARC (NB), leucine-rich repeat (LRR) domains and/or the N-terminal domains typically associated with disease resistance NB-LRR genes in plant genomes, namely Toll interleukin receptor-like (TIR), coiled-coil (CC) and resistance to powdery mildew protein RPW8 (R) domains. X indicates the presence of a putative integrated domain (ID).

**Category**	**Acronym**	**Total**	**Integrated domains (X)**
CC-NB-LRR (X)	CNL	258	16
CC-NB (X)	CN	47	3
CC-LRR	CL	3	
CC (X)	C	14	1
NB-LRR-CC-NB-LRR	*NLCNL*	*1*	
CC(3x)-NB-LRR	*C(3x)NL*	*1*	
RPW8-NB-LRR (X)	RNL	15	3
RPW8	R	1	
TIR-NB-LRR (X)	TNL	186	11
TIR-NB (X)	TN	25	3
TIR-LRR (X)	TL	3	1
TIR	T	151	
NB-LRR (X)	NL	240	11
NB (X)	N	61	4
LRR (X)	L	85	1
**Total**		**1,091**	**54**

Supplementary Table 9 Genetic diversity (π) at 0-fold, 4-fold degeneracy and π_0/π_4 ratio. Estimates were averaged over 1,000 randomly picked genes in each category and repeated 100 times. Mean and 95% confidence intervals (in parentheses) are reported. Values should be multiplied by 10^{-3}.

	Total	Expanded	Contracted	Unchanged
**“3P” Genome sequence**				
**π_0**	5.0 (4.6, 5.5)	7.0 (6.6, 7.4)	3.2 (3.0, 3.4)	3.2 (3.0, 3.5)
**π_4**	11.4 (10.6, 12)	12.5 (11.7, 13.3)	10.8 (10.2, 11.4)	10 (9.2, 10.9)
**π_0/π_4**	0.44 (0.39, 0.49)	0.56 (0.52, 0.61)	0.3 (0.27, 0.32)	0.32 (0.29, 0.37)
**Pool-sequencing**				
**π_0**	5.4 (5.0, 5.7)	7.6 (7.2, 7.9)	2.9 (2.7, 3.0)	3 (2.8, 3.2)
**π_4**	10.8 (10.3, 11.3)	12.5 (12.0, 13.0)	9.5 (9.2, 9,9)	9.3 (8.9, 9.8)
**π_0/π_4**	0.5 (0.46, 0.53)	0.6 (0.57, 0.65)	0.3 (0.28, 0.32)	0.32 (0.3, 0.34)

Supplementary Table 10 Metrics of the pedunculate oak assembly at each step of the Newbler process.

	Newbler A1	Newbler A2	Newbler A3	Newbler A4	Newbler A5
Assembly step	Raw output	Graph simplification	Scaffolding	Gap closing	Contamination removal
# sequences	296,255	198,695	9,025	9,025	8,827
Cumulative size	1,313,577,586	1,330,866,990	1,455,541,024	1,458,028,538	1,455,104,916
N50	9,499	16,207	818,147	821,283	821,707
N90	1,800	3,322	538	194,343	198,501
L50	38,579	23,591	193,405	7538	537
L90	158,717	89,893	1,892	1,893	1,880
% of N’s	0	1.3	11.19	4.63	4.6

Supplementary Table 11 Metrics of pedunculate oak assembly at each step of the Celera process.

	Celera C1	Celera C2
Assembly step	Raw output	Scaffolding
No. of sequences	296,255	14,088
Cumulative size	1,313,577,586	1,273,117,594
N50	9,499	266,385
N90	1,800	55,257
L50	38,579	1,418
L90	158,717	5,257
% of N’s	0	9.24

Supplementary Table 12 Structural manual curation of mRNAs indicating the type of protein coding structure (CDS) curation.

**Total annotated mRNA in the v1 diploid assembly**	**1,714 genes**
**Validation without CDS curation**	1,347 (79%)
**Validation with CDS curation**	367 (21%)
• Exon (donor/acceptor,start/stop)	233
• Gene merge	93
• Gene split	0
• Other not specified	41

Supplementary Table 13 Description of the RNAseq libraries used to annotate non-coding RNA.

RNAseq library file	Tissues/environmental conditions	NCBI accessions
AXF_AAOSW_8_1.fastq.gz<br>AXF_AAOSW_8_2.fastq.gz	Ecodormant buds harvested from two adult trees in 2005 (2005.12.01)	ERP004204
AXF_BAOSW_8_1.fastq.gz<br>AXF_BAOSW_8_2.fastq.gz	Swelling buds harvested from two adult trees in 2006 (2006.24.03)	ERP004204
AXF_CAOSW_8_1.fastq.gz<br>AXF_CAOSW_8_2.fastq.gz	Differentiating xylem sampled in April 2004 from adult trees.	ERP004204
AXF_DAOSW_7_1.fastq.gz<br>AXF_DAOSW_7_2.fastq.gz	Roots harvested from 6-month-old seedlings after exposure to cold, heat, high CO_2 concentration, water stress and hypoxia.	ERP004204
AXF_EAOSW_7_1.fastq.gz<br>AXF_EAOSW_7_2.fastq.gz	Leaves harvested on 6 month old seedlings after exposure to cold, heat, high CO_2 concentration, water stress and hypoxia.	ERP004204
AXF_FAOSW_7_1.fastq.gz<br>AXF_FAOSW_7_2.fastq.gz	Dedifferentiated *in vitro* callus from genotype # DF 159	ERP004204
BHD_AAOSW_1_1.fastq.gz<br>BHD_AAOSW_1_2.fastq.gz	White roots harvested from five-week-old sessile oak seedlings. Pool of 10 seedlings.	ERA763633
BHD_ABOSW_1_1.fastq.gz<br>BHD_ABOSW_1_2.fastq.gz	White roots harvested from five-week-old sessile oak seedlings. Pool of 10 seedlings.	ERA763633
BHD_ACOSW_1_1.fastq.gz<br>BHD_ACOSW_1_2.fastq.gz	White roots harvested from five-week-old sessile oak seedlings. Pool of 10 seedlings.	ERA763633
BHC_BAOSW_3_1_C4VR1ACXX.IND15_noribo_clean.fastq.gz<br>BHC_BAOSW_3_2_C4VR1ACXX.IND15_noribo_clean.fastq.gz	Endodormant buds (sampled Oct. 2nd 2013: pool of 5 sessile oak genotypes from the Laveyron population in the Pyrenees)	ERA763635
BHC_BBOSW_3_1_C4VR1ACXX.IND16_noribo_clean.fastq.gz<br>BHC_BBOSW_3_2_C4VR1ACXX.IND16_noribo_clean.fastq.gz	Endodormant buds : (sampled Oct. 2nd 2013: pool of 5 other sessile oak genotypes from the Laveyron population in the Pyrenees)	ERA763635
BHC_BCOSW_3_1_C4VR1ACXX.IND18_noribo_clean.fastq.gz<br>BHC_BCOSW_3_2_C4VR1ACXX.IND18_noribo_clean.fastq.gz	Ecodormant buds : (sampled March 10th 2014:pool of 5 sessile oak genotypes from the Laveyron population in the Pyrenees)	ERA763635
BHC_BDOSW_3_1_C4VR1ACXX.IND19_noribo_clean.fastq.gz<br>BHC_BDOSW_3_2_C4VR1ACXX.IND19_noribo_clean.fastq.gz	Ecodormant buds : (sampled March 10th 2014: pool of 5 other sessile oak genotypes from the Laveyron population in the Pyrenees)	ERA763635

Supplementary Table 14 Description of the miRNAseq libraries used to identify and validate miRNAs (NCBI bioproject accession: PRJNA361225).

**miRNAseq library file (2 replicates)**	**Sample type**			**NCBI accessions**
	Elevation (m)	Location/ valley/sampling date	Bud dormancy stage /genotypes pooled for library construction	
**A1-Endo.fastq.gz**<br>**A2-Endo.fastq.gz**	1,600	Artouste/<br>Ossau/Oct. 7th 2013	Endodormancy<br>A1<br>A2	 SRR5181470<br>SRR5181469
**A1-Eco.fastq.gz**<br>**A2-Eco.fastq.gz**	1,600	Artouste/<br>Ossau/April 7th 2014	Ecodormancy<br>A1<br>A2	 SRR5181464<br>SRR5181463
**LH1-Endo.fastq.gz**<br>**LH2-Endo.fastq.gz**	800	Le Hourque/<br>Ossau/ Oct. 6th 2013	Endodormancy<br>LH1<br>LH2	 SRR5181472<br>SRR5181471
**LH1-Eco.fastq.gz**<br>**LH2-Eco.fastq.gz**	800	Le Hourque/<br>Ossau/ March 16th 2014	Ecodormancy<br>LH1<br>LH2	 SRR5181466<br>SRR5181465
**J1-Endo.fastq.gz**<br>**J2-Endo.fastq.gz**	100	Josbaig/<br>Ossau/ Oct. 5th 2013	Endodormancy<br>J1<br>J2	 SRR5181474<br>SRR5181473
**J1-Eco.fastq.gz**<br>**J2-Eco.fastq.gz**	100	Josbaig/<br>Ossau/ March 10th 2014	Ecodormancy<br>J1<br>J2	 SRR5181468<br>SRR5181467
**PR1-Endo.fastq.gz**<br>**PR2-Endo.fastq.gz**	1,600	Péguère/<br>Luz/ Oct. 4th 2013	Endodormancy<br>PR1<br>PR2	 SRR5181458<br>SRR5181457
**PR1-Eco.fastq.gz**<br>**PR2-Eco.fastq.gz**	1,600	Péguère/<br>Luz/April 8th 2014	Ecodormancy<br>PR1<br>PR2	 SRR5181452<br>SRR5181451
**P1-Endo.fastq.gz**<br>**P2-Endo.fastq.gz**	800	Papillon/<br>Luz/ Oct. 3rd 2013	Endodormancy<br>P1<br>P2	 SRR5181460<br>SRR5181459
**P1-Eco.fastq.gz**<br>**P2-Eco.fastq.gz**	800	Papillon/<br>Luz/March 17th 2014	Ecodormancy<br>P1<br>P2	 SRR5181454<br>SRR5181453
**L1-Endo.fastq.gz**<br>**L2-Endo.fastq.gz**	100	Laveyron/<br>Luz/ Oct. 2nd 2013	Endodormancy<br>L1<br>L2	 SRR5181462<br>SRR5181461
**L1-Eco.fastq.gz**<br>**L2-Eco.fastq.gz**	100	Laveyron/<br>Luz/March 13th 2014	Ecodormancy<br>L1<br>L2	 SRR5181456<br>SRR5181455

Supplementary Table 15 Number of predicted and annotated ncRNA loci.

**Family/sub-family**		**Software**		**#Unique**
**rRNA**	**#RNAmmer predictions**	**#cmsearch predictions**	**#Overlapping loci**	**#Unique**
**rRNA**				**136**
5S	49	65	44	70
LSU/5.8S	13	14	7	22
SSU	20	52	20	44
**tRNA**	**#tRNAscan-SE predictions**	**#cmsearch predictions**	**#Overlapping loci**	
**tRNA**	827	815	790	852
**miRNA**	**#sRNA-PlAn predictions annotated as miRNA**	**#cmsearch predictions**	**#Overlapping loci**	
**miRNA**	1508	204	59	1594
**Others**	-	**#cmsearch predictions**		
**SnoRNA**				**486**
C/D	-	412	-	412
H/ACA	-	74	-	74
**SnRNA**	-		-	**225**
U1	-	34	-	34
U11	-	1	-	1
U2	-	55	-	55
U12	-	1	-	1
U4	-	33	-	33
U5	-	24	-	24
U6	-	64	-	64
U6atac	-	13	-	13
**RnaseMRP**	-	2	-	2
**RNaseSRP**	-	31	-	31

Supplementary Table 16 Distribution of the various non-coding element categories for small RNAseq data.

Non-coding elements	% aligned reads
Predicted ncRNA (P)	41.0%
LncRNA (L)	25.5%
Transposon elements (T)	38.3%
Total (P+L+T)	72.4%

Supplementary Table 17 Geographical location of the natural stands from which the pedunculate oak genotypes were sampled for pool sequencing.

**Site name:**	ISS Landes
**Country:**	France
**Latitude/Longitude:**	001°05' W / 44°13' N
**Elevation:**	46m
**Total area:**	25,600ha
**Ecosystem:**	Intensively managed
**Tree species:**	*Alnus, Betula, Castanea, Corylus, Crataegus, Fagus, Fraxinus, Pinus, Prunus, Quercus, Salix, Sorbus*
**Land ownership:**	Mainly private
**Protection:**	Includes Natura 2000 sites

Supplementary Table 18 List of selected pedunculate oak genotypes used for pool sequencing.

Tree ID	Circumference (in cm at breast height)	Longitude (degrees, minutes seconds)	Latitude (degrees, minutes seconds)	Longitude (decimal format)	Latitude (decimal format)
74	139	-1.03129337	44.1717826	-1.053592703	44.288285056
352	83	-1.10264801	44.1348514	-1.174022236	44.230142753
357	156	-1.10195197	44.1347728	-1.172088818	44.229924535
358	162	-1.10178383	44.1347546	-1.171621740	44.229873982
501	137	-1.07406943	44.1248027	-1.127970645	44.213340929
521	92	-1.04406161	44.1252738	-1.077948924	44.214649407
523	137	-1.04439618	44.1252668	-1.078878276	44.214629927
602	59	-1.09019065	44.1139846	-1.150529574	44.194401571
607	206	-1.09013664	44.1145476	-1.150379563	44.195965590
1106	190	-1.05490144	44.1353328	-1.096948441	44.231480098
1108	310	-1.05472739	44.1349324	-1.096464983	44.230367702
1135	169	-1.02262027	44.1347846	-1.040611864	44.229957358
1136	138	-1.02280321	44.1346933	-1.041120023	44.229703499
1152	96	-1.00381904	44.1334381	-1.010608455	44.226216872
1153	210	-1.00406239	44.1334619	-1.011284422	44.226283131
1345	264	-1.07068438	44.1436749	-1.118567729	44.243541419
1361	255	-1.053003	44.134922	-1.091675153	44.2303391
1366	269	-1.053368	44.134774	-1.092688243	44.22992846
1410	260	-1.06359865	44.1032791	-1.109996257	44.175775217
1415	413	-1.063471	44.103621	-1.109641898	44.17672361

Supplementary Table 19 List of libraries for each of the three levels (L1, L2, L3) and number of sequences used for somatic mutation detection.

Tree Level	Libray ID _ run ID	NCBI Accession	Read length (before trimming)	#Raw reads	Total length (after trimming)	Mean read length (after trimming)
L1	BBX_AOSW_1_1_D1D53ACXX.IND5		101	178225679	17503027812	98.20710411
L1	BBX_AOSW_1_2_D1D53ACXX.IND5	ERX697294	101	178225679	17201970745	96.51791393
L1	BBX_AOSW_1_1_H32GMBCXX.IND5		251	129340482	31344486178	242.3408796
L1	BBX_AOSW_1_2_H32GMBCXX.IND5	ERX1886616	251	129340482	30061956368	232.4249601
L1	BBX_AOSW_2_1_H32GMBCXX.IND5		251	132162999	32027294956	242.3317812
L1	BBX_AOSW_2_2_H32GMBCXX.IND5	ERX1886622	251	132162999	30994003415	234.5134693
L1	BBX_AOSW_1_2_H57N7BCXX.IND5		251	114968046	26394294031	229.5793914
L1	BBX_AOSW_1_1_H57N7BCXX.IND5	ERX1886621	251	114968046	27764301183	241.4958082
L2	BBX_COSW_2_1_D1D53ACXX.IND7		101	187527862	18297743615	97.57346679
L2	BBX_COSW_2_2_D1D53ACXX.IND7	ERX697297	101	187527862	17809414834	94.96943358
L2	BBX_COSW_1_1_H072TAMXX.IND7		251	88896954	21647068232	243.5074236
L2	BBX_COSW_1_2_H072TAMXX.IND7	ERX697298	251	88896954	20874023496	234.8114593
L2	BBX_COSW_2_1_H072TAMXX.IND7		251	89768432	21850638929	243.4111685
L2	BBX_COSW_2_2_H072TAMXX.IND7	ERX697296	251	89768432	21059108981	234.5937042
L2	BBX_COSW_2_1_H57N7BCXX.IND7		251	141600651	34063995611	240.5638348
L2	BBX_COSW_2_2_H57N7BCXX.IND7	ERX1886620	251	141600651	32276725298	227.9419273
L3	BBX_EOSW_3_1_D1D53ACXX.IND9		101	185076013	18128331412	97.9507345
L3	BBX_EOSW_3_2_D1D53ACXX.IND9	ERX697292	101	185076013	17846097116	96.42577029
L3	BBX_EOSW_2_1_H32GLBCXX.IND9		251	134000800	28711601308	214.2644022
L3	BBX_EOSW_2_2_H32GLBCXX.IND9	ERX1886619	251	134000800	26474644233	197.5707924
L3	BBX_EOSW_1_1_H55MLBCXX.IND9		251	127908274	30449513248	238.0574164
L3	BBX_EOSW_1_2_H55MLBCXX.IND9	ERX1886617	251	127908274	28875401387	225.7508485
L3	BBX_EOSW_2_1_H55MLBCXX.IND9		251	104846648	24974555304	238.2007988
L3	BBX_EOSW_2_2_H55MLBCXX.IND9	ERX1886618	251	104846648	23191160974	221.1912485

Supplementary Table 20 MuTect comparisons indicating whether candidate SNPs are expected to be detected or not, depending on the age of the mutation. L1, L2, L3 = end of selected branches; X_{L1} and X_{L2} = L1-branch and L2-branch initiation sites (see also Fig. 2b).

		Colored tree section in Fig. 2b	MuTect comparisons (reference vs. potentially mutated libraries)					
			L1 vs. L2	L1 vs. L3	L2 vs. L1	L2 vs. L3	L3 vs. L1	L3 vs. L2
**Mutations occurring between levels:**	$X_{L1} – X_{L2}$	blue	X	X	Ø	Ø	Ø	Ø
	X_{L2} – L3	pink	Ø	X	Ø	X	Ø	Ø
	X_{L1} – L1	green	Ø	Ø	X	Ø	X	Ø
	X_{L2} – L2	yellow	X	Ø	Ø	Ø	Ø	X

Supplementary Table 21 List of the 15 eudicot plant genomes selected for the evolutionary analysis. Growth habit or lifespan (W: woody perennials vs. H: annual herbaceous species) is indicated in the last column.

Scientific name	Common name	# of genes	Assembly version	Order	Family	Genus	Growth habit
Arabidopsis lyrata	Lyrate rockcress	32,657	v1.0	Brassicales	Brassicaceae	*Arabidopsis*	H
Arabidopsis thaliana	Thale cress	27,416	TAIR10	Brassicales	Brassicaceae	*Arabidopsis*	H
Citrullus lanatus	Watermelon	23,440	v1	Cucurbitales	Cucurbitaceae	*Citrullus*	H
Fragaria vesca	Strawberry	32,831	v1.1	Rosales	Rosaceae	*Fragaria*	H
Glycine max	Soybean	56,044	Wm82.a2.v1	Fabales	Fabaceae	*Glycine*	H
Ricinus communis	Castorbean	31,221	v0.1	Malpighiales	Euphorbiaceae	*Ricinus*	H
Solanum tuberosum	Potato	35,119	v3.4	Solanales	Solanaceae	*Solanum*	H
Carica papaya	Papaya	27,584	ASGPBv0.4	Brassicales	Caricaceae	*Carica*	W
Citrus clementina	Clementine	24,533	v1.0	Sapindales	Rutaceae	*Citrus*	W
Eucalyptus grandis	Eucalyptus	36,376	v2.0	Myrtales	Myrtaceae	*Eucalyptus*	W
Malus domestica	Apple	63,514	v1.0	Rosales	Rosaceae	*Malus*	W
Populus trichocarpa	Poplar	41,335	v3.0	Malpighiales	Salicaceae	*Populus*	W
Prunus persica	Peach	27,864	v2.1	Rosales	Rosaceae	*Prunus*	W
Theobroma cacao	Cocoa	29,452	v1.1	Malvales	Malvaceae	*Theobroma*	W
Vitis vinifera	Grape	26,346	Genoscope_12X	Vitales	Vitaceae	*Vitis*	W

Supplementary Table 22 Contribution of gene models for the 16 studied species to orthoMCL orthogroups.

Growth habit	Species	Abbreviation	Genes in orthogroups				# orthogroups without gene (%)		#species-specific orthogroups (%)	
			Total	Mean	SD	Max				
Herbaceous species	*Arabidopsis lyrata*	Al	27,260	0.74	1.67	96	19,658	(49.8)	813	(2.1)
	Arabidopsis thaliana	At	24,733	0.67	1.56	124	20,128	(51.0)	141	(0.4)
	Citrullus lanatus	Wa	20,192	0.55	2.66	363	24,069	(61.0)	364	(0.9)
	Fragaria vesca	Fv	25,093	0.68	2.31	167	22,540	(57.2)	1,275	(3.2)
	Glycine max	Gm	46,400	1.26	3.68	295	21,609	(54.8)	1,552	(3.9)
	Ricinus communis	Rc	21,088	0.57	1.23	79	22,186	(56.3)	754	(1.9)
	Solanum tuberosum	St	28,897	0.78	7.01	1062	23,803	(60.4)	1,060	(2.7)
Woody perennials	*Carica papaya*	Cp	20,285	0.55	2.63	395	23,361	(59.2)	520	(1.3)
	Citrus climentina	Cc	21,425	0.58	1.95	130	22,928	(58.1)	316	(0.8)
	Eucalyptus grandis	Eg	29,063	0.79	3.59	228	23,229	(58.9)	722	(1.8)
	Malus domestica	Md	46,524	1.26	4.04	378	19,627	(49.8)	3,324	(8.4)
	Populus trichocarpa	Pt	33,604	0.91	2.75	183	21,923	(55.6)	728	(1.8)
	Prunus persica	Pp	24,651	0.67	5.37	907	22,299	(56.5)	311	(0.8)
	Quercus robur	**Qr**	**22,498**	**0.61**	**3.31**	**359**	**25,031**	**(63.5)**	**479**	**(1.2)**
	Theobroma cacao	Tc	23,608	0.64	2.46	208	22,253	(56.4)	465	(1.2)
	Vitis vinifera	Vv	19,774	0.54	1.49	105	23,893	(60.6)	589	(1.5)

Supplementary Table 23 Major family of repetitive elements identified by RepeatMasker within the sequenced BAC clones.

Family	Length (bp)	%
**DNA transposon**	**289,344**	**36**
hAT	54,917	6
EnSpm/CACTA	38,913	4
MuDR	36,662	4
Helitron	35,283	4
Harbinger	17,583	2
Polinton	17,078	2
Mariner/Tc1	12,792	1
**Retrotransposon**	**504,226**	**63**
-LTR Retrotransposon	361,480	45
Gypsy	222,020	27
Copia	126,984	15
-Non-LTR Retrotransposon	142,746	17
**Total length of repeats**	794,208	

Supplementary Table 24 Oak BAC sequence statistics.

**Total sequence length**	4,344,182 bp
**Sequence length excluding stretches of Ns**	4,282,332 bp (number of Ns: 61,850)
**GC content %**	35.9
**Number of predicted protein coding genes**	198[1], 50[2], 30[3]
**Number of predicted protein coding genes with homology to oak unigene[4]**	198
**tRNA genes**	4
**Gene density**	6 genes/100 kb
**Mean gene length**	4,028 bp[5]
**Mean number of exons per gene**	5.4
**Mean exon length**	232 bp
**% of genes with introns**	83.5
**Average intron length**	615 bp

[1] Approved: gene structure was modified or validated after manual curation.
[2] Problematic: gene structure remains after manual curation.
[3] deleted
[4] from Lesur et al.[23]
[5] UTRs were not considered.

Supplementary Table 25 Summary of overlapping regions between allelic BACs. BAC1 and BAC2 referred to pairs of allelic BACs.

BAC 1	BAC 2	% of BAC 1 covered	% identity	E-value	Range of overlap BAC 1 (bp)	Length of overlapping region_BAC 1 (bp)	Range of overlap BAC 2	Length of overlapping region_BAC 2 (bp)
50E24	177A20	44	98	0.0	74,218-140,871	66,655	21,871-75,811	53,940
5E10	107I07	43	97	0.0	34-52,488	52,454	11,315-86,163	74,848
12J1	121F17	50	97	0.0	3,328-69,071	65,743	1-107,378	107,378
27L03	48K1	27	97	0.0	72-22,592	22,520	94,896-110,662	25,766
64H3	30P1	55	99	0.0	11,197-105,888	94,691	1-87,454	87,454

Supplementary Table 26 Results of BLAST-n alignment (Evalue=0 and identity >95%) between overlapping BAC regions. BAC1 and BAC2 are pairs of allelic BAC.

BAC 1 (start-end)	BAC 2 (start-end)	% identity	E-value
50E24 (82472-97166)	177A20 (101617-86956)	98.313	0.0
50E24 (97342-98711)	177A20 (86962-85573)	97.557	0.0
50E24 (99445-102142)	177A20 (85526-82785)	95.796	0.0
50E24 (102139-102632)	177A20 (77962-77469)	98.178	0.0
50E24 (102630-104811)	177A20 (72280-70100)	99.313	0.0
50E24 (104964-113458)	177A20 (67500-59008)	97.533	0.0
50E24 (115554-119919)	177A20 (59015-54685)	93.276	0.0
50E24 (120139-121173)	177A20 (54688-53673)	88.509	0.0
50E24 (121653-122102)	177A20 (43131-42688)	95.778	0.0
50E24 (122088-130991)	177A20 (42426-33477)	96.247	0.0
50E24 (130983-137845)	177A20 (31276-24398)	95.750	0.0
50E24 (138678-140871)	177A20 (24080-21871)	97.473	0.0
5E10 (34-13493)	107I07 (86163-72751)	96.690	0.0
5E10 (6111-8673)	107I07 (96063-93431)	89.234	0.0
5E10 (15109-16637)	107I07 (72758-71221)	95.596	0.0
5E10 (16633-19460)	107I07 (60951-58146)	95.046	0.0
5E10 (19562-20049)	107I07 (58152-57640)	93.177	0.0
5E10 (20042-25226)	107I07 (57568-52375)	97.546	0.0
5E10 (20099-30015)	107I07 (48048-38134)	95.357	0.0
5E10 (30710-44385)	107I07 (37408-23814)	95.014	0.0
5E10 (44376-47570)	107I07 (18423-15229)	97.444	0.0
5E10 (47654-51561)	107I07 (15245-11315)	95.392	0.0
5E10 (51556-52488)	107I07 (929-1)	98.178	0.0
12J1 (3328-9386)	121F17 (107378-101370)	97.776	0.0
12J1 (10865-15722)	121F17 (101374-96618)	95.163	0.0
12J1 (15714-16429)	121F17 (96088-95351)	92.473	0.0
12J1 (17136-17988)	121F17 (94236-93385)	97.541	0.0
12J1 (17555-25498)	121F17 (92576-84615)	97.074	0.0
12J1 (25929-36992)	121F17 (67374-56308)	97.545	0.0
12J1 (27681-28891)	121F17 (111037-109862)	94.403	0.0
12J1 (40309-55775)	121F17 (51089-35673)	97.417	0.0
12J1 (57057-65797)	121F17 (12263-3515)	97.174	0.0
12J1 (65796-69071)	121F17 (3253-1)	97.063	0.0
27L03 (72-1944)	48K1 (94896-96762)	97.340	0.0
27L03 (1937-6237)	48K1 (97857-102122)	97.846	0.0
27L03 (6914-13853)	48K1 (102111-109054)	97.113	0.0
27L03 (14881-16542)	48K1 (109051-110662)	92.123	0.0
27L03 (15395-16915)	48K1 (70312-71817)	90.582	0.0
27L03 (20326-20919)	48K1 (95799-96389)	93.311	0.0
27L03 (22071-22592)	48K1 (102497-103027)	90.038	0.0
64H3 (11197-22071)	30P1 (87454-76608)	98.232	0.0
64H3 (27384-29525)	30P1 (71127-68965)	98.661	0.0

64H3 (29522-35146)	30P1 (61773-67407)	96.079	0.0
64H3 (35355-37869)	30P1 (61236-58733)	98.648	0.0
64H3 (46894-49574)	30P1 (50014-47328)	97.993	0.0
64H3 (60365-80565)	30P1 (46359-26158)	99.975	0.0
64H3 (83318-94716)	30P1 (17599-6207)	99.073	0.0
64H3 (94715-97846)	30P1 (4554-1423)	100.000	0.0
64H3 (99674-100979)	30P1 (1322-1)	95.925	0.0

Supplementary Table 27 Metrics of the previous release (V1) and current release (V2) of the oak diploid genome assembly.

	Diploid V1[b]	Diploid V2
**Assembly**	454 + Illumina	454 + Illumina + Synthetic Long Reads
**No. of sequences**	17,910	8,827
**Cumulative size**	1,354,311,717	1,455,104,916
**N50**	256,640	821,707
**N90**	35,065	198,501
**L50**	1,468	537
**L90**	6,626	1,880
**% of N's**	11.56	4.6
**Completeness using BUSCO**	274 (90.4%)	275 (90.8%)
**Oak RNA-seq genes (90,786 contigs)[a]**	86,457 (95.2%)	86,488 (95.3%)

[a] from Lesur et al. [23], [b] from Plomion et al. [19]

Supplementary Table 28 Classification of marker-scaffold relationships into four categories. The number of markers and the number of scaffolds within each category are provided.

Scaffold-marker relationship	Comment	Number of markers/2,615	Number of scaffolds (cumulative size Mb)
Category#1	Scaffold anchored with a single marker	165	165 (90.8)
Category#2	Scaffold anchored with at least 2 markers from the same LG	1412	331 (320)
Category#3	Scaffold anchored with more than 50% of the markers from the same LG	898	116 (214)
Category#4	Scaffolds (unassigned) with less than 50% of the markers from the same LG	140	116 (46.7)

Supplementary Table 29 Rank correlations (rho) between genetic and physical positions along the 12 chromosomes. LG: linkage group.

LG	size (cM)	No. of markers	#markers on chromosomes	Chr_start (bp)	Chr_end (bp)	LG_start (cM)	LG_end (cM)	rho
**1**	66.43	421	320	223,913	55,067,536	0.64	66.43	0.998
**2**	103.92	922	676	79,368	115,173,360	0.03	103.92	0.999
**3**	75.98	400	281	244,065	57,437,871	6.3	75.98	0.998
**4**	75.7	291	171	339,968	44,508,357	3.42	75.42	0.994
**5**	85.84	398	263	90,378	70,598,779	0.61	85.41	0.998
**6**	74.87	537	409	201,326	55,995,377	0.64	74.87	0.998
**7**	65.26	419	321	75,105	51,549,230	1.8	63.5	0.998
**8**	70.8	572	459	105,078	71,279,127	1.86	70.2	0.998
**9**	68.7	400	273	118,866	50,074,090	0.94	68.7	0.996
**10**	66.8	381	284	332,011	50,211,705	0.62	66.8	0.998
**11**	66.46	391	316	451,420	51,991,272	1.08	66.46	0.991
**12**	66.82	457	297	677	39,751,979	0.29	66.82	0.996
**Total**	887.58	5,589	4,070		711,376,508		866.28	0.997

Supplementary Table 30 Transposable element annotation: comparison between the 16 eudicot species used in this study.

Scientific name	Woody/ Herbaceous	Common name	Ref.	Assembly length annotated (Mb)	TE (Mb)	TE %	LTR % TE	Non-LTR % TE	Other class I % TE	Class I % TE	Class II % TE	Other % TE
Arabidopsis lyrata	H	*Arabidopsis lyrata*	[38]	207	61	29.7	64	8	0	72	25	3
Arabidopsis thaliana	H	Thale cress	[38]	135	32	23.7	50	28	0	78	6	16
Citrullus lanatus	H	Watermelon	[153]	354	160	45.2	Major	NA	NA	Major	NA	NA
Fragaria vesca	H	Strawberry	[154]	209	48	22.81	69.8	2	0	71.8	28.2	0
Glycine max	H	Soybean	[155]	955	561	58.7	71.5	0.4	0	71.9	28.1	0
Ricinus communis	H	Castor bean	[156]	350	176	50.3	32.2	0.3	3.6	36.1	1.8	62.1
Solanum tuberosum	H	Potato	[157]	727	452	62.2	45.93	4.51	0	50.4	6.2	43.4
Carica papaya	W	Papaya	[42]	815	423	51.9	77		0	77	0.2	22.8
Citrus clementina	W	Clementine	[43]	816	347	42.5	47	2.9	0	49.9	6.3	43.8
Eucalyptus grandis	W	Eucalyptus	[41]	817	409	50	73	7	5	85	11	4
Malus domestica	W	Apple	[40]	818	347	42.4	73.4	15.3	0	88.7	2.1	9.2
Populus trichocarpa	W	Poplar	[158]	820	362	44.2	18.6	1.4	0.1	20.1	6.1	73.8
Prunus persica	W	Peach	[39]	226	67	29.6	66.1	2.1	1.2	69.4	30.6	0
Theobroma cacao	W	Cocoa bean	[159]	346	144	41.5	77.9	0.4	0	78.3	21.7	0
Vitis vinifera	W	Grape	[160]	467	193	41.4	55.9	9.5	1.3	66.7	2	31.3
Quercus robur	**W**	**Oak**	this study	**814**	**421**	**52**	**53.1**	**16**	**1**	**70.1**	**15.2**	**14.7**

Supplementary Table 31 Oak gene structure statistics.

**Total protein coding genes**	25,808
**Gene space (Mb)**	75
**Gene density (# genes / 10 kb)**	0.32
**Gene mean / median (bp)**	2,907
**Gene median (bp)**	2,137
**CDS mean (bp)**	1,174
**CDS median (bp)**	942
**#CDS < 500 bp**	4,367
**#CDS > 3 kb**	1,162
**Genes with introns (%)**	79%
**#Introns/gene (mean)**	3.3

Supplementary Table 32 Horizontal transfers of LTR retrotransposons between oak and other plant species.

**Name of the LTR-retrotransposon family**	**Species involved in the transfer**
RLX-incomp_Qrob_v2_More29k-B-R2774-Map5_reversed	oak / grapevine
RLX-incomp-chim_Qrob_v2_More29k-B-R25479-Map5_reversed	oak / grapevine
RLX-incomp-chim_Qrob_v2_More29k-B-R32795-Map5_reversed	oak / grapevine
RLX-comp_Qrob_v2_More29k-B-G5453-Map6_reversed	oak / grapevine
RLX-comp_Qrob_v2_More29k-B-P1015.803-Map7	oak / grapevine
RLX-incomp_Qrob_v2_More29k-B-R289-Map20_reversed	oak / grapevine
RLX-comp_Qrob_v2_More29k-B-R13571-Map19	oak / poplar
RLX-incomp_Qrob_v2_More29k-B-R2774-Map5_reversed	oak / grapevine / peach tree

Supplementary Table 33 List of putative aquaporins identified in the pedunculate oak genome (haplome assembly). Number of exons and protein length (in amino-acids) are given. The aromatic/arginine selectivity filter (H, transmembrane helix and LE, loop E), the NPA motifs (LB, loop B and LE, loop E) and the five Froger's positions were identified from multiple sequence alignments.

			Ar/R filter				NPA motif		Froger's position						
**gene model ID**	**N° exon**	**protein (AA)**	**H2**	**H5**	**LE1**	**LE2**	**LB**	**LE**	**P1**	**P2**	**P3**	**P4**	**P5**	**subclass**	**remarks**
Qrob_T0687390.2	5	277	W	V	A	R	NPA	NPA	F	S	A	Y	M	NIP1	
Qrob_T0687410.2	5	289	W	V	A	R	NPS	NPA	F	T	A	Y	M	NIP1	
Qrob_T0405130.2	5	261	W	V	A	R	NPA	NPA	F	S	A	Y	I	NIP4	GC at ex/intron boundary
Qrob_T0144140.2	5	273	W	V	A	R	NPA	NPA	F	S	A	Y	V	NIP4	
Qrob_T0275880.2 ([1])	5	268	W	V	A	R	NPA	NPA	F	S	A	Y	V	NIP4	
Qrob_T0748200.2 ([2])	4	298	A	I	G	R	NPS	NPV	F	T	A	F	L	NIP5	
Qrob_T0118430.2	5	304	T	I	G	R	NPA	NPV	F	T	A	Y	M	NIP6	
Qrob_T0697150.2	5	282	A	V	G	R	NPA	NPA	Y	S	A	Y	V	NIP7	
Qrob_T0345370.2 ([*])	4	285	F	H	T	R	NPA	NPA	G	S	A	F	W	PIP1	
Qrob_T0236650.2	4	289	F	H	T	R	NPA	NPA	E	S	A	F	W	PIP1	
Qrob_T0705530.2	4	286	F	H	T	R	NPA	NPA	E	S	A	F	W	PIP1	
Qrob_T0348530.2	4	287	F	H	T	R	NPA	NPA	Q	S	A	F	W	PIP1	
Qrob_T0373060.2	4	278	F	H	T	R	NPA	NPA	M	S	A	F	W	PIP2	
Qrob_T0438960.2	4	262	F	H	T	R	NPA	NPA	Q	S	A	F	W	PIP2	GC at ex/intron boundary

Qrob_T0438980.2	4	262	F	H	T	R	NPA	NPA	Q	S	A	F	W	PIP2	GC at ex/intron boundary
Qrob_T0438970.2	4	262	F	H	T	R	NPA	NPA	Q	S	A	F	W	PIP2	GC at ex/intron boundary
Qrob_T0438950.2	4	262	F	H	T	R	NPA	NPA	Q	S	A	F	W	PIP2	GC at ex/intron boundary
Qrob_T0438990.2	4	286	F	H	T	R	NPA	NPA	Q	S	A	F	W	PIP2	
Qrob_T0602100.2	4	287	F	H	T	R	NPA	NPA	Q	S	A	F	W	PIP2	
Qrob_T0530060.2	4	281	F	H	T	R	NPA	NPA	Q	S	A	F	W	PIP2	
Qrob_T0131450.2	4	281	F	H	T	R	NPA	NPA	A	S	A	F	W	PIP2	
Qrob_T0602110.2	4	285	F	H	T	R	NPA	NPA	Q	S	A	F	W	PIP2	
Qrob_T0237440.2	1	239	A	V	P	N	NPS	NPA	P	A	A	Y	W	SIP	
Qrob_T0714870.2	3	241	I	M	P	N	NPT	NPA	P	A	A	Y	W	SIP	
Qrob_T0098460.2	3	237	S	H	G	S	NPL	NPA	P	V	A	Y	W	SIP	
Qrob_T0108440.2	3	252	H	I	A	V	NPA	NPA	T	S	A	Y	W	TIP1	
Qrob_T0398210.2	3	252	H	I	A	V	NPA	NPA	T	S	A	Y	W	TIP1	
Qrob_T0119780.2	2	251	H	I	A	V	NPA	NPA	T	S	A	Y	W	TIP1	
Qrob_T0656320.2	2	253	H	I	A	V	NPA	NPA	T	S	A	Y	W	TIP1	
Qrob_T0412470.2	3	248	H	I	G	R	NPA	NPA	T	S	A	Y	W	TIP2	
Qrob_T0538460.2	3	250	H	I	G	R	NPA	NPA	T	S	A	Y	W	TIP2	
Qrob_T0264600.2	3	246	H	I	A	R	NPA	NPA	T	S	A	Y	W	TIP4	
Qrob_T0082220.2 (*)	3	234	H	I	A	R	NPA	NPA	T	S	A	Y	W	TIP4	T deletion -> variant protein
Qrob_T0375680.2	3	254	N	V	G	C	NPA	NPA	V	S	A	Y	W	TIP5	

Qrob_T0158140.2	1	236	V	V	A	R	NPM	NPA	M	C	A	F	W	XIP2	
Qrob_T0158150.2 (*)	1	262	V	I	V	G	SPE	NPA	M	C	A	F	W	XIP2	
Qrob_T0158180.2	2	307	I	I	A	K	SPI	NPA	M	C	A	F	W	XIP2	
Qrob_T0158190.2	2	295	I	I	V	K	SPI	NPA	M	C	A	F	W	XIP2	
Qrob_T0158200.2	3	334	I	T	V	R	NPA	NPA	V	C	A	F	W	XIP1	
Qrob_T0656330.2	2	214						NPA						Invalid	unreliable reading frame

([1]) Sequence analysis was performed after the manual merging of Qrob_T0275880.2 and Qrob_T0275890.2.

([2]) Due to poor sequence quality, sequence analysis was performed on its allelic version Qrob_T0751510.2 (qrob_v2_scaffold_2295:14651-1620 following manual curation).

(*) Sequence analysis was performed after the manual curation of intron/exon prediction.

Supplementary Table 34 List of R2R3-MYB, MYB-3R and MYB-4R identified in the pedunculate oak genome (haplome assembly). MYB predicted proteins were retrieved by three different approaches: those containing the MYB domain with the Pfam signature PF00249, those automatically annotated as MYB proteins, and those with homology to one of the Arabidopsis R2R3-MYB proteins after a BLAST-p search with an e-value of $10e^{-10}$ as the threshold. The predicted proteins identified were inspected and manually curated. R2R3-MYB genes were named with consecutive numbers starting from the first gene on the first chromosome scaffold (QroMYB1 - QroMYB129) to the genes not assigned to any chromosome (QroMYB130 - QroMYB139). 3R-MYB and 4R-MYB genes are named with letters in alphabetical order, also starting from the first gene on the first chromosome scaffold (QroMYB3R-A to QroMYB3R-E, and QroMYB4R-A).

MYB ID	Transcript_id	MYB Subgroup	Scaffold_ID on H2.3	Pseudomolecule	Gene start	Gene end	Gene length (in bp UTR + CDS + introns)
QrobMYB1	Qrob_T0404990.2	WPS-III	Qrob_H2.3_Sc0000317	Chr1	414608	415980	1373
QrobMYB2	Qrob_T0371530.2	SAtMYB71	Qrob_H2.3_Sc0000141	Chr1	5600909	5602170	1262
QrobMYB3	Qrob_T0371540.2	SAtMYB71	Qrob_H2.3_Sc0000141	Chr1	5606927	5608155	1229
QrobMYB4	Qrob_T0371920.2	SAtM5	Qrob_H2.3_Sc0000122	Chr1	9166327	9164971	1357
QrobMYB5	Qrob_T0731180.2	S14	Qrob_H2.3_Sc0000439	Chr1	17362712	17364185	1474
QrobMYB6	Qrob_T0252590.2	SAtM80	Qrob_H2.3_Sc0000299	Chr1	17427162	17425846	1317
QrobMYB7	Qrob_T0252570.2	SAtM80	Qrob_H2.3_Sc0000299	Chr1	17472705	17471387	1319
QrobMYB8	Qrob_T0252550.2	WPS-V	Qrob_H2.3_Sc0000299	Chr1	17515334	17513768	1567
QrobMYB9	Qrob_T0252540.2	WPS-V	Qrob_H2.3_Sc0000299	Chr1	17534040	17532476	1565
QrobMYB10	Qrob_T0252530.2	WPS-V	Qrob_H2.3_Sc0000299	Chr1	17555342	17553771	1572
QrobMYB11	Qrob_T0252520.2	WPS-V	Qrob_H2.3_Sc0000299	Chr1	17595722	17593968	1755
QrobMYB12	Qrob_T0252500.2	WPS-V	Qrob_H2.3_Sc0000299	Chr1	17665018	17663242	1777
QrobMYB13	Qrob_T0252490.2	WPS-V	Qrob_H2.3_Sc0000299	Chr1	17716131	17714566	1566
QrobMYB14	Qrob_T0595370.2	SAtM91	Qrob_H2.3_Sc0000542	Chr1	28175476	28174268	1209
QrobMYB15	Qrob_T0660350.2	S5	Qrob_H2.3_Sc0000038	Chr1	39742573	39743584	1012
QrobMYB16	Qrob_T0402380.2	S2 & S3	Qrob_H2.3_Sc0000332	Chr1	45752069	45750448	1622
QrobMYB17	Qrob_T0307500.2	S25	Qrob_H2.3_Sc0000054	Chr1	49689933	49692324	2392
QrobMYB18	Qrob_T0722940.2	S14	Qrob_H2.3_Sc0000511	Chr2	4595691	4597686	1996
QrobMYB19	Qrob_T0059750.2	S22	Qrob_H2.3_Sc0000025	Chr2	5496656	5495769	888
QrobMYB20	Qrob_T0022470.2	S18	Qrob_H2.3_Sc0000016	Chr2	10726752	10728232	1481
QrobMYB21	Qrob_T0270990.2	S19	Qrob_H2.3_Sc0000040	Chr2	18266538	18263431	3108
QrobMYB22	Qrob_T0304630.2	S5	Qrob_H2.3_Sc0000158	Chr2	26868539	26867340	1200
QrobMYB23	Qrob_T0304650.2	S5	Qrob_H2.3_Sc0000158	Chr2	26893414	26894979	1566
QrobMYB24	Qrob_T0304670.2	S5	Qrob_H2.3_Sc0000158	Chr2	26920641	26921682	1042
QrobMYB25	Qrob_T0304700.2	S5	Qrob_H2.3_Sc0000158	Chr2	26949901	26950860	960

QrobMYB26	Qrob_T0304800.2	WPS-I	Qrob_H2.3_Sc0000158	Chr2	27068410	27067516	895
QrobMYB27	Qrob_T0351500.2	SAtM35	Qrob_H2.3_Sc0000145	Chr2	32194944	32197138	2195
QrobMYB28	Qrob_T0418840.2	S14	Qrob_H2.3_Sc0000192	Chr2	39581531	39580189	1343
QrobMYB29	Qrob_T0178010.2	WPS-II	Qrob_H2.3_Sc0000043	Chr2	41309398	41310479	1082
QrobMYB30	Qrob_T0121400.2	SAtM40	Qrob_H2.3_Sc0000083	Chr2	47295425	47296526	1102
QrobMYB31	Qrob_T0203360.2	WPS-II	Qrob_H2.3_Sc0000076	Chr2	53133326	53134402	1077
QrobMYB32	Qrob_T0562460.2	WPS-II	Qrob_H2.3_Sc0000524	Chr2	55606964	55605873	1092
QrobMYB33	Qrob_T0395770.2	S14	Qrob_H2.3_Sc0000314	Chr2	55965127	55966278	1152
QrobMYB34	Qrob_T0398080.2	S6	Qrob_H2.3_Sc0000207	Chr2	69744225	69747170	2946
QrobMYB35	Qrob_T0459570.2	WPS-V	Qrob_H2.3_Sc0000287	Chr2	71044675	71046349	1675
QrobMYB36	Qrob_T0459610.2	WPS-V	Qrob_H2.3_Sc0000287	Chr2	71143342	71141657	1686
QrobMYB37	Qrob_T0324610.2	S5	Qrob_H2.3_Sc0000127	Chr2	72547948	72549947	2000
QrobMYB38	Qrob_T0324630.2	S5	Qrob_H2.3_Sc0000127	Chr2	72611633	72614513	2881
QrobMYB39	Qrob_T0324680.2	S5	Qrob_H2.3_Sc0000127	Chr2	72858478	72860109	1632
QrobMYB40	Qrob_T0365070.2	S1	Qrob_H2.3_Sc0000003	Chr2	85593235	85594741	1507
QrobMYB41	Qrob_T0102440.2	S9a	Qrob_H2.3_Sc0000003	Chr2	87682086	87684183	2098
QrobMYB42	Qrob_T0195940.2	S15	Qrob_H2.3_Sc0000172	Chr2	89155341	89154041	1301
QrobMYB43	Qrob_T0245380.2	SAtM82	Qrob_H2.3_Sc0000027	Chr2	96414306	96415983	1678
QrobMYB44	Qrob_T0278740.2	S14	Qrob_H2.3_Sc0000308	Chr2	106580086	106581601	1516
QrobMYB45	Qrob_T0018660.2	S5	Qrob_H2.3_Sc0000022	Chr2	111265285	111263458	1828
QrobMYB46	Qrob_T0170360.2	S15	Qrob_H2.3_Sc0000015	Chr3	49517391	49516013	1379
QrobMYB47	Qrob_T0202260.2	S9a	Qrob_H2.3_Sc0000176	Chr3	54676627	54673958	2670
QrobMYB48	Qrob_T0038250.2	SAtM46	Qrob_H2.3_Sc0000070	Chr4	3427089	3424578	2512
QrobMYB49	Qrob_T0642710.2	S18	Qrob_H2.3_Sc0000629	Chr4	24187005	24190552	3548
QrobMYB50	Qrob_T0641860.2	WPS-I	Qrob_H2.3_Sc0000468	Chr5	4569573	4570457	885
QrobMYB51	Qrob_T0641880.2	WPS-I	Qrob_H2.3_Sc0000468	Chr5	4694801	4695685	885
QrobMYB52	Qrob_T0641900.2	WPS-I	Qrob_H2.3_Sc0000468	Chr5	4777403	4778287	885
QrobMYB53	Qrob_T0641910.2	S5	Qrob_H2.3_Sc0000468	Chr5	4820012	4818765	1248
QrobMYB54	Qrob_T0070380.2	SAtM35	Qrob_H2.3_Sc0000056	Chr5	18540876	18538781	2096
QrobMYB55	Qrob_T0523450.2	SAtMYB26	Qrob_H2.3_Sc0000464	Chr5	23855006	23856326	1321
QrobMYB56	Qrob_T0221460.2	WPS-V	Qrob_H2.3_Sc0000055	Chr5	26538372	26536935	1438
QrobMYB57	Qrob_T0108420.2	SAtMYB27	Qrob_H2.3_Sc0000198	Chr5	33148198	33147172	1027
QrobMYB58	Qrob_T0072890.2	S11	Qrob_H2.3_Sc0000072	Chr5	40907218	40905536	1683
QrobMYB59	Qrob_T0653450.2	SAtMYB26	Qrob_H2.3_Sc0000325	Chr5	45794689	45793005	1685
QrobMYB60	Qrob_T0697670.2	SAtMYB26	Qrob_H2.3_Sc0000325	Chr5	45851239	45849785	1455
QrobMYB61	Qrob_T0697680.2	SAtMYB26	Qrob_H2.3_Sc0000325	Chr5	45875202	45873379	1824
QrobMYB62	Qrob_T0697730.2	SAtMYB26	Qrob_H2.3_Sc0000325	Chr5	45929859	45928201	1659
QrobMYB63	Qrob_T0701860.2	S11	Qrob_H2.3_Sc0000424	Chr5	65885040	65883694	1347

QrobMYB64	Qrob_T0058400.2	S5	Qrob_H2.3_Sc0000065	Chr5	67078130	67081148	3019
QrobMYB65	Qrob_T0577750.2	S7	Qrob_H2.3_Sc0000053	Chr6	11404917	11400280	4638
QrobMYB66	Qrob_T0005810.2	S15	Qrob_H2.3_Sc0000006	Chr6	21152052	21153310	1259
QrobMYB67	Qrob_T0047220.2	S25	Qrob_H2.3_Sc0000006	Chr6	21502446	21504146	1701
QrobMYB68	Qrob_T0379650.2	SAtMYB71	Qrob_H2.3_Sc0000566	Chr6	25445570	25447050	1481
QrobMYB69	Qrob_T0690000.2	SAtM46	Qrob_H2.3_Sc0000565	Chr6	27337413	27334918	2496
QrobMYB70	Qrob_T0199530.2	WPS-I	Qrob_H2.3_Sc0000179	Chr6	43171185	43172069	885
QrobMYB71	Qrob_T0199740.2	S22	Qrob_H2.3_Sc0000179	Chr6	43505358	43506296	939
QrobMYB72	Qrob_T0346410.2	S4	Qrob_H2.3_Sc0000011	Chr6	53280743	53279865	879
QrobMYB73	Qrob_T0418350.2	S10 & S24	Qrob_H2.3_Sc0000204	Chr6	56246814	56245513	1302
QrobMYB74	Qrob_T0738480.2	S1	Qrob_H2.3_Sc0000662	Chr7	9584571	9583099	1473
QrobMYB75	Qrob_T0119930.2	SAtMYB71	Qrob_H2.3_Sc0000123	Chr7	10575199	10573946	1254
QrobMYB76	Qrob_T0388360.2	S14	Qrob_H2.3_Sc0000367	Chr7	47727255	47728879	1625
QrobMYB77	Qrob_T0657180.2	SAtM91	Qrob_H2.3_Sc0000478	Chr7	51905061	51903988	1074
QrobMYB78	Qrob_T0033520.2	S9b	Qrob_H2.3_Sc0000007	Chr8	19882307	19880196	2112
QrobMYB79	Qrob_T0626620.2	S15	Qrob_H2.3_Sc0000156	Chr8	26379626	26380864	1239
QrobMYB80	Qrob_T0647280.2	S18	Qrob_H2.3_Sc0000283	Chr8	36441820	36438939	2882
QrobMYB81	Qrob_T0654710.2	WPS-V	Qrob_H2.3_Sc0000642	Chr8	38444539	38445951	1413
QrobMYB82	Qrob_T0668650.2	WPS-V	Qrob_H2.3_Sc0000642	Chr8	38539863	38541297	1435
QrobMYB83	Qrob_T0668640.2	WPS-V	Qrob_H2.3_Sc0000642	Chr8	38597083	38598848	1766
QrobMYB84	Qrob_T0466400.2	S11	Qrob_H2.3_Sc0000570	Chr8	40545518	40544193	1326
QrobMYB85	Qrob_T0436030.2	S22	Qrob_H2.3_Sc0000008	Chr8	47999625	48000389	765
QrobMYB86	Qrob_T0437590.2	WPS-II	Qrob_H2.3_Sc0000334	Chr8	51157045	51155572	1474
QrobMYB87	Qrob_T0303790.2	S23	Qrob_H2.3_Sc0000251	Chr8	66214685	66211087	3599
QrobMYB88	Qrob_T0411100.2	S21	Qrob_H2.3_Sc0000251	Chr8	66330876	66332389	1514
QrobMYB89	Qrob_T0277200.2	S21	Qrob_H2.3_Sc0000457	Chr9	4255611	4253903	1709
QrobMYB90	Qrob_T0344270.2	S5	Qrob_H2.3_Sc0000600	Chr9	5365424	5366566	1143
QrobMYB91	Qrob_T0344260.2	S5	Qrob_H2.3_Sc0000600	Chr9	5379324	5380656	1333
QrobMYB92	Qrob_T0344200.2	S4	Qrob_H2.3_Sc0000600	Chr9	5457882	5456407	1476
QrobMYB93	Qrob_T0191710.2	S5	Qrob_H2.3_Sc0000155	Chr9	7921886	7920786	1101
QrobMYB94	Qrob_T0612820.2	S16	Qrob_H2.3_Sc0000047	Chr9	14775500	14778940	3441
QrobMYB95	Qrob_T0612850.2	S9b	Qrob_H2.3_Sc0000047	Chr9	14807889	14810821	2933
QrobMYB96	Qrob_T0575950.2	S2 & S3	Qrob_H2.3_Sc0000017	Chr9	17357797	17359638	1842
QrobMYB97	Qrob_T0246270.2	SAtMYB88	Qrob_H2.3_Sc0000017	Chr9	19640731	19634180	6552
QrobMYB98	Qrob_T0464600.2	SAtMYB85	Qrob_H2.3_Sc0000446	Chr9	20541214	20543134	1921
QrobMYB99	Qrob_T0123830.2	S10 & S24	Qrob_H2.3_Sc0000112	Chr9	24701012	24702528	1517
QrobMYB100	Qrob_T0460820.2	S21	Qrob_H2.3_Sc0000152	Chr9	29184636	29183320	1317
QrobMYB101	Qrob_T0369530.2	S2 & S3	Qrob_H2.3_Sc0000031	Chr9	36883092	36884669	1578

QrobMYB102	Qrob_T0612260.2	S14	Qrob_H2.3_Sc0000097	Chr9	48108561	48107270	1292
QrobMYB103	Qrob_T0381310.2	SAtMYB27	Qrob_H2.3_Sc0000278	Chr10	8229312	8230354	1043
QrobMYB104	Qrob_T0451690.2	S13	Qrob_H2.3_Sc0000669	Chr10	10999232	11000574	1343
QrobMYB105	Qrob_T0452180.2	S1	Qrob_H2.3_Sc0000085	Chr10	14501122	14502632	1511
QrobMYB106	Qrob_T0555330.2	no subgroup	Qrob_H2.3_Sc0000085	Chr10	14962232	14966952	4721
QrobMYB107	Qrob_T0555340.2	no subgroup	Qrob_H2.3_Sc0000085	Chr10	14980766	14983349	2584
QrobMYB108	Qrob_T0179590.2	SAtMYB26	Qrob_H2.3_Sc0000951	Chr10	17804921	17803483	1439
QrobMYB109	Qrob_T0286880.2	S2 & S3	Qrob_H2.3_Sc0000009	Chr10	18999043	18995821	3223
QrobMYB110	Qrob_T0361180.2	S20	Qrob_H2.3_Sc0000450	Chr10	24976888	24978282	1395
QrobMYB111	Qrob_T0352280.2	WPS-III	Qrob_H2.3_Sc0000347	Chr10	27861855	27860633	1223
QrobMYB112	Qrob_T0439830.2	WPS-III	Qrob_H2.3_Sc0000347	Chr10	27882273	27881283	991
QrobMYB113	Qrob_T0309480.2	S21	Qrob_H2.3_Sc0000263	Chr10	33715578	33714210	1369
QrobMYB114	Qrob_T0416480.2	S21	Qrob_H2.3_Sc0000394	Chr10	40481766	40480595	1172
QrobMYB115	Qrob_T0189820.2	S5	Qrob_H2.3_Sc0000253	Chr11	588966	587658	1309
QrobMYB116	Qrob_T0189800.2	S5	Qrob_H2.3_Sc0000253	Chr11	618203	616862	1342
QrobMYB117	Qrob_T0189780.2	S5	Qrob_H2.3_Sc0000253	Chr11	640279	638759	1521
QrobMYB118	Qrob_T0275930.2	WPS-III	Qrob_H2.3_Sc0000230	Chr11	3182332	3181117	1216
QrobMYB119	Qrob_T0409250.2	SAtMYB26	Qrob_H2.3_Sc0000166	Chr11	10466232	10465025	1208
QrobMYB120	Qrob_T0203780.2	S22	Qrob_H2.3_Sc0000089	Chr11	23625175	23624288	888
QrobMYB121	Qrob_T0011720.2	S4	Qrob_H2.3_Sc0000005	Chr11	35738109	35739384	1276
QrobMYB122	Qrob_T0291490.2	S13	Qrob_H2.3_Sc0000266	Chr11	43032216	43033889	1674
QrobMYB123	Qrob_T0387070.2	S4	Qrob_H2.3_Sc0000389	Chr11	45113992	45112970	1023
QrobMYB124	Qrob_T0099630.2	S5	Qrob_H2.3_Sc0000037	Chr11	51663438	51665216	1779
QrobMYB125	Qrob_T0302600.2	S10 & S24	Qrob_H2.3_Sc0000235	Chr12	6308619	6309765	1147
QrobMYB126	Qrob_T0541420.2	SAtMYB85	Qrob_H2.3_Sc0000023	Chr12	9014270	9012796	1475
QrobMYB127	Qrob_T0082290.2	S20	Qrob_H2.3_Sc0000412	Chr12	16016899	16018134	1236
QrobMYB128	Qrob_T0115630.2	SAtM5	Qrob_H2.3_Sc0000146	Chr12	25663954	25662876	1079
QrobMYB129	Qrob_T0388890.2	SAtM103	Qrob_H2.3_Sc0000652	Chr12	30990081	30992496	2416
QrobMYB130	Qrob_T0026160.2	S20	Qrob_H2.3_Sc0000044	UA	1660115	1661281	1167
QrobMYB131	Qrob_T0468060.2	S22	Qrob_H2.3_Sc0000161	UA	1159605	1158892	714
QrobMYB132	Qrob_T0675440.2	SAtMYB26	Qrob_H2.3_Sc0000165	UA	1378120	1376578	1543
QrobMYB133	Qrob_T0675450.2	SAtMYB26	Qrob_H2.3_Sc0000165	UA	1418714	1417338	1377
QrobMYB134	Qrob_T0122500.2	S18	Qrob_H2.3_Sc0000234	UA	176552	171484	5069
QrobMYB135	Qrob_T0411820.2	SAtM5	Qrob_H2.3_Sc0000354	UA	342530	343605	1076
QrobMYB136	Qrob_T0412190.2	WPS-IV	Qrob_H2.3_Sc0000358	UA	390490	388344	2147
QrobMYB137	Qrob_T0728290.2	S13	Qrob_H2.3_Sc0000840	UA	172040	170522	1519
QrobMYB138	Qrob_T0653500.2	SAtMYB26	Qrob_H2.3_Sc0000945	UA	11717	13539	1823
QrobMYB139	Qrob_T0769430.2	WPS-III	Qrob_H2.3_Sc0001151	UA	32240	33455	1216

QrobMYB3R-A	Qrob_T0010150.2	MYB-3R	Qrob_H2.3_Sc0000013	Chr2	60246757	60253755	6999
QrobMYB3R-B	Qrob_T0576750.2	MYB-3R	Qrob_H2.3_Sc0000478	Chr7	51811899	51816721	4823
QrobMYB3R-C	Qrob_T0033120.2	MYB-3R	Qrob_H2.3_Sc0000007	Chr8	20517700	20521806	4107
QrobMYB3R-D	Qrob_T0129360.2	MYB-3R	Qrob_H2.3_Sc0000474	Chr12	14949147	14953664	4518
QrobMYB3R-E	Qrob_T0264880.2	MYB-3R	Qrob_H2.3_Sc0000046	Chr12	22045628	22052297	6670
QrobMYB4R-A	Qrob_T0439780.2	MYB-4R	Qrob_H2.3_Sc0000347	Chr10	28525076	28534063	8988

Supplementary Table 35 Gene content of the glutaredoxin, thioredoxin and glutathione transferase families in the pedunculate oak genome (haplome assembly) and selected embryophytes. In addition to oak sequences, other sequences were retrieved from genomic data available from thr Phytozome V11 portal by BLAST-p and tBLAST-n analyses using *P. trichocarpa* and *A. thaliana* sequences as references. The different classes in the grx, trx and gst families were defined according to[83–85], respectively.

	Q. robur	*P. trichocarpa*	*A. thaliana*	*V. vinifera*	*P. persica*	*C. clementina*	*F. vesca*	*E. grandis*	*R. communis*	*C. papaya*	*T. cacao*	*O. sativa*	*S. bicolor*	*P. patens*
**GLUTAREDOXINS**	*25*	*38*	*33*	*25*	*24*	*23*	*24*	*32*	*22*	*18*	*17*	*29*	*32*	*15*
Class I	*5*	*6*	*6*	*5*	*5*	*5*	*4*	*7*	*6*	*4*	*5*	*5*	*5*	*5*
C1	*1*	*2*	*1*	*1*	*1*	*1*	*1*	*2*	*1*	*0*	*1*	*0*	*0*	*0*
C2	*1*	*1*	*1*	*1*	*1*	*1*	*1*	*2*	*1*	*1*	*1*	*2*	*2*	*3*
C3	*1*	*1*	*1*	*1*	*1*	*1*	*1*	*1*	*1*	*1*	*1*	*1*	*1*	*1*
C4	*1*	*1*	*1*	*1*	*1*	*1*	*1*	*1*	*1*	*1*	*1*	*1*	*1*	*0*
C5	*0*	*0*	*1*	*0*	*0*	*0*	*0*	*0*	*0*	*0*	*0*	*0*	*0*	*0*
S12	*1*	*1*	*1*	*1*	*1*	*1*	*0*	*1*	*2*	*1*	*1*	*1*	*1*	*1*
Class II	*4*	*5*	*4*	*5*	*5*	*4*	*3*	*5*	*4*	*3*	*4*	*5*	*6*	*8*
S14	*1*	*1*	*1*	*2*	*1*	*1*	*1*	*1*	*1*	*0*	*1*	*1*	*1*	*2*
S15	*1*	*1*	*1*	*1*	*2*	*1*	*0*	*2*	*1*	*1*	*1*	*2*	*2*	*2*
S16	*1*	*1*	*1*	*1*	*1*	*1*	*1*	*1*	*1*	*1*	*1*	*1*	*2*	*1*
S17	*1*	*2*	*1*	*1*	*1*	*1*	*1*	*1*	*1*	*1*	*1*	*1*	*1*	*3*
Class III	*14*	*24*	*21*	*13*	*12*	*12*	*15*	*18*	*10*	*9*	*11*	*17*	*19*	*2*
Class IV	*2*	*3*	*2*	*2*	*2*	*2*	*2*	*2*	*2*	*2*	*2*	*2*	*2*	*0*
**THIOREDOXINS**	*41*	*49*	*41*	*35*	*39*	*31*	*34*	*48*	*31*	*30*	*36*	*34*	*33*	*34*
CDSP32	*1*	*1*	*1*	*1*	*1*	*1*	*1*	*1*	*1*	*1*	*1*	*1*	*1*	*1*
Clot	*1*	*1*	*1*	*1*	*1*	*1*	*1*	*1*	*1*	*0*	*1*	*1*	*1*	*1*
HCF164	*1*	*1*	*1*	*1*	*1*	*1*	*1*	*1*	*1*	*1*	*1*	*1*	*1*	*1*
Trx f	*1*	*1*	*2*	*1*	*1*	*1*	*1*	*2*	*1*	*1*	*1*	*1*	*1*	*3*
Trx h	*6*	*10*	*11*	*6*	*8*	*6*	*8*	*10*	*6*	*8*	*8*	*7*	*6*	*5*
Trx m	*4*	*8*	*4*	*3*	*5*	*3*	*4*	*4*	*4*	*3*	*4*	*4*	*3*	*6*
Trx o	*1*	*1*	*2*	*1*	*1*	*1*	*1*	*1*	*1*	*1*	*1*	*1*	*1*	*1*
Trx x	*1*	*1*	*1*	*1*	*1*	*1*	*1*	*1*	*0*	*1*	*1*	*1*	*1*	*2*

Trx y	1	2	2	1	2	1	1	2	1	1	1	1	1	1
Trx z	1	1	1	1	1	1	1	1	1	1	1	1	1	2
Trx like 1	1	1	1	1	1	1	1	1	1	0	1	1	1	1
Trx like 2	2	3	2	2	2	2	2	2	1	1	2	1	2	2
Trx lilium 1	2	2	3	2	2	2	3	3	2	2	2	2	3	0
Trx lilium 2	1	2	1	1	1	1	1	2	1	1	2	1	1	1
Trx lilium 3	1	1	1	1	1	1	1	1	1	1	1	1	1	1
TDX	1	2	1	1	1	0	1	1	1	0	1	1	0	0
NRX1	5	5	1	5	4	2	0	7	2	3	1	2	2	0
NRX2	1	1	0	1	1	1	1	2	1	1	1	1	1	0
NRX3	1	1	1	1	1	1	1	1	0	1	1	1	1	0
NTRa/b	6	2	2	1	1	1	1	2	2	1	2	2	2	2
NTRc	1	1	1	1	1	1	1	1	1	1	1	1	1	2
FTR-b	1	1	1	1	1	1	1	1	1	0	1	1	1	2
**GLUTATHIONE TRANSFERASES**	88	83	61	59	71	68	50	110	52	36	60	78	90	39
DHAR	1	3	3	2	2	2	2	3	3	2	2	2	3	3
GHR	2	2	4	1	2	2	2	3	2	0	3	2	2	2
GSTL	2	3	3	4	2	3	3	8	3	2	2	3	4	1
mPGES2	2	3	1	2	2	2	2	3	1	1	2	1	1	2
GSTI	0	0	0	0	0	0	0	0	0	0	0	0	0	1
GSTH	0	0	0	0	0	0	0	0	0	0	0	0	0	7
GSTF	12	8	13	8	9	8	5	19	4	5	9	16	17	9
GSTT	1	2	3	1	1	2	1	1	3	1	1	1	2	2
GSTZ	2	2	2	3	2	3	2	2	2	1	2	4	4	1
EF1Bγ	2	3	2	?	2	2	3	1	1	1	1	2	2	3
Ure2p	0	0	0	0	0	0	0	0	0	0	0	0	0	1
Metaxin	1	2	1	1	1	1	1	1	1	1	1	1	1	2
GSTU	62	54	28	36	47	42	28	62	31	21	36	45	53	0
TCHQD	1	1	1	1	1	1	1	7	1	1	1	1	1	5

Supplementary Table 36 List of MLO genes identified in the pedunculate oak genome (haplome assembly). MLO predicted proteins were retrieved by three different approaches: those automatically annotated as MLO proteins, those containing the MLO domain with the Pfam signature PF03094, and those with homology to one of the *Arabidopsis* MLO proteins after BLAST-p search using an e-value of $10e^{-10}$ as the threshold. The predicted proteins identified were inspected and manually curated. The number of predicted transmembrane domains was analyzed with Phobius (http://phobius.sbc.su.se/).

MLO gene ID	Complete/partial protein	MLO Clade	Chrom.	#residues	exons	#Trans membrane domains
Qrob_T0355750.2	complete	VI	1	561	15	7
Qrob_T0355780.2	complete	V	1	584	15	7
Qrob_T0355790.2	complete	V	1	585	15	7
Qrob_T0173130.2	complete	I	2	519	13	7
Qrob_T0222290.2	complete	II	2	510	15	7
Qrob_T0482700.2	complete	III	2	522	15	7
Qrob_T0725960.2	partial		2			
Qrob_T0725970.2	partial		2			
Qrob_T0562700.2	complete	II	5	504	15	8
Qrob_T0346330.2	complete	I	6	564	15	7
Qrob_T0603780.2	complete	III	6	573	15	7
Qrob_T0327490.2	partial		7			
Qrob_T0455210.2	complete	V	8	565	15	7
Qrob_T0468620.2	complete	I	8	558	15	7
Qrob_T0572110.2	complete	V	8	539	14	7
Qrob_T0572120.2	complete	V	8	533	14	7
Qrob_T0572170.2	complete	V	8	575	15	7
Qrob_T0032420.2	complete	II	10	516	14	7
Qrob_T0032520.2	complete	II	10	520	14	7
Qrob_T0032530.2	complete	II	10	521	14	7
Qrob_T0254270.2	complete	V	10	557	15	7
Qrob_T0254250.2	partial		10			
Qrob_T0254260.2	partial		10			
Qrob_T0032510.2	partial		10			
Qrob_T0254290.2	partial		11			
Qrob_T0523040.2	complete	II	Scaf. 408	505	15	7

Supplementary Table 37 Mildew resistance locus o (MLO) family members from selected plant species and their phylogenetic classification[a]. Clade V (in bold) corresponds to the clade for which a function in powdery mildew susceptibility/resistance has been demonstrated. We completed the table provided by Acevedo-Garcia et al.[91] with recently published data and data obtained from the pedunculate oak genome (haplome assembly).

Scientific name	Common name	#MLO genes	Clade ID							
			I	II	III	IV	**V**	VI	VII	Reference*
Arabidopsis thaliana	Thale cress	15	3	3	5	0	**3**	1	0	[161]
Cucumis lanatus	Cucumber	14	4	1	3	0	**3**	1	2	[162]
Cucumis sativus	Cucumber	14	4	2	3	0	**3**	1	1	[163]
Fragaria vesca	Strawberry	18	3	6	1	1	**3**	2	1	[92]
Glycine max	Soybean	39 [c]	7	5	8	2	**11**	6	0	[164]
Gossypium hirsutum	Cotton	38 [c]	8	11	3	2	**8**	2	4	[165]
Malus domestica	Apple	21 [c]	5	5	3	0	**4**	2	1	[92]
Oryza sativa	Rice	12	2	6	1	3	**0**	0	0	[166]
Prunus persica	Peach	16	3	6	2	0	**3**	2	0	[167]
Prunus persica	Peach	19	3	5	2	1	**3**	3	1	[92]
Solanum lycopersicum	Tomato	17[b]	3	4	3	0	**4**	1	1	[168]
Solanum tuberosum	Potato	13	3	4	3	0	**3**	0	0	[169]
Triticum aestivum	Wheat	8	1	3	1	3	**0**	0	0	[170]
Vitis vinifera	Grapevine	17	3	3	2	1	**6**	2	0	[171]
Quercus robur	**Oak**	19	3	6	2	0	**7**	1	0	this study

[a] Only fully characterized MLO families are shown. Classification is based on previous publications.
[b] One truncated MLO family member (SlMLO14) was excluded from phylogenetic analysis
[c] Species with recent whole-genome duplication
* from Acevedo-Garcia et al.[91] until 2014

Supplementary Table 38 Annotation of the *Populus trichocarpa* laccase genes. Poplar laccase gene annotations were updated according to the most recent annotation (v3) available in Phytozome. Synonyms of the gene annotations used in this study are presented, together with previous annotations based on Phytozome v2 annotation.

Annotation [132]	Annotation [129]	Synonym (Annotation v2)	*P trichocarpa* Alias	Gene name (v3)	Transcript name (v3)
PtrLAC1	PtrLAC1	POPTR_0001s14010	PtrLAC1	Potri.001G054600	Potri.001G054600.1
PtrLAC2	PtrLAC2	POPTR_0001s18500	PtrLAC2	Potri.001G184300	Potri.001G184300.1
PtrLAC3	PtrLAC3	POPTR_0001s21380	PtrLAC3	Potri.001G206200	Potri.001G206200.1
PtrLAC4	PtrLAC4	POPTR_0001s25580	PtrLAC4	Potri.001G248700	Potri.001G248700.1
PtrLAC5	PtrLAC5	POPTR_0001s35740	PtrLAC5	Potri.001G341600	Potri.001G341600.1
PtrLAC6	PtrLAC6	POPTR_0001s41160	PtrLAC6	Potri.001G401100	Potri.001G401100.1
PtrLAC7	PtrLAC7	POPTR_0001s41170	PtrLAC7	Potri.001G401300	Potri.001G401300.1
PtrLAC8	PtrLAC8	POPTR_0004s16370	PtrLAC8	Potri.004G156400	Potri.004G156400.1
	PtrLAC9	POPTR_0005s22230	PtrLAC9	Potri.005G200500	Potri.005G200500.1
PtrLAC9	PtrLAC10	POPTR_0005s22240	PtrLAC10	Potri.005G200600	Potri.005G200600.1
PtrLAC10	PtrLAC11	POPTR_0005s22250	PtrLAC11	Potri.005G200700	Potri.005G200700.1
PtrLAC11	PtrLAC12	POPTR_0006s08740	PtrLAC12	Potri.006G087100	Potri.006G087100.1
PtrLAC12	PtrLAC13	POPTR_0006s08780	PtrLAC13	Potri.006G087500	Potri.006G087500.1
PtrLAC13	PtrLAC14	POPTR_0006s09520	PtrLAC14	Potri.006G094100	Potri.006G094100.1
PtrLAC14	PtrLAC15	POPTR_0006s09830	PtrLAC15	Potri.006G096900	Potri.006G096900.1
PtrLAC15	PtrLAC16	POPTR_0006s09840	PtrLAC16	Potri.006G097000	Potri.006G097000.1
PtrLAC49	PtrLAC51	POPTR_0958s00200	PtrLAC17	Potri.006G097100	Potri.006G097100.1

PtrLAC16	PtrLAC17	POPTR_0007s13050	PtrLAC18	Potri.007G023300	Potri.007G023300.1
PtrLAC17	PtrLAC18	POPTR_0008s06430	PtrLAC19	Potri.008G064000	Potri.008G064000.1
PtrLAC18	PtrLAC19	POPTR_0008s07370	PtrLAC20	Potri.008G073700	Potri.008G073700.1
PtrLAC19	PtrLAC20	POPTR_0008s07380	PtrLAC21	Potri.008G073800	Potri.008G073800.1
PtrLAC20	PtrLAC21	POPTR_0009s03940	PtrLAC22	Potri.009G034500	Potri.009G034500.1
PtrLAC21	PtrLAC22	POPTR_0009s04720	PtrLAC23	Potri.009G042500	Potri.009G042500.1
PtrLAC22	PtrLAC23	POPTR_0009s10550	PtrLAC24	Potri.009G102700	Potri.009G102700.1
PtrLAC23	PtrLAC24	POPTR_0009s15840	PtrLAC25	Potri.009G156600	Potri.009G156600.1
PtrLAC24	PtrLAC25	POPTR_0009s15860	PtrLAC26	Potri.009G156800	Potri.009G156800.1
PtrLAC25	PtrLAC26	POPTR_0010s19080	PtrLAC27	Potri.010G183500	Potri.010G183500.1
PtrLAC26	PtrLAC27	POPTR_0010s19090	PtrLAC28	Potri.010G183600	Potri.010G183600.1
PtrLAC27	PtrLAC28	POPTR_0010s20050	PtrLAC29	Potri.010G193100	Potri.010G193100.1
PtrLAC28	PtrLAC29	POPTR_0011s06880	PtrLAC30	Potri.011G071100	Potri.011G071100.1
PtrLAC29	PtrLAC30	POPTR_0011s12090	PtrLAC31	Potri.011G120200	Potri.011G120200.1
PtrLAC30	PtrLAC31	POPTR_0011s12100	PtrLAC32	Potri.011G120300	Potri.011G120300.1
PtrLAC31	PtrLAC32	POPTR_0012s04620	PtrLAC33	Potri.012G048900	Potri.012G048900.1
PtrLAC32	PtrLAC33	POPTR_0013s14890	PtrLAC34	Potri.013G152700	Potri.013G152700.1
PtrLAC33	PtrLAC34	POPTR_0014s09610	PtrLAC35	Potri.014G100600	Potri.014G100600.1
PtrLAC36	PtrLAC38	POPTR_0015s04370	PtrLAC36	Potri.015G040400	Potri.015G040400.1
PtrLAC35	PtrLAC37	POPTR_0015s04350	PtrLAC37	Potri.015G040600	Potri.015G040600.1
PtrLAC34	PtrLAC36	POPTR_0015s04340	PtrLAC38	Potri.015G040700	Potri.015G040700.1
	PtrLAC35	POPTR_0015s04330	PtrLAC39	Potri.015G040800	Potri.015G040800.1
PtrLAC37	PtrLAC39	POPTR_0016s11500	PtrLAC40	Potri.016G106000	Potri.016G106000.1

PtrLAC38	PtrLAC40	POPTR_0016s11520	PtrLAC41	Potri.016G106100	Potri.016G106100.1
PtrLAC39	PtrLAC41	POPTR_0016s11540	PtrLAC42	Potri.016G106300	Potri.016G106300.1
PtrLAC48	PtrLAC50	POPTR_0091s00270	PtrLAC43	Potri.016G107900	Potri.016G107900.1
PtrLAC40	PtrLAC42	POPTR_0016s11950	PtrLAC44	Potri.016G112000	Potri.016G112000.1
PtrLAC41	PtrLAC43	POPTR_0016s11960	PtrLAC45	Potri.016G112100	Potri.016G112100.1
PtrLAC42	PtrLAC44	POPTR_0019s11810	PtrLAC46	Potri.019G088500	Potri.019G088500.1
PtrLAC43	PtrLAC45	POPTR_0019s11820	PtrLAC47	Potri.019G088600	Potri.019G088600.1
PtrLAC44	PtrLAC46	POPTR_0019s11830	PtrLAC48	Potri.019G088700	Potri.019G088700.1
PtrLAC45	PtrLAC47	POPTR_0019s11850	PtrLAC49	Potri.019G088800	Potri.019G088800.1
PtrLAC46	PtrLAC48	POPTR_0019s11860	PtrLAC50	Potri.019G088900	Potri.019G088900.1
PtrLAC47	PtrLAC49	POPTR_0019s14530	PtrLAC51	Potri.019G121700	Potri.019G121700.1

Supplementary Table 39 Number of laccase genes per phylogenetic group for Arabidopsis, poplar and oak.

	A. thaliana	*Q. robur*	*P. trichocarpa*
**Group 1**	1	1	5
**Group 2**	6	14	24
**Group 3**	1	1	1
**Group 4**	3	1	5
**Group 5**	4	1	6
**Group 6**	1	7	6
**Group 7**	1	2	4
**Total**	17	27	51

Supplementary Table 40 Available or soon to be released eudicot whole-genome sequences (as reported in https://genomevolution.org/wiki/index.php/Sequenced_plant_genomes as of 18 December 2016), with growth habit from Zanne et al.[152] (H = herbaceous, W = woody), supplemented with Google searches, and % woody based on the strong prior from Fitzjohn et al.[149] at the genus, family and order levels. The names of the species were updated with taxize R-package, using Plant List version 1.1.

				% woody		
**Order**	**Family**	**Sequenced species**	**Growth form**	**Genus**	**Family**	**Order**
Brassicales	Brassicaceae	*Arabidopsis halleri*	H	0	5	15
Brassicales	Brassicaceae	*Arabidopsis lyrata*	H	0	5	15
Brassicales	Brassicaceae	*Arabidopsis thaliana*	H	0	5	15
Fabales	Fabaceae	*Arachis hypogaea*	H	0	63	62
Caryophyllales	Amaranthaceae	*Beta vulgaris*	H	0	38	42
Brassicales	Brassicaceae	*Brassica napus*	H	0	5	15
Brassicales	Brassicaceae	*Brassica oleracea*	H	0	5	15
Brassicales	Brassicaceae	*Brassica rapa*	H	0	5	15
Brassicales	Brassicaceae	*Camelina sativa*	H	0	5	15
Rosales	Cannabaceae	*Cannabis sativa*	H	0	96	75
Solanales	Solanaceae	*Capsicum annuum*	H	0	62	50
Fabales	Fabaceae	*Cicer arietinum*	H	0	63	62
Cucurbitales	Cucurbitaceae	*Citrullus lanatus*	H	0	13	11
Cucurbitales	Cucurbitaceae	*Cucumis melo*	H	0	13	11
Cucurbitales	Cucurbitaceae	*Cucumis sativus*	H	0	13	11
Asterales	Asteraceae	*Erigeron canadensis*	H	8	25	26
Rosales	Rosaceae	*Fragaria vesca*	H	0	76	75
Fabales	Fabaceae	*Glycine max*	H	13	63	62
Rosales	Cannabaceae	*Humulus lupulus*	H	0	96	75
Fabales	Fabaceae	*Lotus corniculatus*	H	25	63	62
Fabales	Fabaceae	*Lupinus angustifolius*	H	22	63	62
Fabales	Fabaceae	*Medicago truncatula*	H	8	63	62
Lamiales	Phrymaceae	*Mimulus guttatus*	H	27	24	45
Proteales	Nelumbonaceae	*Nelumbo nucifera*	H	0	0	100
Solanales	Solanaceae	*Nicotiana benthamiana*	H	33	62	50
Fabales	Fabaceae	*Phaseolus vulgaris*	H	0	63	62
Brassicales	Brassicaceae	*Raphanus raphanistrum*	H	0	5	15
Brassicales	Brassicaceae	*Sisymbrium irio*	H	0	5	15

Solanales	Solanaceae	*Solanum lycopersicum*	H	65	62	50
Solanales	Solanaceae	*Solanum melongena*	H	65	62	50
Solanales	Solanaceae	*Solanum tuberosum*	H	65	62	50
Lamiales	Lentibulariaceae	*Utricularia gibba*	H	0	3	45
Brassicales	Brassicaceae	*Aethionema arabicum*	H	0	5	15
Ranunculales	Ranunculaceae	*Aquilegia formosa*	H	0	16	31
Brassicales	Brassicaceae	*Capsella rubella*	H	0	5	15
Sapindales	Rutaceae	*Citrus clementina*	W	100	100	100
Brassicales	Cleomaceae	*Cleome houtteana*	H	15	15	15
Brassicales	Brassicaceae	*Eutrema parvulum*	H	0	5	15
Brassicales	Brassicaceae	*Eutrema salsugineum*	H	0	5	15
Lamiales	Lentibulariaceae	*Genlisea aurea*	H	47	3	45
Brassicales	Brassicaceae	*Leavenworthia alabamica*	H	0	5	15
Malpighiales	Linaceae	*Linum usitatissimum*	H	33	54	79
Solanales	Solanaceae	*Lycopersicon pennellii*	H	0	62	50
Rosales	Rosaceae	*Prunus mume*	W	100	76	75
Rosales	Rosaceae	*Pyrus bretschneideri*	W	100	76	75
Solanales	Solanaceae	*Solanum arcanum*	H	65	62	50
Solanales	Solanaceae	*Solanum habrochaites*	H	65	62	50
Solanales	Solanaceae	*Solanum pimpinellifolium*	H	65	62	50
Fabales	Fabaceae	*Vigna radiata*	H	0	63	62
Ericales	Actinidiaceae	*Actinidia chinensis*	W	100	100	84
Malvales	Thymelaeaceae	*Aquilaria agallocha*	W	100	94	86
Sapindales	Meliaceae	*Azadirachta indica*	W	100	100	100
Fagales	Betulaceae	*Betula nana*	W	100	100	100
Fabales	Fabaceae	*Cajanus cajan*	W	100	63	62
Brassicales	Caricaceae	*Carica papaya*	W	100	93	15
Sapindales	Rutaceae	*Citrus sinensis*	W	100	100	100
Gentianales	Rubiaceae	*Coffea canephora*	W	100	82	76
Myrtales	Myrtaceae	*Eucalyptus grandis*	W	100	100	92
Lamiales	Oleaceae	*Fraxinus excelsior*	W	100	99	45
Malvales	Malvaceae	*Gossypium raimondii*	W	93	83	86
Malpighiales	Euphorbiaceae	*Hevea brasiliensis*	W	100	72	79
Rosales	Rosaceae	*Malus domestica*	W	100	76	75
Malpighiales	Euphorbiaceae	*Manihot esculenta*	W	100	72	79

Malpighiales	Salicaceae	*Populus trichocarpa*	W	100	100	79
Rosales	Rosaceae	*Prunus persica*	W	100	76	75
Fagales	Fagaceae	*Quercus robur*	W	100	100	100
Malpighiales	Euphorbiaceae	*Ricinus communis*	W	100	72	79
Malpighiales	Salicaceae	*Salix purpurea*	W	100	100	79
Malvales	Malvaceae	*Theobroma cacao*	W	100	83	86
Ericales	Ericaceae	*Vaccinium corymbosum*	W	100	98	84
Ericales	Ericaceae	*Vaccinium macrocarpon*	W	100	98	84
Vitales	Vitaceae	*Vitis vinifera*	W	100	98	98
Rosales	Rhamnaceae	*Ziziphus jujuba*	W	100	99	75

Supplementary Table 41 Summary of the data used for GO term enrichment analysis for three gene categories: TDG (tamdemly duplicated genes), LDG (long distance-duplicated genes) and SG (singleton genes). Abbreviations are as follows: MF (Molecular Function), BP (Biological Process), CC (Cellular Component).

	Total	MF	BP	CC
**No. of genes with GO terms in the reference**	16,820	15,413	10,073	3,604
**Total No. of GO terms**	3,433	1,179	1,867	387
**No. of TDGs with GO terms**	6,686	6,280	4,103	1,086
**No. of LDGs with GO terms**	6,230	5,680	3,844	1,536
**No. of SGs with GO terms**	3,904	3,453	2,126	982
**Significant GO terms: TDGs**	97	55	32	10
**Significant GO terms: LDGs**	144	65	62	17
**Significant GO terms: SGs**	240	80	130	30

Supplementary Table 42 Summary of the data used for GO term enrichment analysis in the orthogroups expanded in pedunculate oak.

	Total	MF	BP	CC
**No. of genes with GO terms in the reference**	16,820	15,413	10,073	3,604
**No. of genes with GO terms in orthogroups expanded in oak**	4,217	4,032	2,267	445
**Total No. of GO terms in orthogroups expanded in oak**	3,433	1,179	1,867	387
**Significant GO terms in orthogroups expanded in oak**	58	33	17	8

Supplementary Table 43 Summary of gene ontology (GO) enrichment analysis in woody perennials (A) and herbaceous species (B). Abbreviations are as follows: Molecular function (MF), biological process (BP) and cellular component (CC).

A. Woody perennials				
	**Total**	**MF**	**BP**	**CC**
**No. of orthogroups with GO terms in the reference**	36,844	16,703	11,495	5,073
**Total number of GO terms in the reference**	3,936	1,341	2,131	464
**No. of significant expanded orthogroups with GO terms**	108	104	84	39
**No. of significant GO terms in expanded orthogroups**	61	38	19	4

**B. Herbaceous species**				
	**Total**	**MF**	**BP**	**CC**
**No. of orthogroups with GO terms in the reference**	36,844	16,703	11,495	5,073
**Total No. of GO terms in the reference**	3,936	1,341	2,131	464
**No. of significant expanded orthogroups with GO terms**	23	16	12	4
**No. of significant GO terms in expanded orthogroups**	7	5	2	0

11. Supplementary Figures

Supplementary Fig. 1 Genome coverage distribution of the V1 (diploid, black) and V2 (diploid, red) assemblies, showing fewer regions with twice the expected coverage in the V2 assembly, i.e. better resolved haplotypes in the V2 assembly.

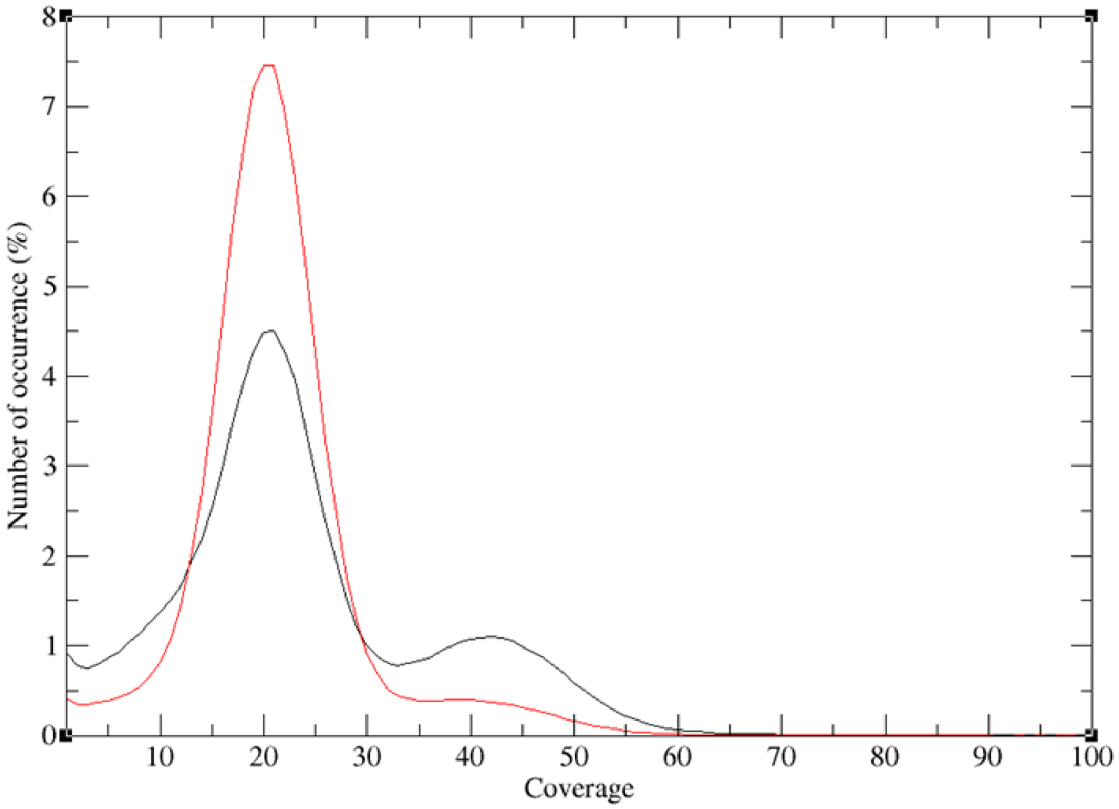

Supplementary Fig. 2 Illustration of the syntenome approach between *Prunus persica* and *Quercus robur*. Pedunculate oak scaffolds are anchored by oak markers (green scaffolds matching red dots of the oak genetic linkage map) or by peach gene models (purple scaffolds) or by both (combined green-purple scaffolds).

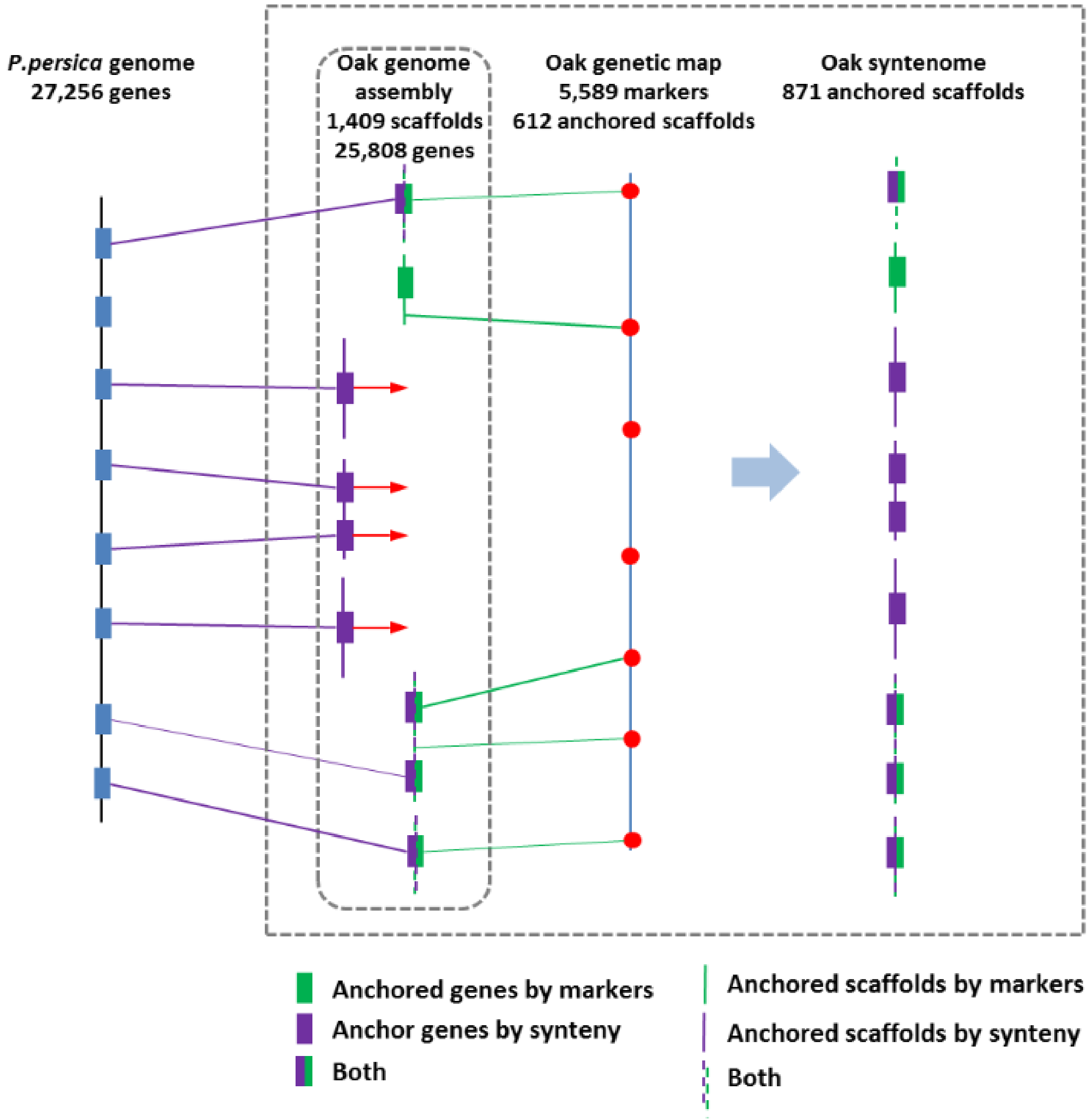

Supplementary Fig. 3 Distribution of TE families. Distribution of TE families according to: **(a)** their main order or superfamily (Gypsy/Copia) in the consensus library (1,750 consensus) and **(b)** their genome coverage (716,192 copies, 52% of the genome).

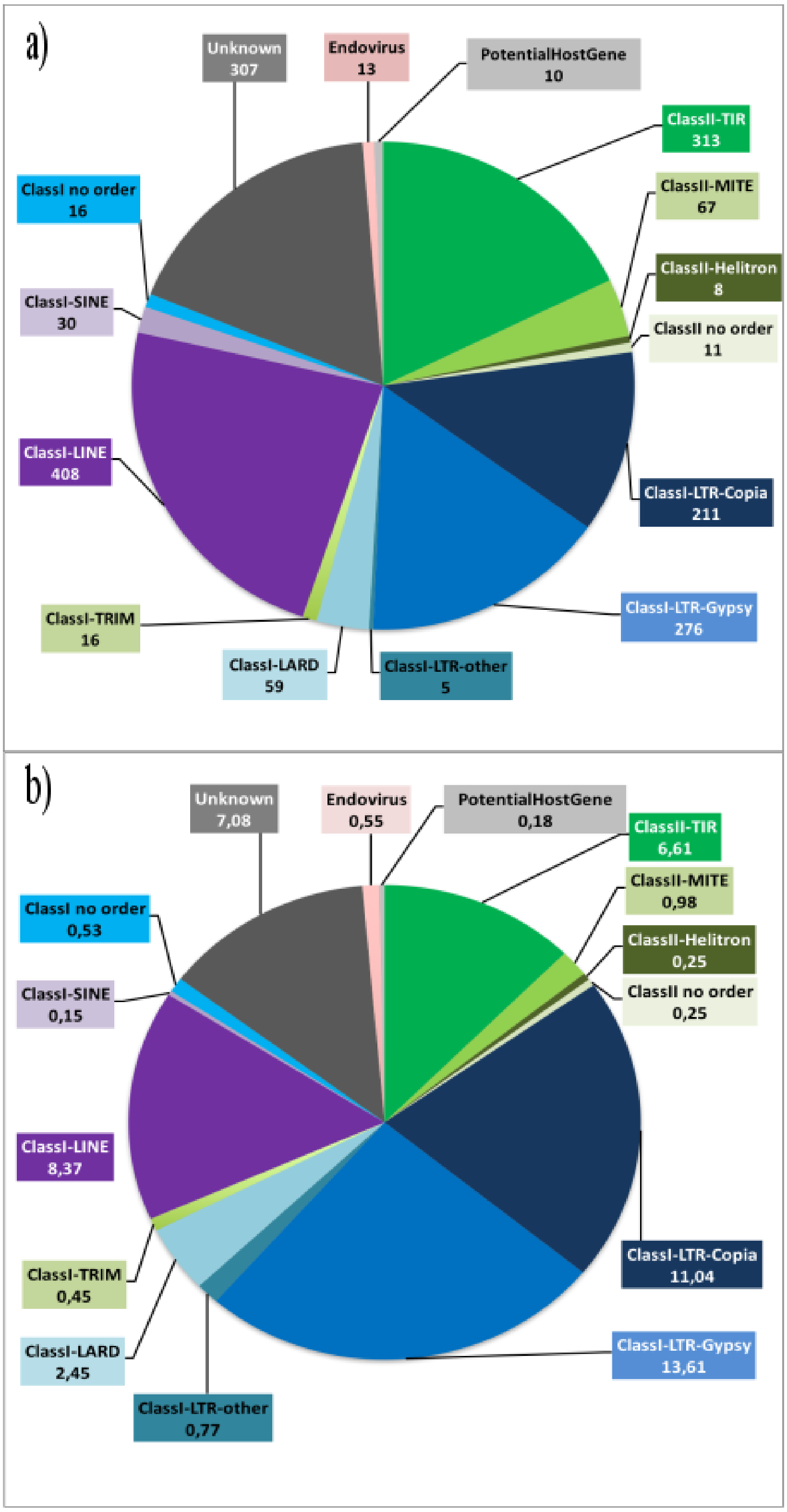

Supplementary Fig. 4 Chromosomal variations of genetic diversity. (**A**) Proportion of heterozygous sites within the "3P" reference genome sequence. (**B**) estimation of Tajima's π at the population level. Both metrics were calculated in 1 Mb windows, sliding by steps of 250 kb. Colors correspond to Tukey's Honestly Significant Difference criterion at the α = 0.05 significance level. Box plots were drawn with R with default parameters. The bottom and top of the box are the 25th and 75th percentile (the lower and upper quartiles, respectively), thus delineating the interquartile range (IQR). The band near the middle of the box is the 50th percentile (i.e. the median). For the ends of the whiskers we used the default box plot parameter for statistical dispersion in R (1.5*IQR). Below the figures the sample size (number of windows) and quantile values are provided for each chromosome and each metric.

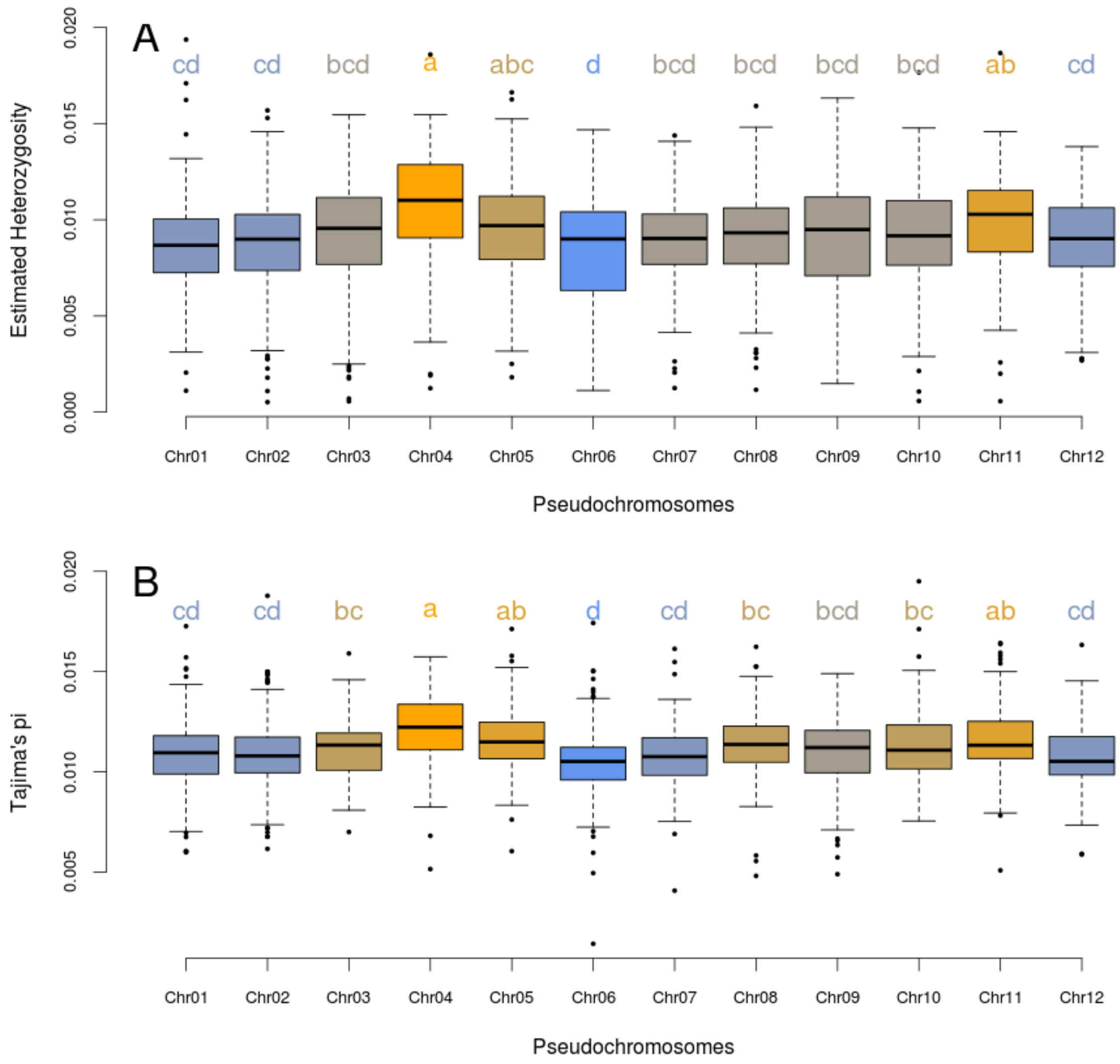

Sample sizes and qualile values.

**A**	chrom	**#windows**	1stcentile	5thcentile	10thcentile	1stquartile	median	3rdquartile	90thcentile	95thcentile	99thcentile
1	Chr01	**130**	0.002363013	0.004393948	0.005261262	0.007251443	0.008672124	0.010039868	0.011519258	0.012145932	0.016840459
2	Chr02	**266**	0.002100697	0.004298015	0.005754484	0.007370706	0.008988398	0.010277284	0.011669246	0.012467453	0.014217985
3	Chr03	**137**	0.001081677	0.002477953	0.004194525	0.007676995	0.009552104	0.011155354	0.012308122	0.012993091	0.013777254
4	Chr04	**99**	0.001895541	0.004651434	0.006457263	0.009059405	0.011011622	0.012866448	0.013945719	0.014408945	0.015519873
5	Chr05	**160**	0.002897904	0.004659509	0.005936392	0.00798619	0.009694981	0.011206569	0.012567117	0.013375598	0.015656735
6	Chr06	**131**	0.001494684	0.002640328	0.003820639	0.006321167	0.009001377	0.010412181	0.011392821	0.01235098	0.013901824
7	Chr07	**107**	0.002069173	0.004260246	0.005199583	0.007680156	0.009023406	0.0102929	0.011745387	0.012485407	0.014034843
8	Chr08	**177**	0.002690201	0.004800595	0.006018946	0.007705742	0.009324316	0.010612334	0.011557058	0.012273925	0.014481952
9	Chr09	**115**	0.001977248	0.00323089	0.004647822	0.007088226	0.009489949	0.011178917	0.012862547	0.014187532	0.015566817
10	Chr10	**119**	0.001263906	0.003383373	0.004767478	0.007632744	0.00916589	0.010998188	0.012691446	0.013874577	0.014762114
11	Chr11	**108**	0.002044077	0.004747339	0.006210409	0.008371558	0.010282012	0.011518549	0.012360799	0.013290434	0.014556657
12	Chr12	**90**	0.002745973	0.003568476	0.005181146	0.007615457	0.009013432	0.01060477	0.011471683	0.011952998	0.012647347

**B**	chrom	**#windows**	1stcentile	5thcentile	10thcentile	1stquartile	median	3rdquartile	90thcentile	95thcentile	99thcentile
1	Chr01	**145**	0.006036747	0.007824487	0.008626952	0.0098874	0.010948342	0.011802318	0.012579441	0.013850033	0.015472398
2	Chr02	**315**	0.007005252	0.008245111	0.00893641	0.009946993	0.01079225	0.011729033	0.013046817	0.013603244	0.014903649
3	Chr03	**144**	0.008221722	0.008864512	0.00936807	0.010093188	0.011332564	0.011924467	0.013027595	0.013648772	0.014547199
4	Chr04	**119**	0.007066025	0.009430628	0.009896027	0.011095004	0.012225594	0.013367221	0.013967201	0.014483707	0.015332212
5	Chr05	**180**	0.008182576	0.008781872	0.009904223	0.010659378	0.011487088	0.01247425	0.013389647	0.013723549	0.015578464
6	Chr06	**165**	0.005597463	0.007781344	0.008438614	0.009589428	0.010516228	0.01122136	0.012670056	0.013657986	0.015023686
7	Chr07	**126**	0.007055785	0.008025906	0.009054159	0.00982444	0.010753628	0.011695085	0.012823905	0.013157198	0.015324665
8	Chr08	**180**	0.00576823	0.008757703	0.009330757	0.010481886	0.011365739	0.01227747	0.013089583	0.013717592	0.015245776
9	Chr09	**139**	0.005964687	0.007555009	0.008606873	0.009947773	0.011206606	0.012067391	0.012986125	0.0136571	0.014526484
10	Chr10	**119**	0.008395567	0.009141206	0.009512838	0.010143959	0.011079144	0.012335854	0.013440607	0.014436443	0.016871645
11	Chr11	**135**	0.007864851	0.008833194	0.009467378	0.010663593	0.011324205	0.012517949	0.013841686	0.014949459	0.016224063
12	Chr12	**101**	0.005913755	0.007754588	0.008757966	0.009844794	0.010519347	0.011761386	0.012460929	0.012820039	0.014536021

Supplementary Fig. 5 Dating of branch insertion and bud sampling for DNA extraction. (**a)** Coring of a lateral branch at its insertion into the main trunk. (**b)** Wood core used to estimate the age of the lateral branch. (**c)** Bud sampling at the extremity of a lateral branch.

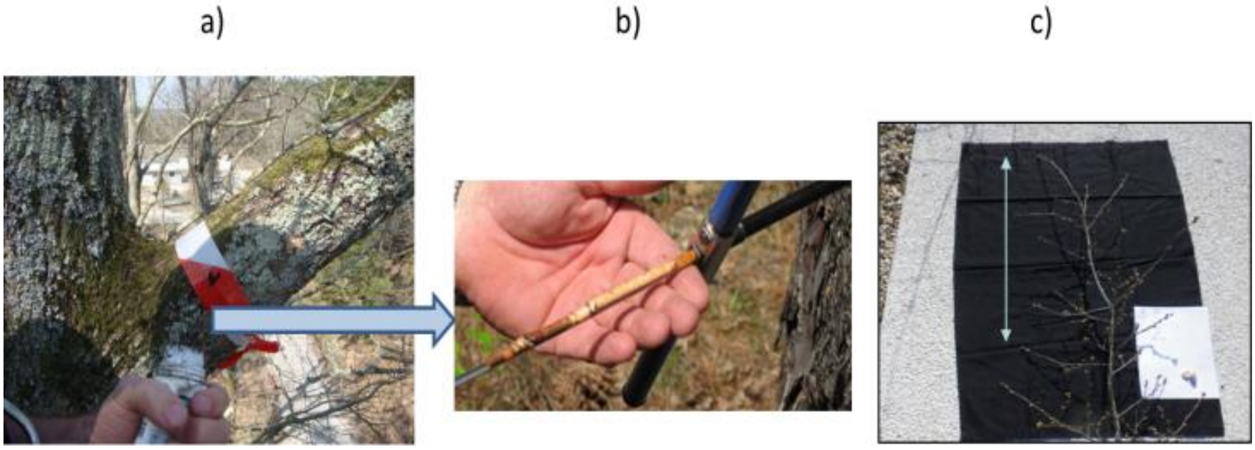

© Grégoire le Provost

Supplementary Fig. 6 Maximum likelihood phylogenetic analysis of TIR-NB-LRR-related genes. The NB domain of TNL-related genes (i.e., TNL, TNLX, NL, NLX, TN, TNX, TL, N) corresponding to orthogroup #1000 was used to study the relationship betwen selected tree (Cc, *Citrus clementina*; Cp, *Carica papaya*; Eg, *Eucalyptus grandis*; Md, *Malus domestica*; Pp, *Prunus persica*; Pt, *Populus trichocarpa*; Qr, *Quercus robur*; Tc, *Theobroma cacao*; Vv, *Vitis vinifera*) and herbaceous plant species (At, *Arabidopsis thaliana*; Al, *Arabidopsis lyrata*; Cl, *Citrullus lanatus*; Fv, *Fragaria vesca*; Gm, *Glycine max*; Rc, *Ricinus communis*; St, *Solanum tuberosum*), represented by brown and green branches, respectively. The oak TNL-related genes clades are shown as light brown squares. The red arrows in these blocks correspond to physical clusters along the genome, and chromosomes (Chr_x) or scaffolds (Scaf_x) are indicated (only the major clusters are indicated). The number of genes from different species falling into the major clades are shown (only the dominant species were counted for each clade). Bootstrap values over 70% and 80% (of 1000 replicates) are indicated by gray and black dots, respectively. Supported terminal nodes are not shown, to make the tree easier to read. The NB domain of APAF-1 was used as an outgroup to root the tree. Clades containing the NB domains of the TNL genes *Rpp4*, *Rpp5*, *Rps4* reported in *A. thaliana* are indicated.

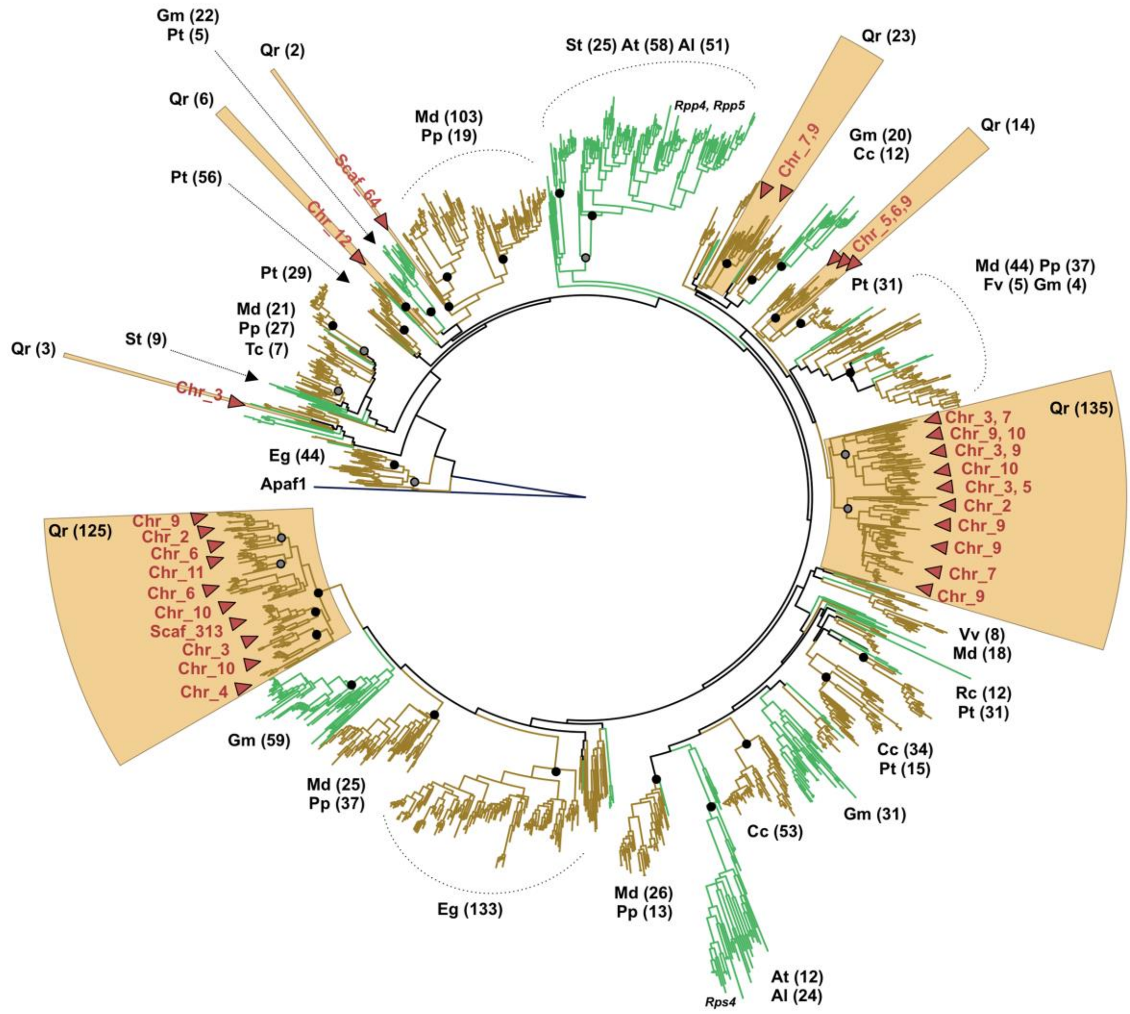

Supplementary Fig. 7 Fold-enrichment over background level (*x*–axis) of the significant gene ontology (GO) terms (*P*<0.01) of the orthogroups expanded in woody perennials. GO terms representing biological processes are shown as red lines, cellular components are shown in blue and molecular functions are shown in green. Sample sizes are provided in **Supplementary Data Set 8** sheet #5).

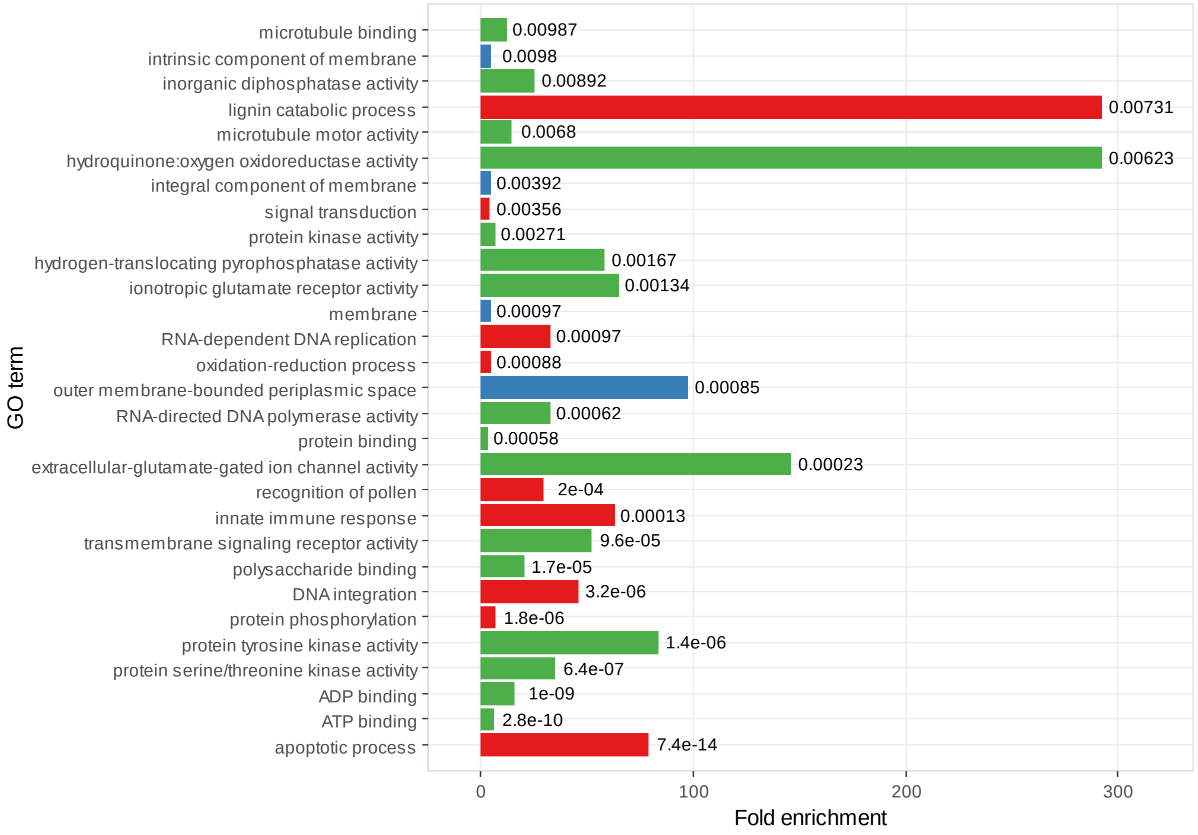

Supplementary Fig. 8 Number of amino acids under positive selection for each of the 24 positions of the LRR domain unit. L: Leu, x: variable, N: Asn, G: Gly, I: Ile, P: Pro.

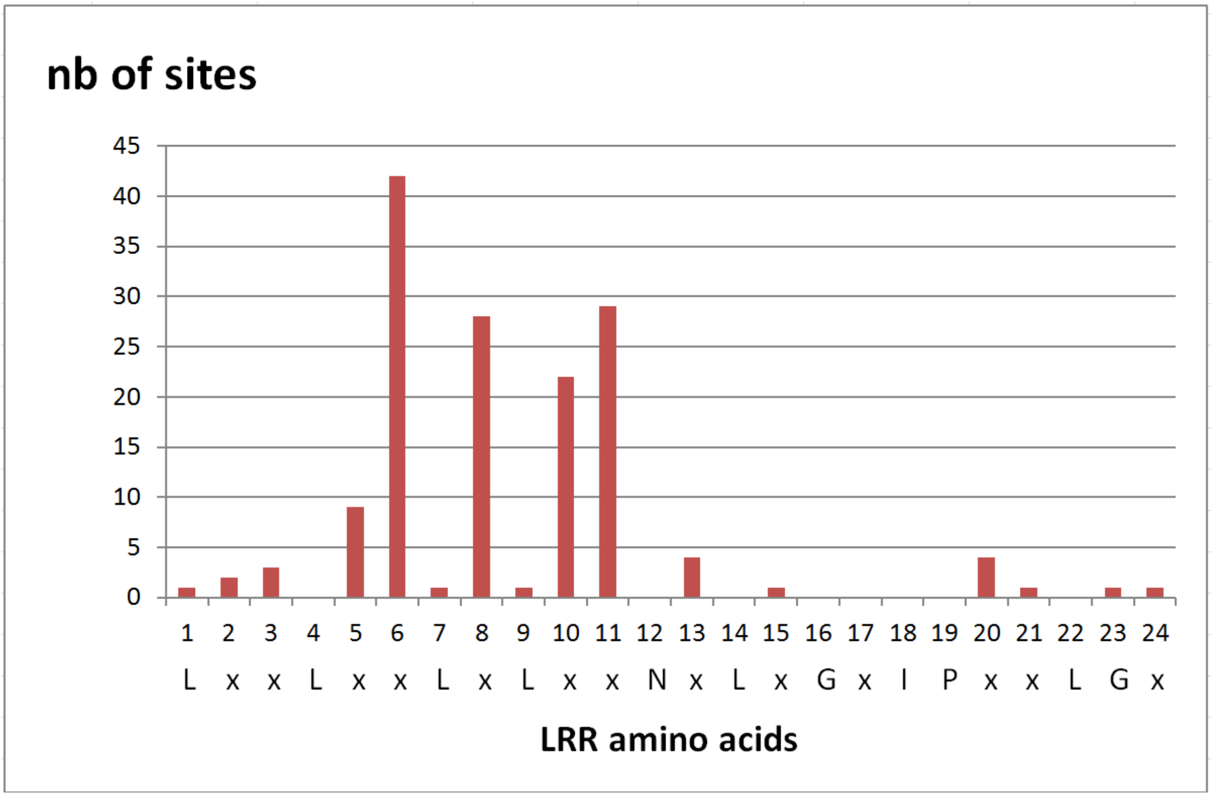

Supplementary Fig. 9 NUCmer alignment and dotplots of Cabog (A) and Newbler (B) scaffolds against two pedunculate oak BACs (177A20 and 107I07).

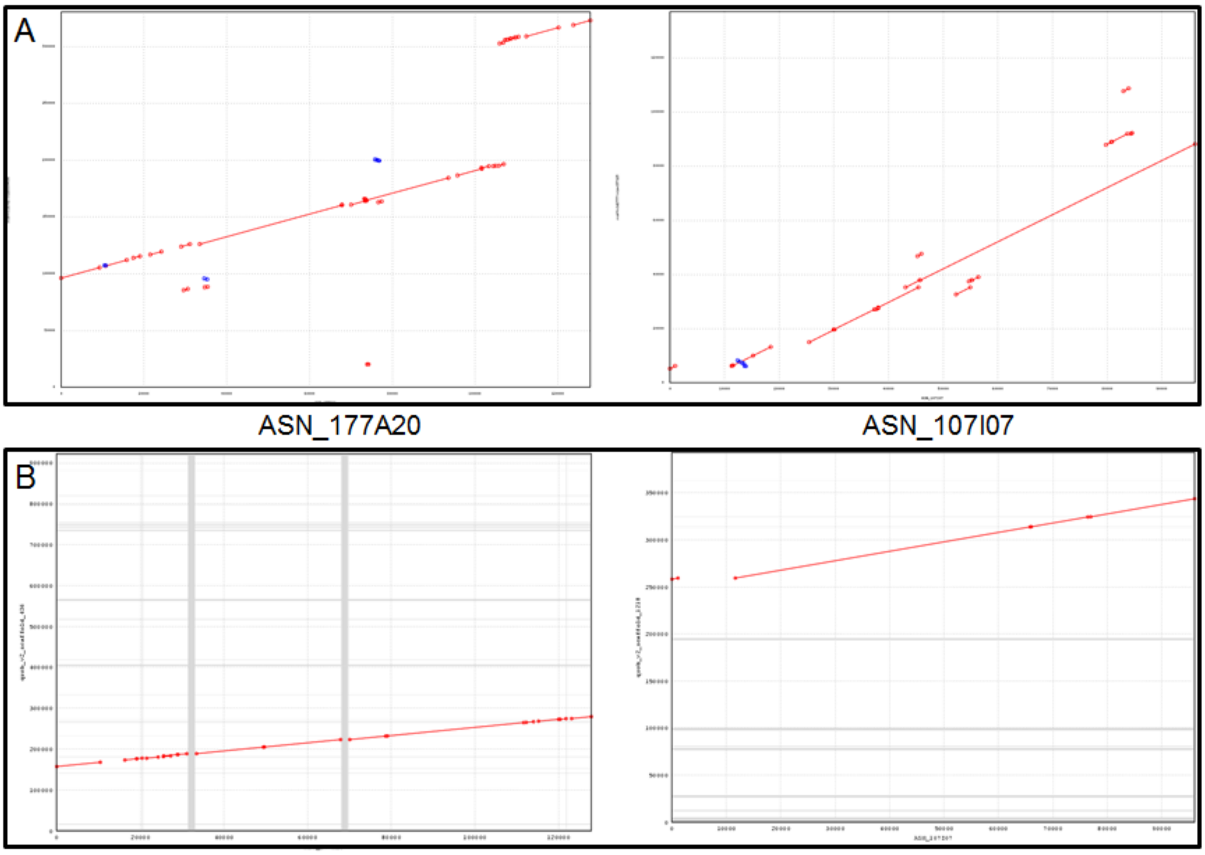

Supplementary Fig. 10 Generation of the haploid assembly (1n) of pedunculate oak with haplomerger. In general, both haplotypes are well separated in the 2n assembly (blue and orange haplotypes). For each aligned block (pink polygons), we retained only the longest sequence (haplotype blue or orange) as recommended by the creators of haplomerger, to maximize gene content. Scaffolds merging the two haplotypes (as Scaffold F) in the 2n assembly were retained, without modifications, in the 1n assembly.

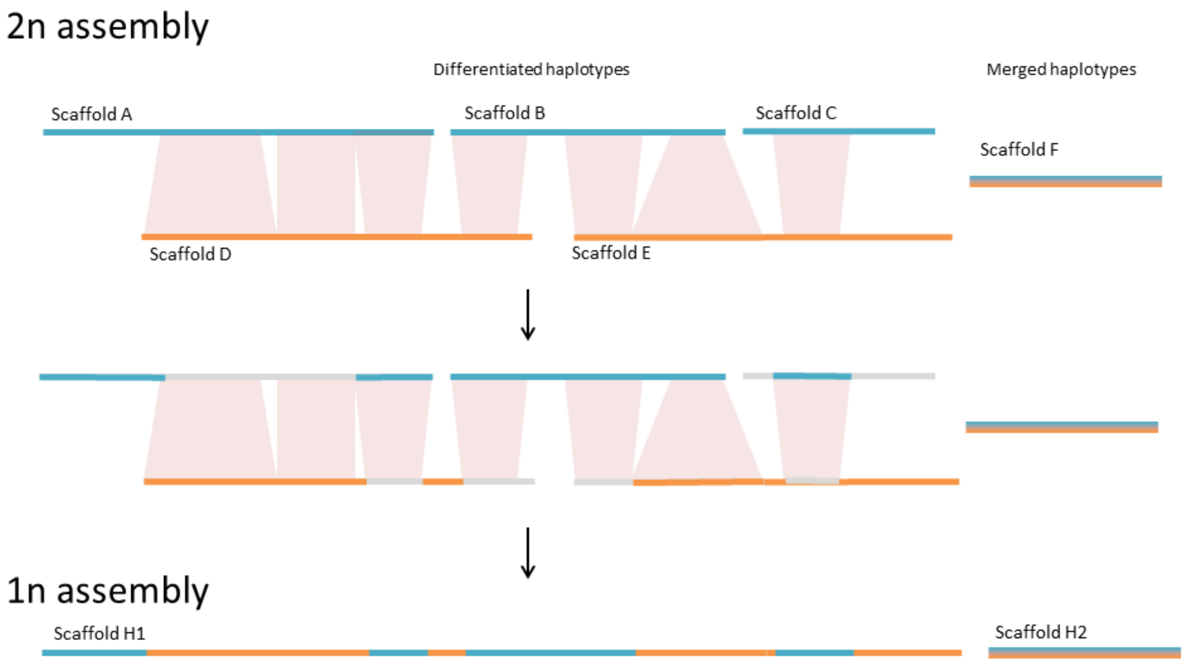

Supplementary Fig. 11 Alignment of two allelic BACs (#50E24 and #177A20) against the V2 diploid assembly (scaffold #0721 and #0436) and the V2 haploid assembly (Sc00000330). Gray boxes represent NUCmer alignments, blue rectangles correspond to SNPs specific to BAC #177A20 and red rectangles to SNPs specific to BAC #50E24. Green boxes correspond to flanking transposable elements.

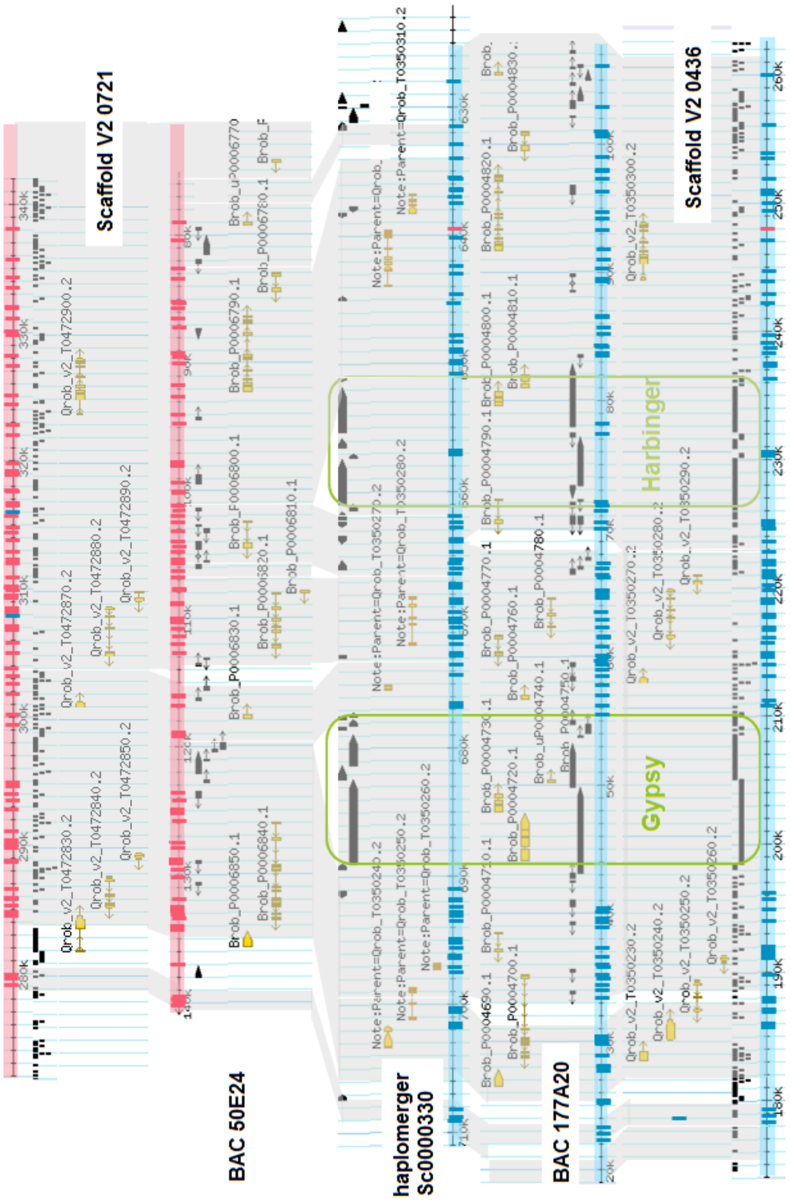

Supplementary Fig. 12 Alignment of two allelic BACs (#12J1 and #121F17) against the V2 diploid assembly (scaffold #0397 and #01822) and the V2 haploid assembly (Sc00000226). Gray boxes represent NUCmer alignments, blue rectangles correspond to SNPs specific to BAC #121F17 and red rectangles to SNPs specific to BAC #12J1. Green boxes correspond to flanking transposable elements.

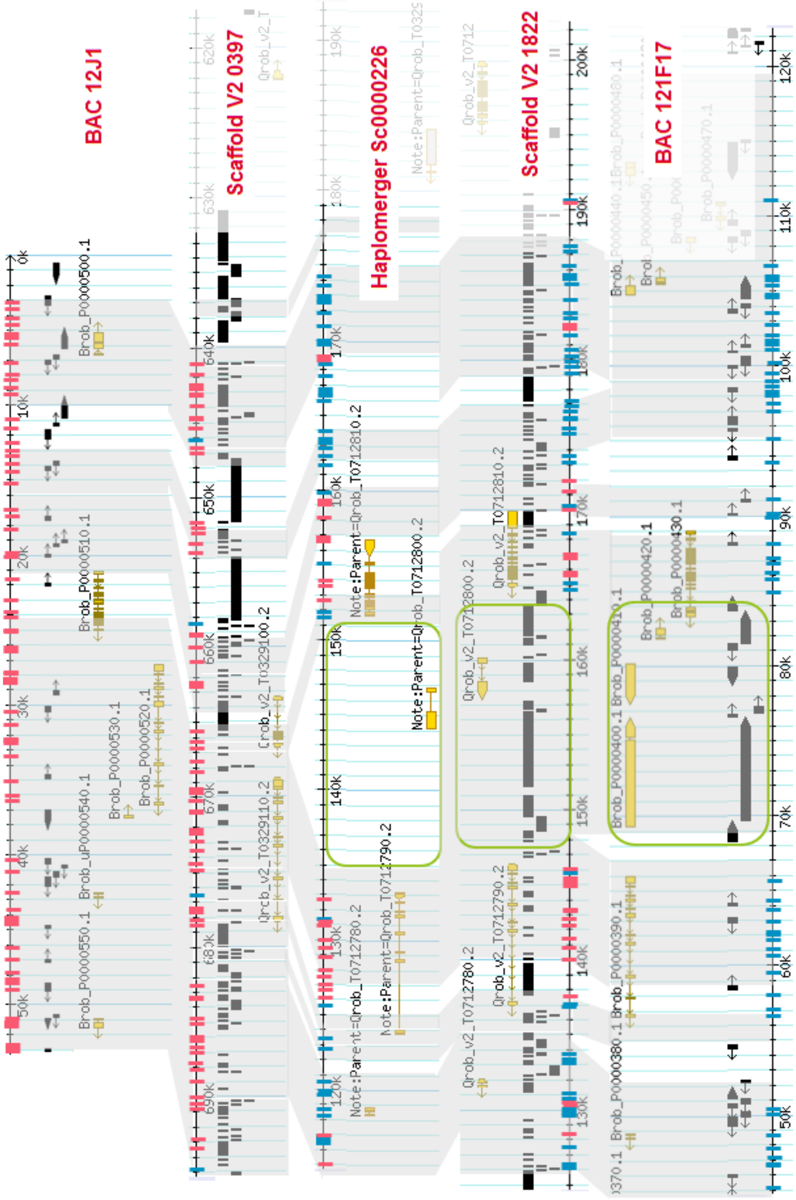

Supplementary Fig. 13 Alignment of the two allelic BACs (#107I07 and #5E10) against the V2 diploid assembly (scaffold #1218 and #0333) and the V2 haploid assembly (Sc00000189). Gray boxes represent NUCmer alignments, blue rectangles correspond to SNPs specific to BAC #107I07 and red rectangles to SNPs specific to BAC #5E10. Green boxes correspond to flanking transposable elements.

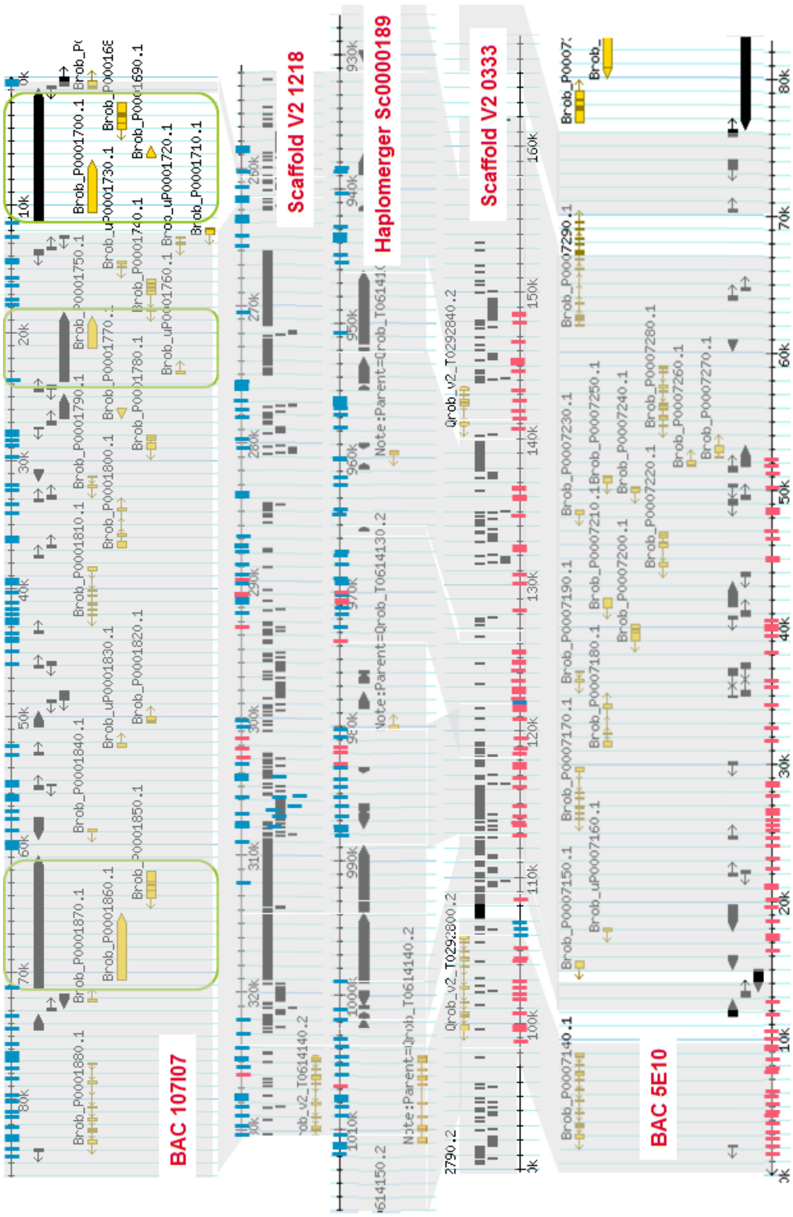

Supplementary Fig. 14 Region of the Qrob_H2.3_Sc0000378 scaffold containing a snoRNA cluster. Intergenic regions and snoRNAs are shown on separate lines. The name of the predicted snoRNA is given at the beginning of the predicted gene. For C/D box-predicted ncRNAs, the C and D motifs are colored in red, the terminal hairpin is colored in blue. For H/ACA box-predicted ncRNAs, the hairpins are colored in blue and green. The secondary structure is also indicated in parentheses. The ACA box is shown in red. Pink and blue shaded motifs at the beginning of the cluster represent putative RNA pol II promoter motifs (site II and TEF, respectively).

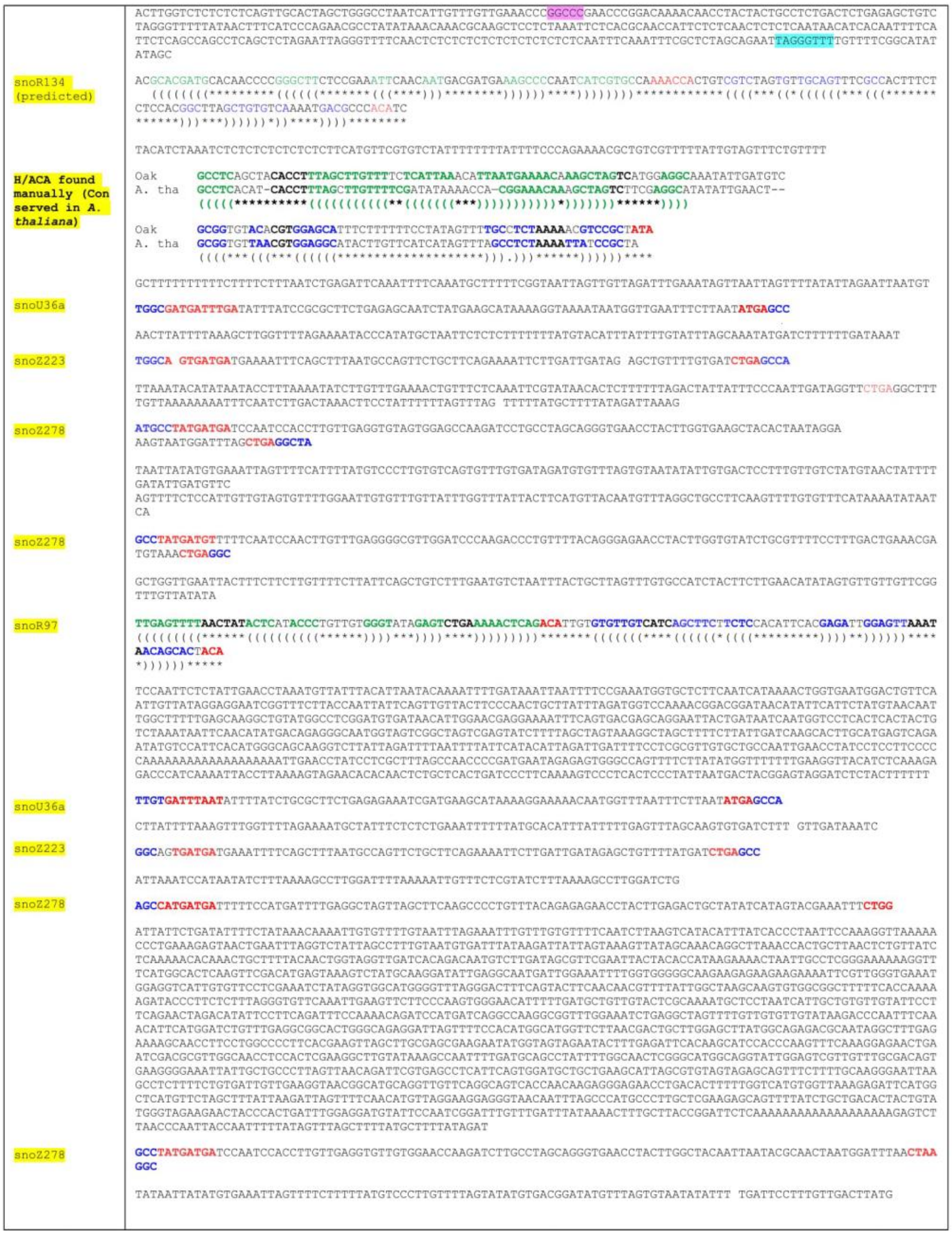

Supplementary Fig. 15 Genome-wide proportion of heterozygous sites (warm colors) and Tajima's π (cold colors) estimates. The proportion of heterozygous sites was calculated after calling heterozygous SNPs within the "3P" reference genome sequence by remapping all Illumina reads of this genotype. Tajima's π was estimated by a whole-genome sequencing strategy based on a pool of 20 pedunculate oaks. Both metrics were calculated in 1 Mb sliding windows moving in 250 kb steps.

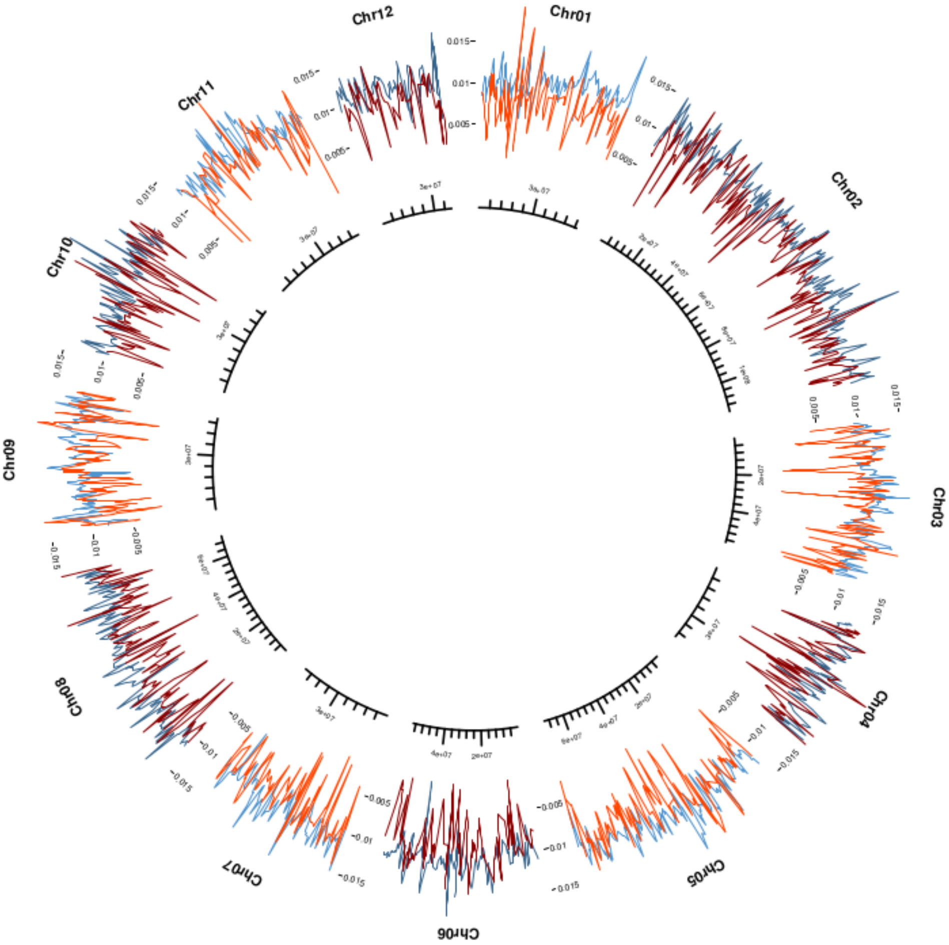

Supplementary Fig. 16 Dotplot comparison of oak-grape-peach-cocoa genomes. Dot illustration of grape-cocoa, grape-peach and grape-oak genome comparisons. Considering grape to be the closest modern representative of the n=21 rosid ancestor (derived from a post-γ ancestor with 7 protochromosomes shown in color on the *y*-axis of the dotplots), clear relationships are observed between the grape-cocoa (1:1), grape-peach (1:1) and grape-oak (1:1) genomes (see dotplot diagonals in each chart, shown with green circles), supporting the absence of lineage-specific polyploidization events in the considered species.

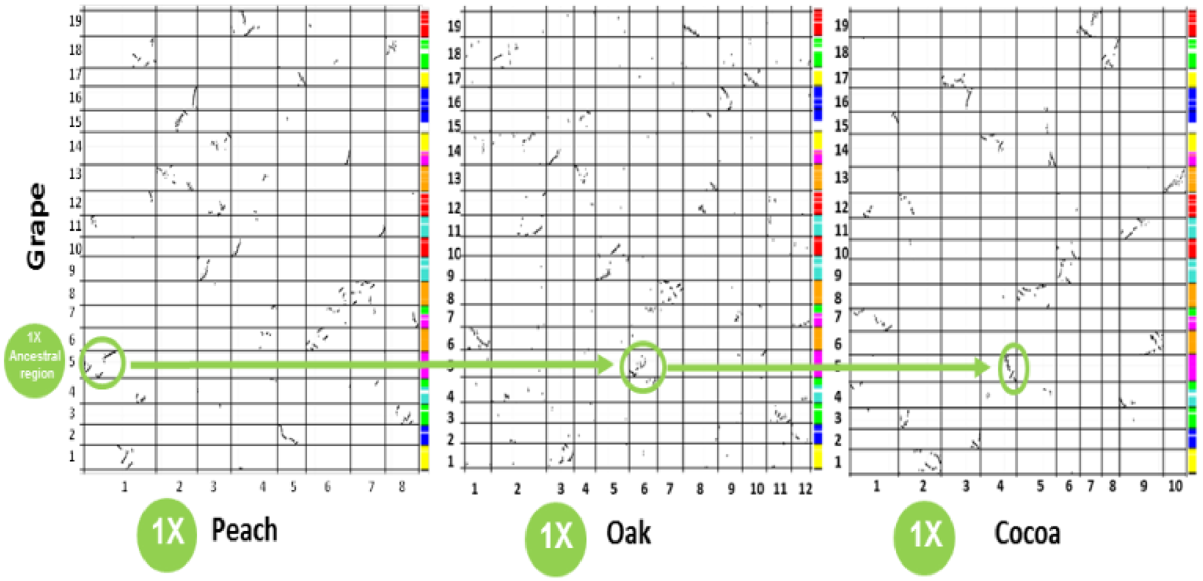

Supplementary Fig. 17 List of 16 plant species used for gene expansion contraction analysis in pedunculate oak. (**A**) Phylogenetic tree format of the 16 species used in orthoMCL/CAFE software. (**B**) Phylogenetic tree representation of the 16 species. Red dots correspond to branch specific whole genome duplication events. Species initials refer to **Supplementary Table 7**.

A

[(St:120,(Vv:117,(((Wa:101,((Md:53,(Pp:52,Fv:52):1):47,(Gm:99,Qr:99):1):1):1,(Rc:81,Pt:81):21):8,(Eg:109,(Cc:95,(Tc:87,(Cp:72,(At:5,Al:5):67):15):8):14):1):7):3)]

B

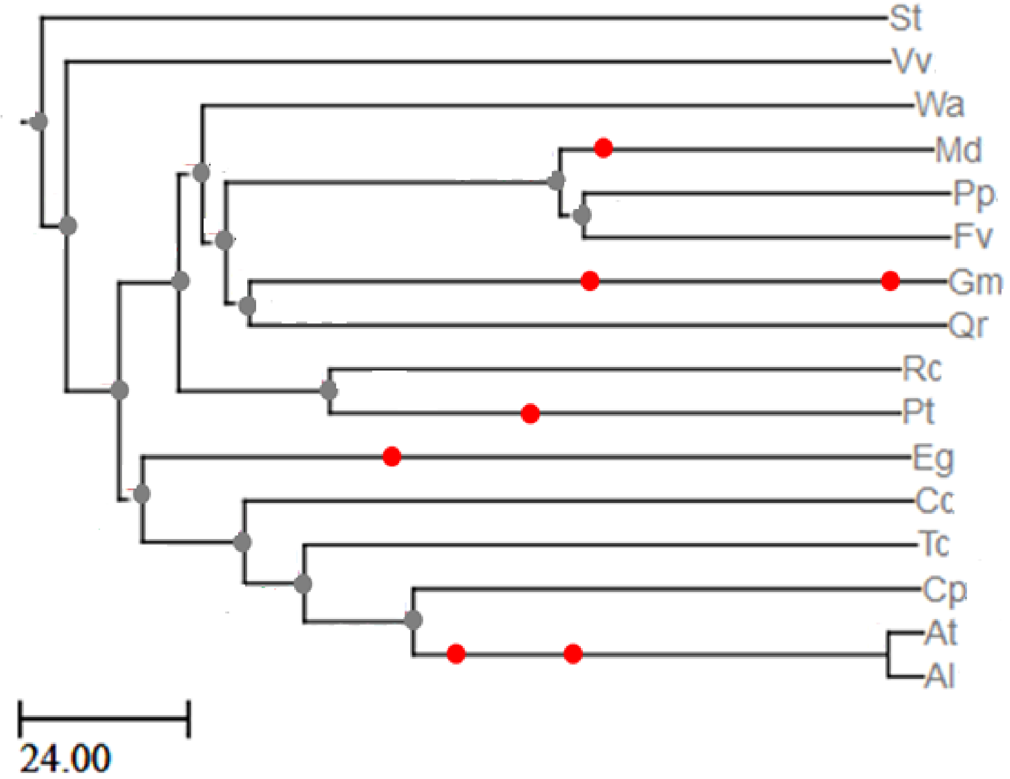

Supplementary Fig. 18 Distribution of the 524 orthogroups expanded in pedunculate oak across 15 plant species.

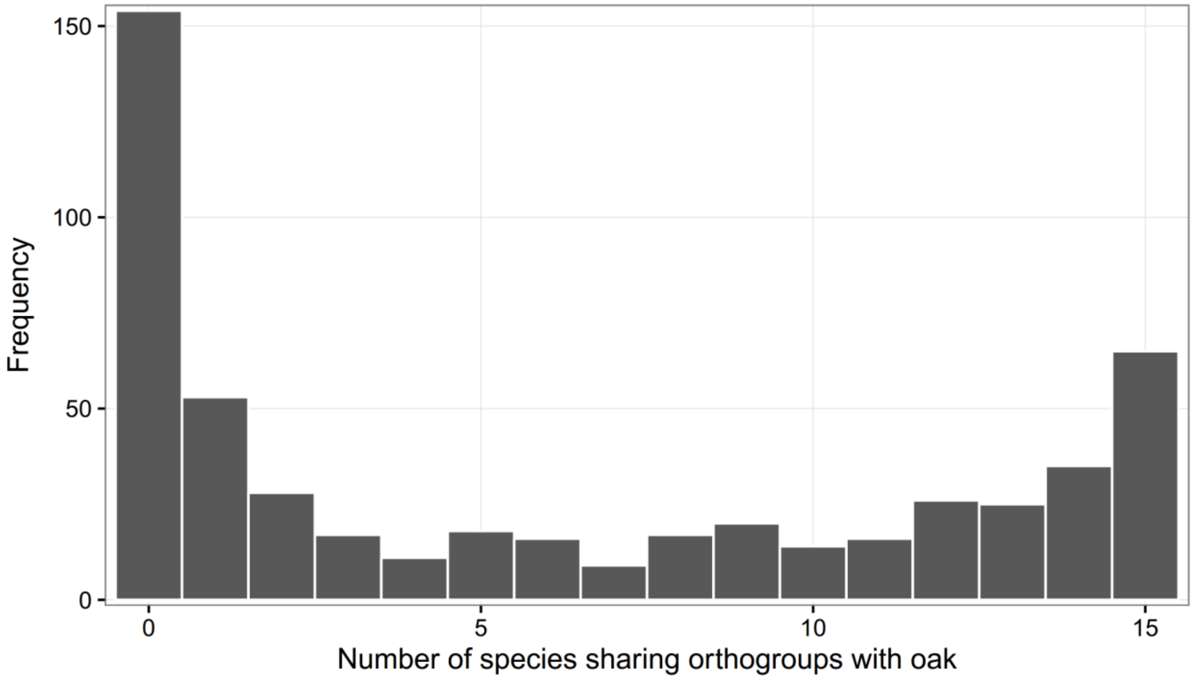

Supplementary Fig. 19 Proportion of genes classified as singleton genes (SGs, blue), tandem duplicated genes (TDGs, green) and long distance-duplicated genes (LDGs, red) in the 524 significant expanded orthogroups in pedunculate oak (PO).

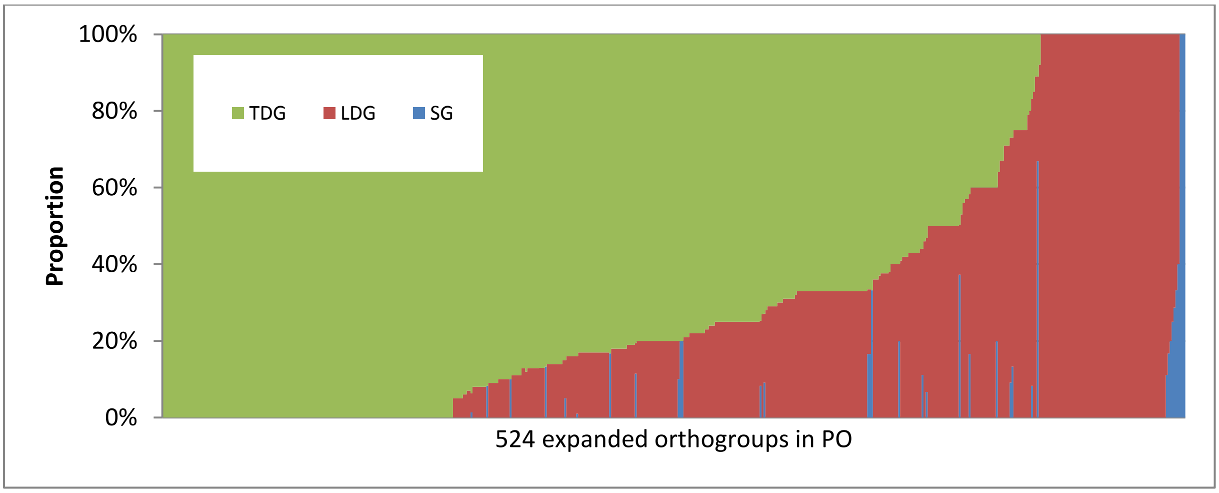

Supplementary Fig. 20 Identification of speciation and duplication events in the pedunculate oak genome. Illustration of the K_s distribution (*x*-axis) of gene pairs (*y*-axis) observed for oak (*Quercus robur*)/peach (*Prunus persica*) orthologs (green) and for grape (blue), peach (red), cocoa (brown) and oak (purple) paralogs. The oak/peach ortholog K_s distribution defines the position of the speciation event between these two species, with a single ancestral triplication event (γ) common to grape, peach, cocoa and oak and predating the speciation event. The burst of tandem duplicates highlighted by the purple K_s peak occurred after oak/peach speciation and appears to be an oak-specific event. Ks values for grape, peach and cocoa paralogous gene pairs were restricted to the γ triplication as a matter of comparison to the corresponding ancestral polyploidization event in oak.

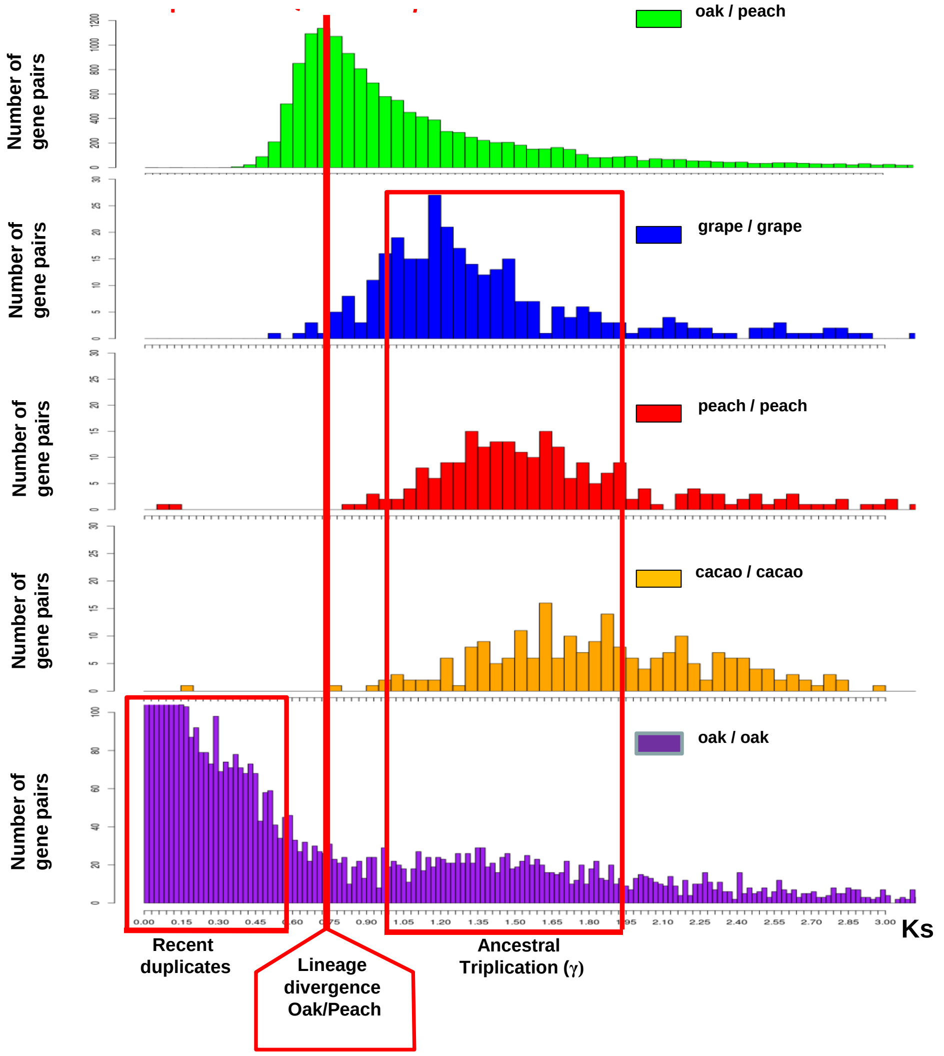

Supplementary Fig. 21 Dot plot representation of duplicates and extracted tandemly duplicated genes (TDGs). Dot plot representation of the pedunculate oak genome against itself for the complete set of paralogous pairs (left) and extracted TDGs (right).

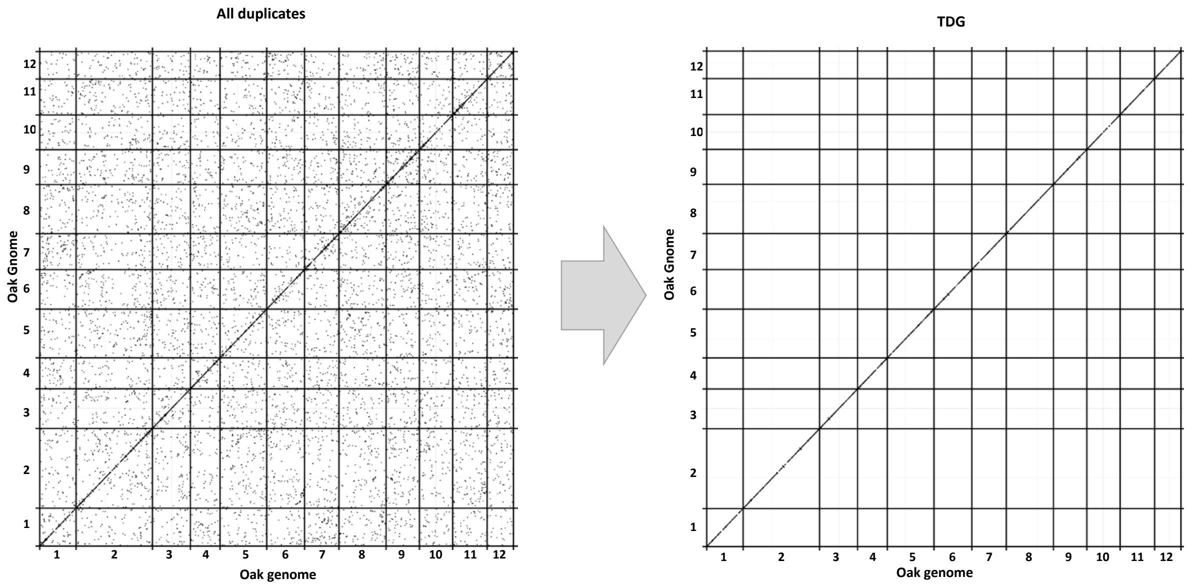

Supplementary Fig. 22 Validation of tandemly duplicated genes in pedunculate oak. (**A**) Distribution of the proportion of gap characters in the alignments. (**B**) Distribution of the proportion of variable sites (SNPs) in the alignments, expressed as a ratio of the number of variable sites to total alignment length, after the exclusion of gap positions. Below each plot, a box plot shows the 2.5th, 25th, 50th, 75th and 97.5th percentiles. Summary statistics for the 11,695 tandem duplicate pairs (black curve), and the 12,603 allelic pairs (light gray curve) identified by comparing the sets of genes in the diploid and haploid versions of the peduculate oak reference genome. Pairwise nucleotide sequence alignments were performed with MUSCLE.

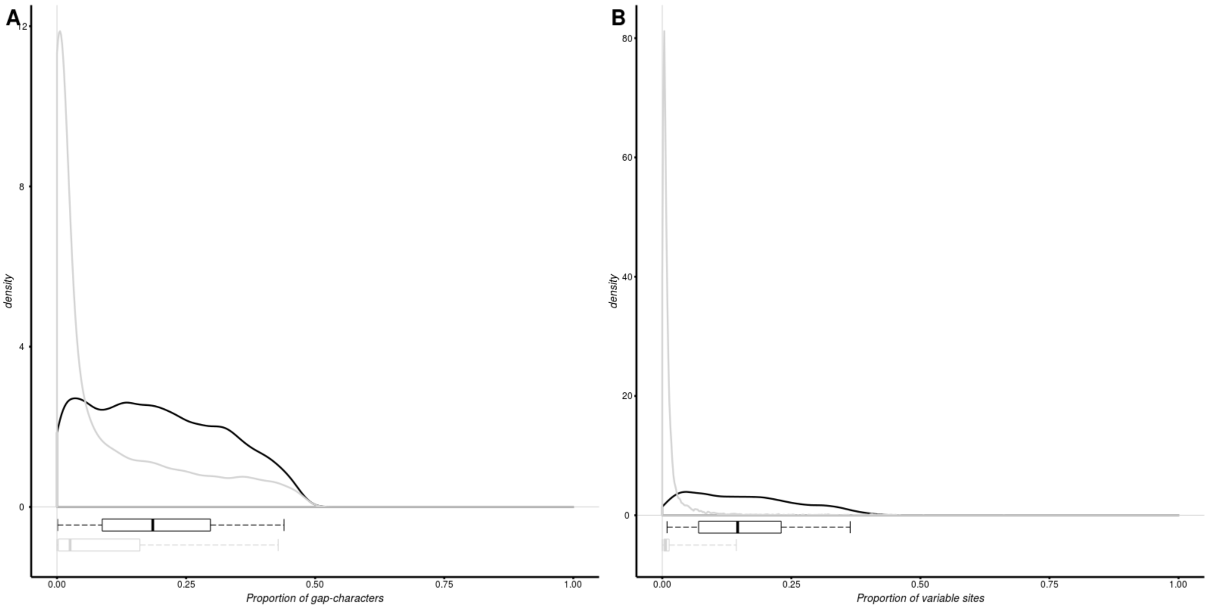

Supplementary Fig. 23 Genome coverage distribution of the longest scaffold (black) and coverage distribution of tandemly duplicated genes (red).

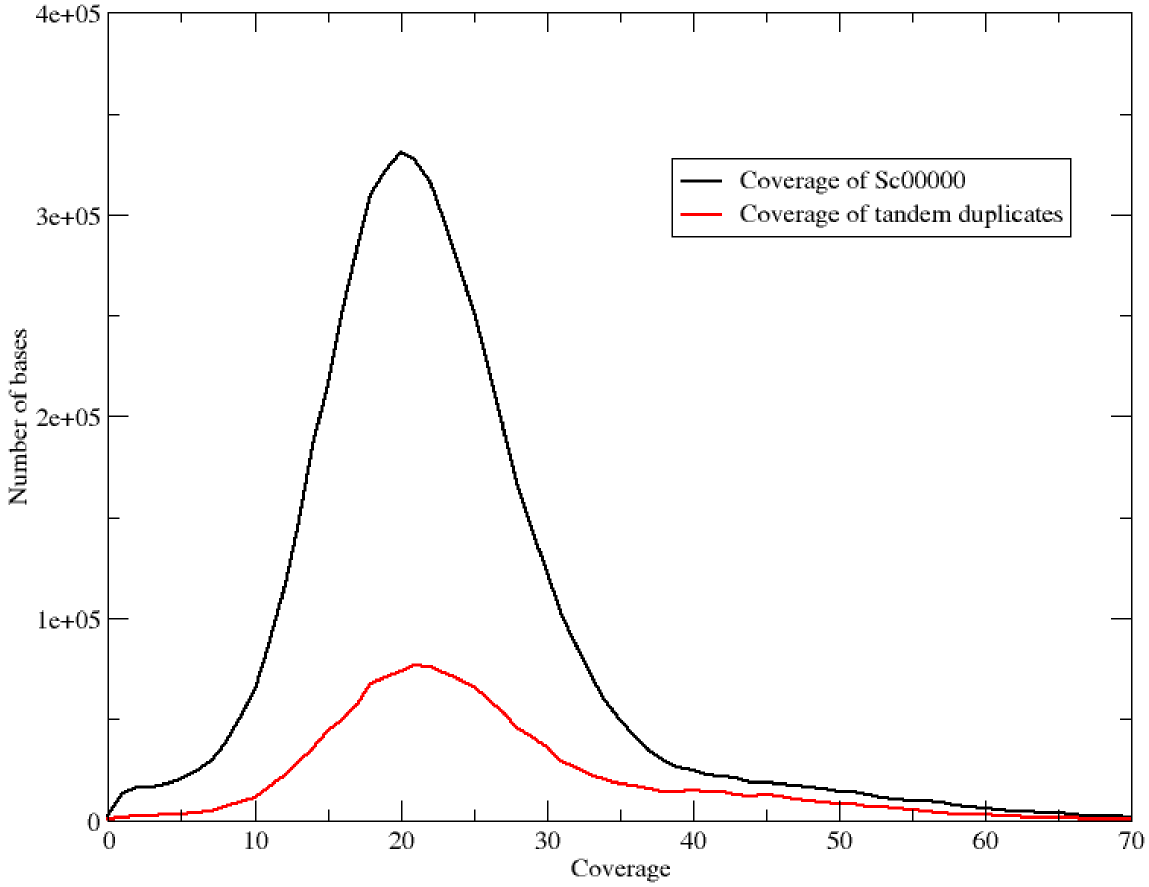

Supplementary Fig. 24 Box plot of the number of genes per orthoMCL cluster for each of the 16 species studied, including pedunculate oak. Species initials refer to **Supplementary Table 7**. Sample size for each species is indicated in **Supplementary Table 22**. A Tukey box plot was used. The bottom and top of the box are the 25th and 75th percentile (the lower and upper quartiles, respectively), thus delineating the interquartile range (IQR). The band near the middle of the box is the 50th percentile (i.e. the median). For the ends of the whiskers we used the default box plot parameter for statistical dispersion in R (1.5*IQR).

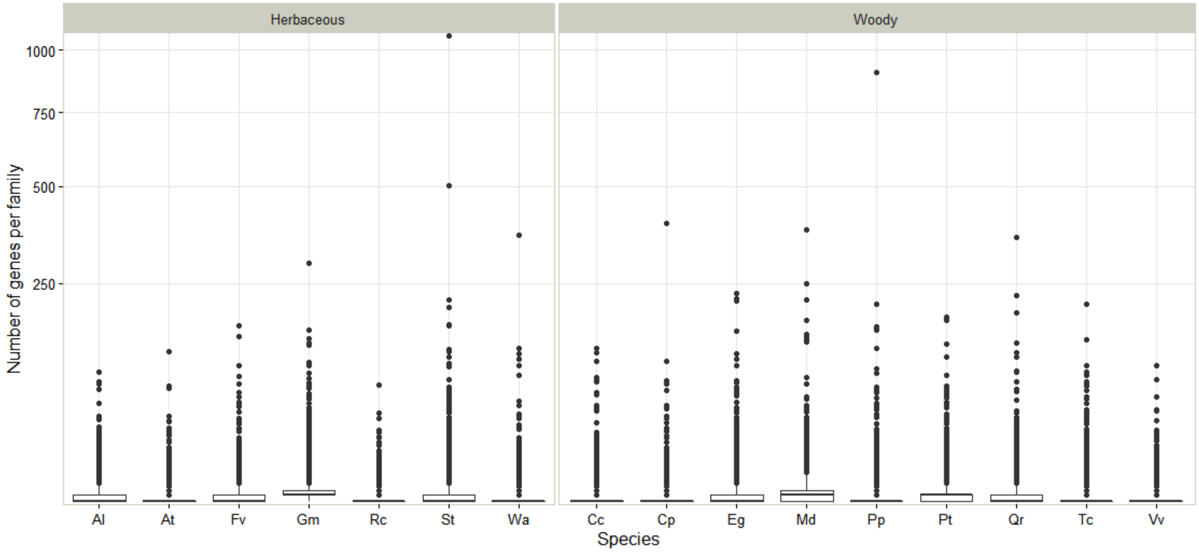

Supplementary Fig. 25 GenomeScope output generated from the 31-mers distribution. The size of the pedunculate oak haploid genome was at 736 Mb.

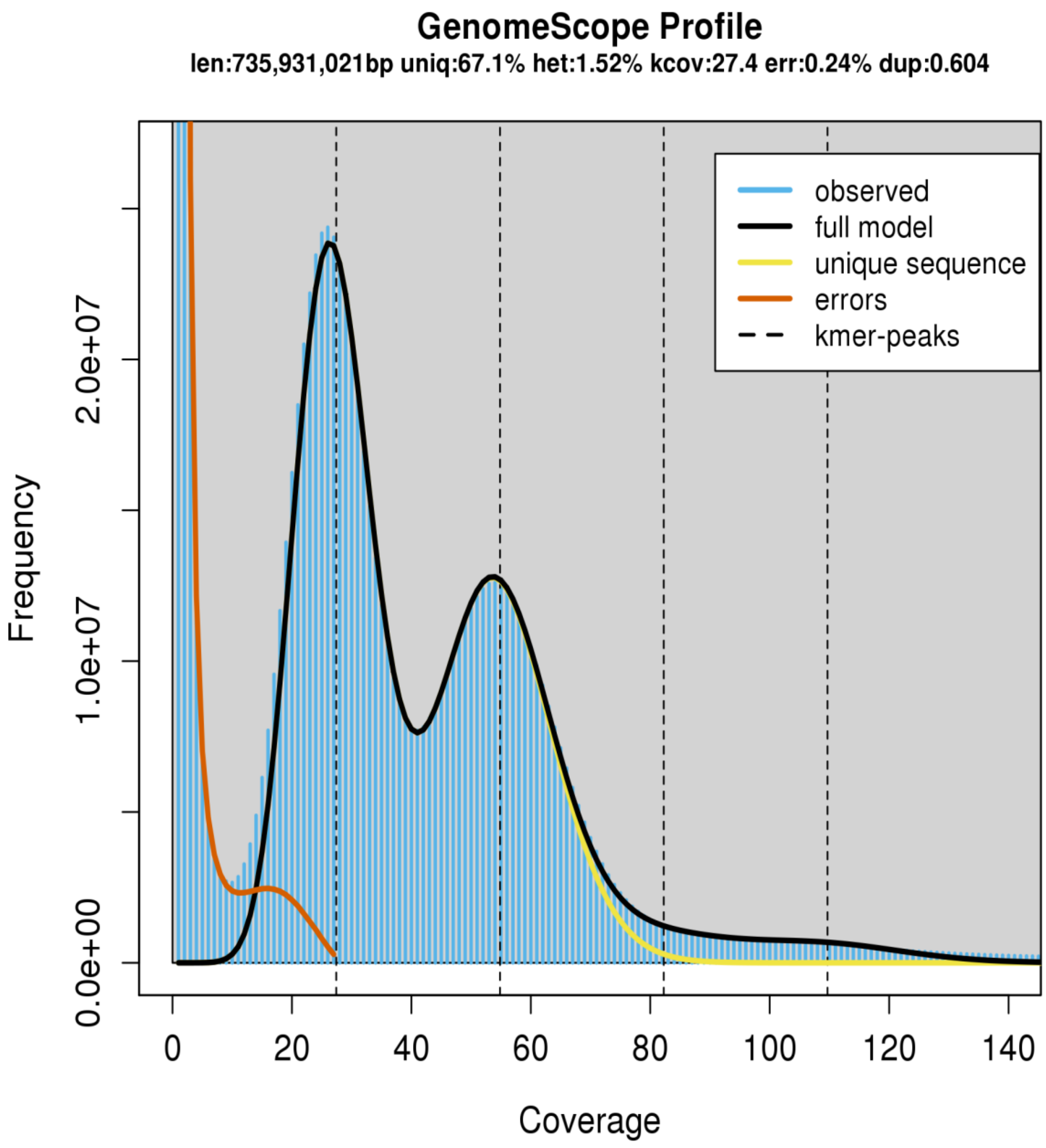

Supplementary Fig. 26 Comparison of allelic BAC structures of the reference Pedunculate oak genotype "3P". Manually curated genes are represented as green arrows with the head indicating the direction of transcription. Repetitive elements are represented as purple boxes. (**A**) 5E10_107I07, (**B**) 27L03_48K01, (**C**) 12J01_121F17, (**D**) 50E24_177A20, (**E**) 64H03_30P01.

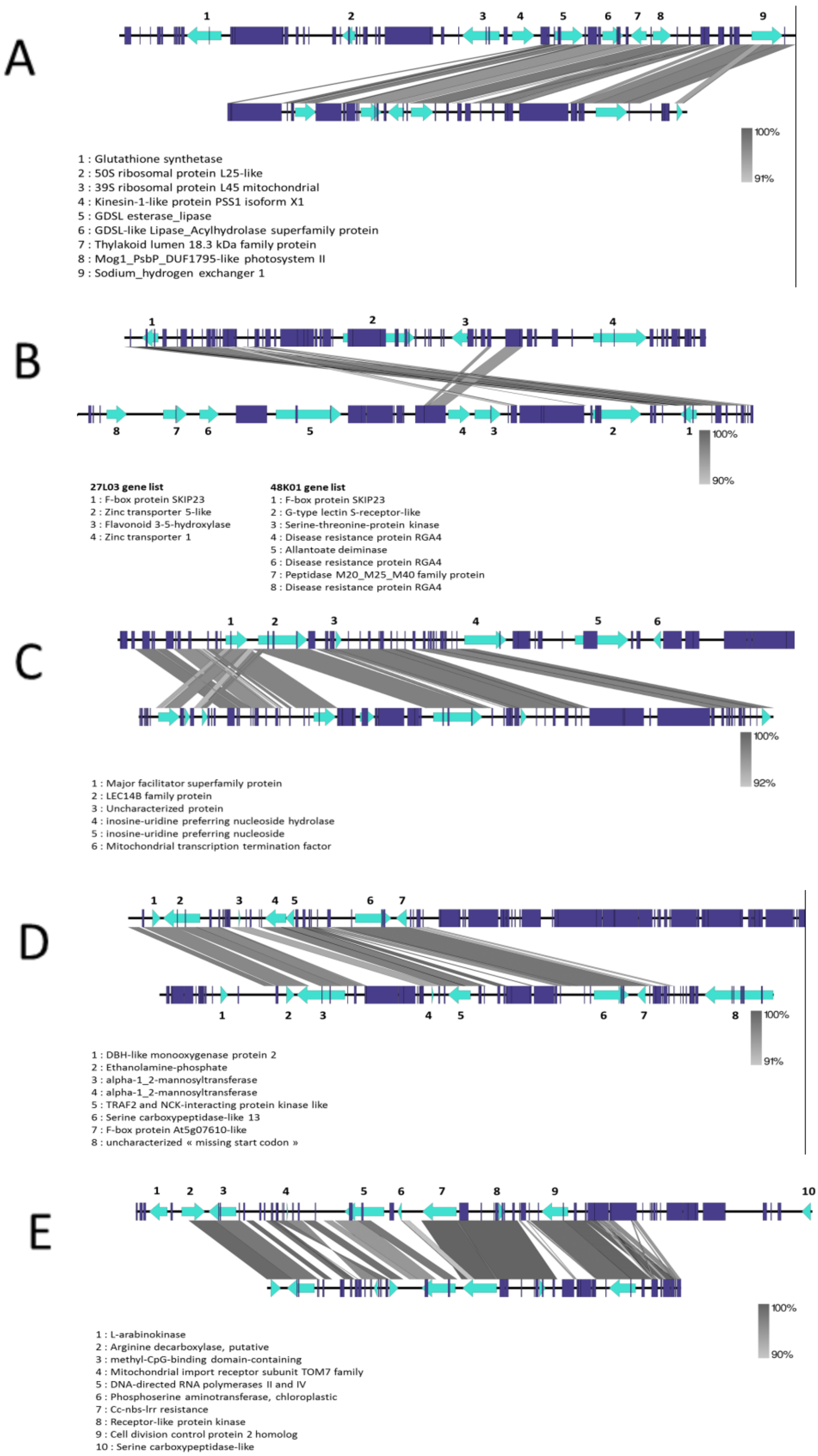

Supplementary Fig. 27 Alignment of two allelic BACs (#50E24 and #177A20) against the V1 diploid assembly (scaffold #1466). Gray boxes represent nucmer alignments, blue rectangles correspond to SNPs specific to BAC #177A20 and red rectangles to SNPs specific to BAC #50E24. Green boxes correspond to flanking transposable elements.

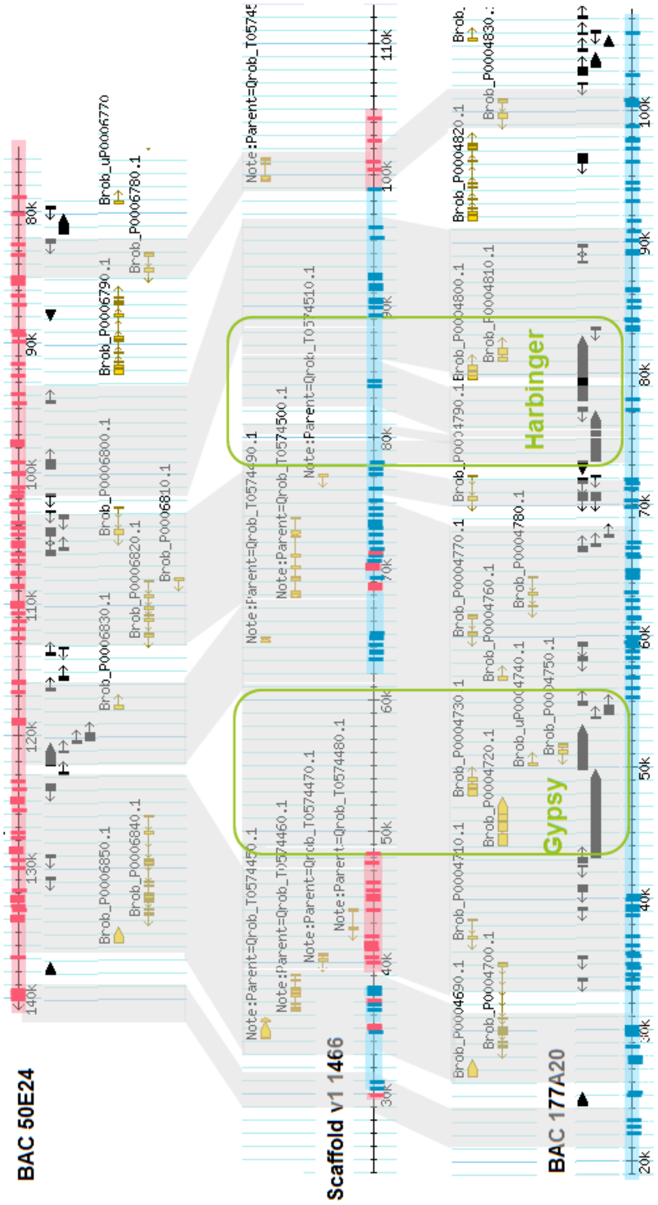

Supplementary Fig. 28 Alignment of two allelic BACs (#12J1 and #121F17) against the V1 diploid assembly (scaffold #3597). Gray boxes represent NUCmer alignments, blue rectangles correspond to SNPs specific to BAC #121F17 and red rectangles to SNPs specific to BAC #12J1. Green boxes correspond to flanking transposable elements.

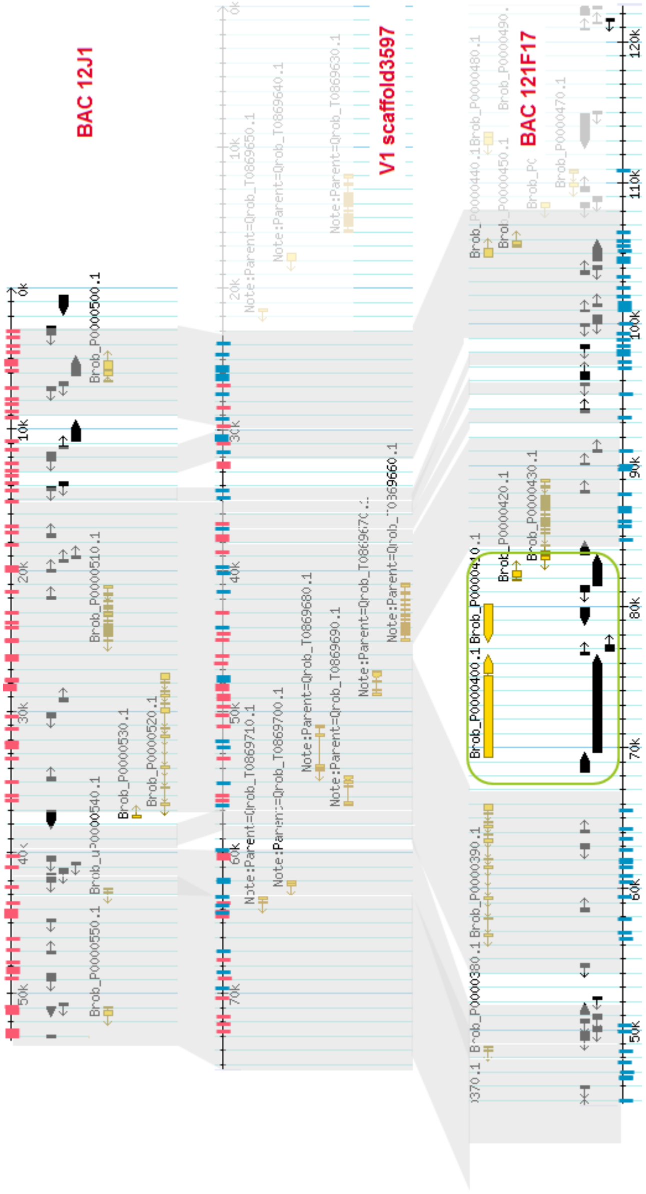

Supplementary Fig. 29 Alignment of two allelic BACs (#107I07 and #5E10) against the V1 diploid assembly (scaffolds #282 and #1030). Gray boxes represent NUCmer alignments, blue rectangles correspond to SNPs specific to BAC #107I07 and red rectangles to SNPs specific to BAC #5E10. Green boxes correspond to flanking transposable elements.

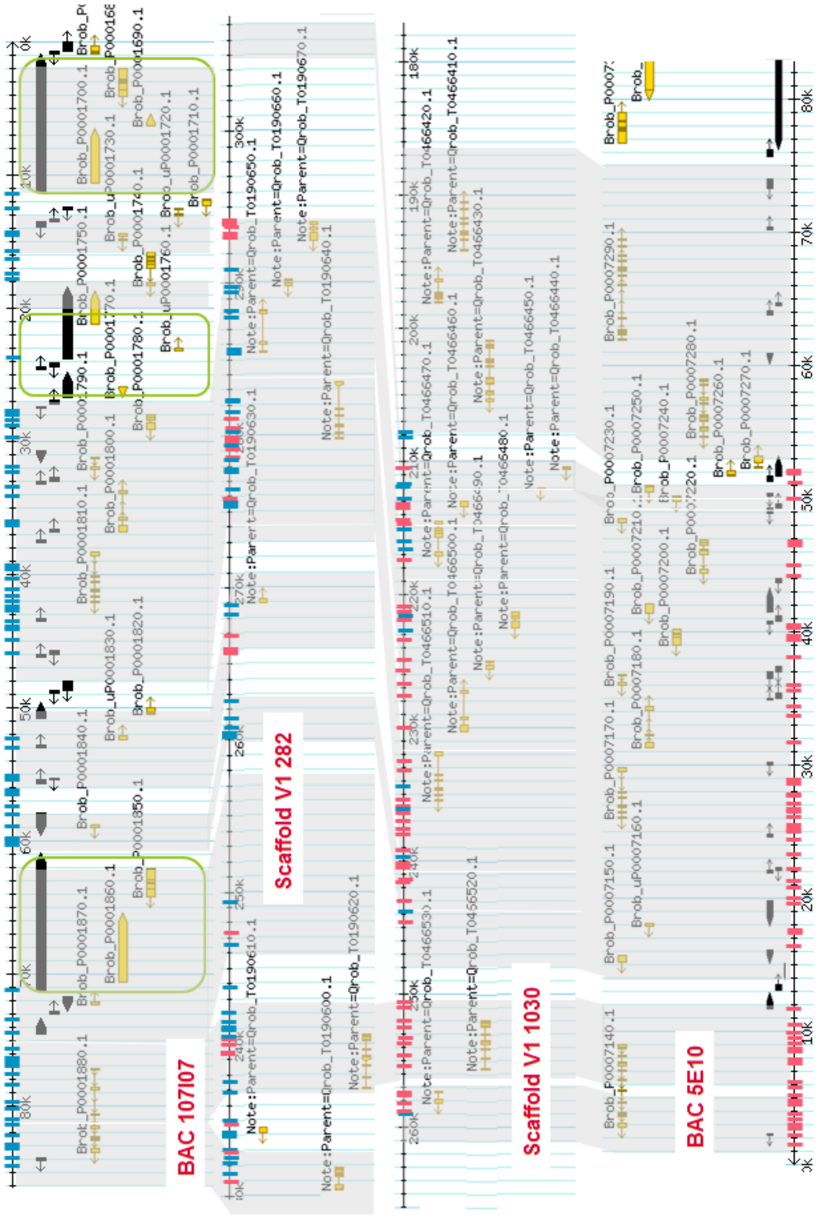

Supplementary Fig. 30 Flow chart indicating the procedure leading to the identification of the 4,070 mapped markers (2,127+1,943) of the oak genome browser "marker" track.

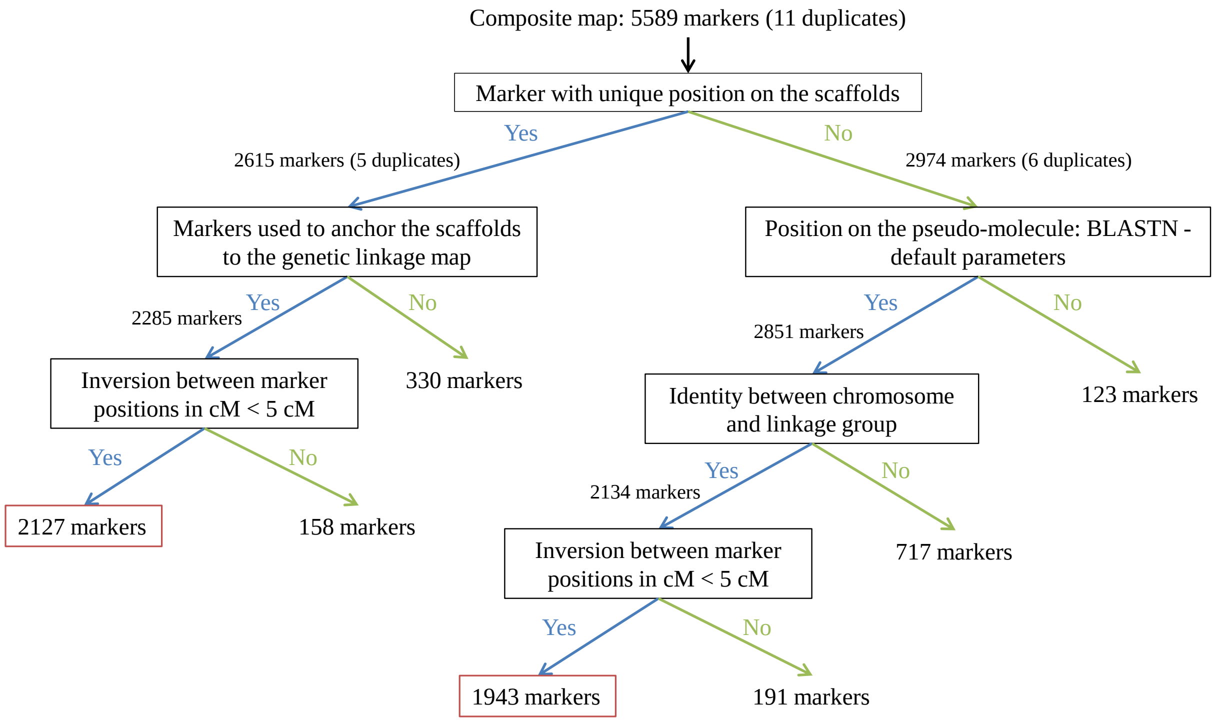

Supplementary Fig. 31 High-density genetic linkage map of the pedunculate oak genome (5,589 markers) showing the map positions of the 4,070 markers aligned on the 12 chromosomes, with possible inversion tolerated within a 5 cM interval.

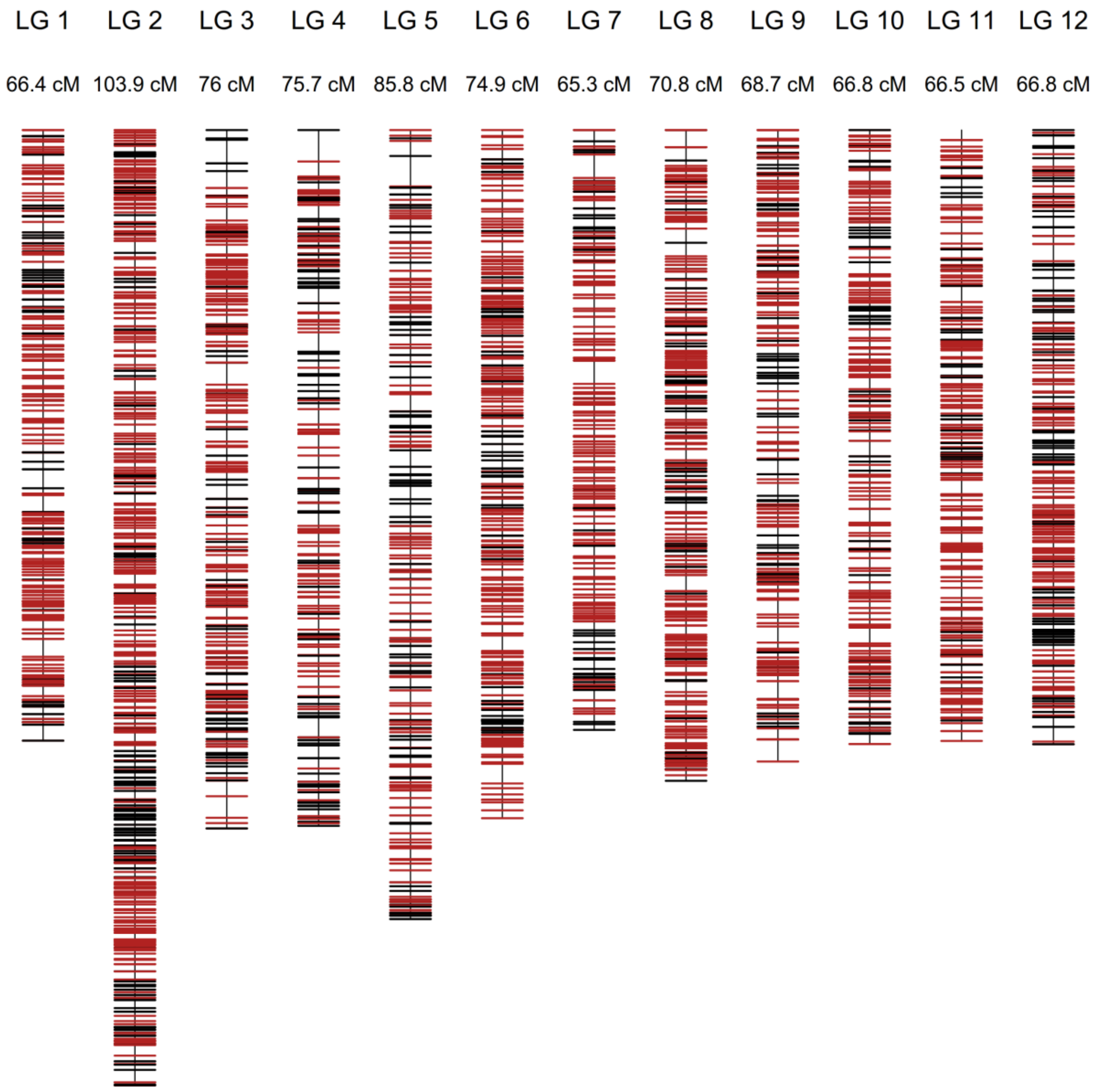

Supplementary Fig. 32 Physical – genetic relationships. Left panels- Physical position (in Mb on the haplome) and genetic location (in cM on the composite linkage map) for 4,070 markers used to populate the “marker” track of the pedunculate oak genome browser. Inversions between marker assignments on the genetic and physical maps are tolerated within a 5 cM window. Set#1 and set#2 markers from **Supplementary Data Set 3 sheet #1** are indicated by blue and red dots, respectively. Right panels- recombination rate along the 12 chromosomes (chr 1-12).

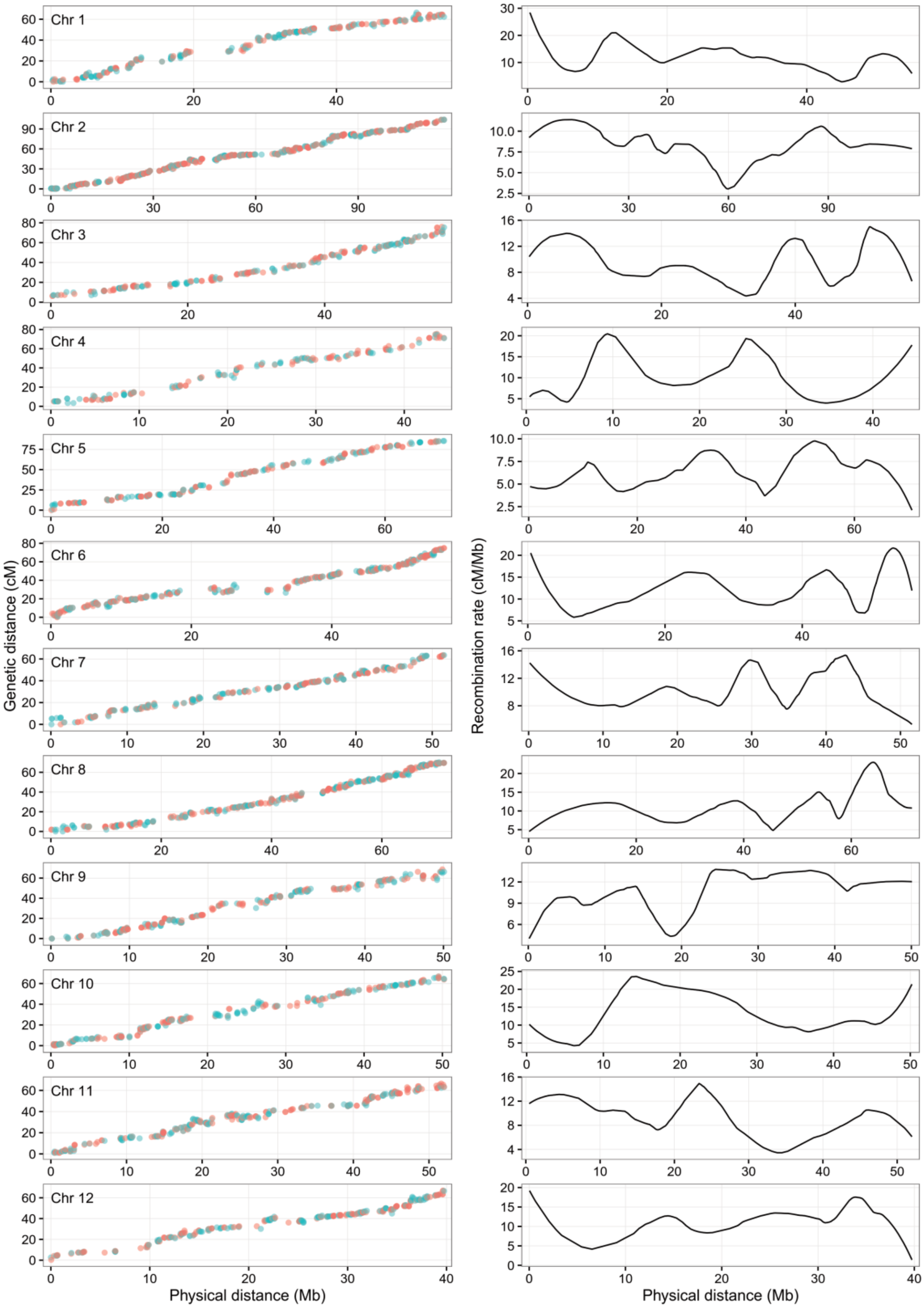

Supplementary Fig. 33 TE *vs* non-TE content in the 16 sequenced genomes considered in this study. The total in Mb (x-axis) corresponds to the fraction of the genome annotated. The tree on the left was generated with the NCBI Taxonomy Browser (www.ncbi.nlm.nih.gov/Taxonomy/CommonTree/wwwcmt.cgi). Only the topology is shown and the branch lengths are not proportional to evolutionary divergence time.

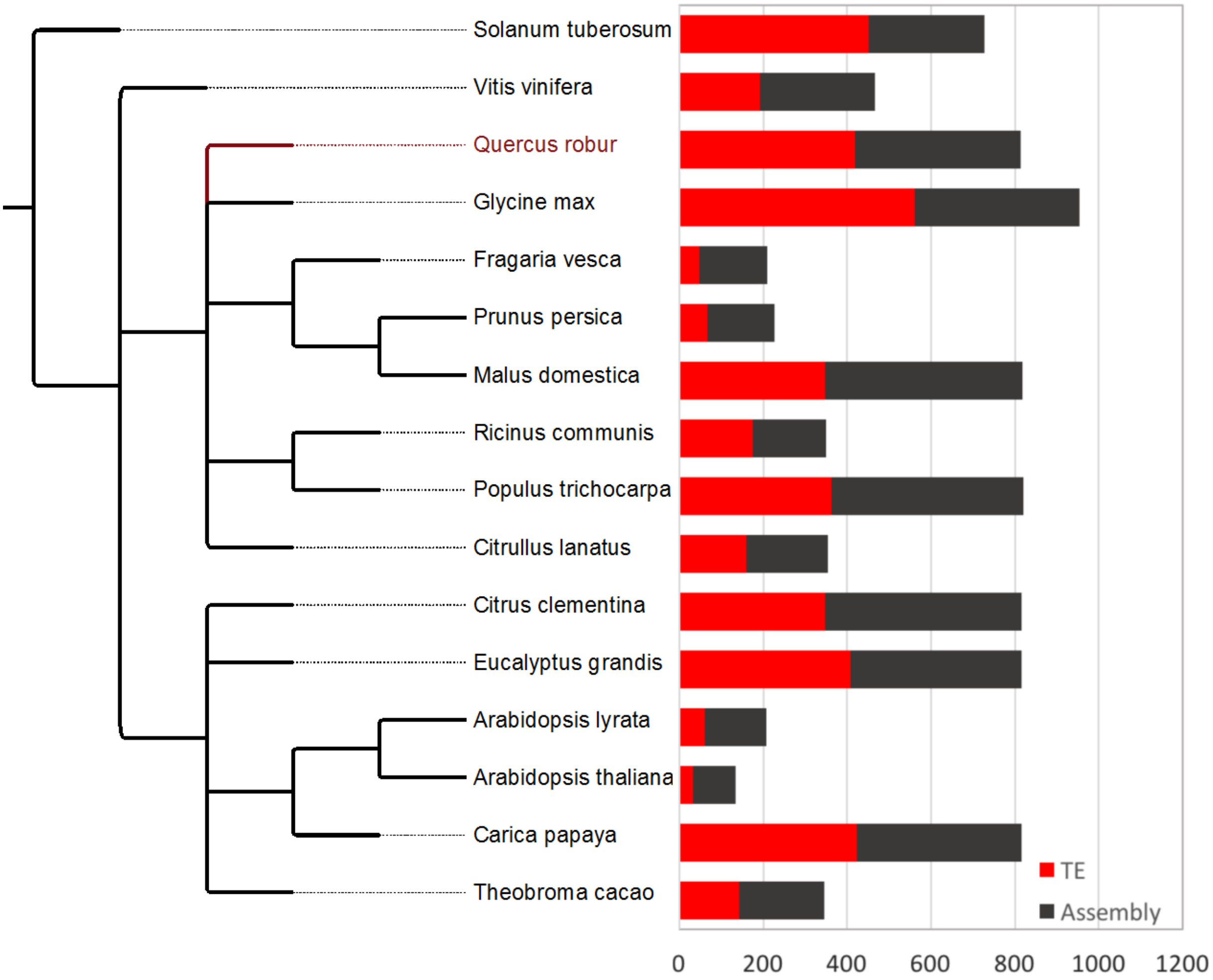

Supplementary Fig. 34 Endogenous viruses in the pedunculate oak genome. Phylogenetic reconstruction from the multiple sequence alignment of 58 reverse transcriptase domains from representative member from endogenous *Caulimoviridae* RTs found in the oak genome (blue branches, n=8), reference RT sequences from eight *Caulimoviridae* genera (n=41), Gypsy LTR retrotransposons (n=5), and from mammalian endogenous retroviruses (ERV, n=4).

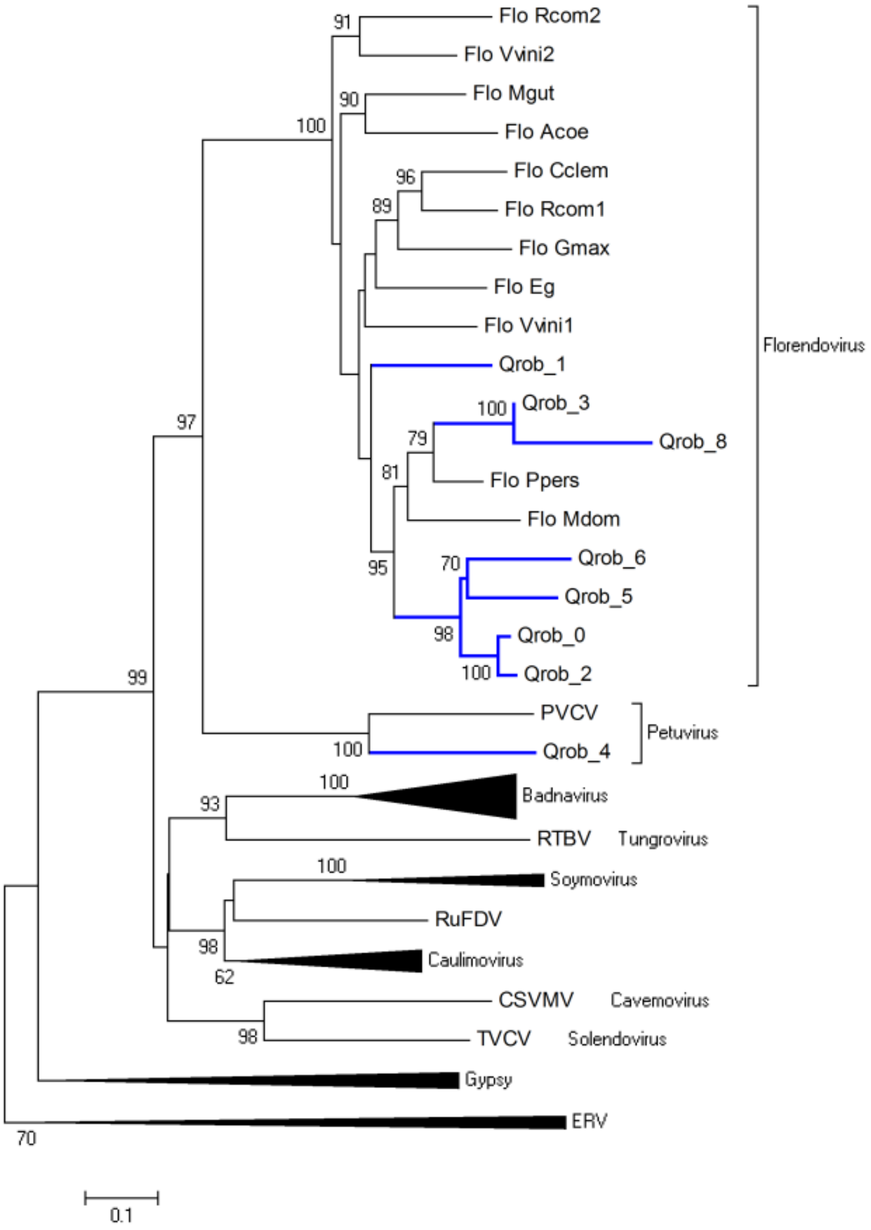

Supplementary Fig. 35 Highly repeated fragments of viruses. Overview of the multiple sequences alignment of 762 highly similar fragments (raw data) from Caulimoviridae found in the pedunculate oak genome.

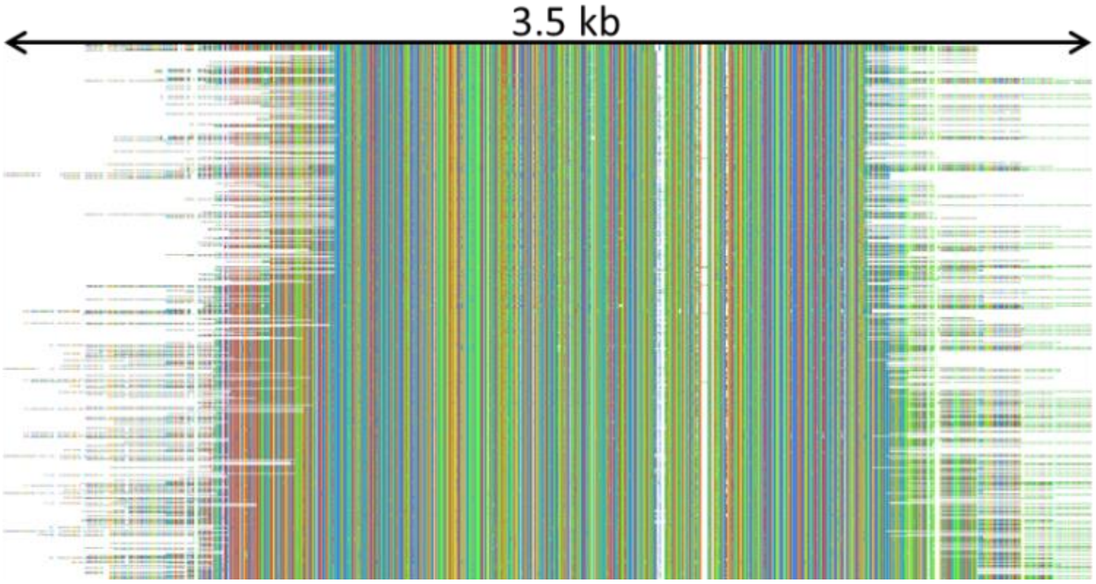

Supplementary Fig. 36 Distribution of Caulimoviridae along the 12 pedunculate oak chromosomes (Qrob_Chr01-12), determined with sliding windows of 300 kb and an overlap of 200 kb.

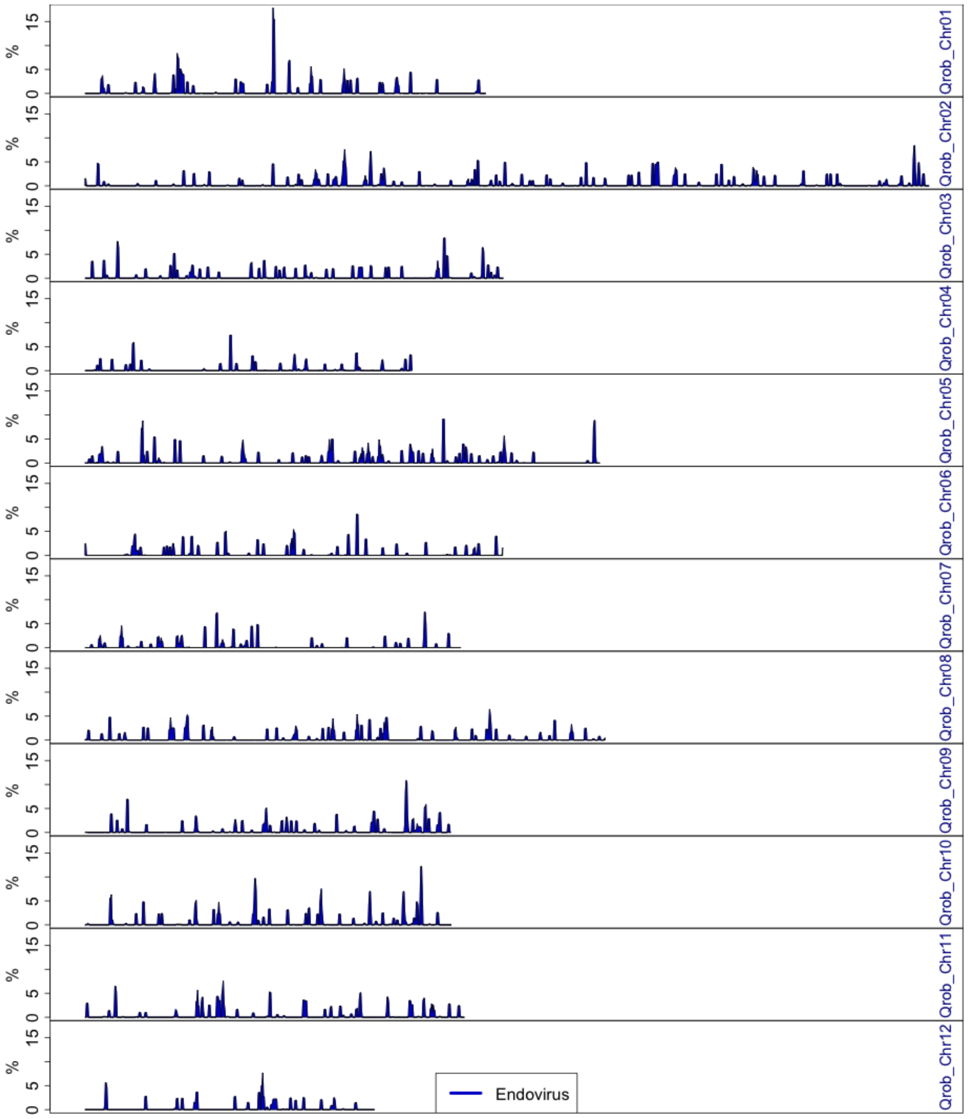

Supplementary Fig. 37 Evidence of protein functions according to annotation category: BLAST/rpsBLAST (red), domain/motifs (green), and localization/targeting-based analysis (blue).

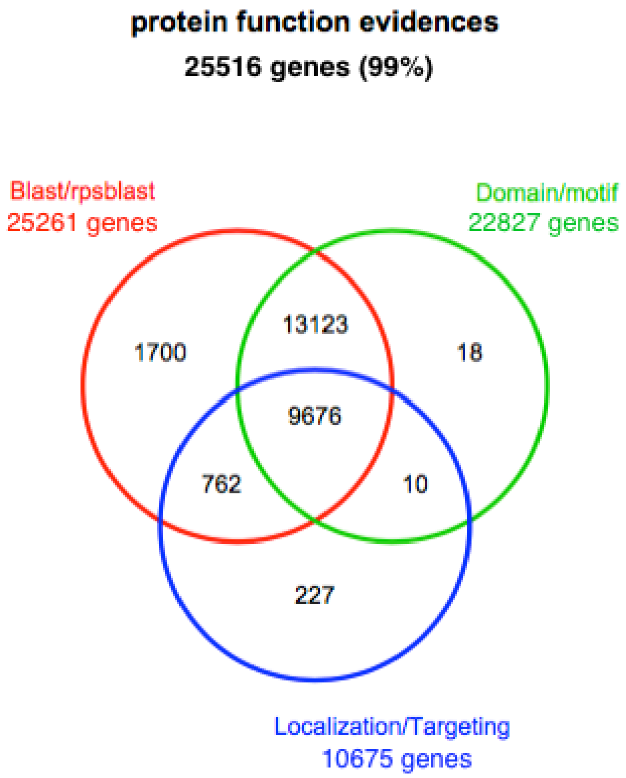

Supplementary Fig. 38 Comparison of observed divergence of TE copies from their respective consensus sequences, for different TE orders and superfamilies. DTX: Class II (DNA) TIR, DHX: Class II Helitron, RLC: Class I LTR Copia, RLG: Class I LTR Gypsy, RLX: Class I LTR other, RIX: Class I LINE, noCat: unclassified TE

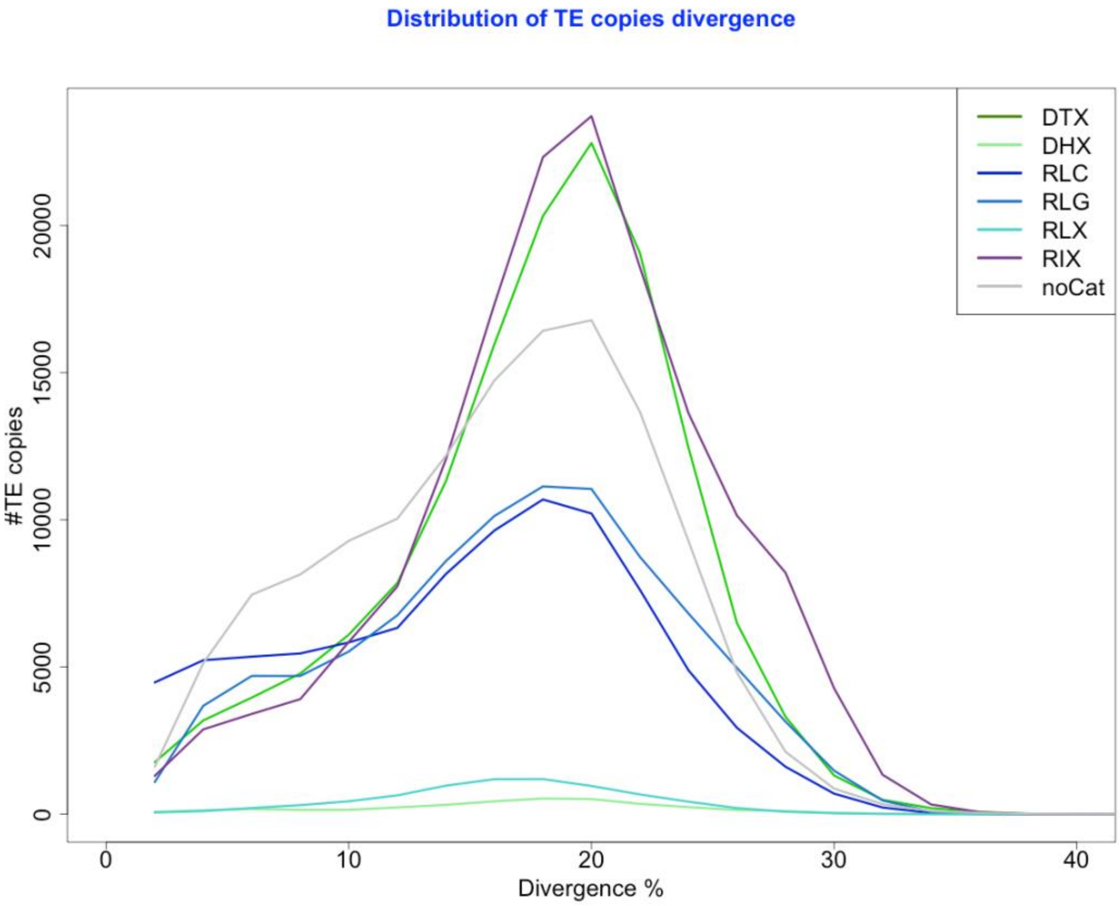

Supplementary Fig. 39 Transpositional dynamics of nine highly repeated LTR-retrotransposon families in the oak genome. Histograms represent the age distribution of the retrotransposons, showing the asynchronism of retrotranspositional activity in pedunculate oak over the last six million years. The magenta curves represent local density estimates. The title of each histogram indicates the family name and its number of copies.

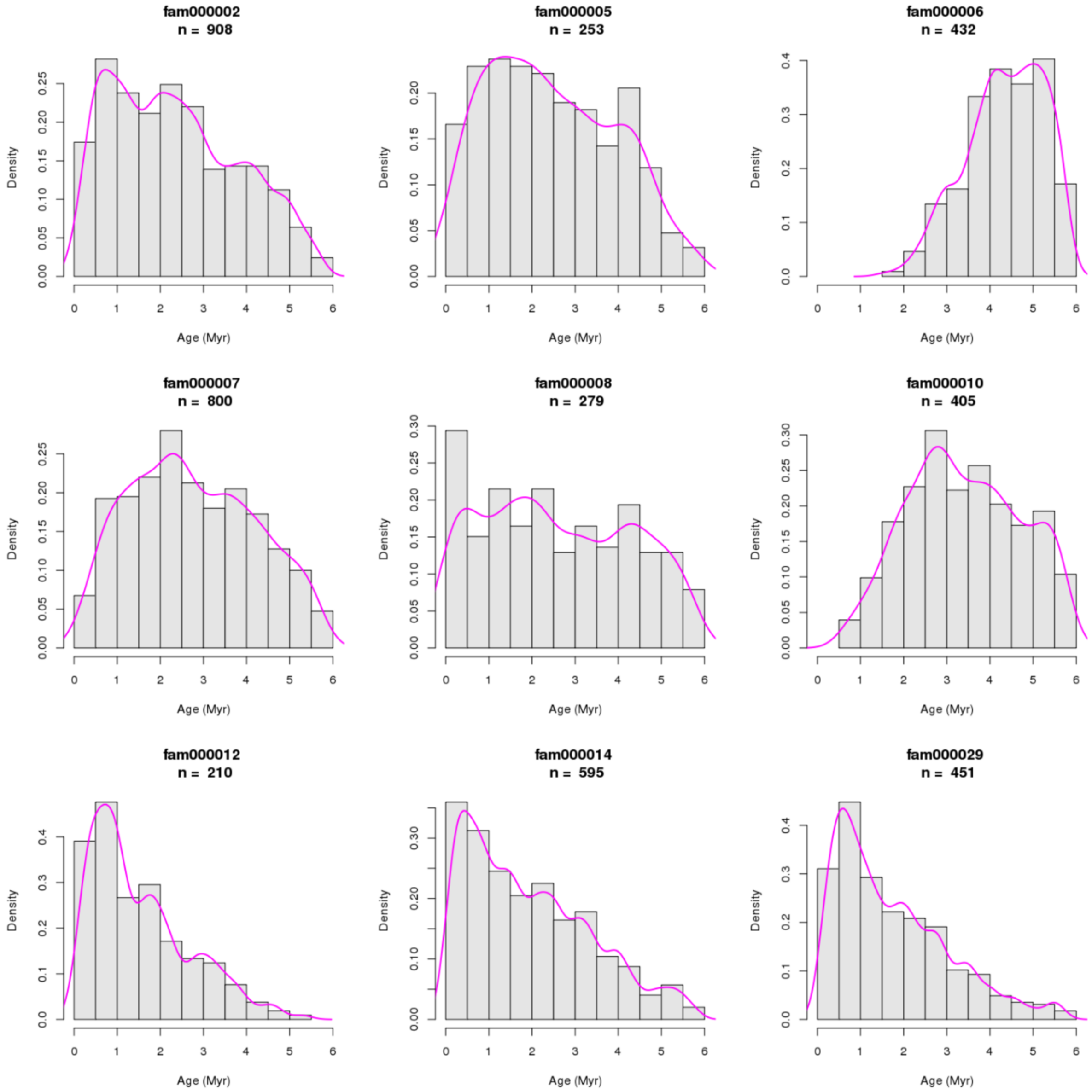

Supplementary Fig. 40 Distribution of TE (red area), genes (green area) and GC content (blue line) along the 12 chromosomes (Qrob_Chr01-12) of the pedunculate oak genome.

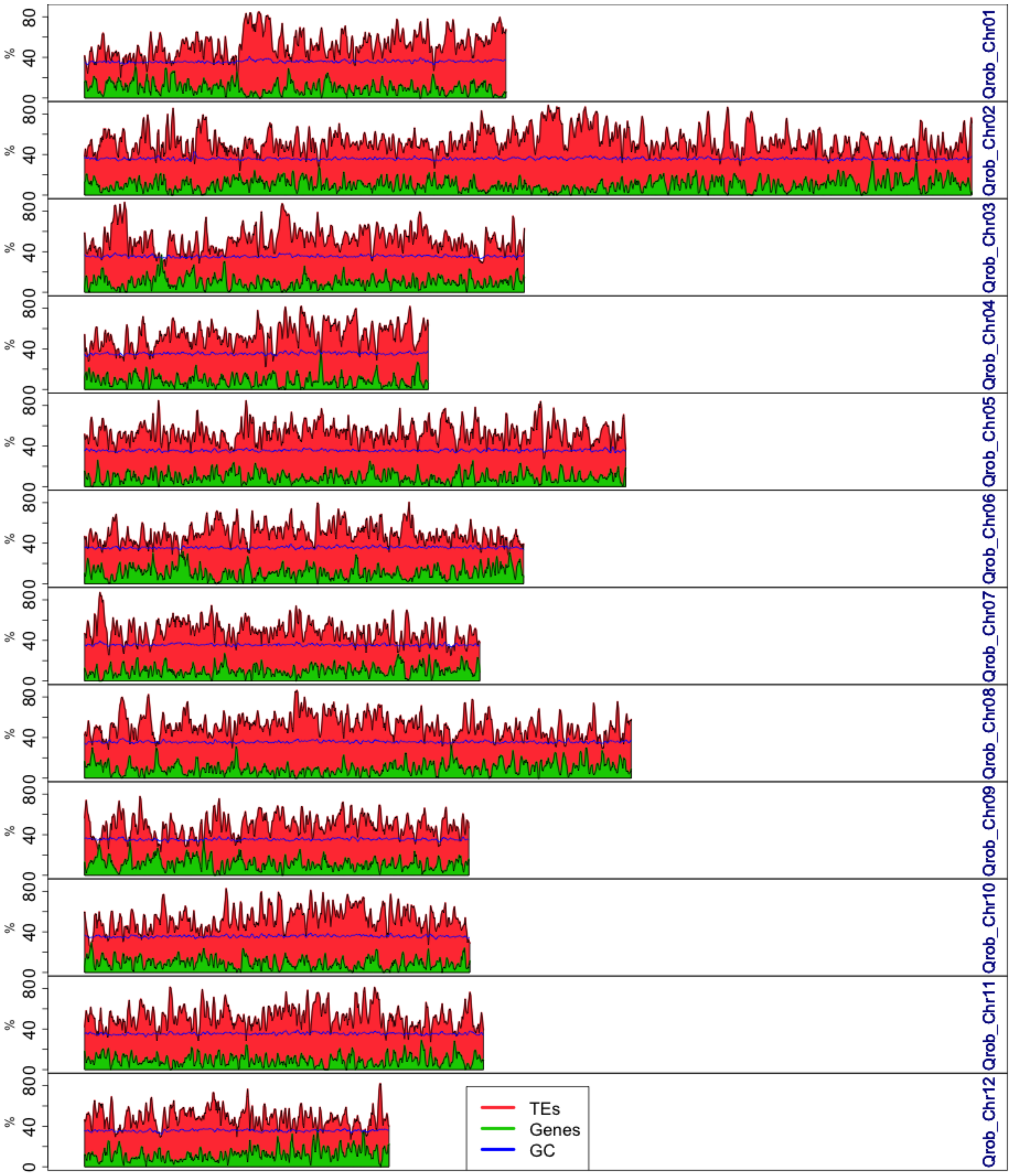

Supplementary Fig. 41 Distribution of the Gypsy (light-blue) and Copia (dark-blue) superfamily of ClassI-LTR retrotransposons along the 12 chromosomes (Qrob_Chr01-12) of the pedunculate oak genome sequence.

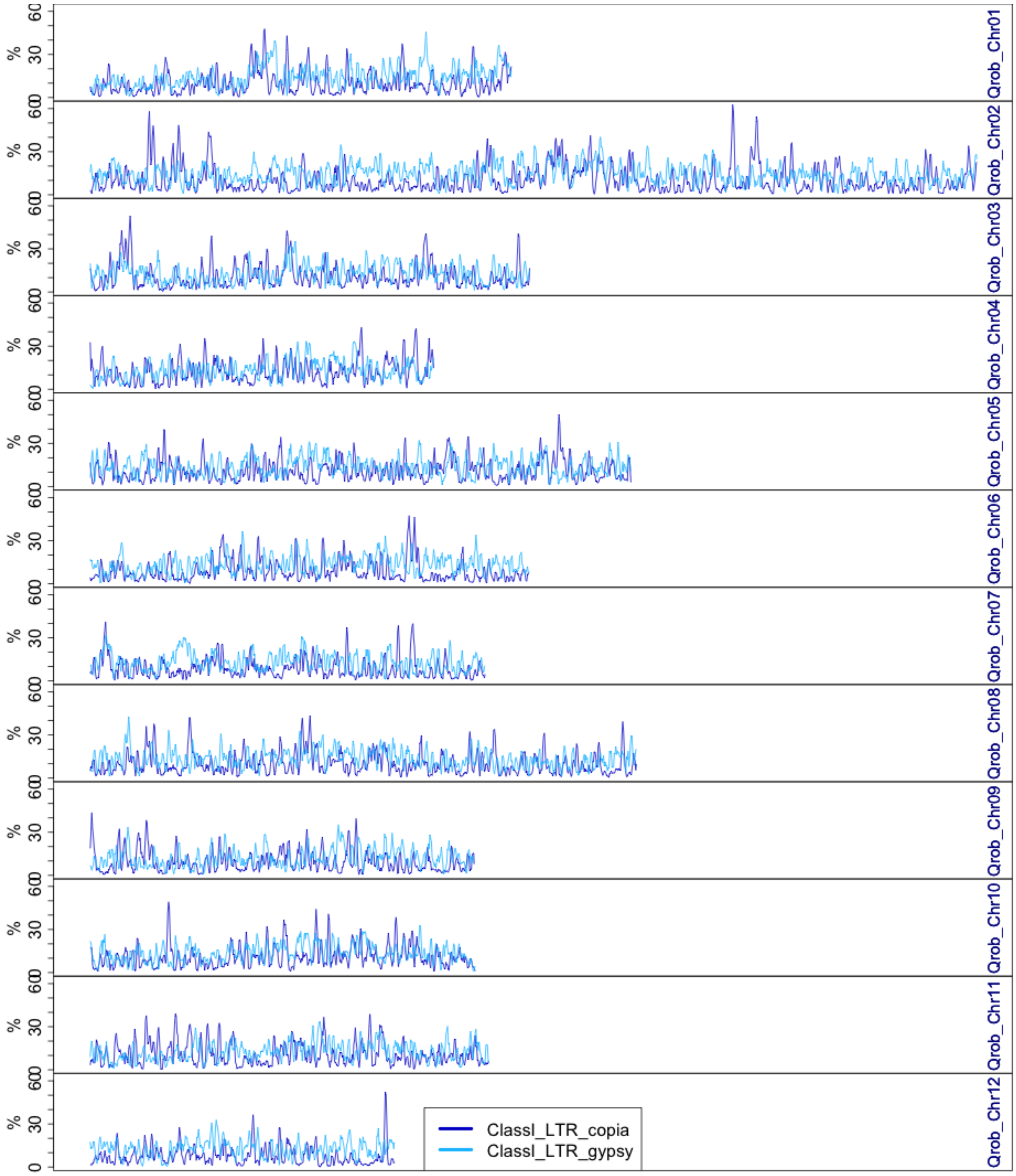

Supplementary Fig. 42 Comparison of gene-to-closest TE distance between genes from expanded gene families (n=5,433 genes) and genes from unchanged gene families (n=15,166 genes). **(A**) Two classes of distance [1-500bp], [501-5000bp] Pearson's Chi-squared test with Yates' continuity correction: *P*-value = $2.2e^{-16}$. (**B**) 10 classes of distance [1-500 bp], [501-1000 bp]...[4501-5000 bp] Pearson's Chi-squared test: *P*-value = $2.2e^{-16}$.

A **B**

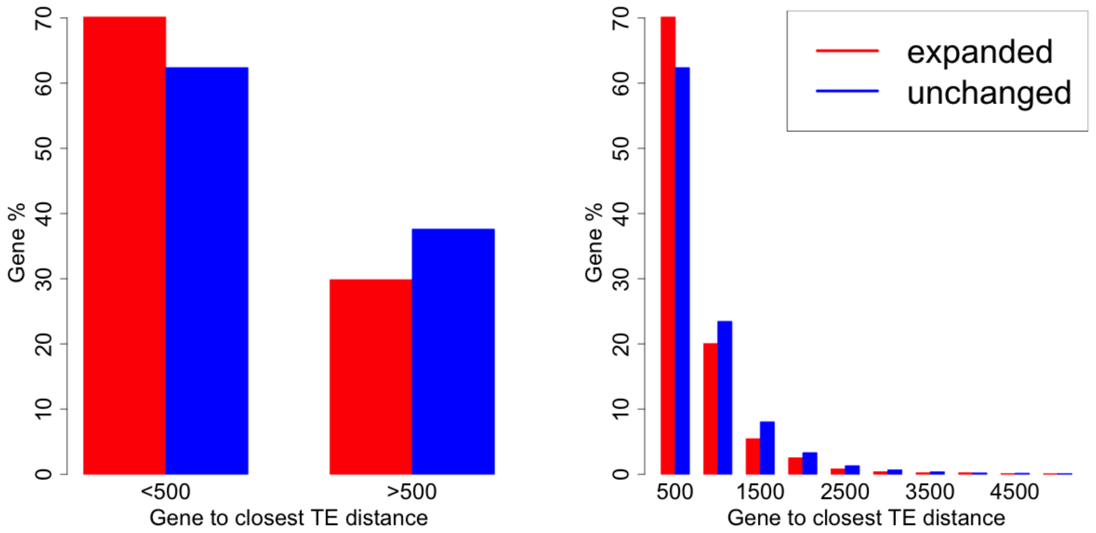

Supplementary Fig. 43 Comparison of gene-to-closest TE distance between sets of tandemly duplicated genes (TDG; n=8,532 genes) and single copy genes (SG; n=6,325 genes). (**A)** Two classes of distance [1-500 bp], [501-5000 bp] Pearson's Chi-squared test with Yates' continuity correction: *P*-value = 2.2e^{-16}. (**B)** 10 classes of distance [1-500 bp], [501-1000 bp]...[4501-5000 bp] Pearson's Chi-squared test: *P*-value = 1.5e10^{-14}.

A **B**

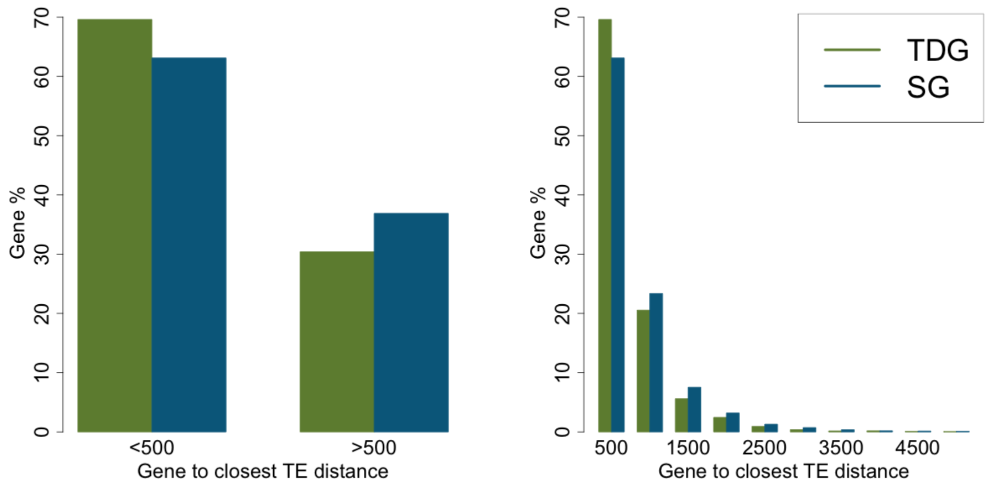

Supplementary Fig. 44 Phylogenetic analysis of aquaporins. (**A**) Proteins are from *Quercus robur* (Qrob, dot), *Arabidopsis thaliana* (At[58]) and *Populus trichocarpa* (Pt[61]). Protein sequences were compared in ClustalW analyses, and a consensus Neighbor-Joining tree was generated in MEGA6 (bootstrap: 500 replicates, distance based on number of differences method excluding gaps). (**B**) Exon-intron structure of *Quercus robur* aquaporins as displayed by GSDS2.0 (http://gsds.cbi.pku.edu.cn/).

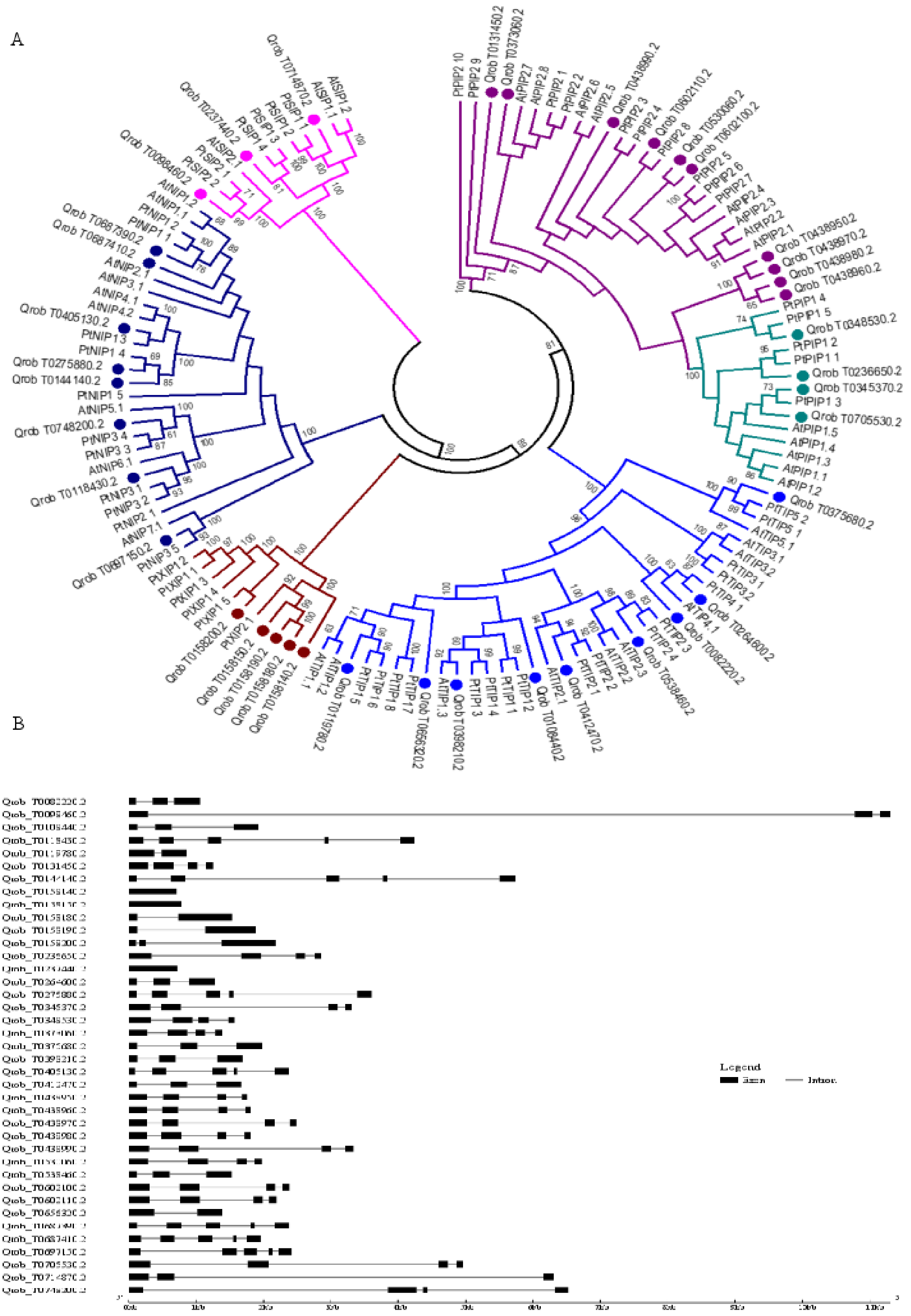

Supplementary Fig. 45 Phylogenetic analysis of R2R3-MYB. R2R3-MYB proteins are from *Quercus robur, Populus trichocarpa, Eucalyptus grandis, Vitis vinifera, Arabidopsis thaliana* and *Oriza sativa*. The R2R3-MYB proteins selected for *E. grandis, V. vinifera, A. thaliana* and *O. sativa* are the same as those used by Soler et al.[63]*, except for LOC_Os01g62410,* which was not used. *For P. trichocarpa,* the *MYB* proteins were selected as described by Chai et al.[172], except for Potri.003G1238001, Potri.015G143400.1, Potri.013G046300.1, Potri.019G018400.2, Potri.006G097300.1, Potri.016G112300.1, and Potri.008G064200.1, which were not included. These sequences were discarded after manual inspection. R2R3-MYBs were aligned using MAFFT with the FFT-NS-i algorithm[173], and a neighbour-joining phylogenetic tree with 1000 bootstrap replicates was constructed with MEGA5[174], with the Jones–Taylor–Thornton substitution model used to calculate the evolutionary distances, a rate of variation between sites with a gamma distribution of 1, and the comparison of sequences with the complete deletion method. Bootstrap values are shown next to the branches. The tree is drawn to scale, with branch lengths calculated on the basis of the number of amino-acid substitutions per site. Each triangle represents a R2R3-MYB subgroup. Subgroup names are included next to each clade, together with a short name to simplify nomenclature. The total number of R2R3-MYB genes of each species, as a whole and for each subgroup, is also included. Subgroups expanded in woody plants are highlighted in light orange or red.

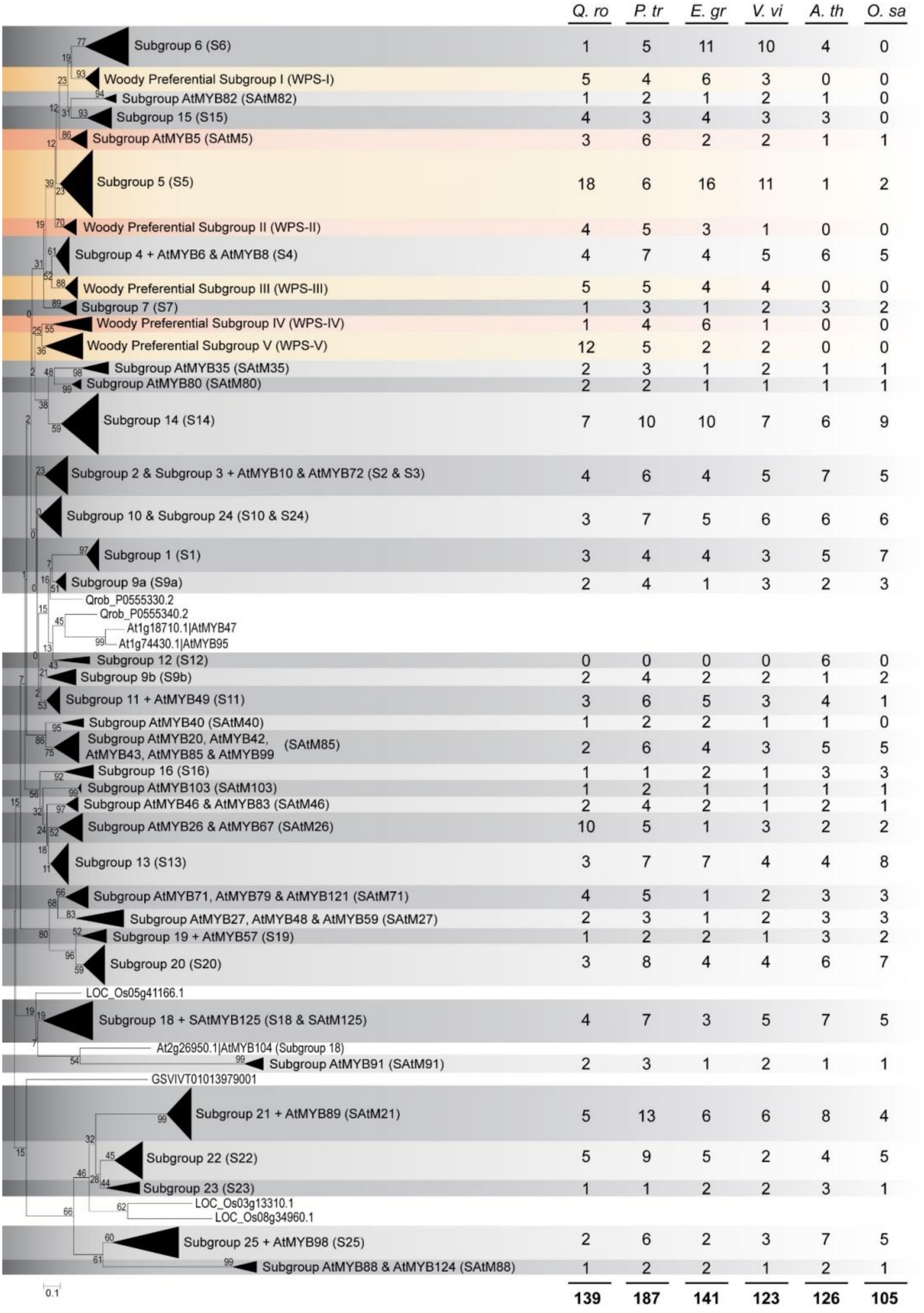
Q. ro P. tr E. gr V. vi A. th O. sa
Subgroup 6 (S6) 1 5 11 10 4 0
Woody Preferential Subgroup I (WPS-I) 5 4 6 3 0 0
Subgroup AtMYB82 (SAtM82) 1 2 1 2 1 0
Subgroup 15 (S15) 4 3 4 3 3 0
Subgroup AtMYB5 (SAtM5) 3 6 2 2 1 1
Subgroup 5 (S5) 18 6 16 11 1 2
Woody Preferential Subgroup II (WPS-II) 4 5 3 1 0 0
Subgroup 4 + AtMYB6 & AtMYB8 (S4) 4 7 4 5 6 5
Woody Preferential Subgroup III (WPS-III) 5 5 4 4 0 0
Subgroup 7 (S7) 1 3 1 2 3 2
Woody Preferential Subgroup IV (WPS-IV) 1 4 6 1 0 0
Woody Preferential Subgroup V (WPS-V) 12 5 2 2 0 0
Subgroup AtMYB35 (SAtM35) 2 3 1 2 1 1
Subgroup AtMYB80 (SAtM80) 2 2 1 1 1 1
Subgroup 14 (S14) 7 10 10 7 6 9
Subgroup 2 & Subgroup 3 + AtMYB10 & AtMYB72 (S2 & S3) 4 6 4 5 7 5
Subgroup 10 & Subgroup 24 (S10 & S24) 3 7 5 6 6 6
Subgroup 1 (S1) 3 4 4 3 5 7
Subgroup 9a (S9a) 2 4 1 3 2 3
Qrob_P0555330.2
Qrob_P0555340.2
At1g18710.1|AtMYB47
At1g74430.1|AtMYB95
Subgroup 12 (S12) 0 0 0 0 6 0
Subgroup 9b (S9b) 2 4 2 2 1 2
Subgroup 11 + AtMYB49 (S11) 3 6 5 3 4 1
Subgroup AtMYB40 (SAtM40) 1 2 2 1 1 0
Subgroup AtMYB20, AtMYB42, AtMYB43, AtMYB85 & AtMYB99 (SAtM85) 2 6 4 3 5 5
Subgroup 16 (S16) 1 1 2 1 3 3
Subgroup AtMYB103 (SAtM103) 1 2 1 1 1 1
Subgroup AtMYB46 & AtMYB83 (SAtM46) 2 4 2 1 2 1
Subgroup AtMYB26 & AtMYB67 (SAtM26) 10 5 1 3 2 2
Subgroup 13 (S13) 3 7 7 4 4 8
Subgroup AtMYB71, AtMYB79 & AtMYB121 (SAtM71) 4 5 1 2 3 3
Subgroup AtMYB27, AtMYB48 & AtMYB59 (SAtM27) 2 3 1 2 3 3
Subgroup 19 + AtMYB57 (S19) 1 2 2 1 3 2
Subgroup 20 (S20) 3 8 4 4 6 7
LOC_Os05g41166.1
Subgroup 18 + SAtMYB125 (S18 & SAtM125) 4 7 3 5 7 5
At2g26950.1|AtMYB104 (Subgroup 18)
Subgroup AtMYB91 (SAtM91) 2 3 1 2 1 1
GSVIVT01013979001
Subgroup 21 + AtMYB89 (SAtM21) 5 13 6 6 8 4
Subgroup 22 (S22) 5 9 5 2 4 5
Subgroup 23 (S23) 1 1 2 2 3 1
LOC_Os03g13310.1
LOC_Os08g34960.1
Subgroup 25 + AtMYB98 (S25) 2 6 2 3 7 5
Subgroup AtMYB88 & AtMYB124 (SAtM88) 1 2 2 1 2 1
0.1
139 187 141 123 126 105

Supplementary Fig. 46 Classification and percentage of pedunculate oak R2R3-MYB genes as a function of their mode of duplication and expansion in woody perennials. R2R3-MYB genes were first classified into three categories on the basis of duplication mode (see online methods): tandem duplicated genes (TDGs), long distance-duplicated genes (LDGs), and singleton genes (SGs). The TDGs and LDGs were further classified into genes belonging or not belonging to subgroups expanded in woody perennials.

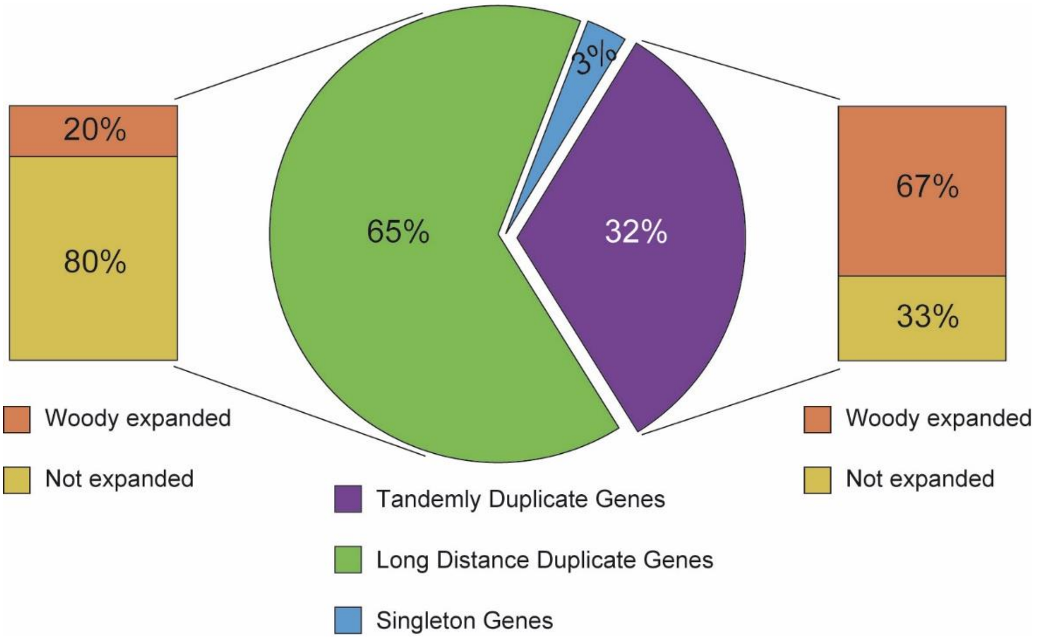

Supplementary Fig. 47 Phylogenetic analysis of SWEET. Sequences were aligned by ClustalW and a tree was constructed with the neighbor-joining method. The different clades of SWEET genes defined by Chen et al.[71] are color-coded. *Qrob* indicates predicted pedunculate oak (*Quercus robur*) polypeptides, and the reference species are abbreviated as follows: *Arabidopsis thaliana* (At); *Solanum tuberosum* (St); *Eucalyptus grandis* (Eg); *Malus domestica* (Md). The tree is rooted on SWEET homologs from *Chlamydomonas reinhardtii* (Cr). The symbols indicate pedunculate oak genes differentially expressed during interactions with the ectomycorrhizal fungi *Piloderma croceum* and *Tuber magnatum*, the ectomycorrhiza helper bacterium *Streptomyces* sp. AcH 505, and the causal agent of oak powdery mildew *Erysiphe alphitoides.* For phylogenetic analysis, protein-coding sequences from *Arabidopsis thaliana*, *Solanum tuberosum*, *Eucalyptus grandis*, *Malus domestica* and *Chlamydomonas reinhardtii* (non-plant reference) were obtained from the NCBI (http://www.ncbi.nlm.nih.gov), and protein-coding sequences from oak were extracted from the haplome. Phylogenetic distances between the SWEET proteins were calculated from a multiple sequence alignment (ClustalW), by the neighbor-joining method (MEGA6), with bootstrapping (1000 replicates).

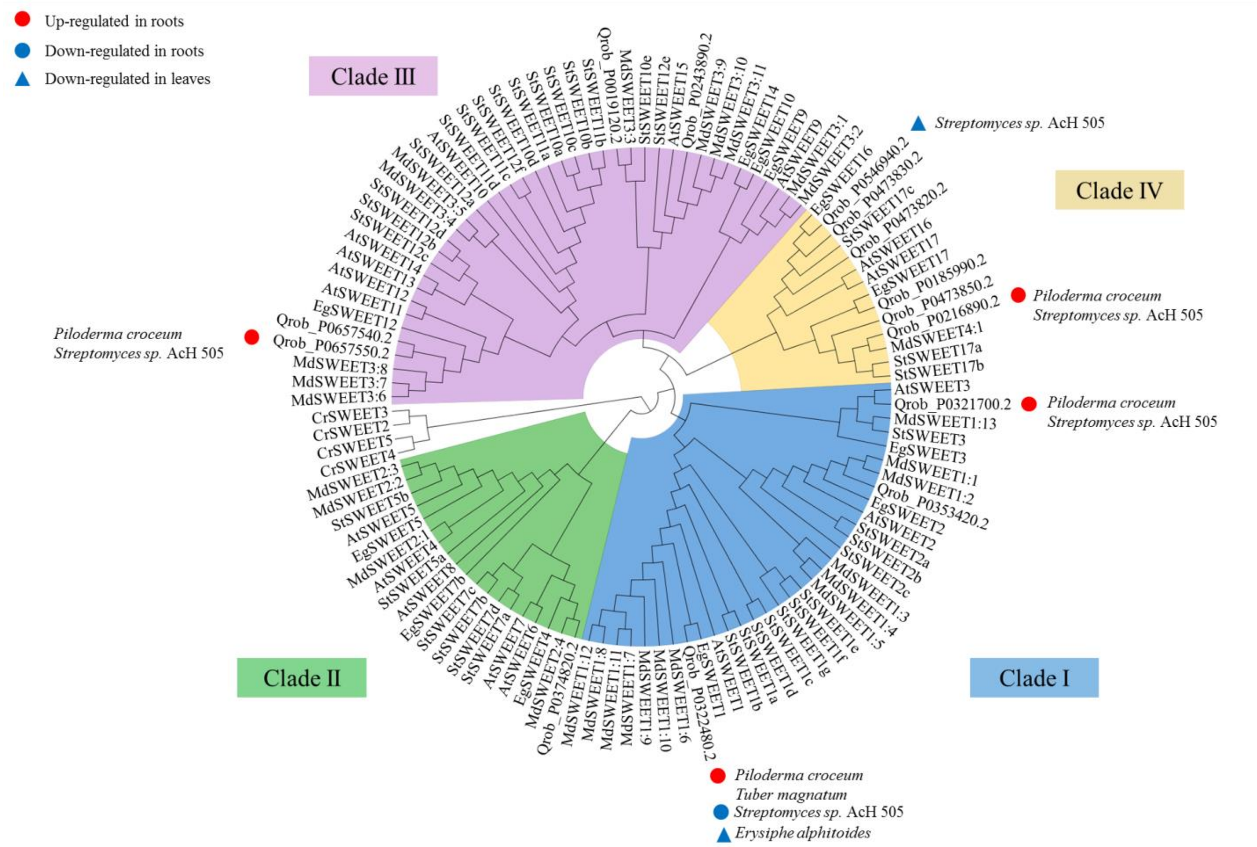

Supplementary Fig. 48 Phylogenetic analysis of GSTUs. Sequences encoding GSTUs from *Quercus robur*, *Populus trichocarpa*, *Arabidopsis thaliana*, *Oryza sativa* and *Sorghum bicolor* were retrieved from Phytozome (https://phytozome.jgi.doe.gov/pz/portal.html). Sequences were then aligned with Clustal-Omega[175]. The alignment was manually adjusted with SeaView software [107] and curated with GBlocks[176]. The unrooted phylogenetic tree was constructed with BioNJ[177] in Seaview and further edited with FigTree software (http://tree.bio.ed.ac.uk/software/figtree/). The robustness of the branches was assessed by the bootstrap method with 1000 replications (not shown). Sequences corresponding to *Quercus robur*, *Populus trichocarpa*, *Arabidopsis thaliana*, *Oryza sativa* and *Sorghum bicolor* GSTUs are shown in red, blue, green, cyan and pink, respectively. The expanded clusters identified in orthoMCL analysis are highlighted on red branches. Given its considerable divergence, the Qrob_P0196930.2 protein annotated as GSTU was removed from the analysis.

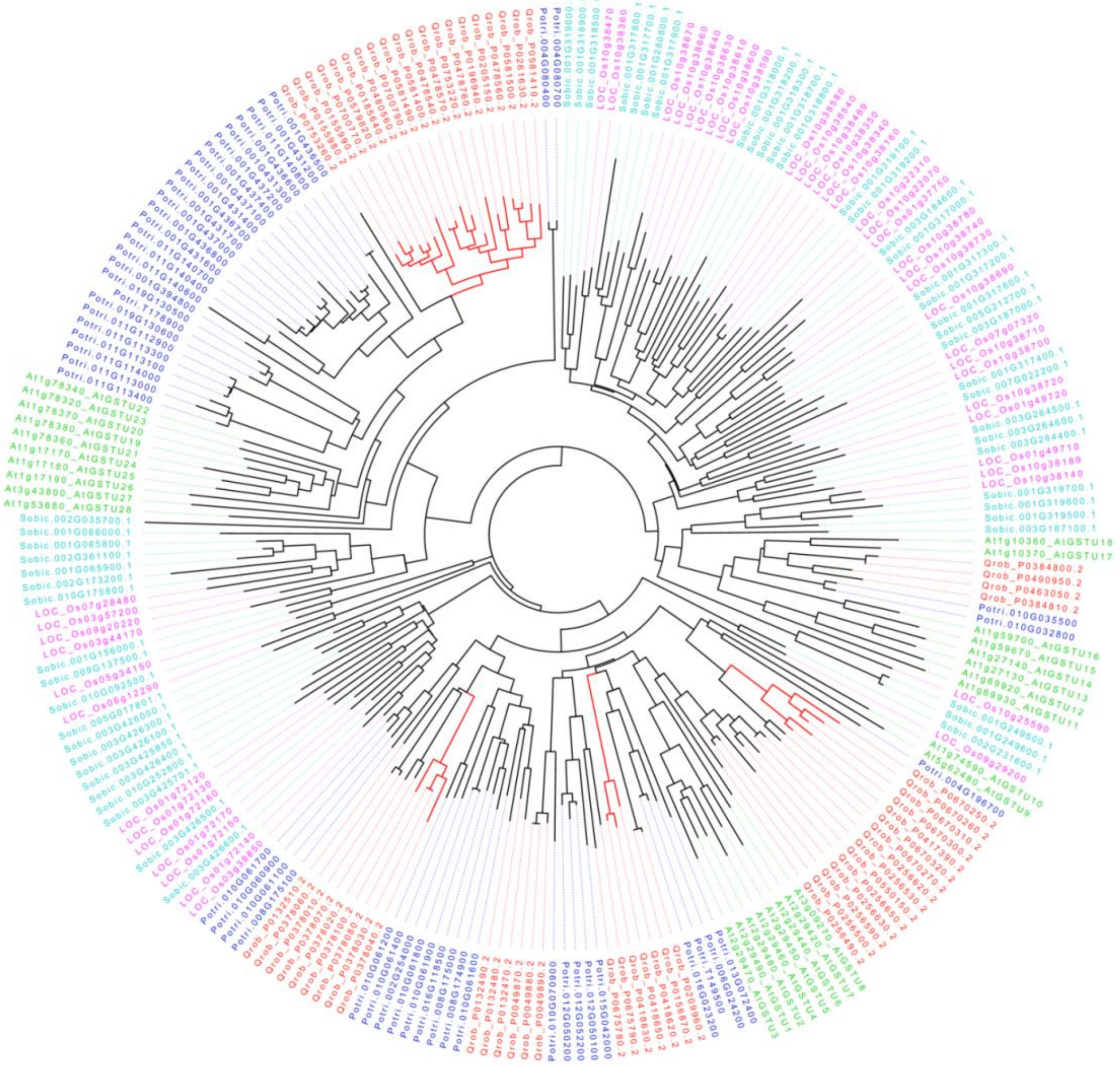

Supplementary Fig. 49 Phylogenetic analysis of MLO. The proteins used are from *Quercus robur* (n=19 regular genes and 7 unreliable genes), *Prunus persica* (n=18)[92] and *Arabidopsis thaliana* (n=7, TAIR - https://www.arabidopsis.org/). The analysis was performed on the Phylogeny.fr platform[178], as follows: alignment with MUSCLE (v3.8.31) configured for highest accuracy; removal of ambiguous regions (i.e. containing gaps and/or poorly aligned) with Gblocks (v0.91b) using the following parameters (minimum length of a block after gap cleaning: 10; no gap positions were allowed in the final alignment; all segments with contiguous nonconserved positions of more than eight residues were rejected; minimum number of sequences for a flanking position: 85%); reconstruction of the phylogenetic tree with the maximum likelihood method implemented in PhyML 3.0. The WAG substitution model was selected, assuming an estimated proportion of invariant sites of 0.003 and four gamma-distributed rate categories to account for rate heterogeneity across sites. The gamma shape parameter was estimated directly from the data (gamma=1.340). The reliability of internal branches was assessed with the aLRT test (SH-Like). Graphical representation and phylogenetic tree generation were achieved with TreeDyn (v198.3). Branch boostrap support values are displayed in blue. Clades were named according to the presence of *Arabidopsis thaliana* and *Prunus persica* proteins [92].

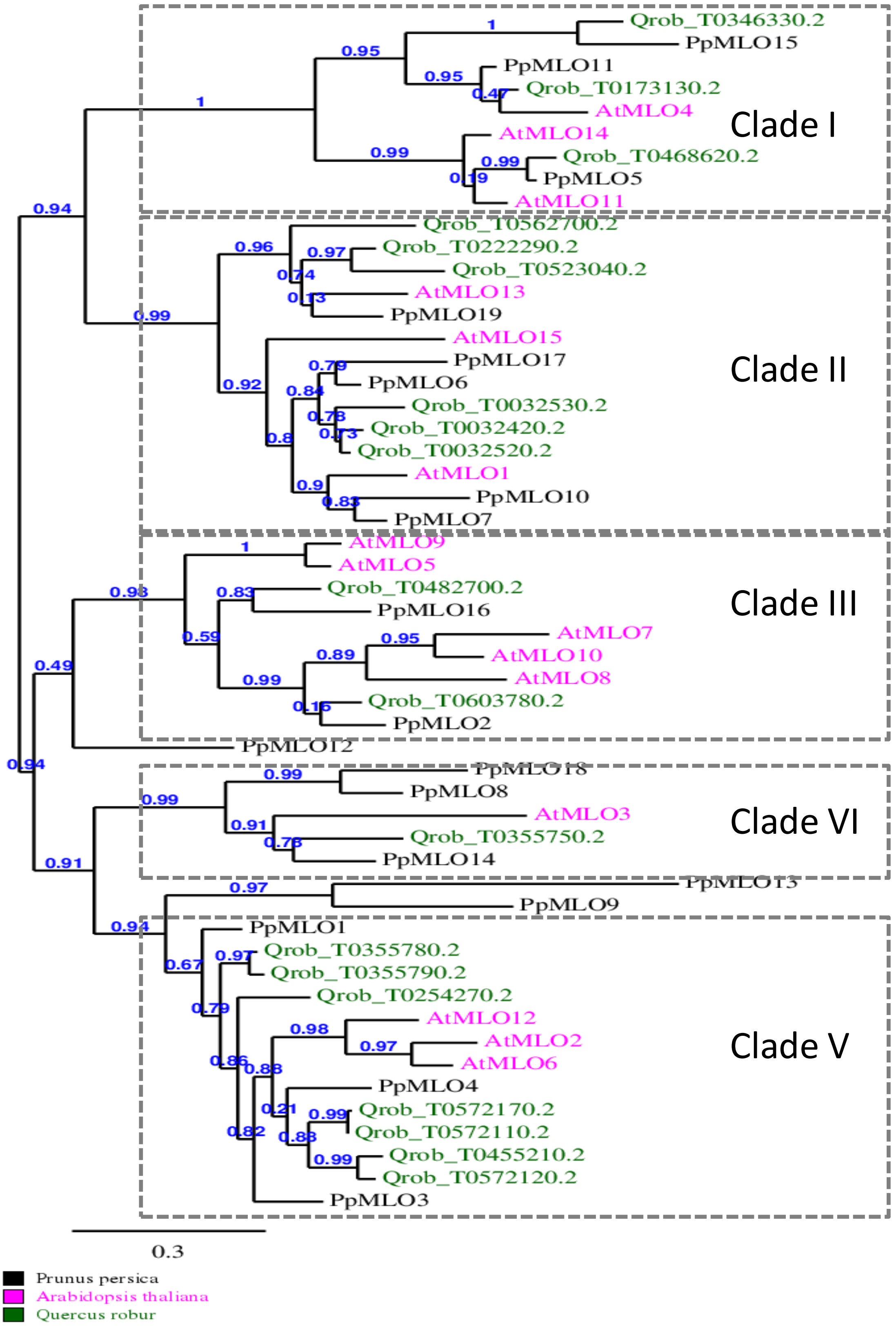
Qrob_T0346330.2
PpMLO15
PpMLO11
Qrob_T0173130.2
AtMLO4
AtMLO14
Qrob_T0468620.2
PpMLO5
AtMLO11
Clade I
Qrob_T0562700.2
Qrob_T0222290.2
Qrob_T0523040.2
AtMLO13
PpMLO19
AtMLO15
PpMLO17
PpMLO6
Qrob_T0032530.2
Qrob_T0032420.2
Qrob_T0032520.2
AtMLO1
PpMLO10
PpMLO7
Clade II
AtMLO9
AtMLO5
Qrob_T0482700.2
PpMLO16
AtMLO7
AtMLO10
AtMLO8
Qrob_T0603780.2
PpMLO2
Clade III
PpMLO12
PpMLO18
PpMLO8
AtMLO3
Qrob_T0355750.2
PpMLO14
Clade VI
PpMLO13
PpMLO9
PpMLO1
Qrob_T0355780.2
Qrob_T0355790.2
Qrob_T0254270.2
AtMLO12
AtMLO2
AtMLO6
PpMLO4
Qrob_T0572170.2
Qrob_T0572110.2
Qrob_T0455210.2
Qrob_T0572120.2
PpMLO3
Clade V
0.3
Prunus persica
Arabidopsis thaliana
Quercus robur

Supplementary Fig. 50 Biosynthesis of hydrolyzable tannins from gallic acid, via the β-glucogallin intermediate.

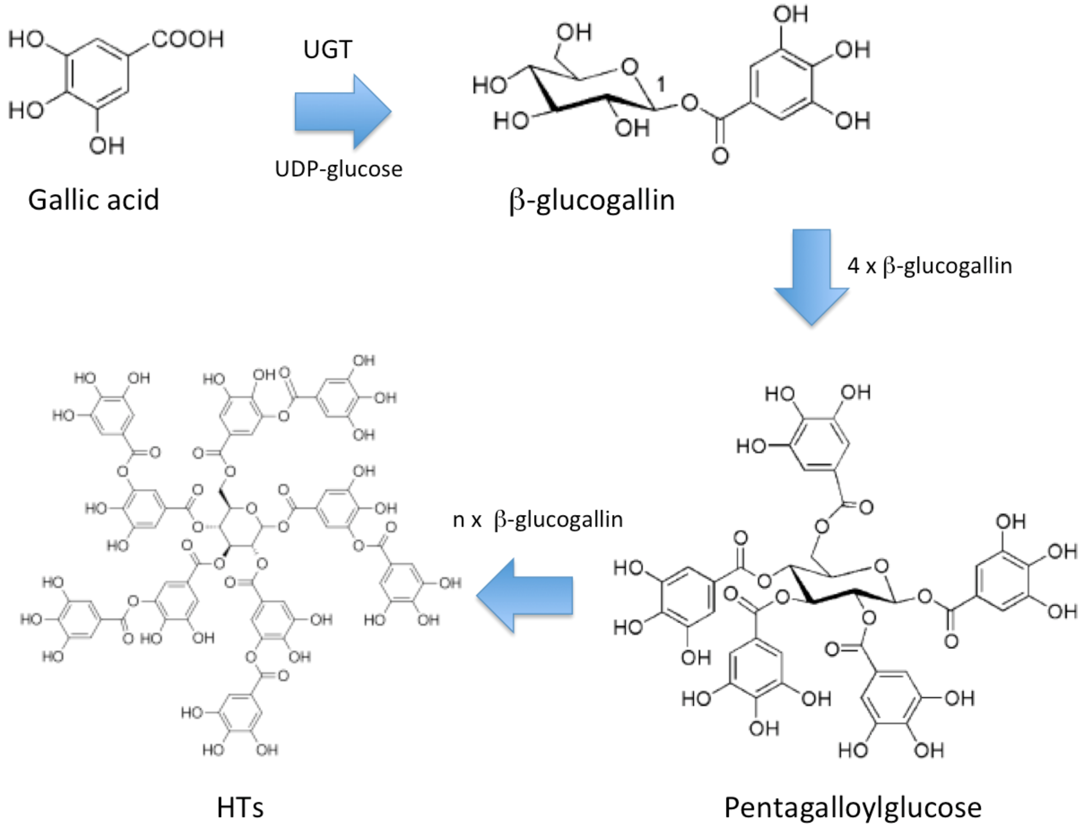

Supplementary Fig. 51 Phylogeny of oak genes potentially involved in hydrolyzable tannin biosynthesis. **(A)** Phylogeny of annotated oak genes and Arabidopsis genes involved in the chorismate pathway. Genes encoding 3-deoxy-D-arabino-heptulosonate-7-phosphate synthase (DHS), 3-dehydroquinate synthase (DHQS), 3-dehydroquinate dehydratase/shikimate 5-dehydrogenase (DHQ-SDH), shikimate kinase (SK), 5-enolpyruvylshikimate 3-phosphate synthase (EPSPS) and chorismate synthase (CS) are presented. **(B)** Phylogeny of the Arabidopsis members and annotated oak members of the UGT 74, 75, 83 and 84 families. Protein sequences were aligned using ClustalW and the UPGMA tree was drawn based on Jukes-Cantor distances (with Geneious 6.1.8).

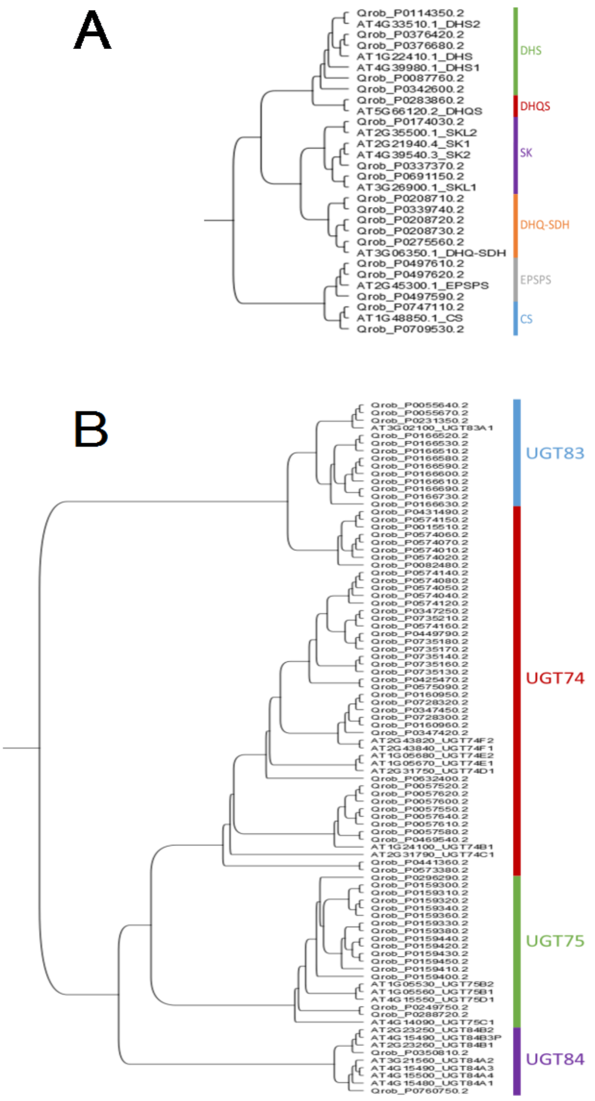

Supplementary Fig. 52 Comparative phylogenetic analysis of the laccase protein sequences from *Quercus robur*, *Populus trichocarpa*, *Eucalyptus grandis*, *Vitis vinifera*, *Arabidopsis thaliana* and *Oriza sativa*. Sequences were aligned with Clustal-Omega[175]. The alignment was manually adjusted with SeaView software[107] and curated with GBlocks[176]. *Arabidopsis* ascorbate oxidases (AO1, AO2, and AO3) were added and used as an outgroup. The phylogenetic tree was calculated with SeaView, using PhyML with the LG model and the aLRT method for branch support, NNI heuristic for optimal tree structure search and BioNJ for optimizing tree topology. The phylogenetic tree was further edited with FigTree software (http://tree.bio.ed.ac.uk/software/figtree/).

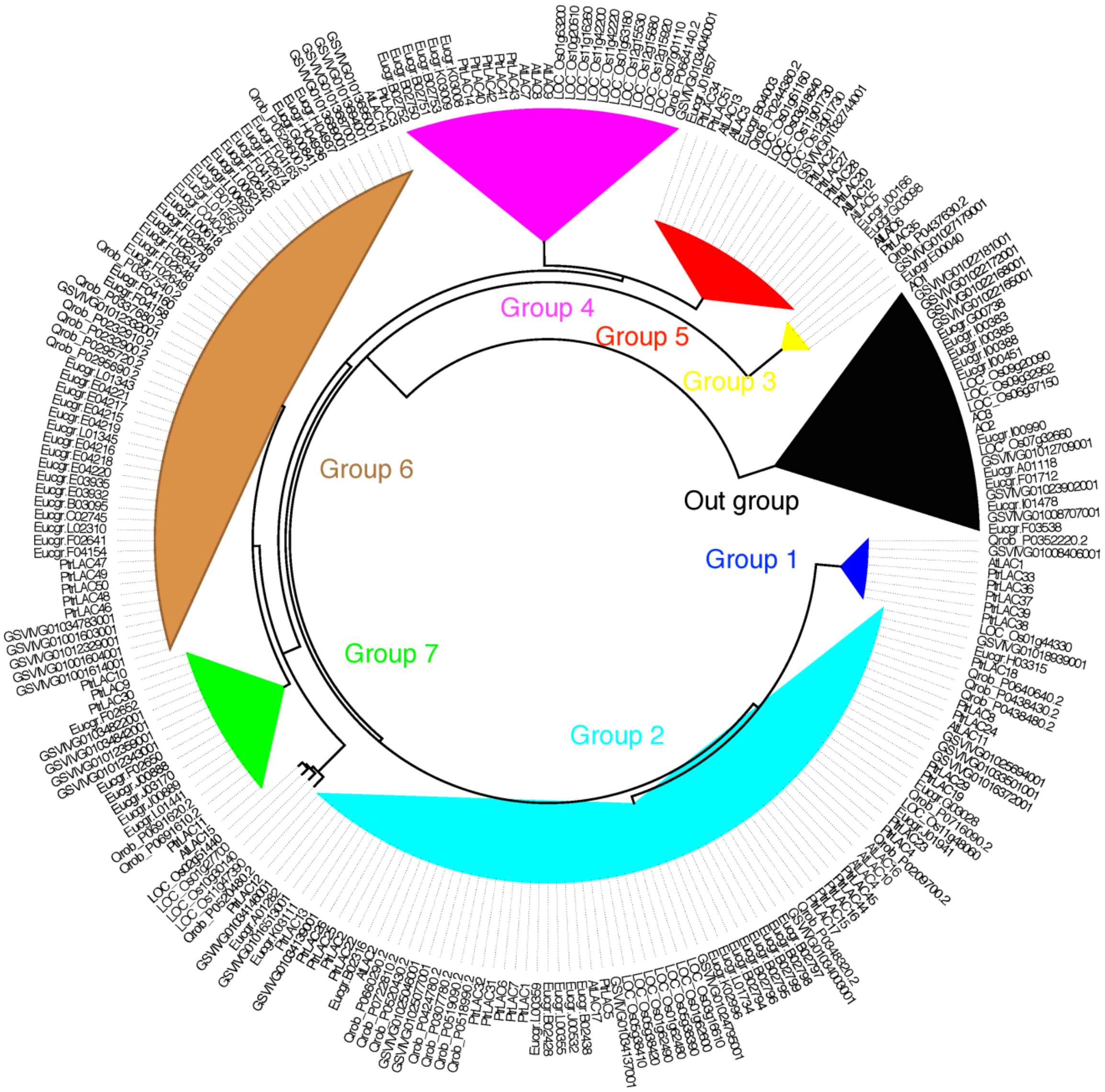

Supplementary Fig. 53 Example of a pedunculate oak specimen sampled for genetic diversity analysis by the pool-seq approach.

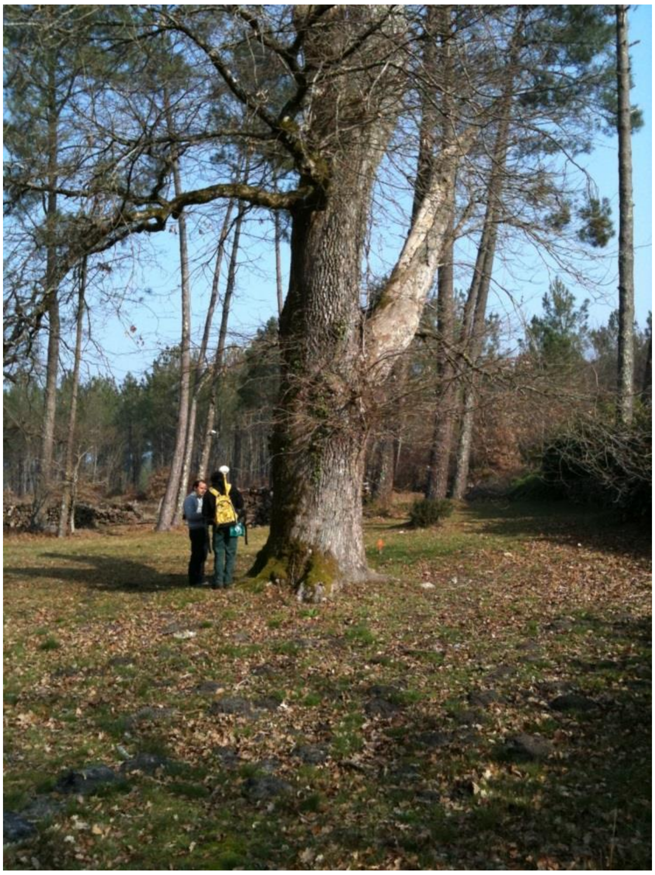

© Christophe Plomion

Supplementary Fig. 54 Distribution of π_4, π_0 and the π_0/π_4 ratio for gene families.

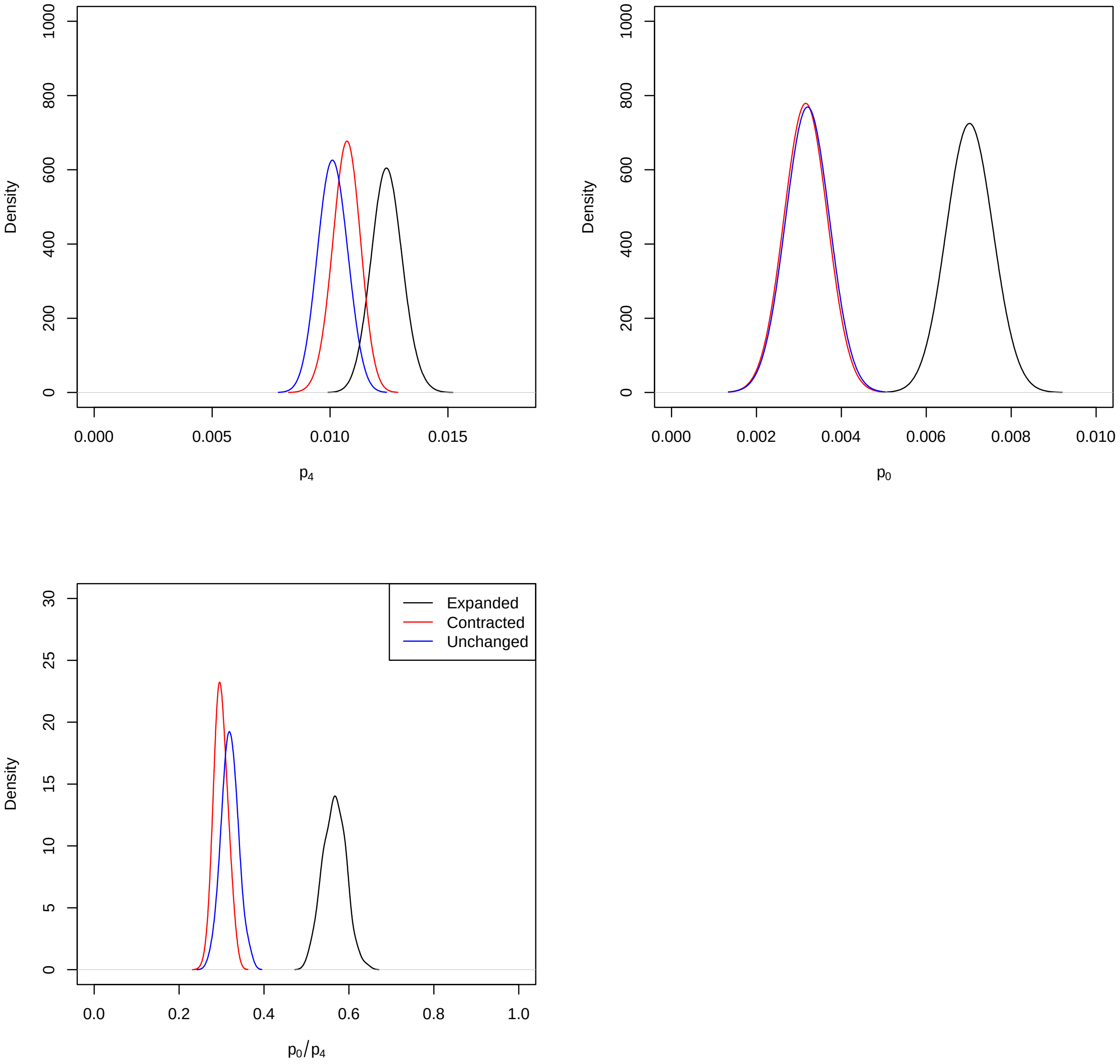

Supplementary Fig. 55 A phylogenetic tree for eudicots, based on the tree generated by Zanne et al.[138] collapsed to the order level with clade sizes denoted by triangle size. For each order, we show the number of woody and herbaceous species for which whole-genome sequences are already or will soon be available (as reported in https://genomevolution.org/wiki/index.php/Sequenced_plant_genomes as of 18 December 2016). Variable and diverse species, according to Fitzjohn et al.[149], for which genome sequences are available are highlighted in green.

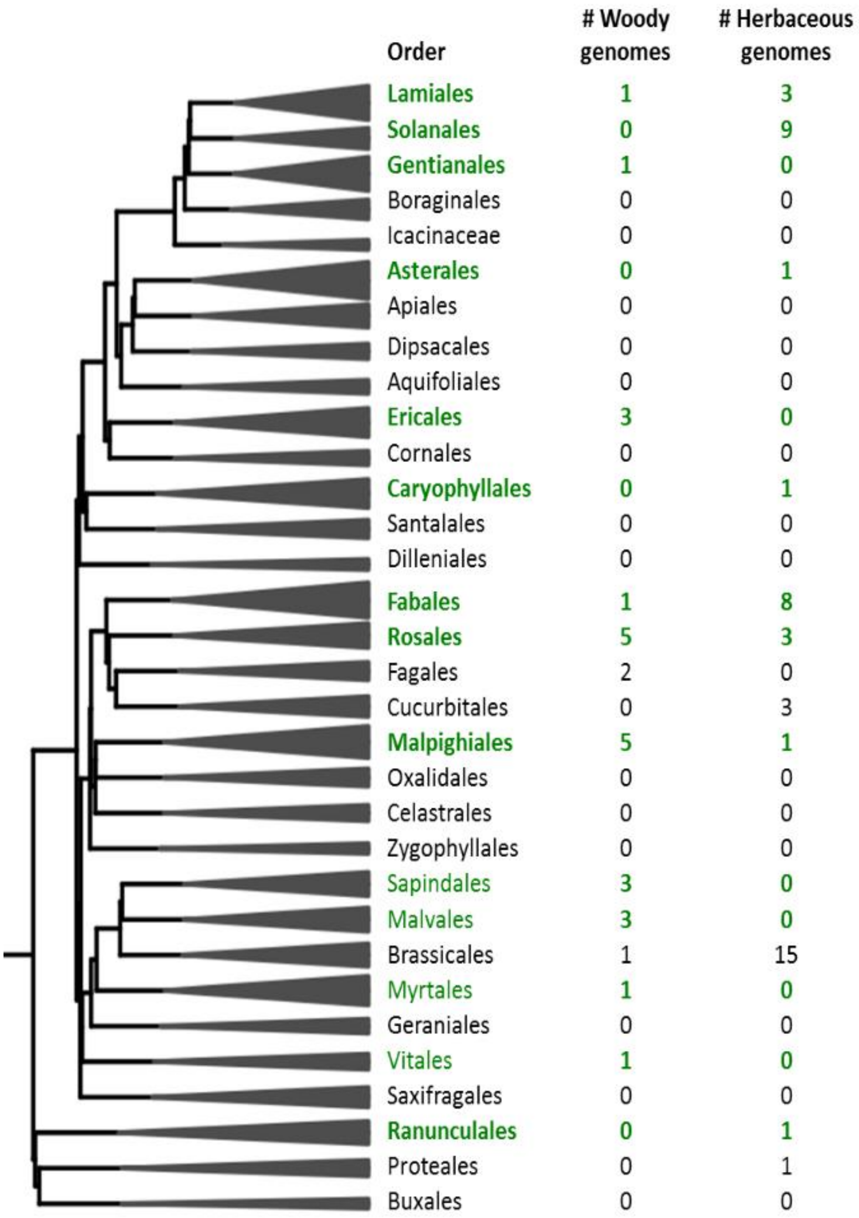

Supplementary Fig. 56 Number of GO terms per gene for the 16,820 pedunculate oak gene models with a GO.

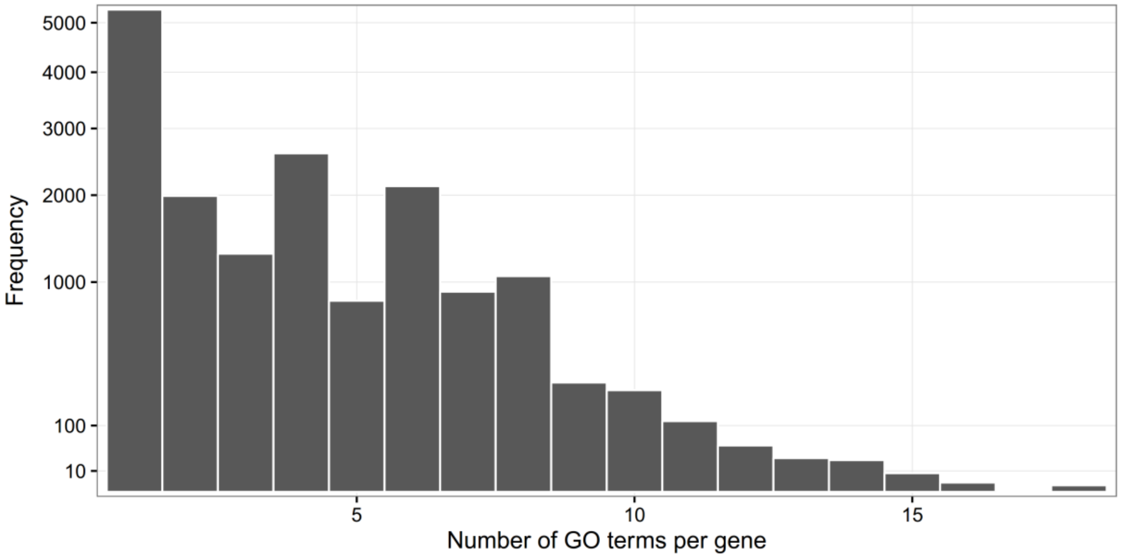

Supplementary Fig. 57 Number of genes per Gene Ontology (GO) term for the 1,722 unique pedunculate oak GO terms.

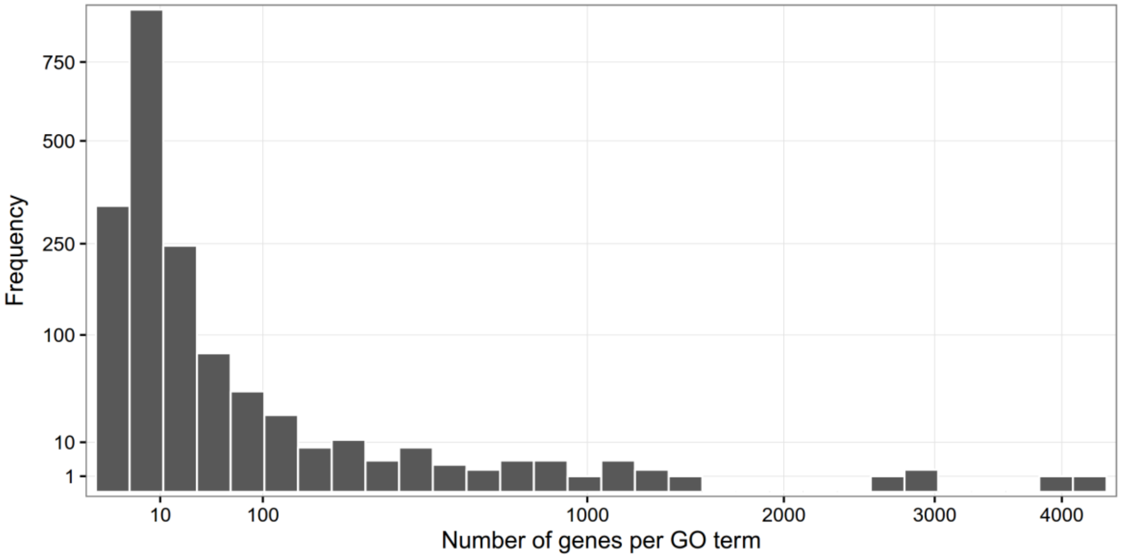

Supplementary Fig. 58 Number of genes within orthoMCL orthogroups expanded in pedunculate oak.

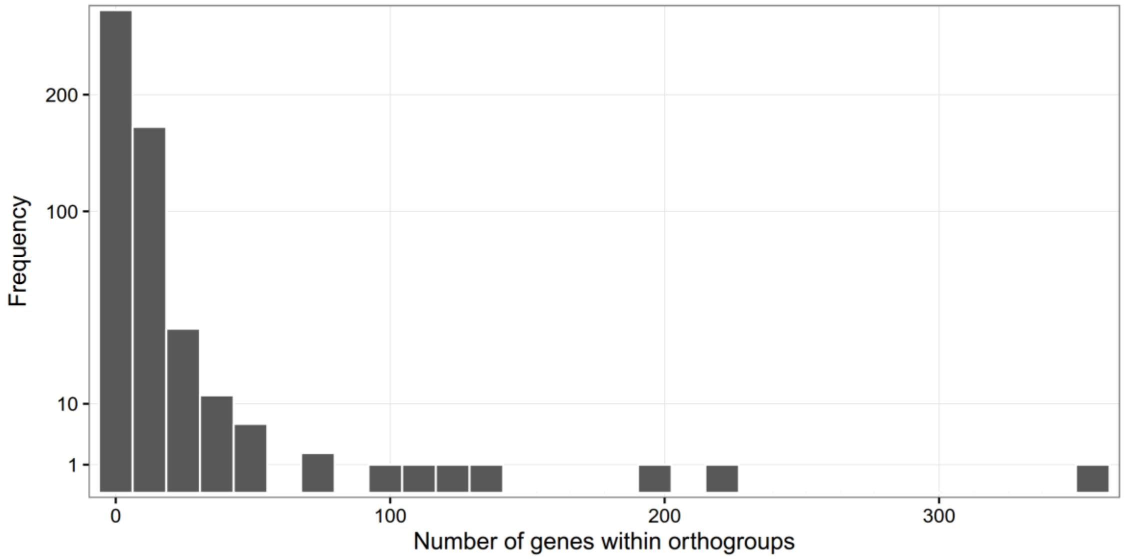

Supplementary Fig. 59 Number of gene ontology (GO) terms per orthogroup expanded in pedunculate oak, for the 1,722 unique GO terms.

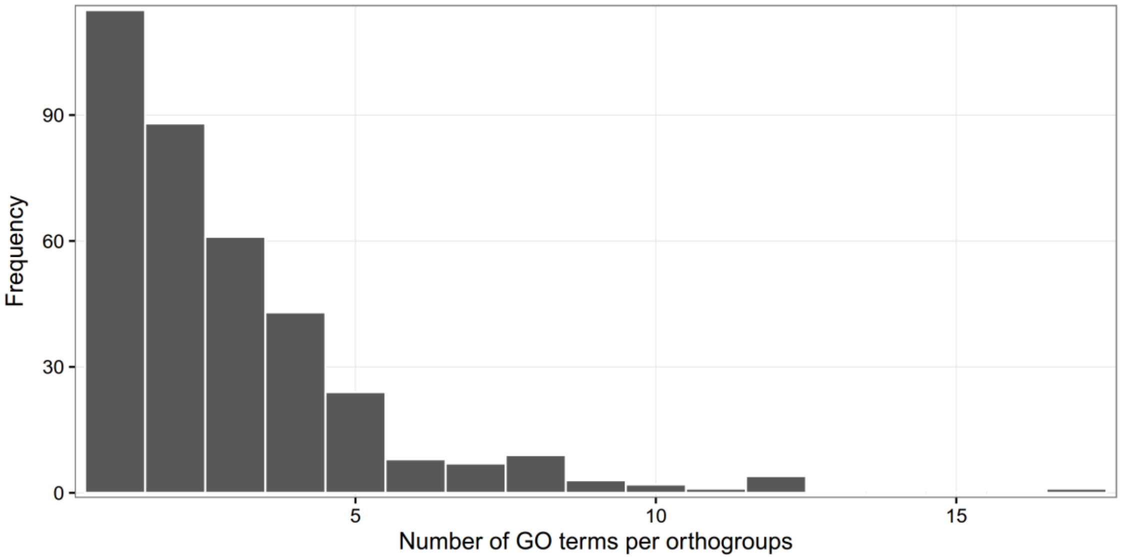

Supplementary Fig. 60 Fold-enrichment (*x*–axis) of significant gene ontology (GO) terms (*P*<0.01) of the orthogroups expanded in pedunculate oak relative to the background of the whole genome. GO representing biological processes are shown as red lines, cellular components are shown in blue and molecular functions are shown in green. The vertical dashed line represents the 1.5 threshold from which we considered interesting biologically relevant enrichments. Sample sizes are provided in **Supplementary Data Set 8** sheet #4.

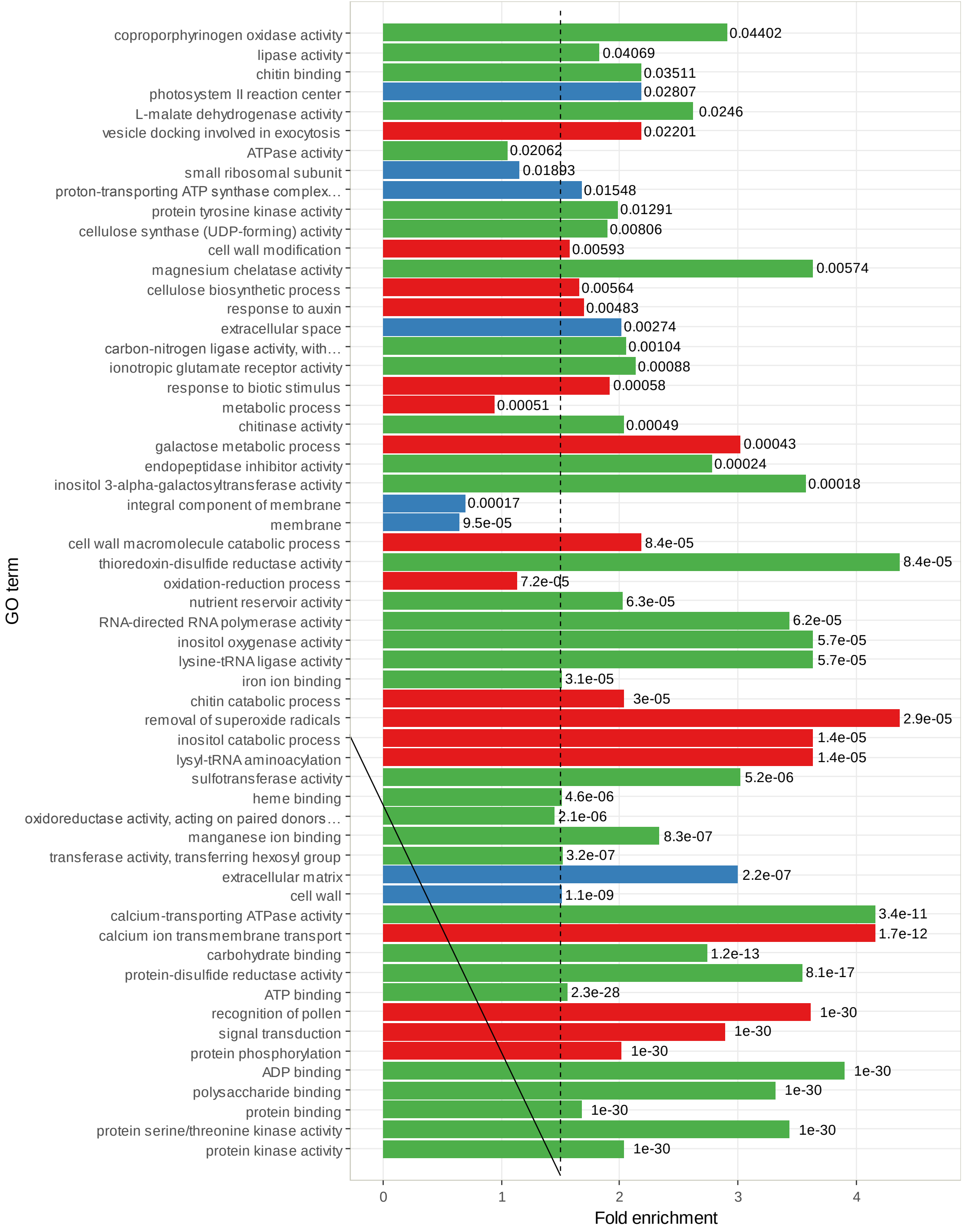

Supplement: Supplementary file 1 — Supplementary Figures 1–60, Supplementary Tables 1–43 and Supplementary References. [file 41477_2018_172_MOESM1_ESM.pdf]
